# Chaperonin genes on the rise: new divergent classes and intense duplication in human and other vertebrate genomes

**DOI:** 10.1186/1471-2148-10-64

**Published:** 2010-03-01

**Authors:** Krishanu Mukherjee, Everly Conway de Macario, Alberto JL Macario, Luciano Brocchieri

**Affiliations:** 1 Department of Molecular Genetics and Microbiology, University of Florida, College of Medicine, 1660 SW Archer Road, Gainesville, FL 32610, USA; 2 Genetics Institute, University of Florida, Cancer and Genetics Research Complex, 2033 Mowry Road, Gainesville, FL 32610, USA; 3 University of Maryland, Columbus Center, 701 East Pratt Street, Baltimore, MD 21202, USA

## Abstract

**Background:**

Chaperonin proteins are well known for the critical role they play in protein folding and in disease. However, the recent identification of three diverged chaperonin paralogs associated with the human Bardet-Biedl and McKusick-Kaufman Syndromes (BBS and MKKS, respectively) indicates that the eukaryotic chaperonin-gene family is larger and more differentiated than previously thought. The availability of complete genome sequences makes possible a definitive characterization of the complete set of chaperonin sequences in human and other species.

**Results:**

We identified fifty-four chaperonin-like sequences in the human genome and similar numbers in the genomes of the model organisms mouse and rat. In mammal genomes we identified, besides the well-known CCT chaperonin genes and the three genes associated with the MKKS and BBS pathological conditions, a newly-defined class of chaperonin genes named CCT8L, represented in human by the two sequences CCT8L1 and CCT8L2. Comparative analyses from several vertebrate genomes established the monophyletic origin of chaperonin-like MKKS and BBS genes from the CCT8 lineage. The CCT8L gene originated from a later duplication also in the CCT8 lineage at the onset of mammal evolution and duplicated in primate genomes. The functionality of CCT8L genes in different species was confirmed by evolutionary analyses and in human by expression data. Detailed sequence analysis and structural predictions of MKKS, BBS and CCT8L proteins strongly suggested that they conserve a typical chaperonin-like core structure but that they are unlikely to form a CCT-like oligomeric complex. The characterization of many newly-discovered chaperonin pseudogenes uncovered the intense duplication activity of eukaryotic chaperonin genes.

**Conclusions:**

In vertebrates, chaperonin genes, driven by intense duplication processes, have diversified into multiple classes and functionalities that extend beyond their well-known protein-folding role as part of the typical oligomeric chaperonin complex, emphasizing previous observations on the involvement of individual CCT monomers in microtubule elongation. The functional characterization of newly identified chaperonin genes will be a challenge for future experimental analyses.

## Background

Hsp60-like chaperonin proteins are well known for their role in assisting protein folding and in protecting cells from the deleterious effects of stress [1-5]. The eukaryotic cell expresses representatives of two distinct groups of chaperonin genes that are otherwise typical of bacteria (Group I) or archaea (Group II). In eukaryotes, Group I chaperonins are mostly expressed in mitochondria and chloroplasts, and Group II chaperonins are found in the eukaryotic cytosol [1,6-10]. Chaperonin proteins form typical multi-subunit double-ringed structures collectively called "chaperonins" [9-13]. The Group I chaperonins are typically formed by the products of a single gene (*groEL *in bacteria; *hsp60*/*cpn60 *in mitochondria) assembled into a 14-subunit double-ringed structure in bacteria and into a double or single-ringed structure in mitochondria [14]. Eukaryotic Group II chaperonin proteins assemble in a similar double-ringed oligomeric structure, called TRiC or CCT complex [15], composed of 16 subunits that in human are encoded by nine distinct genes (*tcp1*/*cct1*, *cct2-5*, *cct6A-B*, *cct7-8*) [8-10]. The CCT complex is mostly known for its role in folding the cytoskeleton proteins actin and tubulin [7,16] and mutations in individual CCT subunits lead to defects in the functioning of the cytoskeleton and mitosis arrest [17].

As for other chaperones, the malfunctioning of chaperonin proteins has been associated with various human pathological conditions, the chaperonopathies [18-20]. In this respect, besides the canonical *cct *and *cpn60 *genes described above, three divergent *hsp60*-like genes have been more recently identified [21-23] in association with pathological conditions. One gene, MKKS [21], was named for its association with the developmental disease McKusick-Kaufman Syndrome and was soon after also identified as BBS6 [24] for its association with the Bardet-Biedl Syndrome (BBS), another developmental condition involving cilium-related dysfunction [25]. More recently two other *hsp60*-like BBS genes, named BBS10 [22] and BBS12 [23], have been identified among fourteen genes (BBS1 to BBS14) so far associated with BBS. The protein products of MKKS/BBS6, BBS10 and BBS12 localize to the basal body of cilia and to the centrosome [26-28]. We will hereafter refer to the MKKS/BBS6 gene as MKKS, and collectively to the three *hsp60*-like BBS genes as the "BBS genes". The identification of these genes provides new perspectives on the spectrum of functionalities of Hsp60-like proteins in eukaryotes and on their role in development.

The recognition of chaperonopathies has increased the importance of elucidating the entire set of chaperone genes present in the human genome [19]. The work reported here was conceived to: a) identify all Hsp60-like sequences encoded in the human and other genomes including all diverged chaperonin genes; b) reconstruct the evolutionary origins and relations of diverged chaperonin genes; c) distinguish with bioinformatics methods functional genes from pseudogenes; d) characterize structural properties of the corresponding proteins. We mostly devoted our attention to the characterization of the evolutionary history and structural properties of newly or recently identified sequences, referring the reader to the vast amount of published literature for information on functional/structural properties and the evolutionary history of mitochondrial Cpn60 or CCT-complex proteins.

Exhaustive searches of *hsp60*-like sequences were carried out in human and other genomes following and extending our "chaperonomics" methodological protocol [29]. The extensive analysis of the genomes of human and other vertebrate species lead to the identification and characterization of many previously unknown sequences and to the discovery of a new, mammal-specific class of chaperonin proteins. Classification, evolutionary analysis and structural characterization of diverged chaperonin-like sequences should provide valuable information for future studies on the functional roles of these proteins.

## Results

### Chaperonin sequences in the human genome

To identify all human *hsp60*-like sequences we queried the human genome using the nine human CCT subunit and mitochondrial Cpn60 sequences. Analogous extensive searches were performed in the mouse and rat genomes using corresponding queries. In the human genome, we found a total of 54 sequences with significant similarity to Hsp60 proteins (Tables 1 and 2). Fifteen sequences had a NCBI Entrez [30] gene descriptor assigned. Nine of these corresponded to the canonical CCT-subunit sequences and one, HSPD1, encoded the mitochondrial Cpn60 protein. Three sequences corresponded to the BBS genes MKKS, BBS10 and BBS12. We recovered two additional uncharacterized sequences designated in the NCBI Entrez Gene database as CCT8L1 and CCT8L2. Besides these complete Hsp60-like sequences, a sequence domain conserved across eukaryote species with highest similarity to the apical domain of the CCT3 protein has also been reported in PIKFYVE [31], a kinase belonging to the Fab1p protein family involved in corneal pathological conditions [32]. In addition, we identified 39 other human *hsp60 *sequences that did not correspond to a gene descriptor in the NCBI Entrez Gene database (Table 2). All of these sequences contained in-frame stop codons or frame-shifts, suggesting that they were most likely pseudogenes. Thirty-five of these had not been described in the Pseudogene.org pseudogene database [33] and 33 were not listed in the Ensembl database [34], and are here annotated and classified for the first time. In analogous searches of the complete genomes of mouse and rat, we identified in each genome 14 chaperonin genes (nine for the canonical CCT monomers, one for the mitochondrial Cpn60, three BBS genes and one CCT8L gene), 38 pseudogenes in mouse and 61 pseudogenes in rat (see additional file 1: Table S1, for mouse sequences, and additional file 2: Table S2, for rat sequences).

**Table 1:** The human *hsp60 *genes

Name^1^	Alternative names	Start^2^	End^3^	Str^4^	Chr^5^	Loc^6^	IF^7^	Exons^8^	aa^9^
**CCT1^10^**	TCP1, CCTa, CCTα TCP-1-α	160,119,520	160,130,731	-	6	q25.3	2	12, 7	556, 401
**CCT2**	CCT β, TCP-1-β	68,266,317	68,280,052	+	12	q15	1	14	535
**CCT3**	CCT γ, TCP-1-γ	154,545,617	154,572,307	-	1	q23.1	3	13, 13, 12	545,544, 507
**CCT4**	CCT δ, TCPD, TCP-1-δ	61,950,076	61,969,146	-	2	p15	1	13	539
**CCT5**	CCT ε, TCP1E, TCP-1-ε	10,303,453	10,317,892	+	5	p15.2	1	11	541
**CCT6A**	CCT ζ, CCT ζ-1, TCP-1-ζ, CCT6, Cctz, HTR3, TCP20, TCPZ, TTCP20	56,087,036	56,098,269	+	7	p11.2	2	14, 13	531,486
**CCT6B**	CCT ζ-2, TCP-1-ζ-2, Cctz2, TSA303, Tcp20	30,279,183	30,312,525	-	17	q12	1	14	530
**CCT7**	CCT η, TCP-1-η, Ccth, NIP7-1	73,320,279	73,333,494	+	2	p13.2	2	12, 7	543,339
**CCT8**	CCT θ, TCP-1-θ, Cctq	29,350,670	29,367,782	-	21	q21.3	1	15	548
**CCT8L1**	LOC155100	151,773,495	151,775,165	+	7	q36.1	1	1	557
**CCT8L2**	GROL, CESK1	15,451,770	15,453,440	-	22	q11.1	1	1	557
**MKKS**	BBS6	10,333,898	10,342,162	-	20	p12.2	2	4, 4	570,570
**BBS10**	C12orf58, FLJ23560	75,263,727	75,266,269	-	12	q21.2	1	2	723
**BBS12**	C4orf24, FLJ35630, FLJ41559	123,882,498	123,884,627	+	4	q27	1	1	710
**HSPD1**	GROEL, HSP60, SPG13, CPN60, HuCHA60	198,060,018	198,071,817	-	2	q33.1	2	11, 11	573,573
*(PIKFYVE)*^11^	CFD, FAB1, PIP5K, PIP5K3	209,182,591	209,190,094	+	2	q34	1	5	224

^1^Official NCBI Entrez gene database name; ^2^Start and ^3^End of coding region; ^4^Strand "+" indicates sequenced strand. "-" indicates complementary strand; ^5^Chromosome; ^6^Chromosome location; ^7^Number of isoforms; ^8^Number of exons. Multiple numbers indicate the number of exons in each isoform; ^9^Total amino acids; ^10^The official Entrez name is TCP1. CCT1 improves consistency with other subunit gene names. ^11^Fab1_TCP sequence domain of PIKFYVE kinase, most similar to the apical domain of CCT3. Features refer to the domain portion of the gene/protein.

**Table 2:** The human *hsp60 *pseudogenes

Name^1^	Start^2^	End^3^	Str^4^	Chr^5^	Loc^6^	Ex^7^	P/D^8^	Ka/Ks^9^	LRT^10^	FS^11^	SC^12^	aa^13^
CCT1-1P	19,986,638	19,987,216	+	12	p12.2	1	P	0.75	0.16	5	2	190
CCT1-2P	41,621,756	41,623,646	-	5	p13.1	2?	D	1.21	0.15	7	5	512
CCT1-3P^14^	42,801,030	42,802,033	+	7	p14.1	3	D	0.68	2.10	1	1	367
CCT3-1P	16,177,578	16,178,178	+	8	p22	1	P	1.02	0.0	2	2	159
CCT4-1P	64,177,578	64,409,590	+	X	q12	3	D	0.65	1.76	0	3	512
CCT4-2P	140,344,301	140,345,787	-	7	q34	4	D	0.82	1.24	2	10	278
CCT5-1P^14,15^	78,382,086	78,382,680	+	13	q31.1	1	P	0.81	0.20	3	4	549
CCT5-2P^15^	78,382,866	78,382,967	-	13	q31.1	1	?	-	-	-	1	34
CCT5-3P	114,876,388	114,877,290	+	5	q22.3	1	P	0.25	2.12	6	3	201
CCT6-1P	14,692,965	14,693,954	-	5	p15.2	1	P	1.00	0.0	2	2	330
CCT6-2P	109,013,584	109,014,117	-	11	q22.3	1	P	0.90	0.0	0	2	178
CCT6-3P^16^	64,162,812	64,171,325	+	7	q11.21	8	D	0.43	3.06	5	4	289
CCT6-4P	191,915,332	191,916,879	+	3	q28	1	P	0.57	6.90**	9	4	292
CCT6-5P^14,16^	64,853,564	64,865,440	+	7	q11.21	10	D	0.84	0.34	12	6	399
CCT7-1P^14^	92,251,627	92,307,366	-	5	q15	1	P	0.45	1.88	3	5	145
CCT7-2P	150,242,815	150,243,240	+	6	q25.1	1	P	0.87	0.10	3	4	552
CCT8-1P^14^	145,141,482	145,143,137	-	1	q21.1	1	P	1.14	0.10	2	3	561
HSPD1-1P^14^	135,744,902	135,745,039	-	5	q31.1	1	P	1.46	0.27	0	0	48
HSPD1-2P^14,17^	21,919,402	21,920,175	-	5	p14.3	1	P	0.90	0.10	1	1	264
HSPD1-3P	43,602,029	43,602,280	-	20	q13.12	4	D	-	-	0	1	84
HSPD1-4P	88,065,673	88,066,269	+	6	q15	1	P	0.55	1.08	4	5	199
HSPD1-5P^14^	55,191,053	55,192,769	+	12	q13.2	1	P	0.56	3.08	2	1	499
HSPD1-6P^14^	36,783,612	36,785,195	-	3	P22.3	1	P	0.59	2.46	2	2	443
HSPD1-7P^18^	7,263,938	7,265,475	+	8	p23.1	1	P	1.12	0.18	5	4	396
HSPD1-8P	145,986,418	145,987,946	+	4	q31.21	1	P	0.63	2.32	4	3	458
HSPD1-9P^18^	7,785,932	7,787,502	-	8	p23.1	1	P	0.91	0.08	5	3	416
HSPD1-10P	8,058,884	8,082,857	+	12	p13.31	2?	D	0.78	0.94	1	1	307
HSPD1-11P	95,130,459	95,132,169	+	5	q15	5	D	0.74	2.44	6	6	375
HSPD1-12P	78,321,372	78,323,341	+	13	q31.1	1	P	0.62	4.98*	5	4	410
HSPD1-13P	153,068,626	153,068,943	+	6	q25.2	1	P	0.54	2.84	1	2	108
HSPD1-14P^14^	37,465,288	37,466,827	-	13	q13.3	4	D	0.68	3.3	6	3	361
HSPD1-15P	19,269,394	19,270,353	+	5	p14.3	4	D	0.74	1.24	4	4	241
HSPD1-16P	105,082,802	105,083,755	+	11	q22.3	2?	D	0.51	6.24*	5	4	199
HSPD1-17P	34,077,070	34,078,293	+	1	p35.1	3	D	0.74	1.48	2	2	217
HSPD1-18P	56,105,684	56,108,736	+	20	q13.32	5	D	0.48	10.84**	3	2	299
HSPD1-19P	50,318,868	50,319,008	+	10	q11.23	1	?	2.42	0.72	0	0	47
HSPD1-20P	78,924,341	78,924,478	-	12	q21.31	1	?	0.40	3.08	0	0	46
HSPD1-21P	60,994,430	60,994,876	-	5	q12.1	6	D	-	-	0	6	155
HSPD1-22P	29,181,851	29,183,334	-	21	q21.3	2?	D	0.69	1.4	3	4	344

^1^Pseudogene names follow the HUGO nomenclature. They are composed of the name of the parental gene followed by a unique number identifier and the suffix "P" (Pseudogene); ^2^Start and ^3 ^End positions of the pseudogene on the chromosome; ^4^Strand; ^5^Chromosome; ^6^Location on the chromosome; ^7^Number of exons. A question mark indicates gene fragments with uncertain numbers of exons; ^8^Processed (P), duplicated (D) or undetermined (?); ^9^Ratio of non-synonymous vs. synonymous substitution rates; ^10^Likelihood Ratio Test (LRT) values. Values different from 1.0 with probability *p *< 0.01 (**) or *p *< 0.05 (*) are shown in bold-face; ^11^Number of Frame-Shifts recognized in the coding region of the pseudogene; ^12^Number of in-frame Stop Codons recognized in the coding region of the pseudogene; ^13^Length in amino acids of pseudo-translation of the recognized pseudogene sequence; ^14^Ten pseudogenes previously reported in the Ensembl (roman), Pseudogene.org (italics) or NCBI (bold) databases: CCT1-3P = OTTHUMG00000033751; CCT5-1P = *Human.chr13.mb78*; CCT6-5P = ENSP00000275603, *Human.chr7.mb64*; CCT7-1P = ENST00000399032; CCT8-1P = *Human.chr1.mb145*; HSPD1-1P = ENSG00000162241, *Human.chr5.mb135*; HSPD1-2P = ENSP00000328369; HSPD1-5P = **LOC644745**; HSPD1-6P = **LOC645548**; HSPD1-14P = OTTHUMG00000016753; ^15,16,18^Tandemly duplicated;^17^Previously identified as Hsp60s2 (Hsp60 short form 2).

### Evolutionary origins of human BBS and CCT8L genes

**Figure 1:**
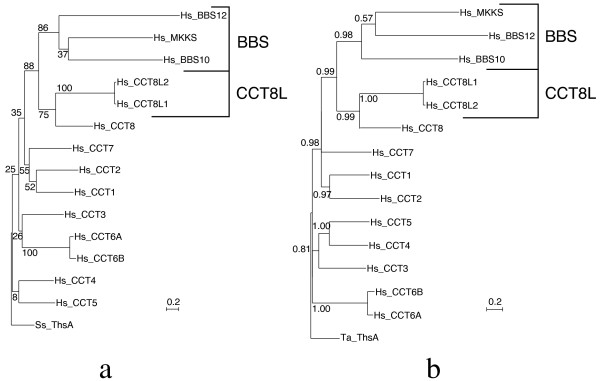
**Evolutionary trees of CCT proteins**. (a) Maximum-likelihood evolutionary tree of all human chaperonin-like proteins, including CCT monomers, MKKS, BBS10, BBS12 and the two members, CCT8L1 and CCT8L2, of the newly defined CCT8L class. Numbers associated with each branch indicate bootstrap support from 100 replicates. Tree rooted by the archaeal thermosome alpha subunit of *Sulfolobus solfataricus *(Ss_ThsA). (b) Bayesian evolutionary tree of the same sequences shown in (a). The numbers assigned to each branch indicate posterior probabilities. Tree rooted by the thermosome alpha subunit of *Thermoplasma acidophilum *(Ta_ThsA). The scale bars represent the indicated number of substitutions per position for a unit branch length.

A maximum-likelihood (ML) phylogenetic tree of human chaperonin-like proteins (Figure 1a) indicated that Hsp60-like BBS proteins are monophyletic (bootstrap support 86%) and that their common ancestor derived from a duplication event in the CCT8 lineage (bootstrap support 88%). The tree also showed that the unique ancestor of the two closely related genes CCT8L1 and CCT8L2 also originated in the CCT8 lineage from a more recent duplication event (bootstrap support 75%). The relation of BBS and CCT8L proteins with the CCT8 chaperonin subunit was confirmed with strong conditional probability support (0.99) by Bayesian tree construction (Figure 1b).

Although the association of BBS and CCT8L proteins with the CCT lineage was robustly supported, the high divergence of these sequences could produce clustering in the trees due to long-branch attraction. To address this concern, we built independent ML trees for each BBS or CCT8L sequence adding them separately to the tree of CCT subunits. All individual trees confirmed with strong bootstrap support the association of each BBS or CCT8L lineage with the CCT8 lineage (see additional file 3: Figure S1, additional file 4: Figure S2, additional file 5: Figure S3 and additional file 6: Figure S4). A ML evolutionary tree including *hsp60*-gene homologs found in the genomes of eighteen other vertebrate species, including representatives of several mammals, chicken, frogs, and fish, also confirmed the origin of BBS and CCT8L genes from the CCT8 lineage (see additional file 7: Figure S5).

**Figure 2:**
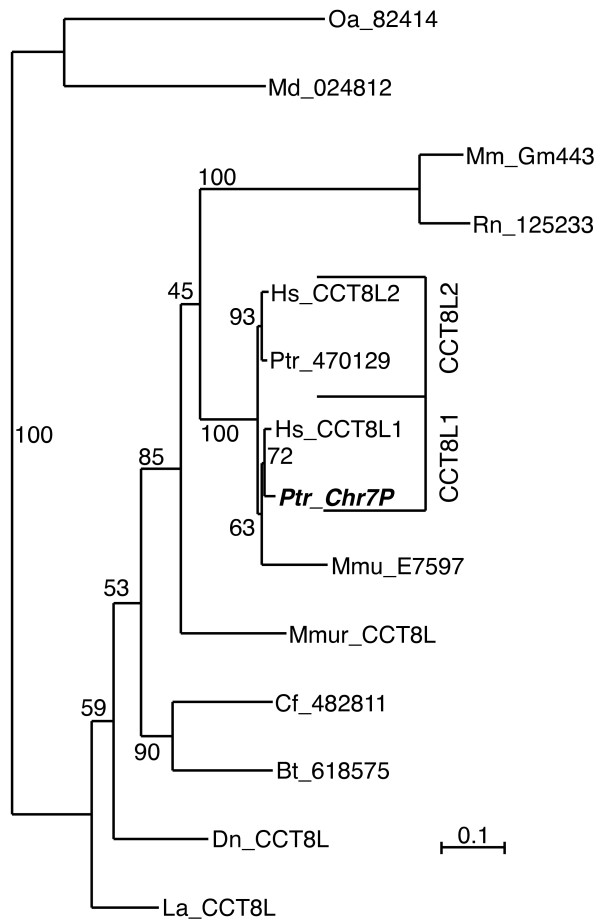
**Evolutionary tree of CCT8L sequences**. ML tree of CCT8L sequences from various mammal genomes. The homolog of human CCT8L1 in chimp (Ptr) is characterized as pseudogene and is shown in bold-italics font. Species abbreviations: Bt, *Bos taurus *(cow); Cf, *Canis lupus familiaris *(dog); Dn, *Dasypus novemcinctus *(nine-banded armadillo); Dr, *Danio rerio *(zebrafish); Ec, *Equus caballus *(horse); Ga, *Gasterosteus aculeatus *(stickleback, fish); Gg, *Gallus gallus domesticus *(chicken); Hs, *Homo sapiens *(human); La, *Loxodonta africana *(african bush elephant); Md, *Monodelphis domestica *(south american gray short-tailed opossum, marsupial); Mm, *Mus musculus *(mouse); Mmu, *Macaca mulatta *(rhesus monkey); Mmur, *Microcebus murinus *(gray mouse lemur); Oa, *Ornithorhynchus anatinus *(platypus); Ol, *Oryzias latipes *(the medaka or japanese killifish); Pp, *Pongo pygmaeus *(northwest bornean orangutan); Ptr, *Pan troglodytes *(chimpanzee); Rn, *Rattus norvegicus *(rat); Tn, *Tetraodon nigroviridis *(spotted green pufferfish); Tr, *Takifugu rubripes *(japanese pufferfish); Xl, *Xenopus laevis *(african clawed frog, amphibian); Xt, *Xenopus tropicalis *(western clawed frog, amphibian). The scale bar represents the indicated number of substitutions per position for a unit branch length.

We did not find CCT8L genes in the genomes of chicken, *Xenopus laevis*, or *Danio rerio*, representatives respectively of the reptile/bird, amphibian and fish lineages. However, among mammals we identified orthologs of CCT8L genes in genomes not only of placental mammals (Eutheria), but also of the marsupial opossum (Metatheria) and of the egg-laying platypus (Prototheria), suggesting that the CCT8L gene class originated at the onset of mammal evolution. All CCT8L gene orthologs were intron-less, indicating that their ancestor originated from a retro-transposition event. Two copies of CCT8L sequences were found in human and chimp and one CCT8L gene in all other genomes examined, including those from the other primate rhesus monkey (*Macaca mulatta*) and gray mouse lemur (*Microcebus murinus*) (Figure 2), suggesting that a duplication of the CCT8L gene occurred in Hominoidea after their separation from old world monkeys. However, the lone gene copy of CCT8L identified in rhesus monkey clustered with CCT8L1 in evolutionary trees (Figure 2), suggesting an earlier duplication of the gene and successive loss of the CCT8L2 copy from the genome of rhesus monkey. Close inspection of protein alignments revealed that the rhesus monkey CCT8L sequence included an anomalously diverged segment of about 50 amino acids of uncertain alignment. Excluding this segment from the analysis we obtained a different and more robustly supported tree topology (75% vs. 20% bootstrap value, see additional file 8: Figure S6, panels a and b), consistent with a later duplication of the CCT8L gene in Hominoidea. The tree also indicated that the removed segment was alone responsible for the overall higher evolutionary rate predicted for this sequence (see additional file 8: Figure S6).

### Differentiation rate of BBS and CCT8L proteins

The branch lengths of the trees shown in Figure 1 indicate that BBS and CCT8L proteins have differentiated at much higher rates than CCT subunits. We applied a newly-developed, unbiased measure of differentiation called "B-index" (see Methods) to calculate differentiation of MKKS, BBS10 and BBS12 proteins from their respective last ancestor common to Actinopterygii (ray-finned fishes) and Sarcopterygii (including tetrapods), determined by rooting the trees with CCT8 proteins from corresponding fish and tetrapod species. Similarly, we calculated differentiation of CCT8L proteins from a eutherial ancestor rooting their tree with corresponding sets of CCT8 proteins (see footnotes of Table 3 and legend for Figure 2 for species represented in each tree). We estimated for the MKKS family an average evolutionary distance from their root of almost 0.7 substitutions per site, corresponding to a 6-fold higher rate of differentiation compared to the number of substitutions estimated in CCT8 proteins over the same period of time. For BBS10 and BBS12, we calculated a distance of about 1.0-1.2 substitutions per site, corresponding to a substitution rate about 8-10 times higher than in CCT8. Finally, for the mammal-specific family of CCT8L proteins, we estimated an evolutionary distance from their mammal root of about 0.3 substitutions per site. The smaller divergence of CCT8L proteins compared to BBS proteins reflects the more recent origin of the CCT8L gene. However, when scaled to the evolution of CCT8 sequences over the same periods of time, the substitution rate of CCT8L proteins was about 14-15 times higher than in CCT8 and 1.4-2.3 times higher than in BBS proteins.

**Table 3:** Divergence of BBS and CCT8L proteins relative to CCT8 proteins

	MKKS	BBS10	BBS12	CCT8L^1^
**No. species^2^**	**14**	**11**	**11**	**5**

Size (*W*_*B*_)^3^	5.7770	4.6020	5.8949	3.3859
B-index (*B*_*B*_)^4^	0.6976	1.1079	1.0284	0.3196
Unbiased pair-wise distance (*B*_*B *_× 2)	1.3952	2.2159	2.0568	0.6393
*L*_*B *_(*B*_*B *_× *W*_*B*_)^5^	4.0300	5.0987	6.0623	1.0822
Average *D*_*ij *_(*D*_*B*_)^6^	2.0951	2.9660	3.2858	0.8017
				
**CCT8^7^**				
Size (*W*_*C*_)	5.5202	4.5503	4.3647	3.1100
B-index (*B*_*C*_)	0.1146	0.1373	0.0992	0.0227
Unbiased pair-wise distance (*B*_*C *_× 2)	0.2291	0.2747	0.1983	0.0454
*L*_*C *_(*B*_*C *_× *W*_*C*_)	0.6324	0.6250	0.4328	0.0706
Average *D*_*ij *_(*D*_*C*_)	0.3394	0.3687	0.2709	0.0545
				
*B*_*B*_/*B*_*C*_	6.0873	8.0692	10.3669	14.0793
*L*_*B*_/*L*_*C*_	6.3725	8.1579	14.0072	15.3286
*D*_*B*_/*D*_*C*_	6.1730	8.0445	12.1292	14.7101
*W*_*B*_/*W*_*C*_	1.0465	1.0114	1.3506	1.0887

^1^Only the human CCT8L2 branch was included in the tree. The CCT8L1 branch had equivalent length; ^2^Chaperonin-BBS sequences used in the trees were from the following species (see the legend for Figure 2 for a complete list of abbreviations and species names). MKKS: Bt, Cf, Dr, Ec, Ga, Gg, Hs, Md, Mm, Mmu, Ol, Rn, Tr, Xt; BBS10: Bt, Cf, Dr, Ec, Ga, Hs, Md, Mm, Ol, Rn, Tr; BBS12: Bt, Cf, Dr, Ec, Ga, Gg, Hs, Mm, Ol, Rn, Tr, Xl; CCT8L: Bt, Cf, Hs, Mm, Rn. ^3^Size is the average number of sequences contained in a cluster over evolutionary time (see Methods); ^4^The B-index measures the average substitutions per site (evolutionary distance) of the sequences within a cluster from their common ancestor; ^5^*L *is the length of the tree (sum of the lengths of all branches); ^6^Average *D*_*ij *_is the average pair-wise evolutionary distance of the sequences; ^7^Estimates for CCT8 were computed over corresponding species represented by the sets of MKKS, BBS10, BBS12 or CCT8L proteins (see footnote 2, above).

### Functional constraints in the evolution of CCT8L genes

**Figure 3:**
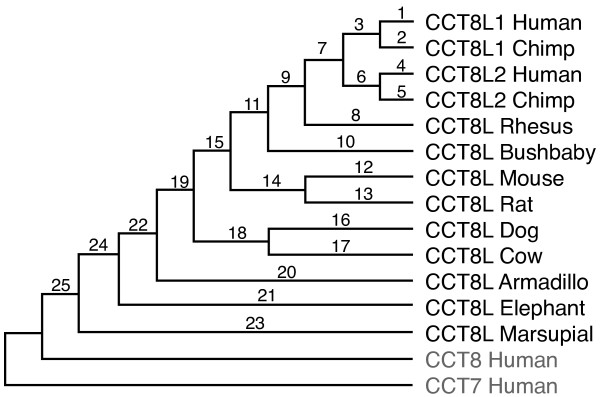
**Evolutionary relations of CCT8L genes**. Schematic representation of evolutionary relations of CCT8L genes from different eukaryotic species rooted by CCT8 and CCT7 sequences. The numbers associated with each branch identify the branches for which branch-specific Ka/Ks values are evaluated (Table 4).

We tested functionality of CCT8L genes from several species estimating ratios of non-synonymous and synonymous substitution rates (Ka/Ks) along their respective lineages (see Methods). The results of this analysis are shown in Table 4, which indicates the gene(s) analyzed (foreground), the two genes used to identify foreground and background branches, the estimated Ka/Ks values and their significance. The evolutionary lineages for which Ka/Ks values were evaluated correspond to the branch numbers identified in the overall tree topology shown in Figure 3. In this tree are represented the "molecular tree" of mammal phylogenetic relations [35], the gene duplication event involving the CCT8L gene family in primates as inferred by this analysis, and the pre-mammal separations of the CCT7, CCT8 and CCT8L families of paralogs. This topology is in agreement with the evolutionary tree of CCT8L genes (Figure 2) with the only exception of the weakly supported position of the CCT8L sequence from rhesus monkey (see above). The highly significant constraints in non-synonymous substitution rates (Ka/Ks < 1.0) estimated in the overall evolution of the CCT8L family (Table 4, foreground genes: "All CCT8L1/2") indicated that the CCT8L sequences are genes generally expressing functional proteins. In evaluating Ka/Ks ratios for individual CCT8L gene lineages (Table 4), significantly constrained evolution (Ka/Ks < 1.0) was detected for branches leading to most sequences, including those of murids, lemur, cow, dog, elephant, marsupial, and to the human CCT8L1 and CCT8L2 group along the hominoid lineage. Constrained evolution was also estimated for the CCT8L genes of armadillo and rhesus monkey, and for human CCT8L1 and human and chimp CCT8L2 after divergence of human and chimp, although in these cases Ka/Ks values did not reach significance. In the cases of the human and chimp CCT8L1 and CCT8L2 genes, the lack of significance can be related to the loss of power of the test since few mutations accumulated after separation of these sequences (see additional file 9: Table S3). In the case of rhesus monkey CCT8L, we found that its relatively high estimate of Ka/Ks (= 0.73) was due to the previously mentioned 50-amino-acid diverged region within this sequence. After removing this region we estimated Ka/Ks = 0.55. Only for the lineage of chimp CCT8L1 we estimated Ka/Ks ≅ 1, consistent with differentiation of a non-functional sequence. Since this sequence was also characterized by an internal stop codon and a frame-shift, all evidence strongly suggests that chimp CCT8L1 is a pseudogene.

**Table 4:** Ka/Ks substitution ratios in CCT8L genes evolution

Foreground genes^1^	Background genes^1^	Foreground Ka/Ks^2^	LRT (*p*)^3^	Foreground branches^4^
All CCT8L1/2	Human CCT8, Human CCT7	0.29	205.06 (<0.001)	1 to 25
Human CCT8L1	Chimp CCT8L1, Human CCT8L2	0.58	0.88	1
Chimp CCT8L1	Human CCT8L1, Human CCT8L2	1.02	0.00	2
Human CCT8L2	Chimp CCT8L2, Human CCT8L1	0.48	1.2	4
Chimp CCT8L2	Human CCT8L1, Human CCT8L2	0.39	1.8	5
Human CCT8L2	Human CCT8L1, Rhesus CCT8L	0.42	4.02 (<0.05)	4+6
Human CCT8L1	Human CCT8L2, Rhesus CCT8L	0.29	5.72 (<0.05)	1+3
Mouse and Rat CCT8L	Cow CCT8L, Human CCT8L2	0.38	31.14 (<0.001)	12+13+14
Mouse CCT8L	Rat CCT8L, Human CCT8L2	0.64	1.21	12
Rat CCT8L	Mouse CCT8L, Human CCT8L2	0.49	5.91 (<0.05)	13
Rhesus CCT8L	Lemur CCT8L, Human CCT8L2	0.73 (0.55)^5^	1.91 (1.22)^5^	8
Lemur CCT8L	Human CCT8L2, Mouse CCT8L	0.29	36.82 (<0.001)	10
Dog CCT8L	Cow CCT8L, Human CCT8L2	0.31	12.07 (<0.001)	16
Cow CCT8L	Dog CCT8L, Human CCT8L2	0.13	113.78 (<0.001)	17
Armadillo CCT8L	Elephant CCT8L, Human CCT8L2	0.36	0.57	20
Elephant CCT8L	Marsupial CCT8L, Human CCT8L2	0.29	14.96 (<0.001)	21
Marsupial CCT8L	Elephant CCT8L, Human CCT8L2	0.31	62.63 (<0.001)	23+24

^1^See text for the definition and meaning of Foreground and Background species; ^2^Ka/Ks is the estimated ratio of non-synonymous and synonymous substitution rates; ^3^LRT, Likelihood Ratio Test results for estimated Ka/Ks vs. Ka/Ks = 1.0 (see Methods). Probabilities (*p*) not shown signify *p *> 0.05; ^4^Foreground-branch numbers correspond to the numbering in the schematic tree shown in Figure 3. ^5^Values in parenthesis were obtained after removing an unusually diverged region from rhesus CCT8L (see text).

To assess the functionality of human CCT8L sequences we investigated their expression profiles in comparison to those of human CCT monomers and BBS genes (see additional file 10: Table S4). Expression of CCT8L2 was confirmed by fifteen ESTs mostly identified from the testis, whereas only one EST identified as a CCT8L1 transcript has been so far reported (NCBI UniGene database, November 20, 2009). Querying the NCBI GEO microarray database, we found 542 expression-profile records identifying expression of CCT8L2, and none identifying expression of CCT8L1 (as of November 20, 2009). It must be noted, however, that CCT8L2 and CCT8L1 have similarity of 97.3% at the DNA level. Similarly to CCT8L2, another mammal-specific chaperonin gene, CCT6B, is also expressed almost exclusively in the testis, from which 160 ESTs have been reported versus an average of 4.4 ESTs (from 0 to 10 per tissue) found in all other tissues.

### Pseudogenes

We identified in the human genome 39 sequences with significant similarity to CCT or HSPD1 genes that either were short fragments or were characterized by in-frame stop codons or frame-shifts. Based on their corruption, we classified these sequences as pseudogenes (Table 2). Similarly, searching the mouse and rat genomes we identified 38 and 61 pseudogenes, respectively (see additional file 1: Table S1 and additional file 2: Table S2). Most of these sequences have not been previously reported and are here systematically annotated and classified for the first time.

Based on phylogenetic-tree reconstructions (see additional file 11: Figure S7) or on similarity for the most corrupted sequences, we identified the association of 17 pseudogenes from human, 16 from mouse and 29 from rat with one of the nine CCT genes. None of the pseudogenes were related to MKKS, BBS10, BBS12 or CCT8L. To estimate the time of origin of the pseudogenes, we constructed trees using their translated sequences and chaperonin subunits from various vertebrate species (see additional file 12: Figures S8, and additional file 13: Figure S9). The trees indicated that all recognizable human CCT pseudogenes originated in the mammal lineage after separation from the reptile/bird lineage.

**Figure 4:**
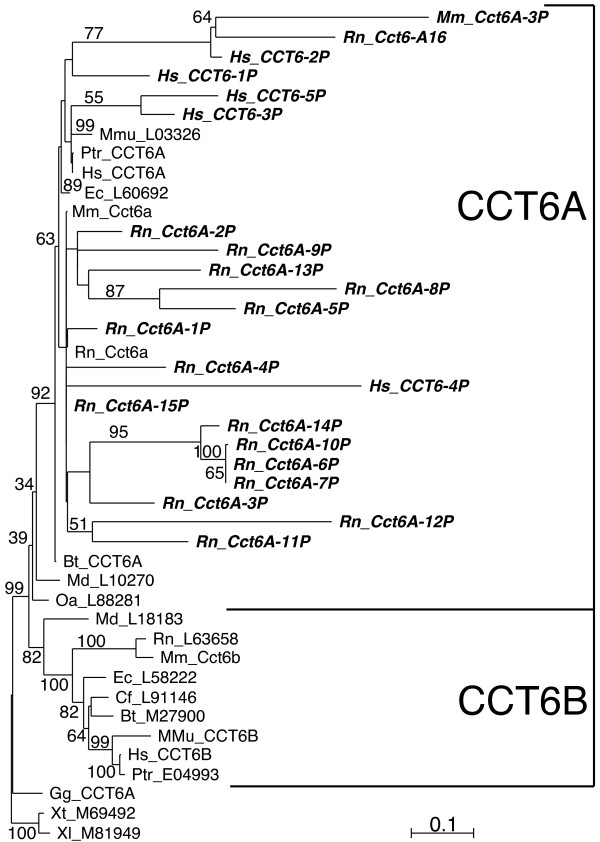
**Evolutionary tree of vertebrate CCT6 proteins**. ML tree of CCT6 proteins from mammals, chicken, and frog (in roman font) and translated sequences of the related pseudogenes from human, mouse, and rat (in bold-italics font). Only one copy of CCT6 was found in chicken and frog. Two copies, CCT6A and CCT6B, were found in all mammals examined, including marsupial (Md) and platypus (Oa). The CCT6 sequences from chicken (Gg) and from the two amphibians *Xenopus laevis *(Xl) and *Xenopus tropicalis *(Xt) were used to root the tree. All human, mouse, and rat pseudogenes clustered with the CCT6A sequences. Numbers next to branches indicate percent bootstrap values. Only bootstrap values > 30% are shown. For all species abbreviations see the legend for Figure 2. The scale bar represents the indicated number of substitutions per position for a unit branch length.

Of particular interest were the evolutionary relations of CCT6 genes and pseudogenes. Two CCT6 gene copies (CCT6A and CCT6B) were found, besides placental mammals, also in platypus and in opossum (see additional file 11: Figure S7), suggesting that the duplication of the CCT6 gene occurred in mammal evolution before separation of Theria (marsupial and placental mammals) and Prototheria (monotremes). We constructed an evolutionary tree of mammal CCT6 genes and pseudogenes (Figure 4) rooted by the corresponding gene sequences from chicken and frog (the diverged sequence Oa_con2651 from platypus was excluded from this tree to avoid long-branch attraction). Surprisingly, all recognizable human, mouse, and rat pseudogenes belonging to the CCT6 class branched in the tree from the CCT6A lineage after separation of the platypus, marsupial and placental mammal lineages.

**Figure 5:**
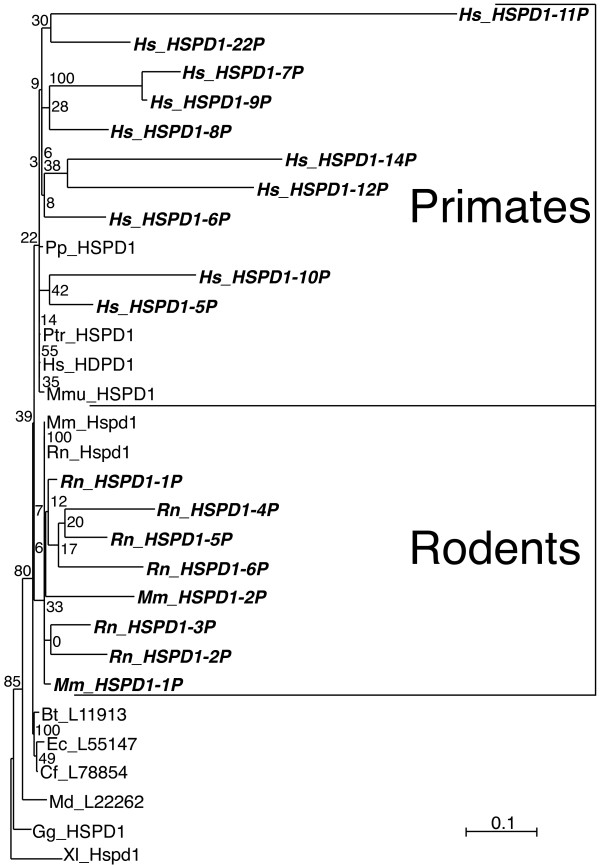
**Evolutionary tree of vertebrate mitochondrial Cpn60**. ML tree of mitochondrial Cpn60 proteins from mammals, chicken, and frog (in roman font) and translated sequences of the related pseudogenes from human, mouse, and rat (in bold-italics font). Highly degraded pseudogenes for which only fragments could be detected were not considered. Human pseudogenes clustered with primate Cpn60 sequences whereas mouse and rat pseudogenes clustered with rodent counterparts, indicating independent evolution of these pseudogenes in these species. For all species abbreviations see legend for Figure 2. The scale bar represents the indicated number of substitutions per position for a unit branch length.

Twenty-two pseudogenes in human (Table 2), and 22 and 32 pseudogenes in mouse and rat, respectively (see additional file 1: Table S1 and additional file 2: Table S2), associated with the mitochondrial HSPD1 gene (Group I *cpn60*). Evolutionary trees incorporating all pseudogenes from different vertebrate species were uninformative due to the presence among the pseudogenes of highly corrupted sequences, resulting in extensive long-branch attraction (not shown). An ML tree built using only translations of the most conserved pseudogenes (Figure 5) showed weakly supported but consistent association of the human pseudogenes with HSPD1 from primates, whereas pseudogenes from mouse and rat all associated with murid Hspd1 sequences, also indicating their relatively recent origin.

### Ka/Ks ratio in the evolution of putative pseudogene sequences

Our characterization of many *hsp60 *sequences as pseudogenes was based on the presence of signs of corruption in the sequence (in-frame stop codons and frame-shifts). However, in-frame stop codons and frame-shifts may correspond to truncated proteins that are still functional. For example, although human HSPD1-5P and HSPD1-6P sequences contain signs of sequence corruption, EST data indicate that these sequences are expressed and possibly functional (see additional file 14: Table S5). To confirm our characterization, we estimated Ka/Ks ratios in trees that identified the pseudogene-sequence lineage (branch) including as out-group its parental gene and the orthologous gene sequence from chicken (see Methods). The results of these analyses (Table 2) showed in most cases Ka/Ks values not significantly different from 1.0, as expected in the differentiation of pseudogene sequences not constrained by coding of functional amino acids. Significant differences in mutation rate were estimated in the case of four sequences. These sequences, however, contained multiple in-frame stop codons and frame-shifts (Table 2).

### Structural features of BBS and CCT8L proteins

**Figure 6:**
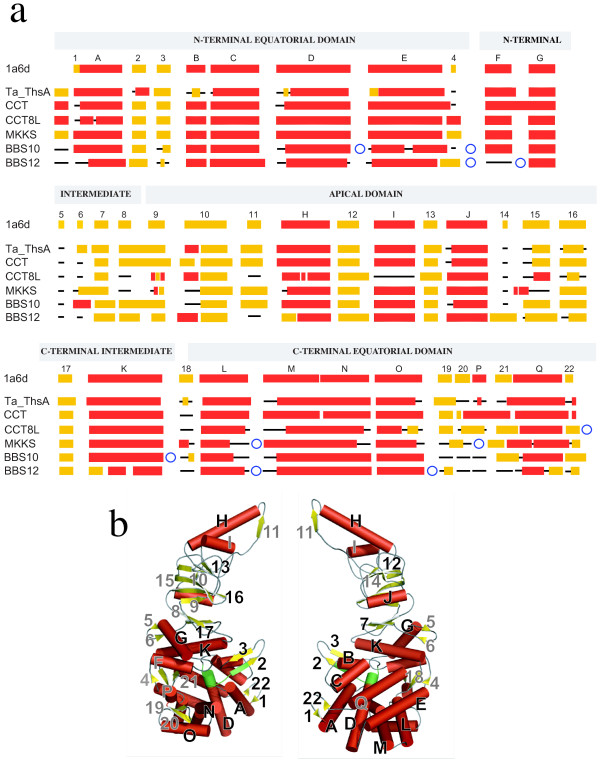
**Secondary structure predictions of chaperonin proteins**. (a) Secondary structure predictions of *Thermoplasma acidophilum *thermosome alpha subunit ThsA (line Ta_ThsA), human CCTs, mammal CCT8Ls and vertebrate BBSs (lines MKKS, BBS10 and BBS12) compared to the secondary structure description of ThsA (top line 1a6d) determined from its crystal structure (PDB code 1a6d, chain A). Helices are represented as red boxes, beta-strands as yellow boxes and loops as black lines. Secondary structure elements in 1a6d are labeled in succession with numbers (strands) or letters (helices). The first 16 N-terminal residues of ThsA, predicted to contain a strand, are not included in the 1a6d crystal structure (top line). Secondary structure elements in all proteins recognized as homologous to the thermosome chain elements by sequence similarity and positional equivalence are vertically aligned. Blue circles indicate the position of sequence insertions in CCT8L and BBS sequences. (b) The three-dimensional fold of the secondary structure elements in the thermosome structure 1a6d chain A. Red cylinders represent helices and yellow arrows represent strands. Labels (i.e., letters and numbers) correspond to those in panel "a". Elements not predicted in some of the BBS and CCT8L sequences are labeled in gray. The positions of the ATP binding and hydrolysis sites are highlighted in green.

Because of their high sequence divergence, it is unclear whether BBS and CCT8L Hsp60-like proteins conserve the typical fold of chaperonin subunits and their ability to assemble into typical oligomeric chaperonin complexes. Chaperonin monomers are characterized by three structural domains (apical, intermediate and equatorial) with distinct functional roles and it was relevant to investigate whether BBS and CCT8L proteins conserve each of the domains typical of chaperonins. Experimental models of eukaryotic Group II chaperonins are not available but their structural properties can be inferred by comparison with their closest relative, the archaeal thermosome. To infer tertiary-structure conservation in BBS and CCT8L proteins we predicted the secondary structure for each family from alignments of multiple sequences, excluding structure and sequence information from other families. The results of these predictions are schematically represented in Figure 6a, in relation to the secondary structure description of the PDB structure 1a6d chain A of the thermosome subunit ThsA from *Thermoplasma acidophilum *[36] (see additional file 15: Figure S10, additional file 16: Figure S11, additional file 17: Figure S12, additional file 18: Figure S13, additional file 19: Figure S14, and additional file 20: Figure S15 for detailed representations of multiple alignments, secondary structure predictions and alignments to the secondary-structure elements of ThsA). In Figure 6a, the secondary structure description of ThsA is shown (line "1a6d") in relation to the position of the equatorial, intermediate, and apical domains. The position of these elements in the tertiary structure of ThsA is represented in Figure 6b. Results of a blind test of the performance of the method on the corresponding ThsA sequence are also shown (Figure 6a, line "Ta_ThsA"). In this test most strand and helix elements (all "core" helices) described in the crystal structure were correctly predicted by the method, increasing our confidence in the reliability of other predictions. As expected, extensive conservation of predicted secondary-structure elements were also obtained from the alignment of human CCT sequences (Figure 6a, line "CCT") with only few discrepancies involving mostly short beta strands (4, 5, 18, and 21) and one short helix (P) exposed at the external surface of the archaeal thermosome complex. Secondary-structure predictions for mammal CCT8L and for vertebrate MKKS, BBS10 or BBS12 sequences were also largely consistent with the secondary-structure description of thermosome proteins. In the equatorial domain, CCT8L and BBS structure predictions corresponded to the mostly alpha-helical composition of this region. Variations were more obvious in BBS12 and involved mostly terminal elements of helices (most notably helices P and Q) and exposed beta-strands (strands 19-21). In the intermediate domain the core helical-bundle elements (helices F, G, and K) as well as the extensive beta-sheet composition of this region were predicted in all BBS and CCT8L proteins. Exceptions were, in all sequences, the two short strands 5 and 6, which are part of an external elongated loop in the thermosome structure, and, in BBS12, the N-terminal part of helix K, which in the thermosome protrudes towards the central cavity covering the ATP hydrolysis site (Figure 6b). The apical domain is formed in the thermosome by a 4-strand anti-parallel beta-sheet (strands 9, 10, 15, and 16) with strand 10 extending into a second parallel beta-sheet (strands 10, 12, 13, and 14). The two sheets are flanked by a helix (J) and are surmounted by a structure composed of two contacting helices (H and I) and an extended loop including strand 11. All helices and most strands of the apical domain were recognized in BBS sequences. Most obvious differences were observed in BBS12 proteins, where the long apical helix H was predicted to be shortened, and in CCT8L, where helix I and strand 11 were not predicted.

### Differentiation of monomer-monomer interaction regions in BBS and CCT8L proteins

To investigate the potential of CCT8L and BBS proteins to establish intra-ring and inter-ring monomer-monomer contacts, we investigated the relative conservation of predicted contact positions in CCT, BBS and CCT8L sequences. We identified potential contact positions in these families based on homology to the positions involved in inter-monomer contacts in the crystal structure of the *T. acidophilum *thermosome complex (PDB code 1a6d). After identifying all contact positions in CCT monomers, we distinguished among them those that conserved similar amino acid types across the nine monomers. We counted how many amino acid types observed in all or in conserved contact positions of CCT monomers were also observed in the *T. acidophilum *Thsa sequence, in human CCT8Ls or in human BBS sequences (Table 5). A complete list of all and conserved positions considered and of the residue types observed in these positions in all sequences can be found in additional file 21: Table S6. Thsa and CCT subunits conserve 89% similarity in monomer-monomer contact positions, which is substantially higher than the average similarity (62%-66%) of all homologous positions between the two families. The higher similarity of monomer-monomer contact regions is consistent with functional conservation between the two families of these positions. In contrast, the high rate of differentiation in comparison to global average differentiation shown in putative monomer-monomer contact positions in BBS or CCT8L sequences (Table 5), suggests a loss of capability to associate into a typical CCT-like oligomeric complex. This result is consistent with the presence in BBS proteins of inserted elements (Figure 6) that would interfere with formation of the complex [22,23].

**Table 5:** Conservation of monomer-monomer contact residues relative to CCT subunits^1^

Protein	MM	CMM	RR	Global^2^
ThsA	78 (83.9)	16 (94.1)	13 (86.7)	62.0-66.4
BBS12	37 (39.8)	7 (41.2)	6 (40.0)	35.5-38.0
BBS10	45 (48.4)	8 (47.1)	7 (46.7)	34.3-35.6
MKKS	42 (45.2)	8 (47.1)	7 (46.7)	48.8-51.6
CCT8L	54 (58.1)	8 (47.1)	7 (46.7)	53.4-61.1

^1^Conservation of archaeal ThsA and human BBS and CCT8L sequences relative to human CCT monomers. Sequence-positions are considered conserved if they are occupied by residue-types appearing in the homologous position in any of the human CCT sequences. Ninety-three intra-ring contact positions and 15 inter-ring contact positions were identified from the thermosome structure (1ad6). Contact positions were defined by a distance of their side-chain heavy atoms of at most 4.0Å from any heavy atom of the nearby monomer in the thermosome structure. For each protein family, the table indicates the number and percentage (in parenthesis) of positions conserved among: all 93 intra-ring contact positions (**MM**); seventeen intra-ring contact-positions conserved among human CCT monomers (**CMM**); all 15 inter-ring contact positions, none of which were conserved among CCT monomers (**RR**). ^2^**Global **indicates the range of similarities (percent values) of each sequence to human CCT-subunit proteins within all aligned positions.

### Conservation of ATP-binding and hydrolysis residues in BBS and CCT8L proteins

**Figure 7:**
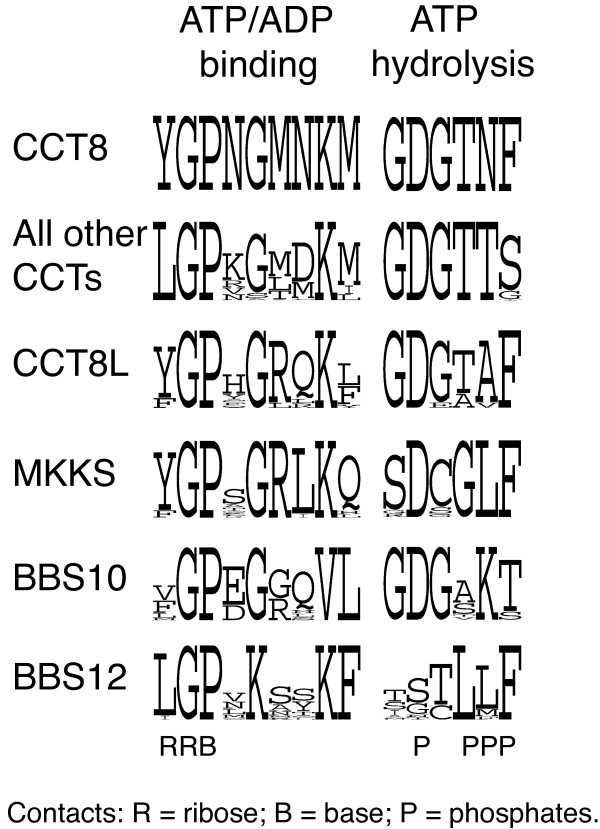
**Profile logos of ATP-binding and ATP-hydrolysis sites in chaperonin proteins**. Sequence profiles of ATP/ADP-binding and ATP-hydrolysis sites for CCTs, CCT8L and BBS (MMKS, BBS10 and BBS12) proteins from the multiple sequence alignments of sequences obtained from the species listed in the legend for Figure 2. Letters indicate the amino acid types observed at each position. The height of each stack of symbols in each position is proportional to the information content at that position and the height of each letter within the stack is proportional to the frequency of the corresponding residue at that position. Residues involved in direct contacts with base, ribose or phosphate groups, as determined by homology to the known thermosome structures, are indicated.

We compared conservation in CCT, BBS and CCT8L sequences of the ATP-binding and ATP-hydrolysis motifs typical of chaperonins of Group II (Figure 7). Although there is considerable variation among BBS and CCT8L sequences at some of the ATP-binding positions, we observed complete conservation of the crucial ATP-binding dipeptide Gly-Pro, suggesting that these otherwise divergent proteins conserve ATP-binding ability. In the ATP-hydrolysis sites, substantial loss of conservation has been reported in MKKS [27] and in BBS12 [23]. In the CCT8L, MKKS and BBS10 families, unusual substitutions are observed in phosphate-binding positions and within the catalytic triad, where only Asp is conserved in MKKS. The effect that these mutations may have on the hydrolytic activity in these protein families is unclear. The high level of differentiation of this region in BBS12 (where the ATP-hydrolysis motif is not recognizable) strongly suggests that BBS12 has lost hydrolytic activity.

### Conservation of substrate-binding positions

Three positions crucial in determining substrate-specificity of CCT monomers have been identified in the distal region of helix I in the apical domain [37]. We analyzed conservation at these positions across vertebrate species in all Group II chaperonin families and in the Fab1_TCP domain across vertebrate orthologs of the PIKFYVE protein kinase (Table 6). These positions are strikingly conserved within each CCT monomer type (with the exception of CCT6B) across species and are characteristically different between monomer types. They are mostly conserved also in the Fab1_TCP domain across vertebrate sequences. In contrast, in BBS and, particularly, in CCT8L sequences, the homologous positions are significantly more differentiated.

**Table 6:** Conservation of potential substrate-binding residue positions^1^

Family	*I*^2^	*i *+1^2^	*i*+4^2^	Description
CCT1	K	Y	DE	Lys/Tyr/Acidic
CCT2	Q	L	A (GQ)^3^	Gln/Leu/Ala
CCT3	H	Y	KR	His/Tyr/Basic
CCT4	H	F	K	His/Phe/Lys
CCT5	H	L	Q	His/Leu/Gln
CCT6A	D	A	K	Asp/Ala/Lys
CCT6B	DE	AILMSV	K (R)	Acidic/Medium-Small/Lys
CCT7	Q	Y	D (Y)	Gln/Tyr/Asp
CCT8	H	Y	K	His/Tyr/Lys
CCT8L	DILPT	HLQR	KNRY	Variable/Variable/Polar-Basic
MKKS	Q (H)	FY (H)	DEMQST	Gln/Aromatic/Medium-Small
BBS10	Y (AFQS)	CLY (W)	LMQV	Tyr/Variable/Variable
BBS12	E (KLQ)	KR (HQ)	HNR (ASD)	Glu/Basic/Polar-Basic
Fab1_TCP^4^	D (EN)	I (LMV)	Q	Asp/Ile/Gln

1 Conservation evaluated among sequences in vertebrate genomes. 2 Potential substrate binding positions, corresponding to yeast CCT1 positions 308, 309 and 312 (*i *= 308) [37]. ^3^Rare substitutions are listed in parenthesis. ^4^Fab1_TCP domain of vertebrate PIKFYVE orthologs.

## Discussion

We identified the full complement of chaperonin *hsp60 *genes and pseudogenes encoded in the human genome and, for comparison, in the genomes of the model organisms mouse and rat. We delimited the set of *hsp60 *genes encoded in the human genome to: a) nine canonical *cct *genes (CCT1 to CCT8 including CCT6A and CCT6B) involved in formation of the CCT complex; b) the *cpn60 *gene (HSPD1) of mitochondrial origin; c) the three highly diverged *hsp60*-like BBS genes MKKS, BBS10 and BBS12; and d) a newly characterized class of genes, CCT8L, represented in human by CCT8L1 and CCT8L2. We also identified a plethora of pseudogene sequences, many of which had not been previously reported. The comparative analyses of these families of functional genes and of their pseudogenes revealed their evolutionary history and relationships.

In contrast to the uncertainty of the duplication pattern of canonical CCT subunits (our results and [38,39]) the origin of Hsp60-like BBS and CCT8L proteins was unambiguously identified by phylogenetic tree reconstructions. Our analyses indicated that *hsp60*-like BBS genes originated monophyletically from a gene duplication event in the CCT8 gene lineage. In addition, we determined that the CCT8L family also originated in the CCT8 lineage, from a more recent retrotransposition event. The presence of this gene family in placental mammals, marsupials and monotremes but not in reptiles/birds or other vertebrate species, indicates that this family originated at the onset of mammal evolution, before divergence of Theria and Prototheria. Presence of two highly similar CCT8L genes (CCT8L1 and CCT8L2) in the genomes of human and chimp and of a single copy in other mammal genomes, including rhesus monkey, suggests that the duplication of this gene occurred in the ape lineage (Hominoidea) after its divergence from the old-world monkeys (Cercopithecidae). Multiple evidence gathered in this work indicates that CCT8L sequences (and at least one of the two paralogs in Hominoidea) encode for functional genes: (i) reduced rates of non-synonymous mutation were estimated along their lineages, as expected for functionally-constrained protein-coding genes; (ii) pseudogenes as ancient or more recent than the CCT8L genes were heavily degenerated and no pseudogenes pre-dating mammal evolution could be identified. In contrast, although CCT8L sequences originated early in mammal evolution, they did not show signs of degeneration (with the exception of the chimp CCT8L1 ortholog); (iii) multiple EST and microarray data have been collected for CCT8L2, mostly from testis, and one EST for CCT8L1 has been reported from placental tissue (as per the UniGene EST and GEO expression data, November 23, 2009). These features taken together are strong evidence that at least CCT8L2 in Hominoidea and the lone CCT8L gene in other mammal lineages encode for functional proteins. The sparse expression of CCT8L1 in human and the presence of one in-frame stop codon and one frame-shift in its orthologous sequence from chimp raise doubts about the functionality of this sequence.

Numerous sequences associated with *cct *or *cpn60 *genes found in the human, mouse or rat genomes were classified as pseudogenes based on the presence of internal stop codons, frame-shifts and non-significant difference in synonymous and non-synonymous mutation rates. Among them, the sequences HSPD1-5P and HSPD1-6P appear to be expressed based on EST analysis (see additional file 14: Table S5) and may represent instances of expressed pseudogenes [40]. A general explosion of pseudogene generation in the human and murid lineages after they separated from the carnivore lineage has been reported [41]. Our analysis of chaperonin pseudogenes is consistent with this observation, although their relatively high rate of degeneration suggests that pseudogenes generated before the origin of mammals may have degraded beyond recognition. The intense duplication of chaperonin sequences witnessed by the many pseudogenes identified in the human and murid genomes, very likely provided opportunities for multiple paralogy, resulting in the proliferation of chaperonin classes in the vertebrate and mammal lineages.

Although the Hsp60-like BBS and CCT8L protein families have considerably differentiated from the canonical CCT subunits and within themselves, our analyses indicated that they still conserve the overall three-domain structure typical of CCT proteins. Structure and sequence variations predicted for their apical domains may reflect distinctive substrate specificities. In particular, lack of conservation at positions crucial in providing substrate-specificity to CCT monomers [37] suggests that BBS and CCT8L proteins may interact with their substrate(s) in different regions as compared with the canonical CCT subunits. Sequence differentiation patterns and acquisition of inserted elements in correspondence to potential monomer-monomer contact regions suggested that BBS and CCT8L proteins do not assemble in a CCT-like complex. This prediction is supported by experimental evidence showing that MKKS localizes as a free monomer at the pericentriolar material of centrosomes [27]. In this respect, it is also interesting to observe that among BBS and CCT8L sequences the ATP-hydrolysis motif "Gly-Asp-Gly-Thr", remarkably conserved among canonical chaperonins [42], has differentiated in MKKS and in BBS12 [23,27]. This condition may indicate that these families have lost the hydrolytic activity necessary for the functionality of the chaperonin complex [43-52]. It has been shown for the archaeal thermosome complex that mutation of the ATP-hydrolysis-motif Asp residue prevents hydrolysis and productive protein folding [49] and that some CCT subunits, among which CCT8, dissociate *in vitro *from the complex in conditions that prevent hydrolysis of ATP [53].

Functionalities independent from formation of the complex have also been reported for canonical CCT subunits. TCP1 monomers not in complex confer enhanced salt tolerance in plants [54]. Individual CCT subunits have been reported to associate *in vitro *with cytoskeleton structures, selectively binding to microtubule filaments [55] or to actin polymerizing filaments [56]. The localization of Hsp60-like BBS proteins at the cilium basal body and at the centrosome [26-28] suggests that they may also interact and associate with, for example, cytoskeleton structures in promoting the correct development of cilia [28,57]. The multiple structural and experimental evidence that BBS and CCT8L proteins do not form a canonical CCT-like complex provides strong indication that eukaryotic Group II chaperonin-protein functionalities extend beyond those of the typical oligomeric complex.

## Conclusions

Chaperonin proteins are key players in ensuring and preserving cell and organism functionality under normal and stressful conditions and their biological and medical importance is undeniable. The recent discovery of *hsp60 *genes directly implicated in specific pathological conditions, the chaperonopathies, extends our understanding of the roles of chaperonin proteins in cellular processes and enhances awareness of their importance in pathology [18-20]. Here, we have provided a comprehensive, unifying framework encompassing all members of the extended *hsp60 *family of genes and pseudogenes. This unifying framework contributes to our understanding of the evolutionary history of the extended *hsp60 *family and widens our perspectives on the multiple roles that chaperonin proteins have acquired in vertebrates. Our findings highlight how differentiation of the chaperonin protein family in mammals has been facilitated by intense processes of gene duplication. The roles, mechanisms of action, and involvement in pathogenesis of individual chaperonin molecules beyond those typical of their canonical oligomeric complexes constitute aspects of chaperonin physiology particularly promising for future experimental testing.

## Methods

### Identification of chaperonin genes in eukaryotic genomes

Searches of genes for Hsp60-like proteins were exhaustively performed using TBLASTN [58] at Ensembl [34] and BLAT [59] at UCSC [60] on the genome sequences of human (NCBI Assembly 36, Genebuild Ensembl Dec 2006), mouse (NCBI Assembly m37, Genebuild Ensembl Apr 2007) and rat (Assembly RGSC 3.4, Genebuild Ensembl Feb 2006). We used the nine canonical human CCT proteins and the Cpn60 protein (mitochondrial Hsp60) as queries. We recursively queried the genomes with the sequences recovered from previous searches until no other Hsp60 sequences were detected. We used both search engines also to recover the full list of annotated *hsp60*-like genes in several other mammal genomes and in chicken. Sequences from frog (*Xenopus *sp.) were retrieved from the NCBI nr (non-redundant) database using PSI-BLAST [61] with Cpn60 and the individual CCT subunits as queries. To recover complete *hsp60 *gene and pseudogene sequences, after the TBLASTN searches the genomic sequences from approximately 2,000 nt upstream to 2,000 nt downstream of the hit-regions were excised and the *hsp60 *sequences were extracted using the homology-based gene prediction method implemented in FGENESH+ [62] at the Softberry web site [63]. For pseudogenes, when FGENESH+ failed to recognize the complete sequence due to in-frame stop codons or frame shifts in the sequence, the coding region was manually reconstructed, aligning the three-frame-translations of the genomic sequence to the query sequence with the multiple protein alignment program ITERALIGN [64]. The Pseudogene.org [33,65] database and Ensembl [34], Entrez [30] and HUGO [66] annotations were consulted for the presence of annotated human pseudogenes, as recorded in our tables of results.

### Multiple sequence alignment and secondary structure prediction

Multiple sequence alignments were obtained using MUSCLE [67], which in previous analyses [68,69] performed well when aligning divergent sequences. Alignments were manually adjusted as needed. Predictions of secondary structure for each protein family were performed from their multiple alignment using the Jnet algorithm as implemented in the JPRED-3 secondary structure prediction server [70,71].

### Evolutionary tree reconstructions

To infer phylogenetic relationships, evolutionary trees were obtained using the maximum-likelihood (ML) tree-building procedure implemented in PHYML [72] using the default JTT substitution model and 100 bootstrap resampling replicates (each ML tree reconstruction being quite time consuming). Selected trees were compared with those obtained with the Bayesian approach implemented in MrBayes 3.1 [73] using the WAG substitution model and 10,000 iterations for the MCMC process. Conditional probabilities were estimated sampling the MCMC process every 10 iterations after 2,500 burn-in iterations (sample size 750).

### Estimates of evolutionary divergence of sequence families

We obtained rates of divergence among families of sequences using a newly developed estimator, called "B-index". The B-index is an unbiased estimator of the average divergence of a family of sequences from its last common ancestor (root) that takes into consideration the correlations among sequences determined by their phylogenetic tree. Briefly, given a rooted tree, a terminal branch of length *d*_*i *_of the original tree is considered a "cluster" of size *w*_*i *_= 1 and length *d *= *d*_*i*_. Each fork-structure comprising two terminal branches (clusters) of lengths *d*_1 _and *d*_2 _and sizes *w*_1 _and *w*_2 _bifurcating from a stem-branch of length *d*_*s *_is considered in turn. The average length *d *of each fork-structure is computed as *d *= (*d*_1 _+ *d*_2_)/2 + *d*_*s *_and the average size *w *of the structure is defined as *w *= [2(*d*_1 _+ *d*_2_)/2 + 1*d*_*s*_]/[(*d*_1 _+ *d*_2_)/2 + *d*_*s*_] = (*d*_1 _+ *d*_2 _+ *d*_*s*_)/*d*. Each fork-structure is progressively replaced by a corresponding cluster of length *d *and size *w*. The procedure is repeated merging bifurcating clusters of lengths *d*_1 _and *d*_2 _and sizes *w*_1 _and *w*_2 _connected to a stem-branch of length *d*_*s *_into a larger cluster of average length *d *= (*w*_1_*d*_1 _+ *w*_2_*d*_2_)/(*w*_1 _+ *w*_2_) + *d*_*s *_and average size *w *= (*d*_1_*w*_1 _+ *d*_2_*w*_2 _+ *d*_*s*_)/*d*, until the tree is reduced to two clusters connected to the root (*d*_*s *_= 0). The global average differentiation *D *("B-index") and size *W *can finally be computed as *D *= (*w*_1_*d*_1 _+ *w*_2_*d*_2_)/(*w*_1 _+ *w*_2_) and *W *= *w*_1 _+ *w*_2_. It can be shown that *DW *= *L *is the length of the tree (sum of all branch lengths). If two sequence families *A *and *B *are sampled from the same set of species and *W*_*A *_= *W*_*B*_, then *D*_*B*_/*D*_*A *_= *L*_*B*_/*L*_*A *_and the relative rate of differentiation of the two families of sequences can be estimated by the ratio of their tree lengths. The B-index has several advantages compared to the most commonly used average pair-wise sequence-similarity measure: (i) it takes into account the correlation among sequences imposed by the topology of the evolutionary tree; (ii) in contrast to average pair-wise similarity, its expectations are invariant over the number and phylogenetic relations of sequences sampled from a cluster with the same common ancestor and evolutionary model; and (iii) with the B-index, the average differentiation rate of a protein family relative to a reference family sharing the same evolutionary relations (e.g., sampled from the same set of species) is simply estimated by the ratio of the lengths of the evolutionary trees of the two families.

### Estimates of ratios of non-synonymous vs. synonymous mutation rate (Ka/Ks)

Classification of *hsp60 *sequences as functional genes or pseudogenes was supported by the absence or presence of in-frame stop codons and frame-shifts, and by estimating non-synonymous *vs. *synonymous mutation-rate ratios (Ka/Ks) along relevant branches of evolutionary trees. Estimates were obtained using the maximum-likelihood branch-specific model implemented in PAML4 [74]. In the case of pseudogenes, Ka/Ks values are expected not to significantly differ from 1 (absence of positive or negative selection at the protein level) whereas protein-coding genes, whose evolution is dominated by negative or positive selection, are expected to be characterized, respectively, by Ka/Ks < 1 or Ka/Ks > 1. Briefly, we applied the PAML4 "branch-specific model" creating an evolutionary tree including the sequences whose evolutionary lineage was tested, the appropriate sister sequence (in the case of pseudogenes, the gene sequence from whose lineage the pseudogene originated) and an out-group sequence. The tree branch(es) to be tested are designated as "foreground" and other branches as "background." Using the branch-specific model the Ka/Ks ratio is estimated for the foreground branch(es) and an analogous ratio is estimated for the background branches. The likelihood *L*_1 _generated using this evolutionary model is compared to the likelihood *L*_0 _of a null model where Ka/Ks for foreground branches is fixed to 1.0. In the Log-likelihood Ratio Test (LRT) the significance of the likelihood differences between the model with free estimate of Ka/Ks and the null model is estimated by the quantity 2•ln(*L*_1_/*L*_0_), which approximates a χ^2 ^distribution.

### Data availability

All relevant gene and pseudogene information, including start and end positions, chromosomal location, strand, number of exons, GenBank accession number for functional genes, and Ensembl or Pseudogene.org ID for pseudogenes, can be found in additional file 22: Table S7. Newly annotated sequences have been approved and deposited in the Human Genome Organization (HUGO) database [66].

## Abbreviations

BBS: Bardet-Biedl Syndrome; CCT: Chaperonin Containing TCP1; ML: Maximum-Likelihood; MMKS: McKusick-Kaufman Syndrome; TRiC: TCP1 Ring Complex.

## Authors' contributions

KM participated in research and methodological approach design, carried out all searches and most data analyses, wrote drafts of the manuscript and participated in its refinement, compiled all tables and produced most figures; EC de M and AJLM envisioned the research project, started data collection and participated in research design and in manuscript preparation; LB participated in research design and methodological approach, produced differentiation and mutation-accumulation estimates and analyses and participated in writing the manuscript. All authors read and approved the final manuscript.

## Supplementary Material

Additional file 1 **Table S1**. Mouse *hsp60 *genes and pseudogenes. Click here for file

Additional file 2 **Table S2**. The rat *hsp60 *genes and pseudogenes. Click here for file

Additional file 3 **Figure S1**. Phylogenetic tree of human CCT1-8 and CCT8L proteins. Click here for file

Additional file 4 **Figure S2**. Phylogenetic tree of human CCT1-8 and MKKS proteins. Click here for file

Additional file 5 **Figure S3**. Phylogenetic tree of human CCT1-8 and BBS10 proteins. Click here for file

Additional file 6 **Figure S4**. Phylogenetic tree of human CCT1-8 and BBS12 proteins. Click here for file

Additional file 7 **Figure S5**. Phylogenetic tree of vertebrate CCT1-8, MKKS, BBS10, BBS12 and CCT8L proteins. Click here for file

Additional file 8 **Figure S6**. Phylogenetic trees of CCT8L protein sequences from primates (a,b) and partial alignment showing a divergent region in the sequence from rhesus monkey (c). Click here for file

Additional file 9 **Table S3**. Codon-base specific counts of mutation events along human and chimp CCT8L evolutionary branches. Click here for file

Additional file 10 **Table S4**. Expression pattern (EST counts) of the human CCT and BBS genes from the UniGene database. Click here for file

Additional file 11 **Figure S7**. Evolutionary tree of vertebrate CCT1-8 and CCT8L proteins including associated human pseudogenes. Click here for file

Additional file 12 **Figure S8**. Evolutionary trees of individual CCT1, CCT3 and CCT4 proteins from vertebrates including associated human pseudogenes. Click here for file

Additional file 13 **Figure S9**. Evolutionary trees of individual CCT5, CCT7 and CCT8 proteins from vertebrates including associated human pseudogenes. Click here for file

Additional file 14 **Table S5**. Expression pattern of the human *cpn60 *gene (HSPD1) and pseudogenes from the UniGene database. Click here for file

Additional file 15 **Figure S10**. Alignment and secondary-structure prediction of archaeal thermosome sequences. Click here for file

Additional file 16 **Table S11**. Alignment and secondary-structure prediction of human CCT1-8 protein sequences. Click here for file

Additional file 17 **Table S12**. Alignment and secondary-structure prediction of vertebrate CCT8L protein sequences. Click here for file

Additional file 18 **Table S13**. Alignment and secondary-structure prediction of vertebrate MKKS protein sequences. Click here for file

Additional file 19 **Table S14**. Alignment and secondary-structure prediction of vertebrate BBS10 protein sequences. Click here for file

Additional file 20 **Table S15**. Alignment and secondary-structure prediction of vertebrate BBS12 protein sequences. Click here for file

Additional file 21 **Table S6**. Residue types at monomer-monomer interaction positions in thermosome, CCT, BBS, and CCT8L proteins. Click here for file

Additional file 22 **Table S7**. Database and sequence information on all hsp60-like sequences identified in the human, mouse and rat genomes. Click here for file
