# Supplementary material for: Chaperonin genes on the rise: new divergent classes and intense duplication in human and other vertebrate genomes
Source: BMC Evol Biol. 2010 Mar 1;10:64. doi: 10.1186/1471-2148-10-64 (PMC2846930; doi:10.1186/1471-2148-10-64)
Supplement: Additional file 1 — Table S1. Mouse hsp60 genes and pseudogenes. [file 1471-2148-10-64-S1.DOC]

Table S1. The mouse *hsp60* genes and pseudogenes

| **A. Genes** | | | | | | |
| --- | --- | --- | --- | --- | --- | --- |
| **Name** | **Start^1^** | **End^2^** | **Str^3^** | **Chr^4^** | **Loc^5^** | **Ex^6^** |
| Cct1 | 13,109,331 | 13,117,933 | + | 17 | A2 | 12 |
| Cct2 | 116,490,071 | 116,500,106 | - | 10 | qD2 | 14 |
| Cct3 | 88,103,257 | 88,125,467 | + | 3 | qF1 | 13 |
| Cct4 | 22,890,754 | 22,902,933 | + | 11 | qA3.3 | 13 |
| Cct5 | 31,520,686 | 31,531,460 | - | 15 | qB3.2 | 11 |
| Cct6a | 130,293,315 | 130,321,693 | + | 5 | F | 13 |
| Cct6b | 82,532,867 | 825,77,729 | - | 11 | B5 | 14 |
| Cct7 | 85,409,109 | 85,418,268 | + | 6 | D1 | 11 |
| Cct8 | 87,484,236 | 87,496,033 | - | 16 | qC3.3 | 15 |
| Gm443 (CCT8L) | 25,022,107 | 25,023,792 | + | 5 | qA3 | 1 |
| Mkks | 136,700,005 | 136,706,971 | - | 2 | qF3 | 4 |
| Bbs10 | 110,735,779 | 110,738,219 | + | 10 | qD1 | 4 |
| Bbs12 | 37,217,982 | 37,220,105 | + | 3 | qB | 1 |
| Hspd1 | 55,135,135 | 55,143,783 | - | 1 | qC1.2 | 11 |

| **B. Cct-related pseudogenes** | | | | | | |
| --- | --- | --- | --- | --- | --- | --- |
| **Name** | **Start^1^** | **End^2^** | **Str^3^** | **Chr^4^** | **Loc^5^** | **Ex^6^** |
| Cct1-1P | 67,963,157 | 67,964,324 | - | 4 | qC1 | 3 |
| Cct1-2P | 13,129,036 | 13,136,541 | + | 17 | qA1 | 2 |
| Cct3-1P | 90,721,084 | 90,722,575 | - | 11 | qC | 5 |
| Cct3-2P | 52,913,471 | 52,915,552 | + | 4 | B3 | 3 |
| Cct3-3P | 113,303,406 | 113,303,687 | - | 6 | qE3 | 1 |
| Cct4-1P | 26,227,193 | 26,228,371 | - | 7 | qA3 | 3 |
| Cct6A-1P | 52,361,244 | 52,362,111 | + | 8 | qB1.3 | 3 |
| Cct6A-2P | 97,306,133 | 97,306,902 | - | 14 | qE2.1 | 2 |
| Cct6A-3P | 79,544,122 | 79,545,468 | + | 18 | qE3 | 1 |
| Cct7-1P | 12,863,162 | 12,881,543 | + | X | A1.3 | 5 |
| Cct7-2P | 87,401,390 | 87,403,113 | + | 18 | E4 | 5 |
| Cct7-3P | 20,811,426 | 20,812,537 | + | 1 | qA4 | 4 |
| Cct7-4P | 48,381,857 | 48,382,517 | - | 11 | qB1.2 | 1 |
| Cct7-5P | 58,647,550 | 58,647,741 | + | 13 | qB1 | 1 |
| Cct8-1P | 51,795,721 | 51,795,882 | + | 2 | qC1.1 | 1 |
| Cct8-2P | 35,774,568 | 35,782,271 | - | 3 | qB | 3 |

**Table S1 (continued)**

| **C. Hspd1-related pseudogenes** | | | | | | |
| --- | --- | --- | --- | --- | --- | --- |
| **Name** | **Start^1^** | **End^2^** | **Str^3^** | **Chr^4^** | **Loc^5^** | **Ex^6^** |
| Hspd1-1P^7^ | 41,312,239 | 41,313,954 | + | 11 | qA5 | 1 |
| Hspd1-2P | 105,808,716 | 105,810,383 | - | 4 | qC7 | 1 |
| Hspd1-3P | 101,742,761 | 101,744,429 | + | 14 | qE2.3 | 5 |
| Hspd1-4P | 64,965,611 | 64,967,286 | - | 8 | qB3.1 | 3 |
| Hspd1-5P | 85,271,516 | 85,279,593 | + | 8 | qC2 | 6 |
| Hspd1-6P | 49,455,871 | 49,457,564 | - | 1 | qC1.1 | 5 |
| Hspd1-7P | 77,889,757 | 77,891,210 | - | 3 | qE3 | 3 |
| Hspd1-8P | 89,108,911 | 89,111,283 | - | 4 | qC4 | 5 |
| Hspd1-9P | 11,672,585 | 11,673,644 | - | 12 | qA1.1 | 1 |
| Hspd1-10P | 93,743,990 | 93,745,672 | + | X | qC3 | 2 |
| Hspd1-11P | 12,515,616 | 12,517,225 | + | 6 | qA1 | 3 |
| Hspd1-12P | 45,234,912 | 45,235,851 | + | 1 | qC1.1 | 2 |
| Hspd1-13P | 24,133,639 | 24,135,093 | - | 15 | qA2 | 2 |
| Hspd1-14P | 79,001,216 | 79,001,752 | + | 2 | qC3 | 1 |
| Hspd1-15P | 12,819,585 | 12,820,103 | + | 9 | qA1 | 1 |
| Hspd1-16P | 61,872,231 | 61,873,436 | - | 3 | qE1 | 1 |
| Hspd1-17P | 5,053,347 | 5,054,702 | + | 1 | qA1 | 2 |
| Hspd1-18P | 58,011,087 | 58,011,631 | - | 8 | qB2 | 1 |
| Hspd1-19P | 61,460,831 | 61,461,298 | + | 16 | qC1.3 | 1 |
| Hspd1-20P | 98,441,180 | 98,441,466 | + | 2 | qE1 | 2 |
| Hspd1-21P | 91,375,464 | 91,376,660 | + | 4 | qC5 | 2 |
| Hspd1-22P | 48,192,945 | 48,193,097 | - | 17 | qC | 1 |

^1^Start and ^2^End of the gene or pseudogene in the genome; ^3^Strand; ^4^Chromosome; ^5^Location; ^6^Number of exons; ^7^Identified in Ensembl.
