# Supplementary material for: Chaperonin genes on the rise: new divergent classes and intense duplication in human and other vertebrate genomes
Source: BMC Evol Biol. 2010 Mar 1;10:64. doi: 10.1186/1471-2148-10-64 (PMC2846930; doi:10.1186/1471-2148-10-64)
Supplement: Additional file 2 — Table S2. The rat hsp60 genes and pseudogenes. [file 1471-2148-10-64-S2.DOC]

Table S2. The rat *hsp60* genes and pseudogenes

| **A. Genes** | | | | | | |
| --- | --- | --- | --- | --- | --- | --- |
| **Name** | **Start^1^** | **End^2^** | **St^3^** | **Chr^4^** | **Loc^5^** | **Ex^6^** |
| Tcp1 | 42,103,629 | 42,111,145 | - | 11 | q12 | 12 |
| Cct2 | 56,394,922 | 56,402,261 | - | 7 | q22 | 9 |
| Cct3 | 180,383,106 | 180,433,933 | + | 2 | q34 | 12 |
| Cct4 | 103,675,244 | 103,687,376 | + | 14 | q22 | 13 |
| CCt5 | 83,681,899 | 83,692,935 | - | 2 | q22 | 11 |
| Cct6a | 27,992,366 | 28,002,203 | + | 12 | q13 | 14 |
| Cct6b (L63658) | 72,107,176 | 72,144,991 | - | 10 | q26 | 11 |
| Cct7 | 119,714,400 | 119,722,770 | + | 4 | q34 | 11 |
| Cct8 | 27,234,460 | 27,245,292 | - | 11 | q11 | 15 |
| Cct8L (125233) | 5,004,090 | 5,005,772 | - | 4 | q11 | 1 |
| Mkks | 124,975,607 | 124,981,825 | - | 3 | q36 | 4 |
| BBS10 (60748) | 50,317,911 | 50,320,406 | + | 7 | q21 | 2 |
| BBS12 (61608) | 123866726 | 123868843 | + | 2 | q25 | 1 |

Table S2 (continued 1)

| **B. Cct-related pseudogenes** | | | | | | |
| --- | --- | --- | --- | --- | --- | --- |
| **Name** | **Start ^1^** | **End ^2^** | **St^3^** | **Chr^4^** | **Loc^5^** | **Ex^6^** |
| Cct1-1P | 122,326,746 | 122,328,035 | - | 4 | q34 | 1 |
| Cct2-1P | 94,540,595 | 94,541,059 | + | X | q31 | 2 |
| Cct3-1P | 21,483,523 | 21,485,110 | + | 19 | p11 | 3 |
| Cct3-2P | 51,648,197 | 51,649,727 | - | 13 | q13 | 4 |
| Cct3-3P | 93,409,548 | 93,410,990 | + | X | q31 | 3 |
| Cct3-4P | 149,237,097 | 149,237,635 | - | 4 | q42 | 2 |
| Cct5-1P | 111,419,580 | 111,419,580 | - | X | q32 | 2 |
| Cct5-2P | 17,626,224 | 17,626,583 | - | 8 | q13 | 1 |
| Cct6A-1P | 31,685,049 | 31,688,384 | - | 8 | q13 | 5 |
| Cct6A-2P | 88,702,370 | 88,703,903 | + | 10 | q32.1 | 2 |
| Cct6A-3P | 240,279 | 241,905 | + | 5 | q11 | 3 |
| Cct6A-4P | 76,957,850 | 76,959,406 | - | 5 | q24 | 3 |
| Cct6A-5P | 35,236,443 | 35,237,988 | - | 18 | p11 | 4 |
| Cct6A-6P | 67,148,659 | 67,170,130 | - | 1 | q12 | 5 |
| Cct6A-7P | 1,252,980 | 1,254,555 | - | 1 rnd^7^ | N.A.^8^ | 5 |
| Cct6A-8P | 79,050,104 | 79,053,455 | - | 13 | q22 | 5 |
| Cct6A-9P | 88,811,021 | 88,812,383 | - | 16 | q12.5 | 4 |
| Cct6A-10P | 67,148,933 | 67,150,124 | - | 1 | q12 | 3 |
| Cct6A-11P | 83,591,559 | 83,592,798 | + | 6 | q23 | 5 |
| Cct6A-12P | 50,508,448 | 50,509,665 | - | 9 | q22 | 4 |
| Cct6A-13P | 66,456,610 | 66,457,113 | - | 14 | q11 | 1 |
| Cct6A-14P | 67,168,555 | 67,168,845 | - | 1 | q12 | 1 |
| Cct6A-15P | 8,728,585 | 8,728,815 | + | 5 | q11 | 2 |
| Cct6A-16P | 76,283,952 | 76,284,215 | + | 18 | q12.3 | 1 |
| Cct7-1P | 216,186,823 | 216,188,454 | + | 1 | q43 | 1 |
| Cct7-2P | 145,306,136 | 145,307,710 | + | 5 | q36 | 3 |
| Cct7-3P | 19,556,657 | 19,557,897 | + | 9 | q13 | 3 |
| Cct8-1P | 43,940,890 | 43,941,006 | + | 11 | q12 | 1 |
| Cct8-2P | 32,962,674 | 32,962,763 | + | 3 | q12 | 1 |

Table S2 (continued 2)

| **C. Hspd1-related pseudogenes** | | | | | | |
| --- | --- | --- | --- | --- | --- | --- |
| **Name** | **Start ^1^** | **End ^2^** | **St^3^** | **Chr^4^** | **Loc^5^** | **Ex^6^** |
| Hspd1-1P | 107,651,987 | 107,653,696 | + | 14 | q22 | 1 |
| Hspd1-2P | 42,554,408 | 42,556,112 | + | 14 | p11 | 1 |
| Hspd1-3P | 58,629,782 | 58,633,066 | + | 17 | q12.1 | 2 |
| Hspd1-4P | 41,523,338 | 41,525,050 | - | 14 | p11 | 2 |
| Hspd1-5P | 179,041,398 | 179,043,070 | - | 1 | q35 | 2 |
| Hspd1-6P | 30,691,244 | 30,692,932 | + | 20 | q11 | 4 |
| Hspd1-7P | 103,951,550 | 103,953,587 | - | 1 | q22 | 2 |
| Hspd1-8P | 3,553,285 | 3,554,991 | + | 13 | p13 | 1 |
| Hspd1-9P | 234,250,749 | 234,252,288 | + | 2 | q43 | 1 |
| Hspd1-10P | 76,955,304 | 76,956,924 | - | 5 | q24 | 4 |
| Hspd1-11P | 19,170,317 | 19,172,008 | + | 6 | q12 | 6 |
| Hspd1-12P | 220,584,917 | 220,585,779 | + | 2 | q42 | 1 |
| Hspd1-13P | 43,887,785 | 43,896,197 | + | 9 | q22 | 4 |
| Hspd1-14P | 109,039,079 | 109,040,435 | - | 5 | q32 | 4 |
| Hspd1-15P | 37,890,620 | 37,892,172 | + | 4 | q21 | 5 |
| Hspd1-16P | 110,646,161 | 110,647,153 | + | X | q32 | 1 |
| Hspd1-17P | 84,112,821 | 84,114,244 | + | X | q31 | 4 |
| Hspd1-18P | 46,394,084 | 46,394,824 | - | 9 | q22 | 1 |
| Hspd1-19P | 52,019,335 | 52,019,963 | - | 20 | q13 | 1 |
| Hspd1-20P | 8,089,404 | 8,090,167 | + | 15 | p16 | 2 |
| Hspd1-21P | 132,806,121 | 132,806,537 | + | X | q35 | 1 |
| Hspd1-22P | 58562739 | 58,563,142 | - | 17 | q12.1 | 1 |
| Hspd1-23P | 151675083 | 151,676,174 | - | 2 | q31 | 4 |
| Hspd1-24P | 414366 | 414,686 | - | 15 rnd^7^ | N.A.^8^ | 1 |
| Hspd1-25P | 75608618 | 75,611,557 | - | 2 | q22 | 2 |
| Hspd1-26P | 80028738 | 80,029,070 | - | 6 | q23 | 1 |
| Hspd1-27P | 11777078 | 11,777,682 | + | 7 | q11 | 2 |
| Hspd1-28P | 62800517 | 62,800,732 | + | 2 | q16 | 1 |
| Hspd1-29P | 14696828 | 14,698,283 | - | 5 | q12 | 5 |
| Hspd1-30P | 53891997 | 53,892,110 | - | 9 | q31 | 1 |
| Hspd1-31P | 53483173 | 53,483,310 | + | 4 | q22 | 1 |
| Hspd1-32P | 30834360 | 30,834,470 | - | 4 | q13 | 1 |

^1^Start and ^2^End of the gene/pseudogene in the genome; ^3^Strand; ^4^Chromosome; ^5^Location; ^6^Number of exons; ^7^Location not known; ^8^N.A., Not Available.
