# Supplementary material for: Chaperonin genes on the rise: new divergent classes and intense duplication in human and other vertebrate genomes
Source: BMC Evol Biol. 2010 Mar 1;10:64. doi: 10.1186/1471-2148-10-64 (PMC2846930; doi:10.1186/1471-2148-10-64)
Supplement: Additional file 4 — Figure S2. Phylogenetic tree of human CCT1-8 and MKKS proteins. [file 1471-2148-10-64-S4.PDF]

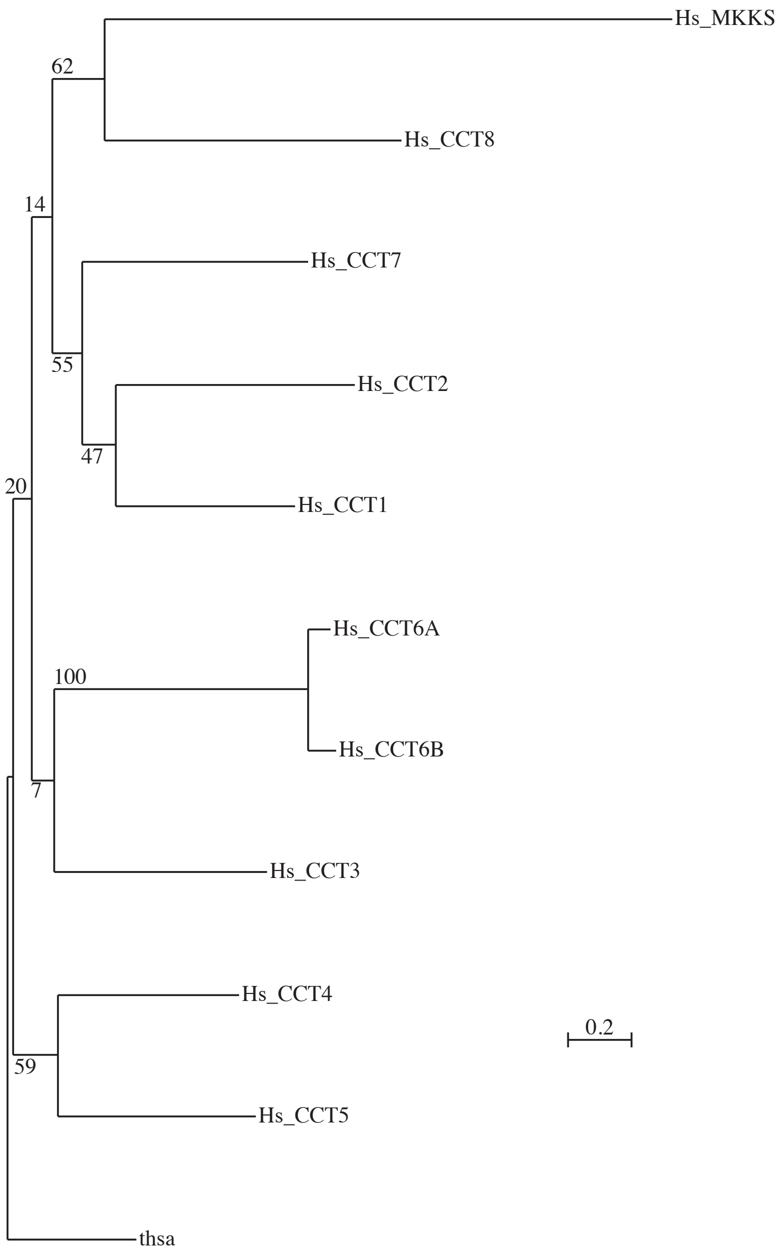

Figure S2. ML tree of human CCT proteins with MKKS (BBS6), excluding other BBS proteins and CCT8L proteins. "thsa" represents the *Thermoplasma acidophilum* alpha subunit of the thermosome. The scale bar represents the indicated number of substitutions per position for a unit branch length.
