# Supplementary material for: Chaperonin genes on the rise: new divergent classes and intense duplication in human and other vertebrate genomes
Source: BMC Evol Biol. 2010 Mar 1;10:64. doi: 10.1186/1471-2148-10-64 (PMC2846930; doi:10.1186/1471-2148-10-64)
Supplement: Additional file 7 — Figure S5. Phylogenetic tree of vertebrate CCT1-8, MKKS, BBS10, BBS12 and CCT8L proteins. [file 1471-2148-10-64-S7.PDF]

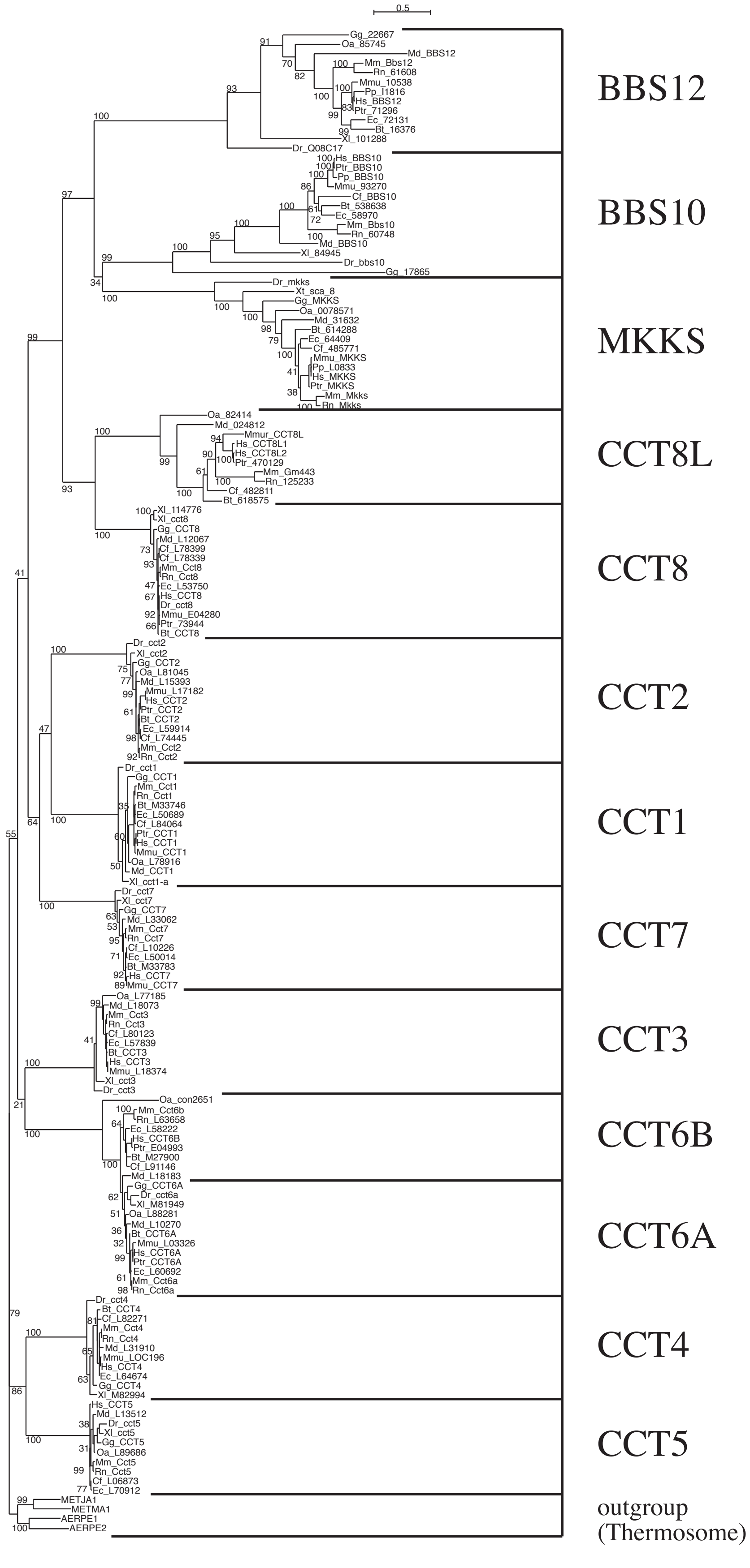


Figure S5. ML tree of the CCT proteins from vertebrate lineages (mammals, birds/reptiles, amphibians, fish) showing the relations of the BBS and CCT8L Classes with CCT8. Species abbreviations: Bt, *Bos Taurus*; Cf, *Canis familiaris*; Dr, *Danio rerio*; Ec, *Equus caballus*; Gg, *Gallus gallus*; Hs, *Homo sapiens*; Md, *Monodelphis domestica*; Mm, *Mus musculus*; Mmu, *Macaca mulatta*; Mmur, *Microcebus murinus*; Oa, *Ornithorhynchus anatinus*; Ptr, *Pan troglodytes*; Rn, *Rattus norvegicus*; Xl, *Xenopus laevis*; Xt, *Xenous tropicalis*; METJA, *Methanocaldococcus jannaschii* (Euryarchaeota); METMA, *Methanosarcina mazei* (Euryarchaeota); AERPE, *Aeropyrum pernix* (Crenarchaeota). The scale bar represents the indicated number of substitutions per position for a unit branch length.
