# Supplementary material for: Chaperonin genes on the rise: new divergent classes and intense duplication in human and other vertebrate genomes
Source: BMC Evol Biol. 2010 Mar 1;10:64. doi: 10.1186/1471-2148-10-64 (PMC2846930; doi:10.1186/1471-2148-10-64)
Supplement: Additional file 8 — Figure S6. Phylogenetic trees of CCT8L protein sequences from primates (a,b) and partial alignment showing a divergent region in the sequence from rhesus monkey (c). [file 1471-2148-10-64-S8.PDF]

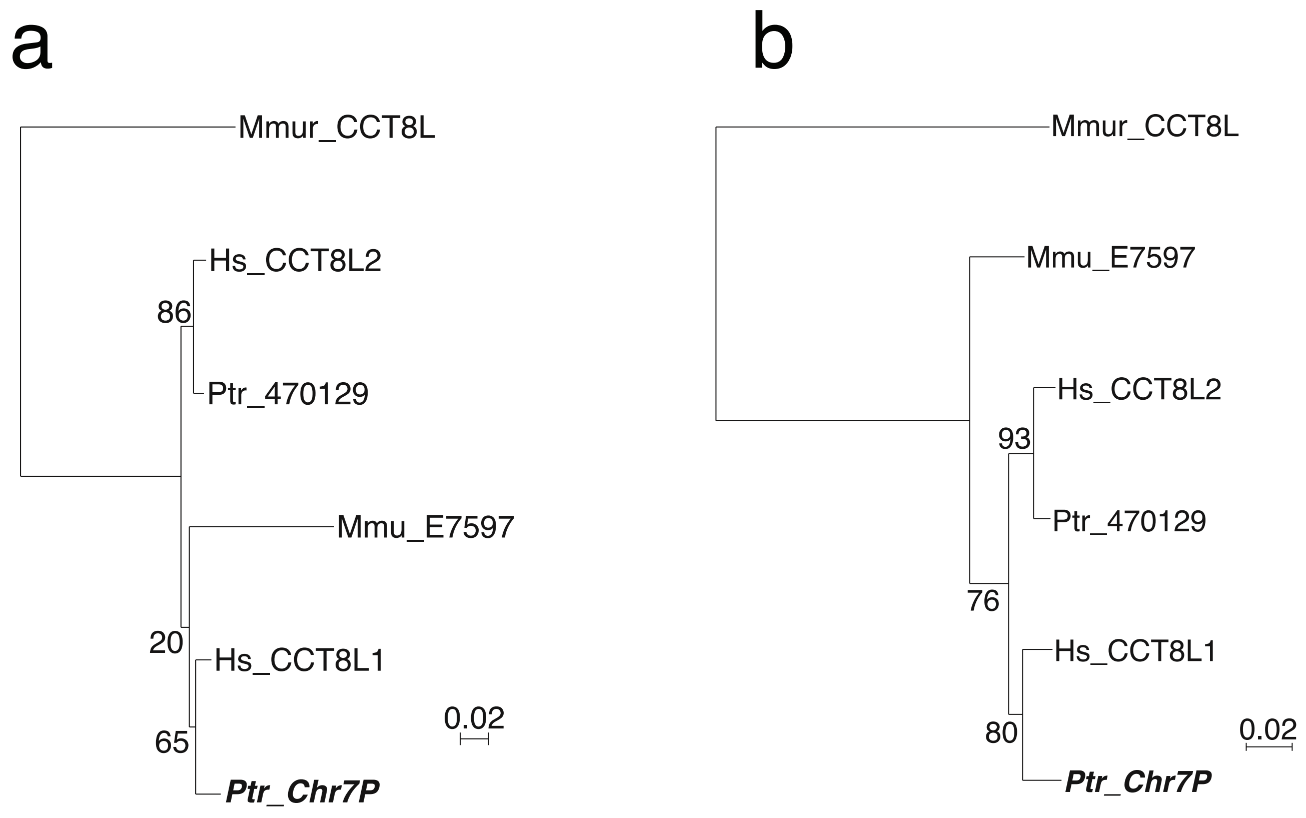

c

```
Mmur_CCT8L   GAGLPRPQLREAYAAAMAEVLST-LPTLAIRS-------LG-PL-ED-PSWALYSVMNTHTLSHTHYLTKLVAHACWAAREL
Mmu_E7597    KAGLPR-RTREATHS-TAEYCHTALPGHPI-SGAFGRSVLGSPFCDEYPHPVLHG-------PP----DQAGGPCCWAIKEL
Hs_CCT8L2    KAGLPRPQLREAYATATAEVLAT-LPSLAIQS-------LG-PL-ED-PSWALHSVMNTHTLSPMDHLTKLVAHACWAIKEL
Ptr_470129   KAGLPRPQLLEAYATATAEVLAT-LPSLAIQS-------LG-PL-ED-PSWALHSVMNTHTLSPMDHLTKLVAHACWAIKEL
Hs_CCT8L1    KFGLPRPQLREAYATATAEVLAT-LPSLAIQS-------LG-PL-ED-PSWALHSVMNTHTLPPMNHLTKLVAHACWAIKEL
Ptr_Chr7P    KAGLPRPQLREAYSTATAEVLAT-LPSLAIQS-------LG-PL-ED-PSWALHSVMNTYTLPPMDHLTKLVAHAC-AIKEL
```

Figure S6. Evolutionary trees of primate CCT8L sequences obtained: (**a**) from the alignment of the complete sequence; (**b**) removing a 50 amino acid region highly diverged in the sequence from rhesus monkey (Mmu) (**c**) Alignment of CCT8L sequence segments from different primate species showing in yellow diverged positions 132-181 from the rhesus monkey sequence. See legend to Figure 2 for species name abbreviations.
