# Supplementary material for: Chaperonin genes on the rise: new divergent classes and intense duplication in human and other vertebrate genomes
Source: BMC Evol Biol. 2010 Mar 1;10:64. doi: 10.1186/1471-2148-10-64 (PMC2846930; doi:10.1186/1471-2148-10-64)
Supplement: Additional file 9 — Table S3. Codon-base specific counts of mutation events along human and chimp CCT8L evolutionary branches. [file 1471-2148-10-64-S9.DOC]

Table S3. Lineage-specific mutation events along human and chimp CCT8L evolutionary branches.^1^

| **Name** | **Branch No.^2^** | **Codon base** | | |
| --- | --- | --- | --- | --- |
|  |  | **I** | **II** | **III** |
| Human CCT8L1 | 1 | 4 | 3 | 5 |
| Chimp CCT8L1 | 2 | 6 | 3 | 5 |
| Common CCT8L1 | 3 | 2 | 1 | 2 |
| Total CCT8L1 |  | 12 | 7 | 12 |
| Human CCT8L2 | 4 | 3 | 1 | 5 |
| Chimp CCT8L2 | 5 | 1 | 3 | 3 |
| Common CCT8L2 | 6 | 2 | 4 | 7 |
| Total CCT8L2 |  | 6 | 8 | 15 |

^1^Number of substitutions events inferred by parsimony along the indicated evolutionary branches; ^2^Branch numbers refer to the schematic evolutionary tree represented in Figure 3.
