# Supplementary material for: Chaperonin genes on the rise: new divergent classes and intense duplication in human and other vertebrate genomes
Source: BMC Evol Biol. 2010 Mar 1;10:64. doi: 10.1186/1471-2148-10-64 (PMC2846930; doi:10.1186/1471-2148-10-64)
Supplement: Additional file 10 — Table S4. Expression pattern (EST counts) of the human CCT and BBS genes from the UniGene database. [file 1471-2148-10-64-S10.DOC]

Table S4. Expression pattern of the human CCT genes1

| **Body tissue/site** | **CCT** | | | | | | | | | | | **BBS** | | |
| --- | --- | --- | --- | --- | --- | --- | --- | --- | --- | --- | --- | --- | --- | --- |
|  | **1** | **2** | **3** | **4** | **5** | **6A** | **6B** | **7** | **8** | **8L1** | **8L2** | **6** | **10** | **12** |
| adipose | 3 | 0 | 5 | 4 | 2 | 1 | 0 | 6 | 5 | 0 | 0 | 0 | 1 | 0 |
| adrenal | 16 | 4 | 23 | 12 | 17 | 6 | 0 | 28 | 13 | 0 | 0 | 2 | 1 | 1 |
| ascites | 11 | 15 | 48 | 18 | 25 | 10 | 0 | 43 | 13 | 0 | 0 | 1 | 0 | 0 |
| bladder | 14 | 14 | 19 | 10 | 19 | 5 | 1 | 14 | 17 | 0 | 0 | 1 | 0 | 2 |
| blood | 44 | 28 | 50 | 16 | 64 | 19 | 0 | 58 | 36 | 0 | 0 | 4 | 1 | 3 |
| bone | 8 | 29 | 23 | 31 | 20 | 25 | 0 | 21 | 14 | 0 | 0 | 2 | 2 | 1 |
| bone marrow | 7 | 12 | 26 | 6 | 18 | 11 | 0 | 9 | 14 | 0 | 0 | 0 | 0 | 0 |
| brain | 494 | 162 | 655 | 281 | 424 | 118 | 10 | 663 | 417 | 0 | 4 | 105 | 36 | 18 |
| cervix | 8 | 8 | 65 | 26 | 38 | 16 | 1 | 55 | 21 | 0 | 0 | 3 | 5 | 0 |
| connective | 26 | 17 | 69 | 96 | 25 | 20 | 1 | 50 | 27 | 0 | 0 | 9 | 0 | 4 |
| ear | 4 | 1 | 4 | 1 | 0 | 6 | 0 | 0 | 9 | 0 | 0 | 0 | 0 | 1 |
| embryonic | 48 | 84 | 118 | 59 | 94 | 98 | 0 | 104 | 83 | 0 | 0 | 11 | 4 | 1 |
| esophagus | 11 | 7 | 34 | 19 | 32 | 3 | 0 | 44 | 28 | 0 | 0 | 2 | 0 | 0 |
| eye | 31 | 41 | 145 | 52 | 67 | 71 | 1 | 133 | 64 | 0 | 0 | 21 | 4 | 4 |
| heart | 32 | 18 | 44 | 33 | 13 | 20 | 0 | 58 | 32 | 0 | 0 | 8 | 0 | 1 |
| intestine | 88 | 43 | 183 | 53 | 87 | 39 | 0 | 156 | 105 | 0 | 0 | 10 | 2 | 3 |
| kidney | 45 | 38 | 132 | 41 | 50 | 50 | 0 | 85 | 52 | 0 | 1 | 14 | 4 | 8 |
| larynx | 2 | 5 | 4 | 2 | 2 | 0 | 0 | 1 | 0 | 0 | 0 | 0 | 1 | 0 |
| liver | 42 | 37 | 98 | 89 | 93 | 66 | 1 | 91 | 59 | 0 | 0 | 13 | 1 | 0 |
| lung | 61 | 58 | 254 | 94 | 115 | 68 | 1 | 150 | 88 | 0 | 0 | 12 | 5 | 2 |
| lymph | 11 | 6 | 171 | 14 | 21 | 34 | 0 | 71 | 21 | 0 | 0 | 0 | 0 | 0 |
| lymph node | 14 | 15 | 17 | 24 | 14 | 28 | 0 | 9 | 5 | 0 | 0 | 0 | 2 | 0 |
| mammary | 43 | 36 | 84 | 39 | 68 | 17 | 0 | 71 | 25 | 0 | 0 | 6 | 5 | 0 |
| mouth | 4 | 1 | 9 | 4 | 10 | 12 | 0 | 10 | 4 | 0 | 0 | 7 | 2 | 3 |
| muscle | 17 | 9 | 66 | 17 | 17 | 26 | 1 | 78 | 17 | 0 | 0 | 1 | 3 | 2 |
| nerve | 2 | 1 | 3 | 0 | 2 | 1 | 0 | 3 | 3 | 0 | 0 | 1 | 0 | 0 |
| ovary | 19 | 14 | 91 | 36 | 42 | 11 | 1 | 81 | 30 | 0 | 0 | 4 | 0 | 0 |
| pancreas | 26 | 30 | 82 | 25 | 49 | 43 | 6 | 143 | 25 | 0 | 0 | 7 | 1 | 0 |
| parathyroid | 2 | 1 | 4 | 11 | 1 | 7 | 0 | 0 | 0 | 0 | 0 | 3 | 0 | 2 |
| pharynx | 8 | 6 | 45 | 5 | 22 | 3 | 1 | 5 | 23 | 0 | 0 | 4 | 0 | 0 |
| pituitary | 2 | 6 | 5 | 1 | 6 | 3 | 0 | 4 | 7 | 0 | 0 | 1 | 0 | 0 |
| placenta | 61 | 72 | 140 | 82 | 109 | 65 | 0 | 115 | 75 | 1 | 0 | 11 | 1 | 0 |
| prostate | 39 | 28 | 113 | 33 | 50 | 46 | 2 | 49 | 36 | 0 | 0 | 11 | 1 | 0 |
| salivary | 0 | 1 | 6 | 2 | 4 | 2 | 0 | 9 | 8 | 0 | 0 | 1 | 0 | 0 |
| skin | 44 | 57 | 239 | 58 | 86 | 103 | 1 | 164 | 59 | 0 | 0 | 11 | 6 | 3 |
| spleen | 22 | 2 | 23 | 9 | 14 | 2 | 1 | 23 | 19 | 0 | 0 | 6 | 0 | 2 |
| stomach | 22 | 27 | 54 | 25 | 35 | 30 | 4 | 55 | 26 | 0 | 0 | 11 | 1 | 0 |
| testis | 202 | 174 | 283 | 146 | 234 | 51 | 160 | 313 | 177 | 0 | 10 | 25 | 7 | 29 |
| thymus | 60 | 7 | 26 | 18 | 16 | 4 | 0 | 34 | 37 | 0 | 0 | 6 | 5 | 0 |
| thyroid | 15 | 7 | 9 | 9 | 9 | 5 | 0 | 9 | 24 | 0 | 0 | 1 | 0 | 0 |
| tonsil | 6 | 0 | 13 | 2 | 5 | 3 | 0 | 16 | 6 | 0 | 0 | 1 | 0 | 0 |
| trachea | 10 | 6 | 6 | 7 | 5 | 0 | 1 | 16 | 11 | 0 | 0 | 5 | 5 | 3 |
| umbilical | 9 | 8 | 16 | 8 | 8 | 3 | 0 | 11 | 13 | 0 | 0 | 1 | 0 | 0 |
| uterus | 65 | 54 | 119 | 60 | 84 | 87 | 1 | 120 | 87 | 0 | 0 | 21 | 3 | 7 |
| vascular | 16 | 13 | 46 | 14 | 30 | 3 | 2 | 32 | 17 | 0 | 0 | 0 | 0 | 1 |

^1^Number of ESTs reported for each body tissue/site.
