# Supplementary material for: Chaperonin genes on the rise: new divergent classes and intense duplication in human and other vertebrate genomes
Source: BMC Evol Biol. 2010 Mar 1;10:64. doi: 10.1186/1471-2148-10-64 (PMC2846930; doi:10.1186/1471-2148-10-64)
Supplement: Additional file 11 — Figure S7. Evolutionary tree of vertebrate CCT1-8 and CCT8L proteins including associated human pseudogenes. [file 1471-2148-10-64-S11.PDF]

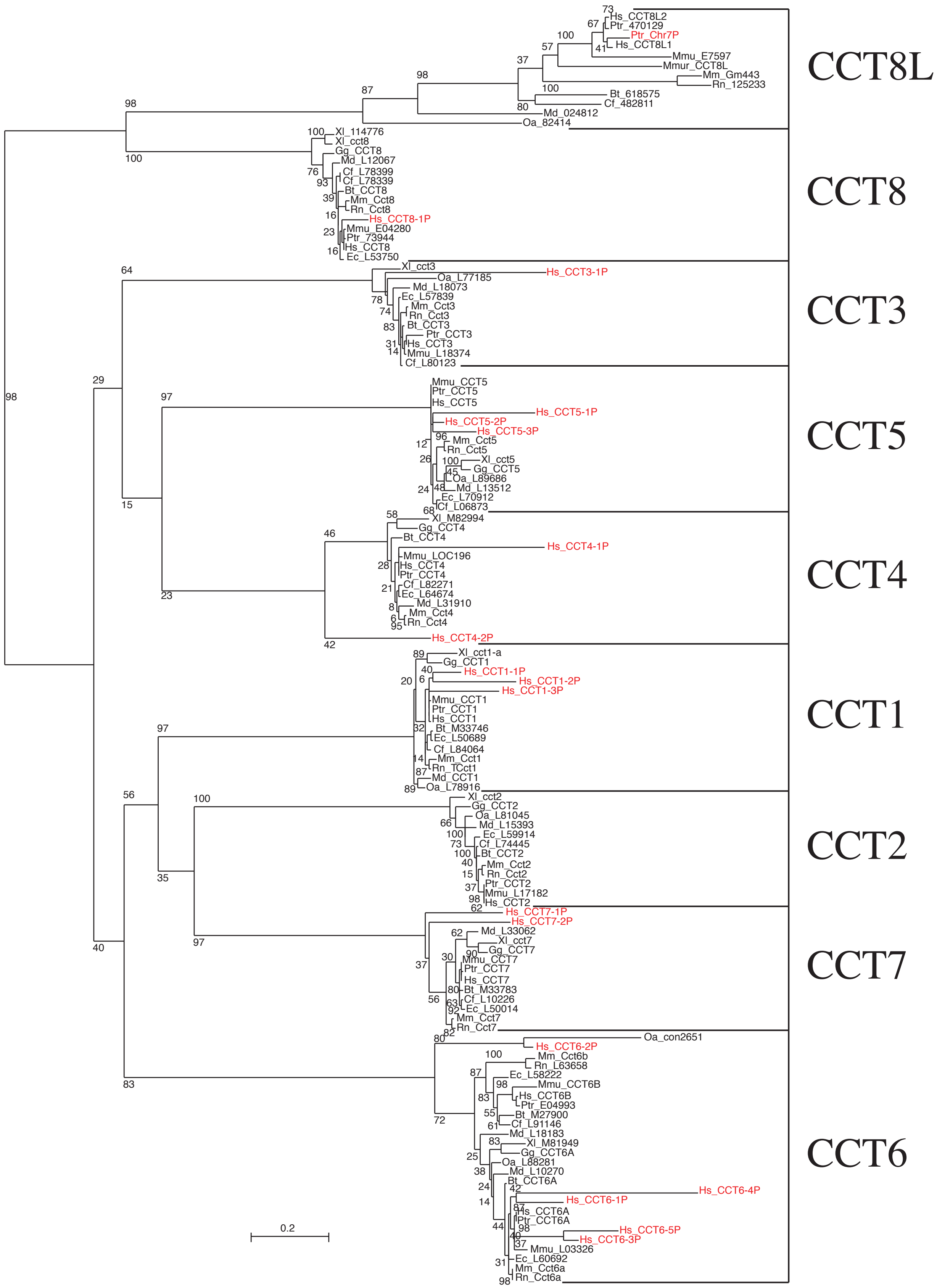

Supplementary figure S7. Summary ML tree of all human pseudogenes (in red font) with CCT and CCT8L from vertebrate species. Pseudogene sequences of CCT8L from chimp and rhesus monkey are also shown in red font. See Legends for Figure S5 and for Figure 2 for species. abbreviations. The scale bar represents the indicated number of substitutions per position for a unit branch length.
