# Supplementary material for: Chaperonin genes on the rise: new divergent classes and intense duplication in human and other vertebrate genomes
Source: BMC Evol Biol. 2010 Mar 1;10:64. doi: 10.1186/1471-2148-10-64 (PMC2846930; doi:10.1186/1471-2148-10-64)
Supplement: Additional file 12 — Figure S8. Evolutionary trees of individual CCT1, CCT3 and CCT4 proteins from vertebrates including associated human pseudogenes. [file 1471-2148-10-64-S12.PDF]

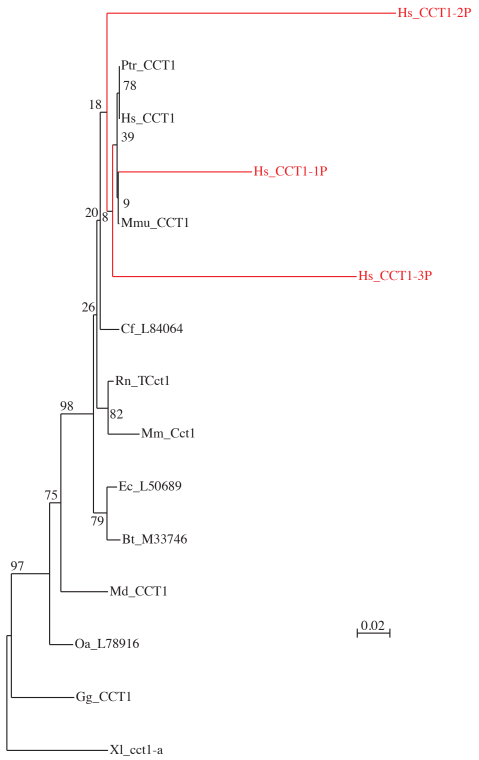

Human CCT1 pseudogenes

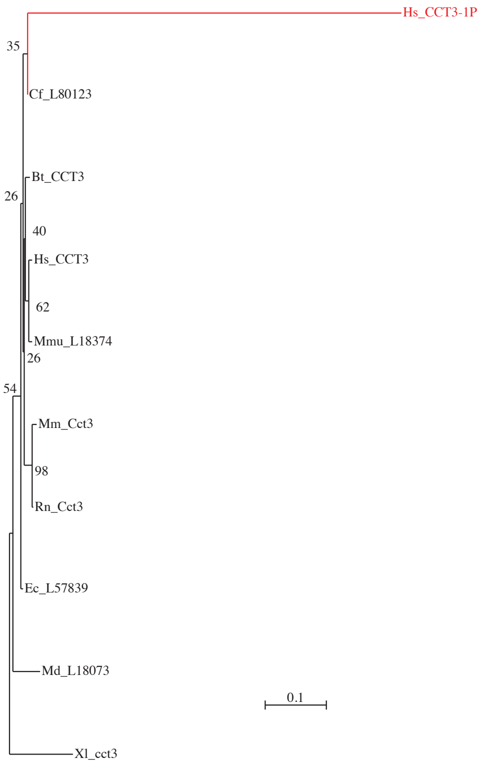

Human CCT3 pseudogenes

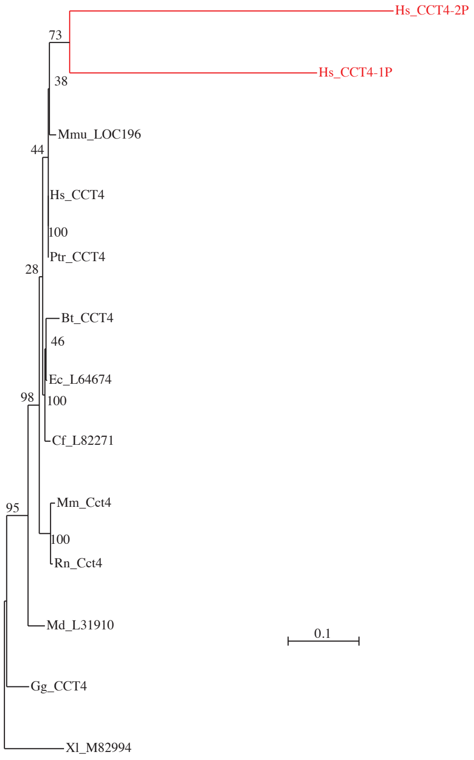

Human CCT4 pseudogenes

Supplementary figure S8. ML trees of individual CCT monomer families including human pseudogenes (in red font). See Legends for Figure S5 and for Figure 2 for species abbreviations. The scale bar represents the indicated number of substitutions per position for a unit branch length.
