# Supplementary material for: Chaperonin genes on the rise: new divergent classes and intense duplication in human and other vertebrate genomes
Source: BMC Evol Biol. 2010 Mar 1;10:64. doi: 10.1186/1471-2148-10-64 (PMC2846930; doi:10.1186/1471-2148-10-64)
Supplement: Additional file 13 — Figure S9. Evolutionary trees of individual CCT5, CCT7 and CCT8 proteins from vertebrates including associated human pseudogenes. [file 1471-2148-10-64-S13.PDF]

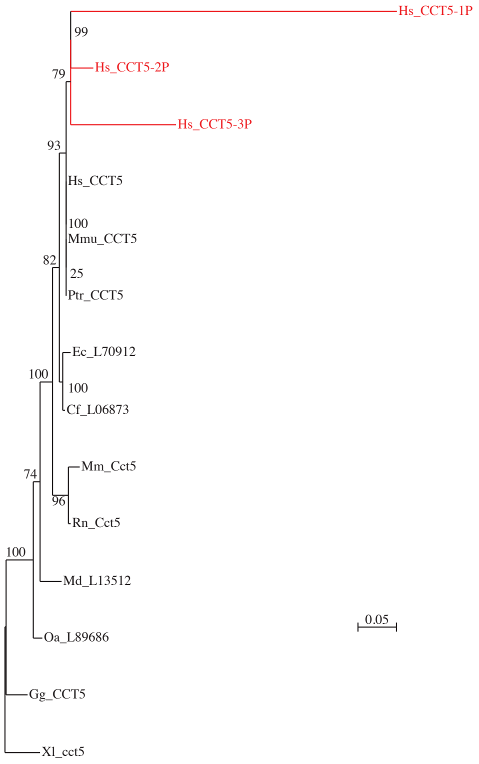

Human CCT5 pseudogenes

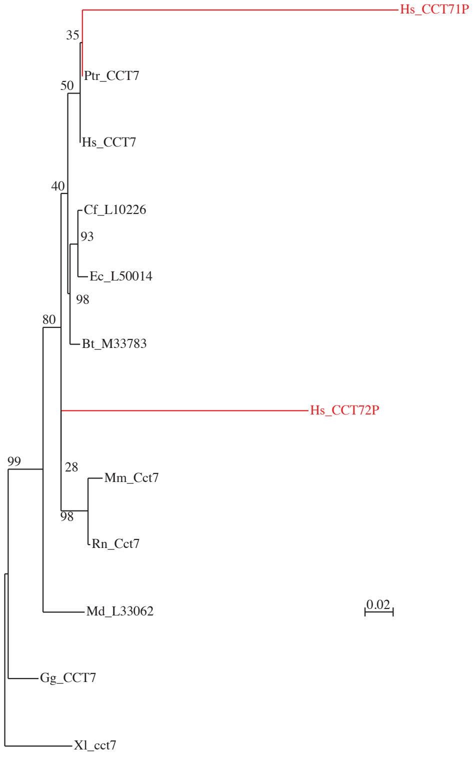

Human CCT7 pseudogenes

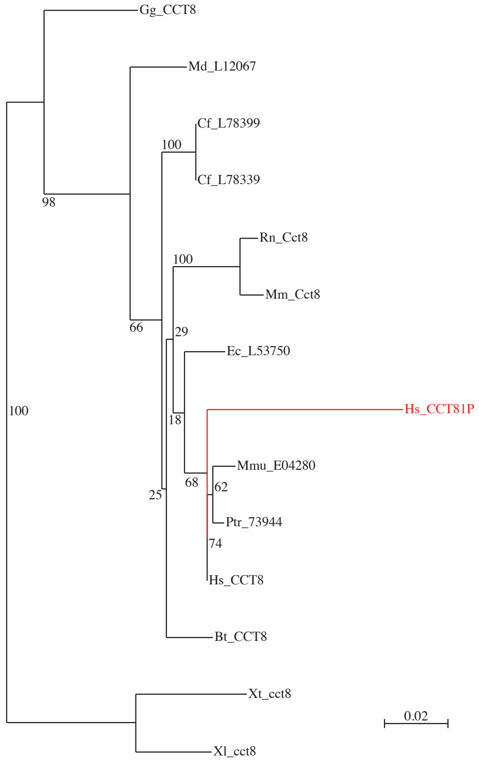

Human CCT8 pseudogenes

Supplementary figure S9. ML trees of individual CCT monomer families including human pseudogenes (in red font). See Legends for Figure S5 and for Figure 2 for species abbreviations. The scale bar represents the indicated number of substitutions per position for a unit branch length.
