# Supplementary material for: Chaperonin genes on the rise: new divergent classes and intense duplication in human and other vertebrate genomes
Source: BMC Evol Biol. 2010 Mar 1;10:64. doi: 10.1186/1471-2148-10-64 (PMC2846930; doi:10.1186/1471-2148-10-64)
Supplement: Additional file 14 — Table S5. Expression pattern of the human cpn60 gene (HSPD1) and pseudogenes from the UniGene database. [file 1471-2148-10-64-S14.DOC]

Table S5. Expression pattern of the human *cpn60* gene (HSPD1) and pseudogenes^1^

| **Body tissue/site** | **HSPD1** | **HSPD1-5P (LOC644745)** | **HSPD1-6P (LOC645548)** |
| --- | --- | --- | --- |
| adipose | 6 | 0 | 0 |
| adrenal | 96 | 0 | 0 |
| ascites | 31 | 1 | 0 |
| bladder | 21 | 0 | 1 |
| blood | 90 | 0 | 0 |
| bone | 14 | 0 | 0 |
| bone marrow | 37 | 1 | 0 |
| brain | 537 | 1 | 0 |
| cervix | 24 | 2 | 0 |
| connective | 32 | 0 | 0 |
| ear | 4 | 0 | 0 |
| embryonic | 101 | 1 | 1 |
| esophagus | 22 | 0 | 0 |
| eye | 45 | 1 | 0 |
| heart | 33 | 0 | 0 |
| intestine | 107 | 0 | 2 |
| kidney | 146 | 0 | 0 |
| larynx | 4 | 0 | 0 |
| liver | 143 | 1 | 0 |
| lung | 96 | 0 | 0 |
| lymph | 30 | 0 | 0 |
| lymph node | 4 | 0 | 0 |
| mammary | 35 | 1 | 0 |
| muscle | 18 | 0 | 0 |
| nerve | 43 | 0 | 0 |

Table S5 (continued)

| **Body tissue/site** | **HSPD1** | **HSPD1-5P (LOC644745)** | **HSPD1-6P (LOC645548)** |
| --- | --- | --- | --- |
| ovary | 3 | 0 | 0 |
| pancreas | 27 | 0 | 0 |
| parathyroid | 24 | 0 | 0 |
| pharynx | 1 | 0 | 0 |
| pituitary | 12 | 0 | 0 |
| placenta | 3 | 0 | 0 |
| prostate | 61 | 0 | 0 |
| salivary | 50 | 10 | 0 |
| skin | 4 | 0 | 0 |
| soft | 75 | 0 | 0 |
| spleen | 21 | 0 | 0 |
| stomach | 31 | 0 | 0 |
| testis | 135 | 0 | 0 |
| thymus | 38 | 0 | 0 |
| thyroid | 17 | 0 | 1 |
| tongue | 0 | 0 | 0 |
| tonsil | 19 | 0 | 0 |
| trachea | 6 | 0 | 0 |
| umbilical cord | 78 | 2 | 0 |
| uterus | 43 | 0 | 0 |
| vascular | 6 | 0 | 0 |

^1^Number of ESTs reported in each body tissue/site.
