# Supplementary material for: Chaperonin genes on the rise: new divergent classes and intense duplication in human and other vertebrate genomes
Source: BMC Evol Biol. 2010 Mar 1;10:64. doi: 10.1186/1471-2148-10-64 (PMC2846930; doi:10.1186/1471-2148-10-64)
Supplement: Additional file 15 — Figure S10. Alignment and secondary-structure prediction of archaeal thermosome sequences. [file 1471-2148-10-64-S15.PDF]

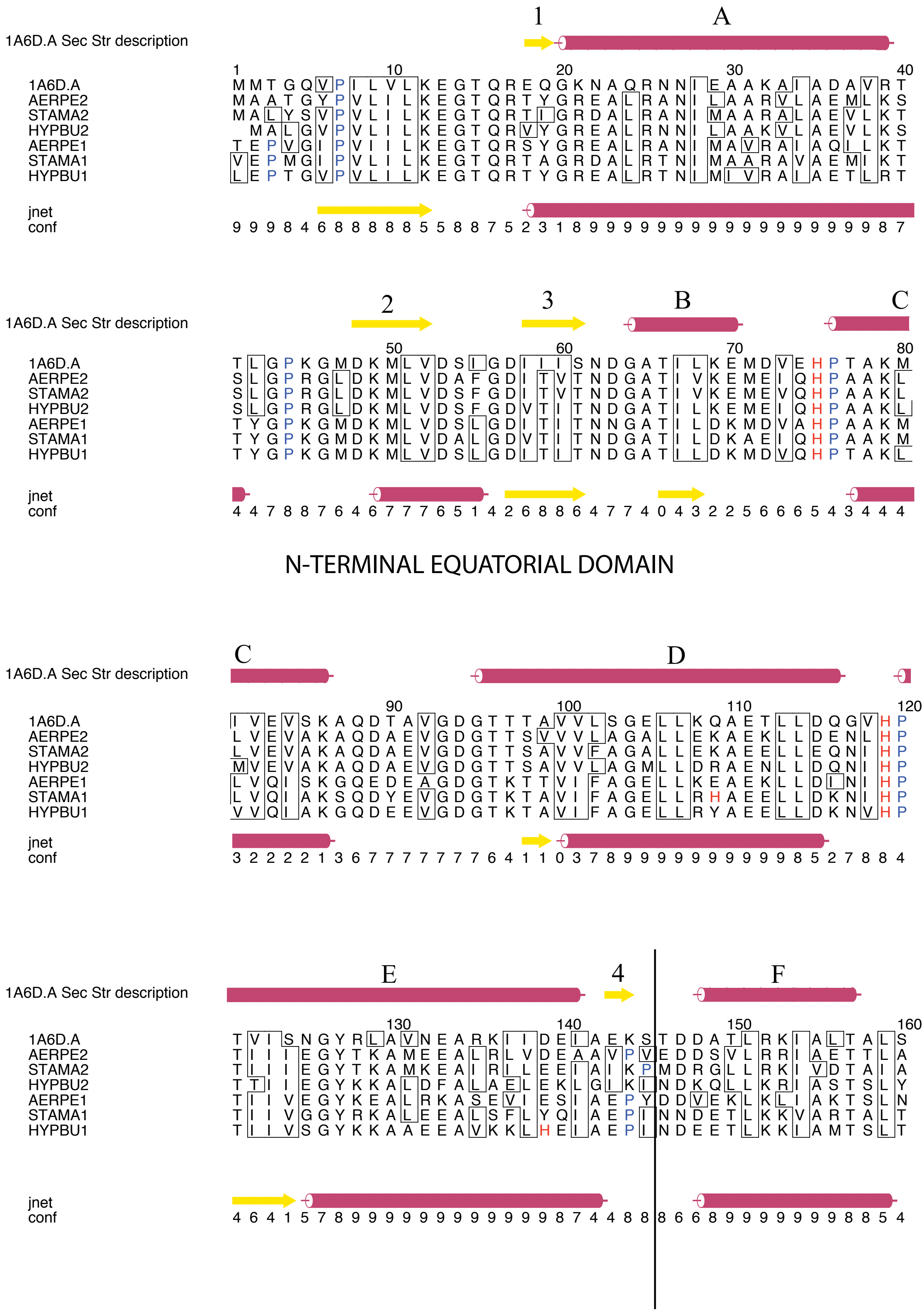
1 A
1A6D.A Sec Str description
1 10 20 30 40
1A6D.A MMTGQVPILVLKEGTQREQGKNAQRNNIEAAKAIADAVRT
AERPE2 MAATGYPVLILKEGTQRTYGREALRANILAARVLAEMLKS
STAMA2 MALYSVPVLILKEGTQRTIGRDALRANIMAARALAEVLKT
HYPBU2 MALGVPVLILKEGTQRVYGREALRNNILAAKVLAEVLKS
AERPE1 TEPVGIPVIILKEGTQRSYGREALRANIMAVRAIAQILKT
STAMA1 VEPMGIPVLILKEGTQRTAGRDALRTNIMAARAVAEMIKT
HYPBU1 LEPTGVPVLILKEGTQRTYGREALRTNIMIVRAIAETLRT
jnet
conf 9 9 9 8 4 6 8 8 8 8 5 5 8 8 7 5 2 3 1 8 9 9 9 9 9 9 9 9 9 9 9 9 9 9 9 9 9 9 8 7
2 3 B C
1A6D.A Sec Str description
50 60 70 80
1A6D.A TLGPKGMDKMLVDSIGDIIISNDGATILKEMDVEHPTAKM
AERPE2 SLGPRGLDKMLVDAFGDITVTNDGATIVKEMEIQHPAAKL
STAMA2 SLGPRGLDKMLVDSFGDITVTNDGATIVKEMEVQHPAAKL
HYPBU2 SLGPRGLDKMLVDSFGDVTITNDGATILKEMEIQHPAAKL
AERPE1 TYGPKGMDKMLVDSLGDITITNNGATILDKMDVAHPAAKM
STAMA1 TYGPKGMDKMLVDALGDVTITNDGATILDKAEIQHPAAKM
HYPBU1 TYGPKGMDKMLVDSLGDITITNDGATILDKMDVQHPTAKL
jnet
conf 4 4 7 8 8 7 6 4 6 7 7 7 6 5 1 4 2 6 8 8 6 4 7 7 4 0 4 3 2 2 5 6 6 6 5 4 3 4 4 4
N-TERMINAL EQUATORIAL DOMAIN
C D
1A6D.A Sec Str description
90 100 110 120
1A6D.A IVEVSKAQDTAVGDGTTTAVVLSGELLKQAETLLDQGVHP
AERPE2 LVEVAKAQDAEVGDGTTSVVVLAGALLEKAEKLLDENLHP
STAMA2 LVEVAKAQDAEVGDGTTSAVVFAGALLEKAEELLEQNIHP
HYPBU2 MVEVAKAQDAEVGDGTTSAVVLAGMLLDRAENLLDQNIHP
AERPE1 LVQISKGQEDEAGDGTKTTVIFAGELLKEAEKLLDINIHP
STAMA1 LVQIAKSQDYEVGDGTKTAVIFAGELLRHAEELLDKNIHP
HYPBU1 VVQIAKGQDEEVGDGTKTAVIFAGELLRYAEELLDKNVHP
jnet
conf 3 2 2 2 2 1 3 6 7 7 7 7 7 7 7 6 4 1 1 0 3 7 8 9 9 9 9 9 9 9 9 9 9 8 5 2 7 8 8 4
E 4 F
1A6D.A Sec Str description
130 140 150 160
1A6D.A TVISNGYRLAVNEARKIIDEIAEKSTDDATLRKIALTALS
AERPE2 TIIIEGYTKAMEEALRLVDEAAVPVEDDSVLRRIAETTLA
STAMA2 TIIIEGYTKAMKEAIRILEEIAIKPMDRGLLRKIVDTAIA
HYPBU2 TTIIEGYKKALDFALAELEKLGIKINDKQLLKRIASTSLY
AERPE1 TIIVEGYKEALRKASEVIESIAEPYDDVEKLKLIAKTSLN
STAMA1 TIIVGGYRKALEEALSFLYQIAEPINNDETLKKVARTALT
HYPBU1 TIIVSGYKKAAEEAVKKLHEIAEPINDEETLKKIAMTSLT
jnet
conf 4 6 4 1 5 7 8 9 9 9 9 9 9 9 9 9 9 9 9 8 7 4 4 8 8 8 6 6 8 9 9 9 9 9 9 9 8 8 5 4

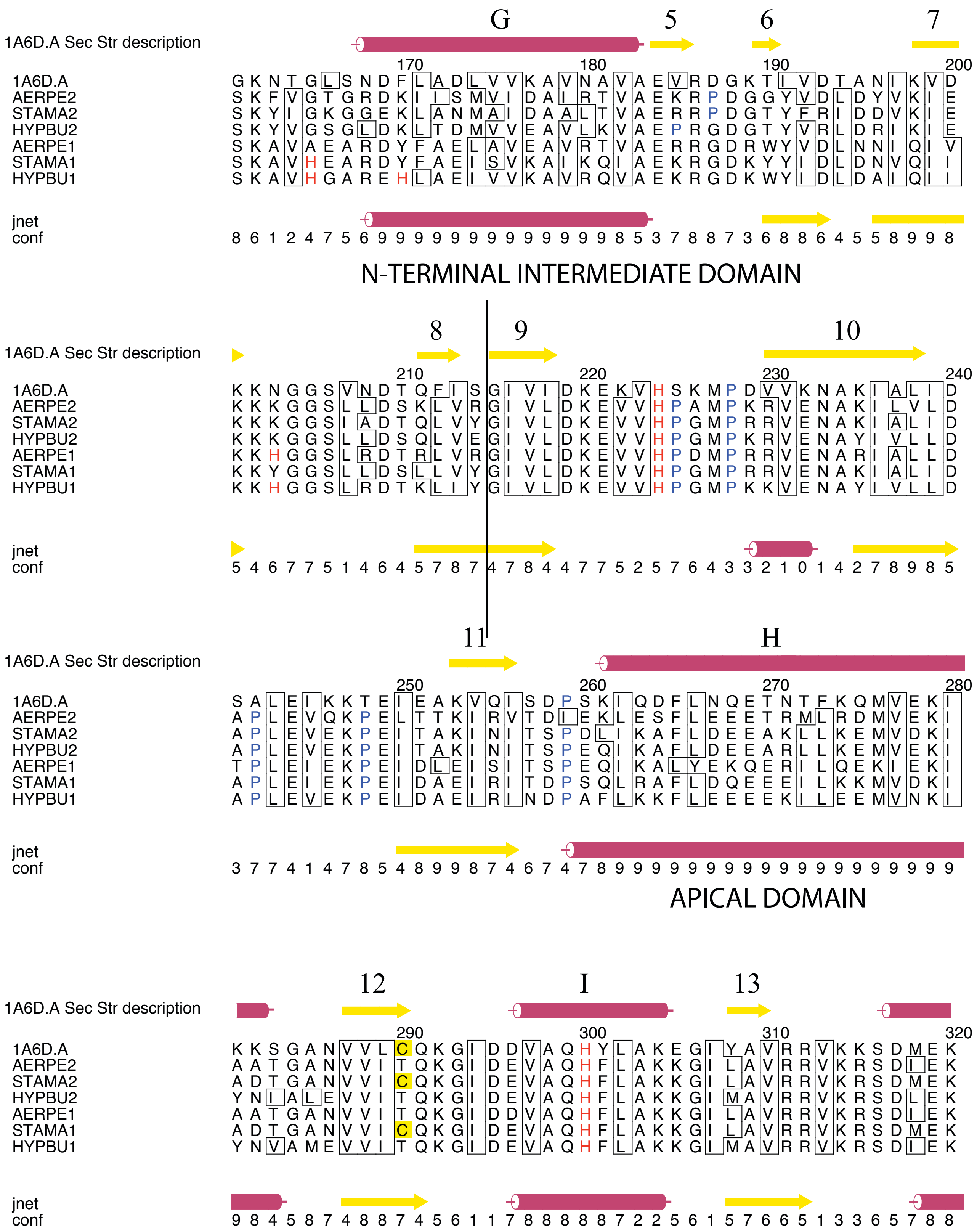
G 5 6 7
1A6D.A Sec Str description
170 180 190 200
1A6D.A G K N T G L S N D F L A D L V V K A V N A V A E V R D G K T I V D T A N I K V D
AERPE2 S K F V G T G R D K I I S M V I D A I R T V A E K R P D G G Y V D L D Y V K I E
STAMA2 S K Y I G K G G E K L A N M A I D A A L T V A E R R P D G T Y F R I D D V K I E
HYPBU2 S K Y V G S G L D K L T D M V V E A V L K V A E P R G D G T Y V R L D R I K I E
AERPE1 S K A V A E A R D Y F A E L A V E A V R T V A E R R G D R W Y V D L N N I Q I V
STAMA1 S K A V H E A R D Y F A E I S V K A I K Q I A E K R G D K Y Y I D L D N V Q I I
HYPBU1 S K A V H G A R E H L A E I V V K A V R Q V A E K R G D K W Y I D L D A I Q I I
jnet
conf 8 6 1 2 4 7 5 6 9 9 9 9 9 9 9 9 9 9 9 9 9 8 5 3 7 8 8 7 3 6 8 8 6 4 5 5 8 9 9 8
N-TERMINAL INTERMEDIATE DOMAIN
8 9 10
1A6D.A Sec Str description
210 220 230 240
1A6D.A K K N G G S V N D T Q F I S G I V I D K E K V H S K M P D V V K N A K I A L I D
AERPE2 K K K G G S L L D S K L V R G I V L D K E V V H P A M P K R V E N A K I L V L D
STAMA2 K K K G G S I A D T Q L V Y G I V L D K E V V H P G M P R R V E N A K I A L I D
HYPBU2 K K K G G S L L D S Q L V E G I V L D K E Q V H P G M P K R V E N A Y I V L L D
AERPE1 K K H G G S L R D T R L V R G I V L D K E V V H P D M P R R V E N A R I A L L D
STAMA1 K K Y G G S L L D S L L V Y G I V L D K E V V H P G M P R R V E N A K I A L I D
HYPBU1 K K H G G S L R D T K L I Y G I V L D K E V V H P G M P K K V E N A Y I V L L D
jnet
conf 5 4 6 7 7 5 1 4 6 4 5 7 8 7 4 7 8 4 4 7 7 5 2 5 7 6 4 3 3 2 1 0 1 4 2 7 8 9 8 5
11 H
1A6D.A Sec Str description
250 260 270 280
1A6D.A S A L E I K K T E I E A K V Q I S D P S K I Q D F L N Q E T N T F K Q M V E K I
AERPE2 A P L E V Q K P E L T T K I R V T D I E K L E S F L E E E T R M L R D M V E K I
STAMA2 A P L E V E K P E I T A K I N I T S P D L I K A F L D E E A K L L K E M V D K I
HYPBU2 A P L E V E K P E I T A K I N I T S P E Q I K A F L D E E A R L L K E M V E K I
AERPE1 T P L E I E K P E I D L E I S I T S P E Q I K A L Y E K Q E R I L Q E K I E K I
STAMA1 A P L E I E K P E I D A E I R I T D P S Q L R A F L D Q E E E I L K K M V D K I
HYPBU1 A P L E V E K P E I D A E I R I N D P A F L K K F L E E E E K I L E E M V N K I
jnet
conf 3 7 7 4 1 4 7 8 5 4 8 9 9 8 7 4 6 7 4 7 8 9 9 9 9 9 9 9 9 9 9 9 9 9 9 9 9 9 9 9
APICAL DOMAIN
12 I 13
1A6D.A Sec Str description
290 300 310 320
1A6D.A K K S G A N V V L C Q K G I D D V A Q H Y L A K E G I Y A V R R V K K S D M E K
AERPE2 A A T G A N V V I T Q K G I D E V A Q H F L A K K G I L A V R R V K R S D I E K
STAMA2 A D T G A N V V I C Q K G I D E V A Q H F L A K K G I L A V R R V K R S D M E K
HYPBU2 Y N I A L E V V I T Q K G I D E V A Q H F L A K K G I M A V R R V K R S D L E K
AERPE1 A A T G A N V V I T Q K G I D D V A Q H F L A K K G I L A V R R V K R S D I E K
STAMA1 A D T G A N V V I C Q K G I D E V A Q H F L A K K G I L A V R R V K R S D M E K
HYPBU1 Y N V A M E V V I T Q K G I D E V A Q H F L A K K G I M A V R R V K R S D I E K
jnet
conf 9 8 4 5 8 7 4 8 8 7 4 5 6 1 1 7 8 8 8 8 8 7 2 2 5 6 1 5 7 6 6 5 1 3 3 6 5 7 8 8

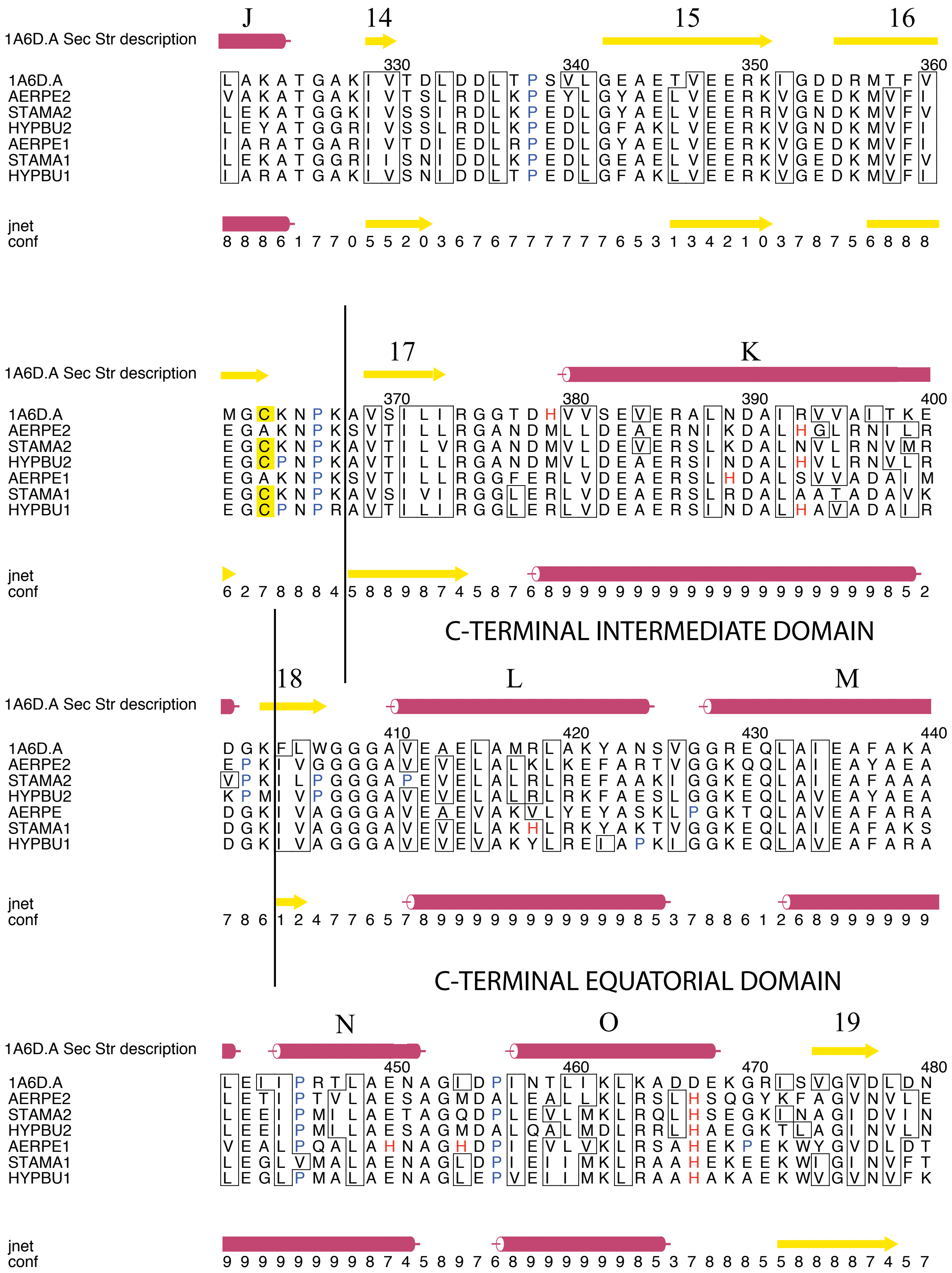
J 14 15 16
1A6D.A Sec Str description
330 340 350 360
1A6D.A LAKATGAKIVTDLDDLTPSVLGEAETVEERKIGDDRMTFV
AERPE2 VAKATGAKIVTSLRDLKPEYLGYAELVEERKVGEDKMVFI
STAMA2 LEKATGGKIVSSIRDLKPEDLGYAELVEERRVGNDKMVFV
HYPBU2 LEYATGGRIVSSLRDLKPEDLGFAKLVEERKVGNDKMVFI
AERPE1 IARATGARIVTDIEDLRPEDLGYAELVEERKVGEDKMVFI
STAMA1 LEKATGGRIISNIDDLKPEDLGEAELVEERKVGEDKMVFV
HYPBU1 IARATGAKIVSNIDDLTPEDLGFAKLVEERKVGEDKMVFI
jnet
conf 8 8 8 6 1 7 7 0 5 5 2 0 3 6 7 6 7 7 7 7 7 7 6 5 3 1 3 4 2 1 0 3 7 8 7 5 6 8 8 8
17 K
1A6D.A Sec Str description
370 380 390 400
1A6D.A MGCKNPKAVSILIRGGTDHVVSEVERALNDAIRVVAITKE
AERPE2 EGAKNPKSVTILLRGANDMLLDEAERNIKDALHGLRNILR
STAMA2 EGCKNPKAVTILVRGANDMVLDEVERSLKDALNVLRNVMR
HYPBU2 EGCPNPKAVTILLRGANDMVLDEAERSINDALHVLRNVLR
AERPE1 EGAKNPKSVTILLRGGFERLVDEAERSLHDALSVVADAIM
STAMA1 EGCKNPKAVSIVIRGGLERLVDEAERSLRDALAATADAVK
HYPBU1 EGCPNPRAVTILIRGGLERLVDEAERSINDALHAVADAIR
jnet
conf 6 2 7 8 8 8 4 5 8 8 9 8 7 4 5 8 7 6 8 9 9 9 9 9 9 9 9 9 9 9 9 9 9 9 9 9 9 8 5 2
C-TERMINAL INTERMEDIATE DOMAIN
18 L M
1A6D.A Sec Str description
410 420 430 440
1A6D.A DGKFLWGGGAVEAELAMRLAKYANSVGGREQLAIEAFAKA
AERPE2 EPKIVGGGGAVEVELALKLKEFARTVGGKQQLAIEAYAEA
STAMA2 VPKILPGGGAPEVELALRLREFAAKIGGKEQLAIEAFAAA
HYPBU2 KPMIVPGGGAVEVELALRLRKFAESLGGKEQLAVEAYAEA
AERPE DGKIVAGGGAVEAEVAKVLYEYASKLPGKTQLAVEAFARA
STAMA1 DGKIVAGGGAVEVELAKHLRKYAKTVGGKEQLAIEAFAKS
HYPBU1 DGKIVAGGGAVEVEVAKYLREIAPKIGGKEQLAVEAFARA
jnet
conf 7 8 6 1 2 4 7 7 6 5 7 8 9 9 9 9 9 9 9 9 9 9 8 5 3 7 8 8 6 1 2 6 8 9 9 9 9 9 9 9
C-TERMINAL EQUATORIAL DOMAIN
N O 19
1A6D.A Sec Str description
450 460 470 480
1A6D.A LEIIPRTLAENAGIDPINTLIKLKADDEKGRISVGVDLDN
AERPE2 LETIPTVLAESAGMDALEALLKLRSLHSQGYKFAGVNVLE
STAMA2 LEEIPMILAETAGQDPLEVLMKLRQLHSEGKINAGIDVIN
HYPBU2 LEEIPMILAESAGMDALQALMDLRRLHAEGKTLAGINVLN
AERPE1 VEALPQALAHNAGHDPIEVLVKLRSAHEKPEKWYGVDLDT
STAMA1 LEGLVMALAENAGLDPIEIIMKLRAAHEKEEKWIGINVFT
HYPBU1 LEGLPMALAENAGLEPVEIIMKLRAAHAKAEKWVGVNVFK
jnet
conf 9 9 9 9 9 9 9 9 8 7 4 5 8 9 7 6 8 9 9 9 9 9 9 8 5 3 7 8 8 8 5 5 8 8 8 8 7 4 5 7

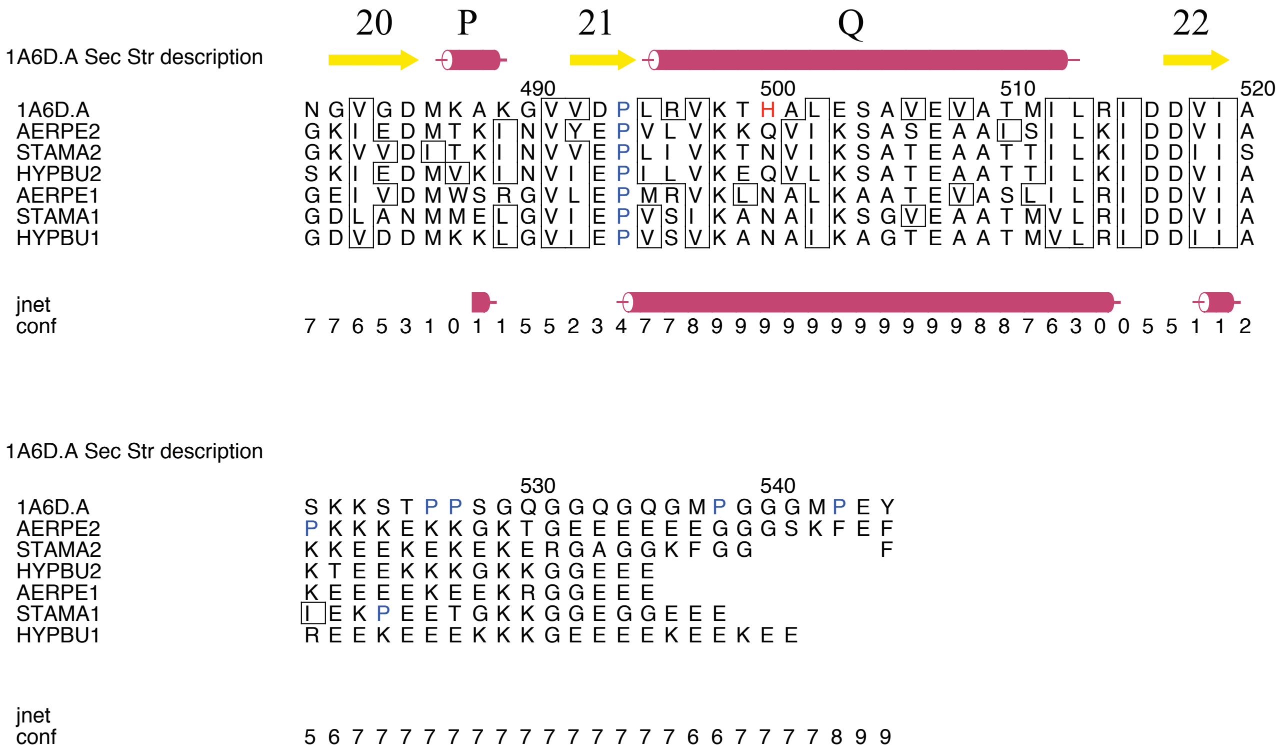

Supplementary figure S10. Secondary-structure predictions (line "jnet") from the alignment of archaeal thermosome-subunit sequences, aligned to the secondary-structure description (line "1A6D.A Sec Str description") of the thermosome alpha-subunit of *Thermoplasma acidophilum* (sequence 1A6D.A), as provided in the PDB entry 1a6d, chain A. Included in the alignment are also each of the two thermosome-subunit sequences from the Crenarchaeota *Aeropyrum pernix* (AERPE), *Staphylothermus marinus* (STAMA) and *Hyperthermus butylicus* (HYPBU). Red cylinders represent alpha helices. Beta strands are represented as yellow arrows. Helices described in the thermosome PDB structure are labelled alphabetically and strands are labelled numerically, following the assignents shown in Figure 6. Vertical lines indicate boundaries between tertiary-structure domains (apical, intermediate and equatorial). Predictions obtained using JPRED-3. Histidine residues are colored in red, proline residues in blue, cysteine residues in yellow and aliphatic residues I, L and V are boxed in the JPRED-3 output. Position-specific confidence in the secondary structure assignments is scored in line "conf" from 0 (lowest confidence) to 9 (highest confidence). A score S indicates a posterior probability (p) range such that $S/10 < p \leq S/10+1$.
