# Supplementary material for: Chaperonin genes on the rise: new divergent classes and intense duplication in human and other vertebrate genomes
Source: BMC Evol Biol. 2010 Mar 1;10:64. doi: 10.1186/1471-2148-10-64 (PMC2846930; doi:10.1186/1471-2148-10-64)
Supplement: Additional file 16 — Table S11. Alignment and secondary-structure prediction of human CCT1-8 protein sequences. [file 1471-2148-10-64-S16.PDF]

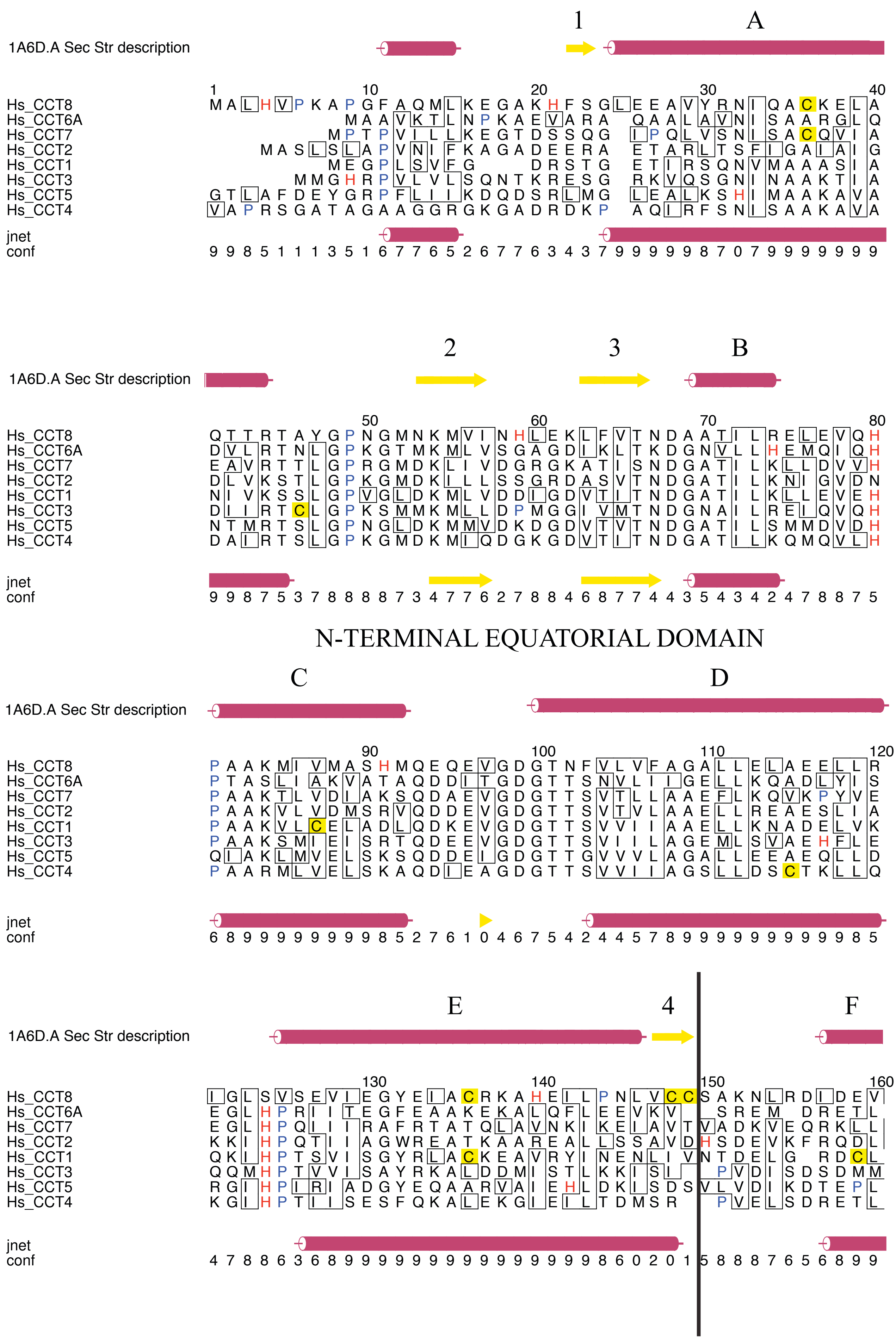
N-TERMINAL EQUATORIAL DOMAIN
1A6D.A Sec Str description
1 A
1 10 20 30 40
Hs_CCT8 M A L H V P K A P G F A Q M L K E G A K H F S G L E E A V Y R N I Q A C K E L A
Hs_CCT6A M A A V K T L N P K A E V A R A Q A A L A V N I S A A R G L Q
Hs_CCT7 M P T P V I L L K E G T D S S Q G I P Q L V S N I S A C Q V I A
Hs_CCT2 M A S L S L A P V N I F K A G A D E E R A E T A R L T S F I G A I A I G
Hs_CCT1 M E G P L S V F G D R S T G E T I R S Q N V M A A A S I A
Hs_CCT3 M M G H R P V L V L S Q N T K R E S G R K V Q S G N I N A A K T I A
Hs_CCT5 G T L A F D E Y G R P F L I I K D Q D S R L M G L E A L K S H I M A A K A V A
Hs_CCT4 V A P R S G A T A G A A G G R G K G A D R D K P A Q I R F S N I S A A K A V A
jnet
conf 9 9 8 5 1 1 1 3 5 1 6 7 7 6 5 2 6 7 7 6 3 4 3 7 9 9 9 9 9 8 7 0 7 9 9 9 9 9 9 9
1A6D.A Sec Str description
2 3 B
50 60 70 80
Hs_CCT8 Q T T R T A Y G P N G M N K M V I N H L E K L F V T N D A A T I L R E L E V Q H
Hs_CCT6A D V L R T N L G P K G T M K M L V S G A G D I K L T K D G N V L L H E M Q I Q H
Hs_CCT7 E A V R T T L G P R G M D K L I V D G R G K A T I S N D G A T I L K L L D V V H
Hs_CCT2 D L V K S T L G P K G M D K I L L S S G R D A S V T N D G A T I L K N I G V D N
Hs_CCT1 N I V K S S L G P V G L D K M L V D D I G D V T I T N D G A T I L K L L E V E H
Hs_CCT3 D I I R T C L G P K S M M K M L L D P M G G I V M T N D G N A I L R E I Q V Q H
Hs_CCT5 N T M R T S L G P N G L D K M M V D K D G D V T V T N D G A T I L S M M D V D H
Hs_CCT4 D A I R T S L G P K G M D K M I Q D D G K G D V T I T N D G A T I L K Q M Q V L H
jnet
conf 9 9 8 7 5 3 7 8 8 8 8 7 3 4 7 7 6 2 7 8 8 4 6 8 7 7 4 4 3 5 4 3 4 2 4 7 8 8 7 5
C D
1A6D.A Sec Str description
90 100 110 120
Hs_CCT8 P A A K M I V M A S H M Q E Q E V G D G T N F V L V F A G A L L E L A E E L L R
Hs_CCT6A P T A S L I A K V A T A Q D D I T G D G T T S N V L I I G E L L K Q A D L Y I S
Hs_CCT7 P A A K T L V D I A K S Q D A E V G D G T T S V T L L A A E F L K Q V K P Y V E
Hs_CCT2 P A A K V L V D M S R V Q D D E V G D G T T S V T V L A A E L L R E A E S L I A
Hs_CCT1 P A A K V L C E L A D L Q D K E V G D G T T S V V I I A A E L L K N A D E L V K
Hs_CCT3 P A A K S M I E I S R T Q D E E V G D G T T S V I I L A G E M L S V A E H F L E
Hs_CCT5 Q I A K L M V E L S K S Q D D E I G D G T T G V V V L A G A L L E E A E Q L L D
Hs_CCT4 P A A R M L V E L S K A Q D I E A G D G T T S V V I I A G S L L D S C T K L L Q
jnet
conf 6 8 9 9 9 9 9 9 9 9 8 5 2 7 6 1 0 4 6 7 5 4 2 4 4 5 7 8 9 9 9 9 9 9 9 9 9 9 8 5
E 4 F
1A6D.A Sec Str description
130 140 150 160
Hs_CCT8 I G L S V S E V I E G Y E I A C R K A H E I L P N L V C C S A K N L R D I D E V
Hs_CCT6A E G L H P R I I T E G F E A A K E K A L Q F L E E V K V S R E M D R E T L
Hs_CCT7 E G L H P Q I I I R A F R T A T Q L A V N K I K E I A V T V A D K V E Q R K L L
Hs_CCT2 K K I H P Q T I I A G W R E A T K A A R E A L L S S A V D H S D E V K F R Q D L
Hs_CCT1 Q K I H P T S V I S G Y R L A C K E A V R Y I N E N L I V N T D E L G R D C L
Hs_CCT3 Q Q M H P T V V I S A Y R K A L D D M I S T L K K I S I P V D I S D S D M M
Hs_CCT5 R G I H P I R I A D G Y E Q A A R V A I E H L D K I S D S V L V D I K D T E P L
Hs_CCT4 K G I H P T I I S E S F Q K A L E K G I E I L T D M S R P V E L S D R E T L
jnet
conf 4 7 8 8 6 3 6 8 9 9 9 9 9 9 9 9 9 9 9 9 9 9 9 8 6 0 2 0 1 5 8 8 8 7 6 5 6 8 9 9

```
                              G                5    6
1A6D.A Sec Str description

                      170                     180                     190                     200
Hs_CCT8     SSLLRTSIMSKQYGNEVFLAKLIAQACVSIFPDSGHFNVD
Hs_CCT6A    IDVARTSLRTKVHALADVLTEAVVDSILAIKKQDEPIDLF
Hs_CCT7     EKCAMTALSSKLISQKAFFAKMVVDAVMMLDDLL---QLK
Hs_CCT2     MNIAGTTLSSKLLTHKDHFTKLAVEAVLRLKGSG---NLE
Hs_CCT1     INAAKTSMSSKIIGNGDFFANMVVDAVLAIKYTDPRYPVN
Hs_CCT3     LNIINSSITTKAISWSSLACNIALDAVKMVQFEEKEIDIK
Hs_CCT5     IQTAKTTLGSKVVNCHRQMAEIAVNAVLTVADMERDVDFE
Hs_CCT4     LNSATTSLNSKVVSYSSLLSPMSVNAVMKVIDPATSVDLR

jnet
conf        9 9 9 9 9 9 9 9 9 9 9 9 9 9 9 9 9 9 9 9 9 9 9 9 9 9 8 8 6 2 5 7 7 7 6 5 1 4 7 5
```

N-TERMINAL INTERMEDIATE DOMAIN

```
            7                   8    9                       10
1A6D.A Sec Str description

                      210                     220                     230                     240
Hs_CCT8     NIRVCKILGSGISSSSVLHGMVFKKETEGDVTSVKDAKIA
Hs_CCT6A    MIEIMEMKHKSETDTSLIRGLVLDHGAPDMKKRVEDAYIL
Hs_CCT7     MIGIKKVQGGALEDSQLVAGVAFKKTFAGQPKKYHNPKIA
Hs_CCT2     AIHIIKKLGGSLADSYLDEGFLLDKKINQ-PKRIENAKIL
Hs_CCT1     SVNILKAHGRSQMESMLISGYALNCVVQGMPKRIVNAKIA
Hs_CCT3     KARVEKIPGGIIEDSCVLRGVMINKDVPRMRRYIKNPRIV
Hs_CCT5     LIKVEGKVGGRLEDTKLIKGVIVDKDFPQMPKKVEDAKIA
Hs_CCT4     DIKIVKKLGGTIDDCELVEGLVLTQKVSG-ITRVEKAKIG

jnet
conf        5 8 8 9 9 8 8 5 4 7 8 8 8 7 5 5 8 9 9 9 9 8 7 1 2 0 0 4 7 6 3 2 1 2 3 6 6 2 6 8
```

```
                                  11                   H
1A6D.A Sec Str description

                      250                     260                     270                     280
Hs_CCT8     VYSCPFDGMITETKGTVLIKTAEELMNFSKGEENLMDAQV
Hs_CCT6A    TCNVSLEYEKTEVNSGFFYKSAEEREKLVKAERKFIEDRV
Hs_CCT7     LLNVELELKAEKDNAEIRVHTVEDYQAIVDAEWNILYDKL
Hs_CCT2     IANTGMDTDKIKIGSRVRVDSTAKVAEIEHAEKEKMKEKV
Hs_CCT1     CLDFSLQKTKMKLGVQVVITDPEKLDQIRQRESDITKERI
Hs_CCT3     LLDSSLEYKKGESQTDIEITREEDFTRILQMEEEYIQQLC
Hs_CCT5     ILTCPFEPPKPKTKHKLDVTSVEDYKALQKYEKEKFEEMI
Hs_CCT4     LIQFCLSAPKTDMDNQIVVSDYAQMDRVLREERAYILNLV

jnet
conf        8 7 5 3 6 7 7 7 7 7 7 7 8 7 4 5 7 7 6 2 5 4 7 8 8 9 9 9 9 9 9 9 9 9 9 9 9 9 9 9
```

APICAL DOMAIN

```
                       12                 I          13
1A6D.A Sec Str description

                      290                     300                     310                     320
Hs_CCT8     KAIADTGANVVVTGGKVADMALHYANKYNIMLVRLNSKWD
Hs_CCT6A    KKIIELKRKVVINQKGIDPFSLDALSKEGIVALRRAKRRN
Hs_CCT7     EKIHHSGAKVVLSKLPIGDVATQYFADRDMFCAGRVPEED
Hs_CCT2     ERILKHGINCFINRQLIYNYPEQLFGAAGVMAIEHADFAG
Hs_CCT1     QKILATGANVILTTGGIDDMCLKYFVEAGAMAVRRVLKRD
Hs_CCT3     EDIIQLKPDVVITEKGISDLAQHYLMRANITAIRRVRKTD
Hs_CCT5     QQIKETGANLAICQWGFDDEANHLLLQNNLPAVRWVGGPE
Hs_CCT4     KQIKKTGCNVLLIQKSISDLALHFLNKMKIMVIKDIERED

jnet
conf        9 9 9 9 8 4 5 8 6 4 7 7 4 4 7 8 7 7 4 6 8 8 9 9 8 7 3 5 8 7 4 8 8 8 5 3 5 2 4 6
```

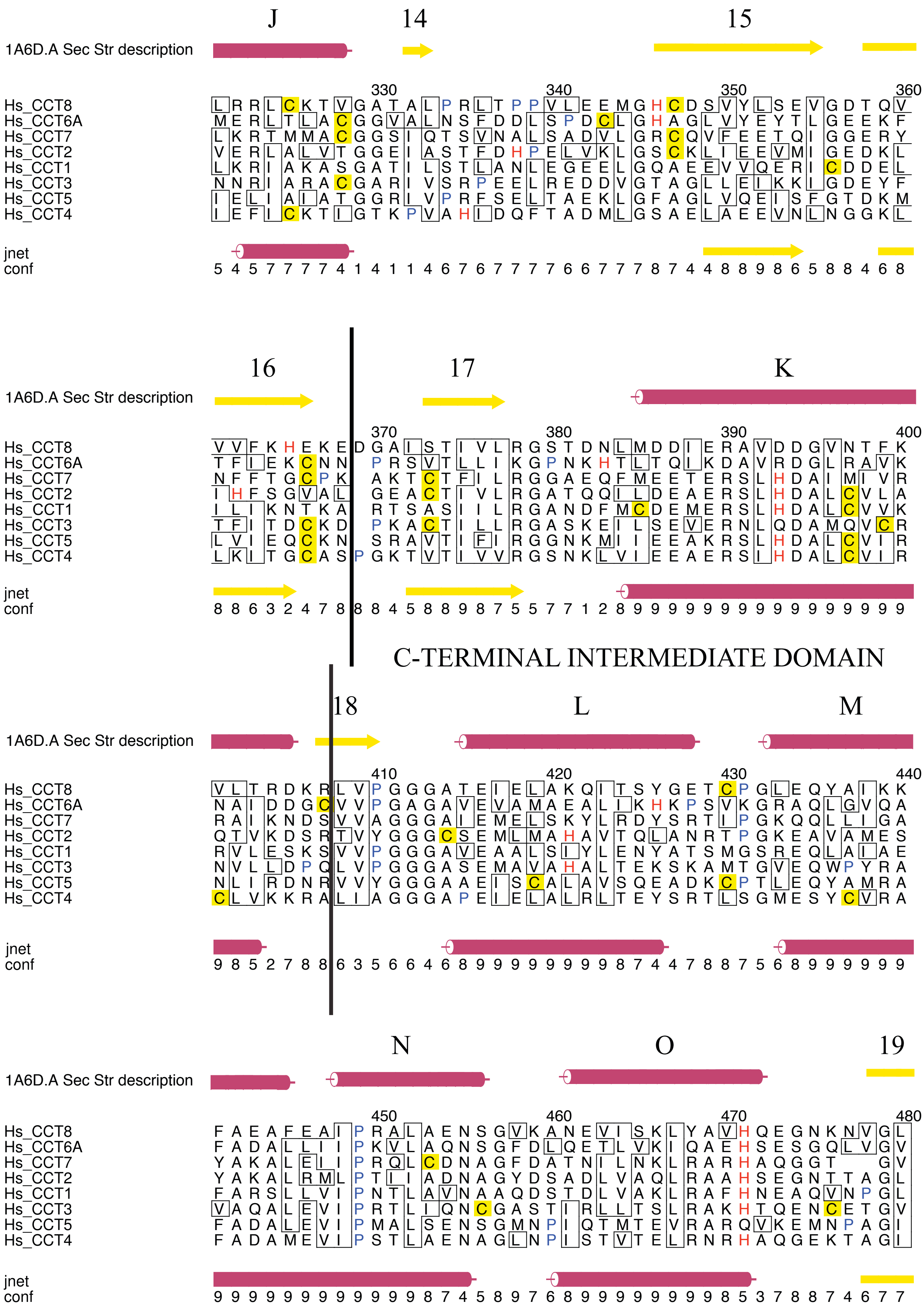
J
14
15
1A6D.A Sec Str description
330
340
350
360
Hs_CCT8 L R R L C K T V G A T A L P R L T P P V L E E M G H C D S V Y L S E V G D T Q V
Hs_CCT6A M E R L T L A C G G V A L N S F D D L S P D C L G H A G L V Y E Y T L G E E K F
Hs_CCT7 L K R T M M A C G G S I Q T S V N A L S A D V L G R C Q V F E E T Q I G G E R Y
Hs_CCT2 V E R L A L V T G G E I A S T F D H P E L V K L G S C K L I E E V M I G E D K L
Hs_CCT1 L K R I A K A S G A T I L S T L A N L E G E E L G Q A E E V V Q E R I C D D E L
Hs_CCT3 N N R I A R A C G A R I V S R P E E L R E D D V G T A G L L E I K K I G D E Y F
Hs_CCT5 I E L I A I A T G G R I V P R F S E L T A E K L G F A G L V Q E I S F G T D K M
Hs_CCT4 I E F I C K T I G T K P V A H I D Q F T A D M L G S A E L A E E V N L N G G K L
jnet
conf 5 4 5 7 7 7 7 4 1 4 1 1 4 6 7 6 7 7 7 6 6 7 7 7 8 7 4 4 8 8 9 8 6 5 8 8 4 6 8
16
17
K
1A6D.A Sec Str description
370
380
390
400
Hs_CCT8 V V F K H E K E D G A I S T I V L R G S T D N L M D D I E R A V D D G V N T F K
Hs_CCT6A T F I E K C N N P R S V T L L I K G P N K H T L T Q I K D A V R D G L R A V K
Hs_CCT7 N F F T G C P K A K T C T F I L R G G A E Q F M E E T E R S L H D A I M I V R
Hs_CCT2 I H F S G V A L G E A C T I V L R G A T Q Q I L D E A E R S L H D A L C V L A
Hs_CCT1 I L I K N T K A R T S A S I I L R G A N D F M C D E M E R S L H D A L C V V K
Hs_CCT3 T F I T D C K D P K A C T I L L R G A S K E I L S E V E R N L Q D A M Q V C R
Hs_CCT5 L V I E Q C K N S R A V T I F I R G G N K M I I E E A K R S L H D A L C V I R
Hs_CCT4 L K I T G C A S P G K T V T I V V R G S N K L V I E E A E R S I H D A L C V I R
jnet
conf 8 8 6 3 2 4 7 8 8 8 4 5 8 8 9 8 7 5 5 7 7 1 2 8 9 9 9 9 9 9 9 9 9 9 9 9 9 9 9 9
C-TERMINAL INTERMEDIATE DOMAIN
18
L
M
1A6D.A Sec Str description
410
420
430
440
Hs_CCT8 V L T R D K R L V P G G G A T E I E L A K Q I T S Y G E T C P G L E Q Y A I K K
Hs_CCT6A N A I D D G C V V P G A G A V E V A M A E A L I K H K P S V K G R A Q L G V Q A
Hs_CCT7 R A I K N D S V V A G G G A I E M E L S K Y L R D Y S R T I P G K Q Q L L I G A
Hs_CCT2 Q T V K D S R T V Y G G G C S E M L M A H A V T Q L A N R T P G K E A V A M E S
Hs_CCT1 R V L E S K S V V P G G G A V E A A L S I Y L E N Y A T S M G S R E Q L A I A E
Hs_CCT3 N V L L D P Q L V P G G G A S E M A V A H A L T E K S K A M T G V E Q W P Y R A
Hs_CCT5 N L I R D N R V V Y G G G A A E I S C A L A V S Q E A D K C P T L E Q Y A M R A
Hs_CCT4 C L V K K R A L I A G G G A P E I E L A L R L T E Y S R T L S G M E S Y C V R A
jnet
conf 9 8 5 2 7 8 8 6 3 5 6 6 4 6 8 9 9 9 9 9 9 9 9 8 7 4 4 7 8 8 7 5 6 8 9 9 9 9 9 9
N
O
19
1A6D.A Sec Str description
450
460
470
480
Hs_CCT8 F A E A F E A I P R A L A E N S G V K A N E V I S K L Y A V H Q E G N K N V G L
Hs_CCT6A F A D A L L I I P K V L A Q N S G F D L Q E T L V K I Q A E H S E S G Q L V G V
Hs_CCT7 Y A K A L E I I P R Q L C D N A G F D A T N I L N K L R A R H A Q G G T G V
Hs_CCT2 Y A K A L R M L P T I I A D N A G Y D S A D L V A Q L R A A H S E G N T T A G L
Hs_CCT1 F A R S L L V I P N T L A V N A A Q D S T D L V A K L R A F H N E A Q V N P G L
Hs_CCT3 V A Q A L E V I P R T L I Q N C G A S T I R L L T S L R A K H T Q E N C E T G V
Hs_CCT5 F A D A L E V I P M A L S E N S G M N P I Q T M T E V R A R Q V K E M N P A G I
Hs_CCT4 F A D A M E V I P S T L A E N A G L N P I S T V T E L R N R H A Q G E K T A G I
jnet
conf 9 9 9 9 9 9 9 9 9 9 9 9 8 7 4 5 8 9 7 6 8 9 9 9 9 9 9 9 9 8 5 3 7 8 8 7 4 6 7 7

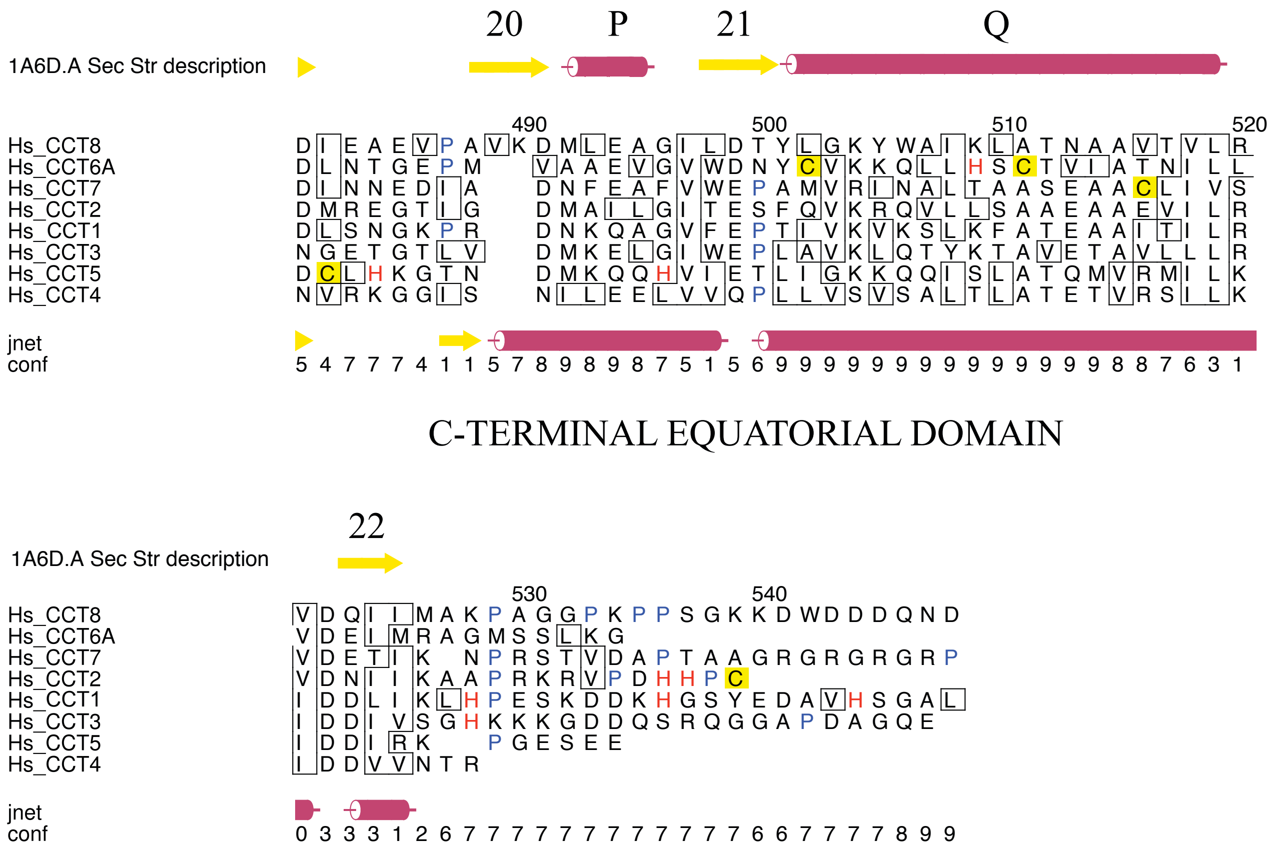

Supplementary figure S11. Alignment and secondary-structure predictions of human CCT sequences compared to PDB secondary-structure description of 1a6d. For an explanation of abbreviations and symbols see Legend for Supplementary figure S10.
