# Supplementary material for: Chaperonin genes on the rise: new divergent classes and intense duplication in human and other vertebrate genomes
Source: BMC Evol Biol. 2010 Mar 1;10:64. doi: 10.1186/1471-2148-10-64 (PMC2846930; doi:10.1186/1471-2148-10-64)
Supplement: Additional file 17 — Table S12. Alignment and secondary-structure prediction of vertebrate CCT8L protein sequences. [file 1471-2148-10-64-S17.PDF]

```
                              1    A
1A6D.A Sec Str description

            1        10        20        30        40
Md_024812   MADPDPSAVNFPQLMNKEIKCLSKQEKSLLGSVAAIKTLA
Mm_Gm443    TQTKVQSDLELPQRLKPGLEKTPEEPSYILRATAAAQTLA
Rn_125233   TQTKVQSDLELPQRLKLGLEKNPEEPLCILRATAAAQTLA
Ec_CCT8L    MGSRAPSATGLPERLEPGPGQRPAEEQHVLSSVAAAQALA
Hs_CCT8L1   MDSTVPSALELPQRLALNPRESPEEEPHLLSSLAAVQTLA
Hs_CCT8L2   MDSTVPSALELPQRLALNPRESPEEEPHLLSSLAAVQTLA
Ptr_470129  MDSTVPSALELPQRLALNPRESPEEEPHLLSSLAAVQTLA
Cf_482811   MAGGATAAPPLPERLGPGPRRRAAQEEHLLCSVPAAQTLA
Bt_618575   MDRRAPTAPELPERLP       AEEKHLLSSLAAADTLA
Dn_CCT8L    MDNRVPSALDLPQLLAQSTEQSTRGEMYLLSSTAAVQALA
La_CCT8L    MDTRGPLAPELPQLLHRSPQPCAAGEKNVLSSTAAAQALA

jnet
conf        9 9 9 8 8 8 8 7 7 6 4 6 8 9 9 9 8 7 4 3 7 8 8 7 6 4 2 0 0 0 5 5 7 9 9 9 9 9 9 9
```

```
                              2        3       B
1A6D.A Sec Str description

                     50        60        70        80
Md_024812   GILRSCYGPYGRLKYLVTSQGKTVCTGYAATILGALELEH
Mm_Gm443    SIIRSCYGPFGRQKFLVTAKGETVCTGHAAAILKALDLEH
Rn_125233   SIIRSCYGPYGLQKFLVSAQGETVCTGHAAAILKALELEH
Ec_CCT8L    RVIRPCYGPHGRQKLLVTARGDTVVTGYAAAILRALELEH
Hs_CCT8L1   NVIRPCYGPHGRQKFLVTMKGETVCTGCATAILRALELEH
Hs_CCT8L2   SVIRPCYGPHGRQKFLVTMKGETVCTGCATAILRALELEH
Ptr_470129  SVIRPCYGPHGRQKFLVTMKGETVCTGCATAILRALELEH
Cf_482811   RVIRPCYGPHGRQKLLVTARGTTVFTGSAAAILQALELEH
Bt_618575   RVLRPCYGPQGRQKLLVTAKGDTVLTGHAAAILRALQLEH
Dn_CCT8L    SVIRSCFGPCGRQKLLVTAKGETICTGYTTAILQALELEH
La_CCT8L    TLIRPCYGPHGRQKLLVTATGETVCTGCATAILRALELEH

jnet
conf        9 8 7 5 3 7 8 8 8 8 8 5 4 8 8 9 8 5 5 8 8 3 4 6 5 2 3 7 7 8 8 7 7 6 3 4 8 8 8 6
```

N-TERMINAL EQUATORIAL DOMAIN

```
                  C                      D
1A6D.A Sec Str description

                     90        100       110       120
Md_024812   PAAQLLREAAQTQAENSGDGTAFVVLLAGALLEQAEAMVR
Mm_Gm443    PAAQFVQELAQTQVENAGDGTVFVVLLTEALLEQAHYLLW
Rn_125233   PAARFVQELAQTQAENTGDGTAFVVLLTEALLEQAQYLLW
Ec_CCT8L    PAARLLREAAQTQAEXNGDEAAFVGLLCSA LERTERLLR
Hs_CCT8L1   PAAWLLREAAQTQAENSGDGTAFVVLLTEALLEQAEQLLK
Hs_CCT8L2   PAAWLLREAGQTQAENSGDGTAFVVLLTEALLEQAEQLLK
Ptr_470129  PAAWLLREAGQTQAENSGDGTAFVVLLTEALLEQAEQLLK
Cf_482811   PAARLLREAAHKQAESCGDGAAFVVLLAEALLQQAEHLLR
Bt_618575   PAARLLREAAQGQAEQSGDGAAFVVLLAQALLAQAERLLR
Dn_CCT8L    PAAWLLREAAQTQAENSGDGTAFVVLLTQGLLEQAEHVLR
La_CCT8L    PAAWLLREASHTQAESRGDGTAFVVLLAGALLEQAELLLR

jnet
conf        6 8 9 9 9 9 9 9 9 9 9 8 8 4 3 7 8 8 8 6 4 1 4 6 7 8 9 9 9 9 9 9 9 9 9 9 9 9 8 7
```

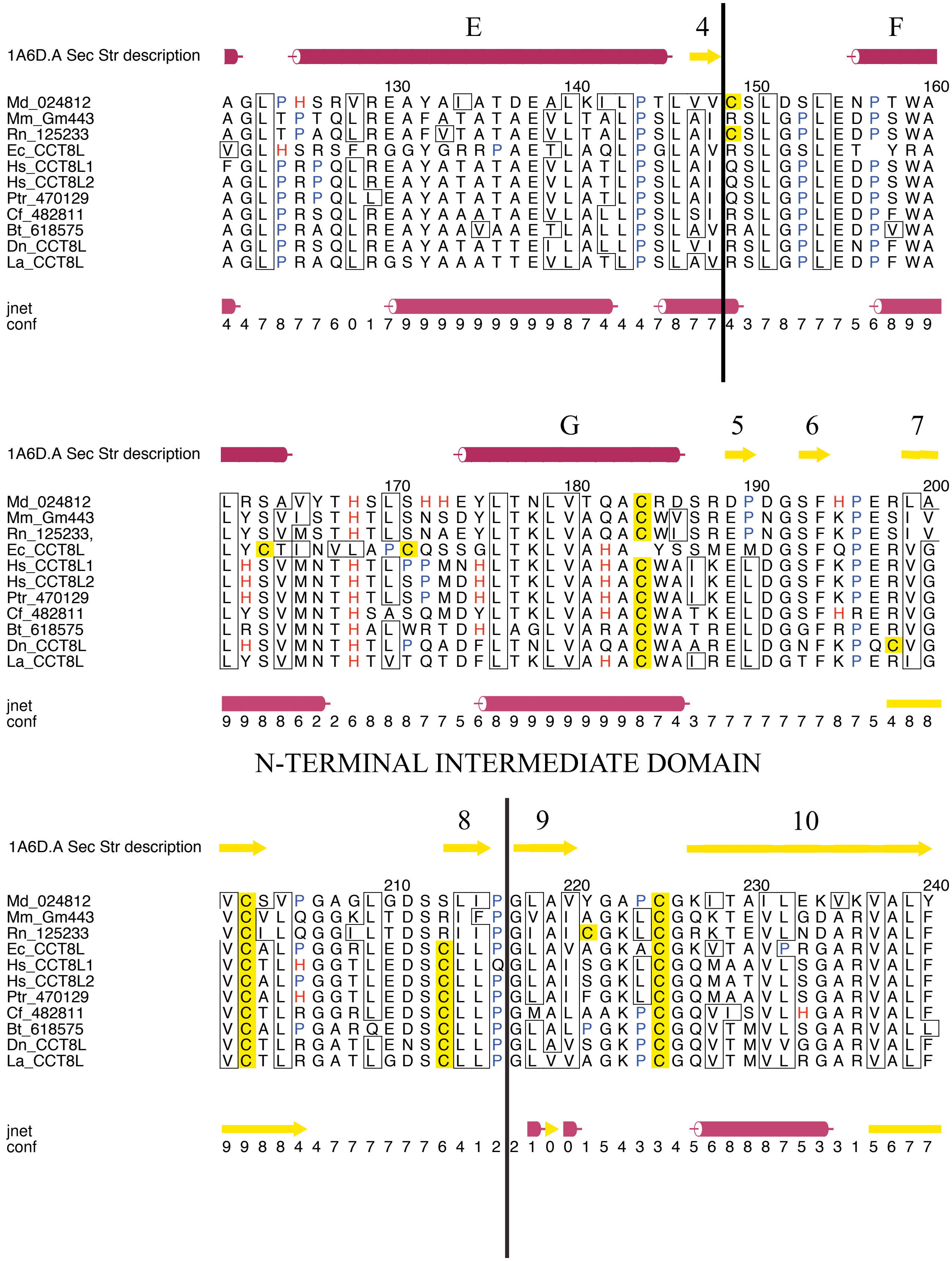
E 4 F
1A6D.A Sec Str description
130 140 150 160
Md_024812 A G L P H S R V R E A Y A I A T D E A L K I L P T L V V C S L D S L E N P T W A
Mm_Gm443 A G L T P T Q L R E A F A T A T A E V L T A L P S L A I R S L G P L E D P S W A
Rn_125233 A G L T P A Q L R E A F V T A T A E V L T A L P S L A I C S L G P L E D P S W A
Ec_CCT8L V G L H S R S F R G G Y G R R P A E T L A Q L P G L A V R S L G S L E T Y R A
Hs_CCT8L1 F G L P R P Q L R E A Y A T A T A E V L A T L P S L A I Q S L G P L E D P S W A
Hs_CCT8L2 A G L P R P Q L R E A Y A T A T A E V L A T L P S L A I Q S L G P L E D P S W A
Ptr_470129 A G L P R P Q L L E A Y A T A T A E V L A T L P S L A I Q S L G P L E D P S W A
Cf_482811 A G L P R S Q L R E A Y A A A T A E V L A L L P S L S I R S L G P L E D P F W A
Bt_618575 A G L P R A Q L R E A Y A A V A A E T L A L L P S L A V R A L G P L E D P V W A
Dn_CCT8L A G L P R S Q L R E A Y A T A T T E I L A L L P S L V I R S L G P L E N P F W A
La_CCT8L A G L P R A Q L R G S Y A A A T T E V L A T L P S L A V R S L G P L E D P F W A
jnet
conf 4 4 7 8 7 7 6 0 1 7 9 9 9 9 9 9 9 9 9 8 7 4 4 4 7 8 7 7 4 3 7 8 7 7 7 5 6 8 9 9
G 5 6 7
1A6D.A Sec Str description
170 180 190 200
Md_024812 L R S A V Y T H S L S H H E Y L T N L V T Q A C R D S R D P D G S F H P E R L A
Mm_Gm443 L Y S V I S T H T L S N S D Y L T K L V A Q A C W V S R E P N G S F K P E S I V
Rn_125233, L Y S V M S T H T L S N A E Y L T K L V A Q A C W I S R E P N G S F K P E S I V
Ec_CCT8L L Y C T I N V L A P C Q S S G L T K L V A H A Y S S M E M D G S F Q P E R V G
Hs_CCT8L1 L H S V M N T H T L P P M N H L T K L V A H A C W A I K E L D G S F K P E R V G
Hs_CCT8L2 L H S V M N T H T L S P M D H L T K L V A H A C W A I K E L D G S F K P E R V G
Ptr_470129 L H S V M N T H T L S P M D H L T K L V A H A C W A I K E L D G S F K P E R V G
Cf_482811 L Y S V M N T H S A S Q M D Y L T K L V A H A C W A T K E L D G S F H R E R V G
Bt_618575 L R S V M N T H A L W R T D H L A G L V A R A C W A T R E L D G G F R P E R V G
Dn_CCT8L L H S V M N T H T L P Q A D F L T N L V A Q A C W A A R E L D G N F K P Q C V G
La_CCT8L L Y S V M N T H T V T Q T D F L T K L V A H A C W A I R E L D G T F K P E R I G
jnet
conf 9 9 8 8 6 2 2 6 8 8 8 7 7 5 6 8 9 9 9 9 9 9 9 8 7 4 3 7 7 7 7 7 7 7 8 7 5 4 8 8
N-TERMINAL INTERMEDIATE DOMAIN
8 9 10
1A6D.A Sec Str description
210 220 230 240
Md_024812 V C S V P G A G L G D S S L I P G L A V Y G A P C G K I T A I L E K V K V A L Y
Mm_Gm443 V C V L Q G G K L T D S R I F P G V A I A G K L C G Q K T E V L G D A R V A L F
Rn_125233 V C I L Q G G I L T D S R I I P G I A I C G K L C G R K T E V L N D A R V A L F
Ec_CCT8L V C A L P G G R L E D S C L L P G L A V A G K A C G K V T A V P R G A R V A L F
Hs_CCT8L1 V C T L H G G T L E D S C L L Q G L A I S G K L C G Q M A A V L S G A R V A L F
Hs_CCT8L2 V C A L P G G T L E D S C L L P G L A I S G K L C G Q M A T V L S G A R V A L F
Ptr_470129 V C A L H G G T L E D S C L L P G L A I F G K L C G Q M A A V L S G A R V A L F
Cf_482811 V C T L R G G R L E D S C L L P G M A L A A K P C G Q V I S V L H G A R V A L F
Bt_618575 V C A L P G A R Q E D S C L L P G L A L P G K P C G Q V T M V L S G A R V A L L
Dn_CCT8L V C T L R G A T L E N S C L L P G L A V S G K P C G Q V T M V V G G A R V A L F
La_CCT8L V C T L R G A T L G D S C L L P G L V V A G K P C G Q V T M V L R G A R V A L F
jnet
conf 9 9 8 8 4 4 7 7 7 7 7 7 7 6 4 1 2 2 1 0 0 1 5 4 3 3 4 5 6 8 8 8 7 5 3 3 1 5 6 7 7

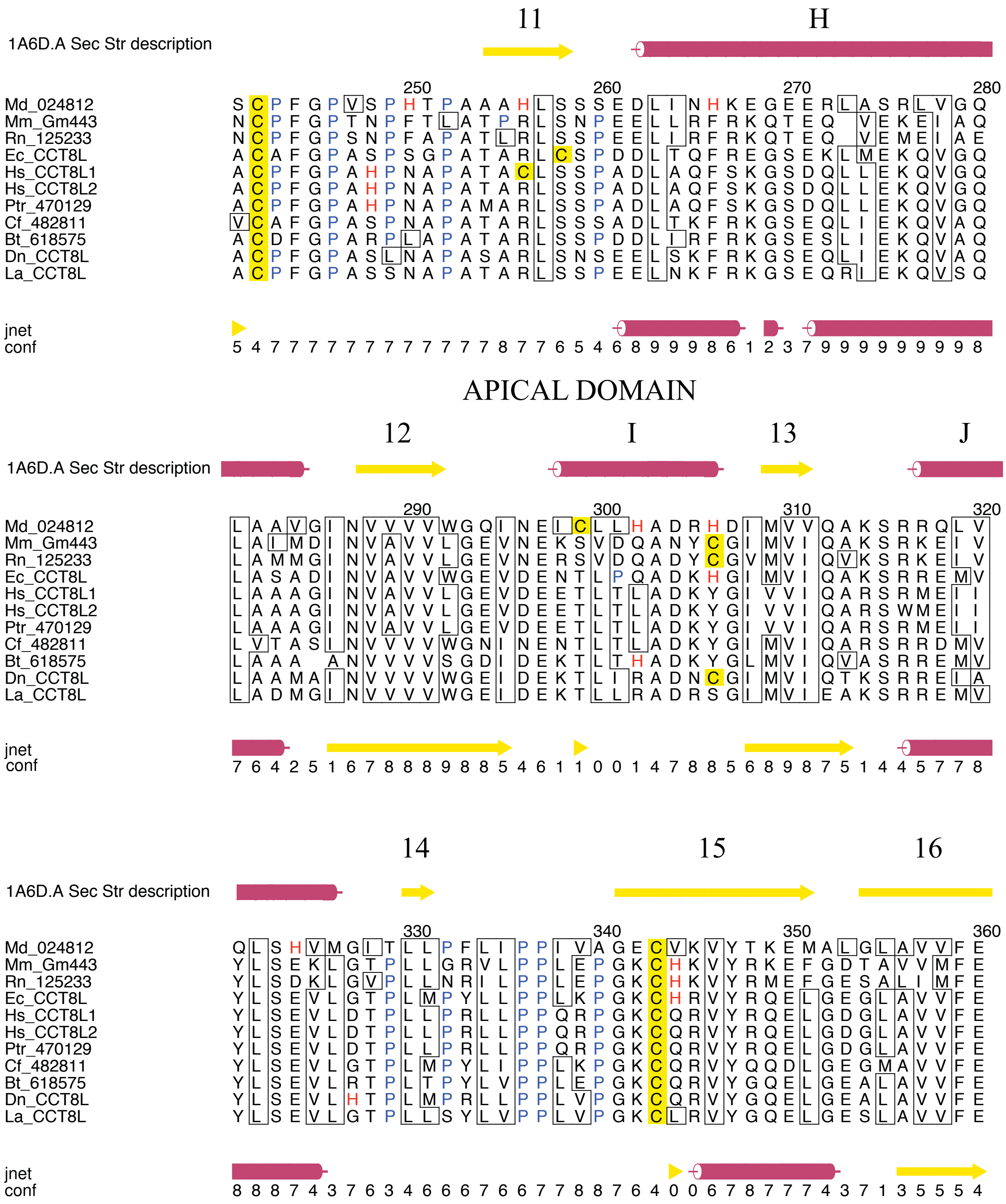
APICAL DOMAIN
1A6D.A Sec Str description
11
H
250
260
270
280
Md_024812 S C P F G P V S P H T P A A A H L S S S E D L I N H K E G E E R L A S R L V G Q
Mm_Gm443 N C P F G P T N P F T L A T P R L S N P E E L L R F R K Q T E Q V E K E I A Q
Rn_125233 N C P F G P S N P F A P A T L R L S S P E E L I R F R K Q T E Q V E M E I A E
Ec_CCT8L A C A F G P A S P S G P A T A R L C S P D D L T Q F R E G S E K L M E K Q V G Q
Hs_CCT8L1 A C P F G P A H P N A P A T A C L S S P A D L A Q F S K G S D Q L L E K Q V G Q
Hs_CCT8L2 A C P F G P A H P N A P A T A R L S S P A D L A Q F S K G S D Q L L E K Q V G Q
Ptr_470129 A C P F G P A H P N A P A M A R L S S P A D L A Q F S K G S D Q L L E K Q V G Q
Cf_482811 V C A F G P A S P N A P A T A R L S S S A D L T K F R K G S E Q L I E K Q V A Q
Bt_618575 A C D F G P A R P L A P A T A R L S S P D D L I R F R K G S E S L I E K Q V A Q
Dn_CCT8L A C P F G P A S L N A P A S A R L S N S E E L S K F R K G S E Q L I E K Q V A Q
La_CCT8L A C P F G P A S S N A P A T A R L S S P E E L N K F R K G S E Q R I E K Q V S Q
jnet
conf 5 4 7 7 7 7 7 7 7 7 7 7 7 8 7 7 6 5 4 6 8 9 9 9 8 6 1 2 3 7 9 9 9 9 9 9 9 8
1A6D.A Sec Str description
12
I
13
J
290
300
310
320
Md_024812 L A A V G I N V V V V W G Q I N E I C L L H A D R H D I M V V Q A K S R R Q L V
Mm_Gm443 L A I M D I N V A V V L G E V N E K S V D Q A N Y C G I M V I Q A K S R K E I V
Rn_125233 L A M M G I N V A V V L G E V N E R S V D Q A D Y C G V M V I Q V K S R K E I V
Ec_CCT8L L A S A D I N V A V V W G E V D E N T L P Q A D K H G I M V I Q A K S R R E M V
Hs_CCT8L1 L A A A G I N V A V V L G E V D E E T L T L A D K Y G I V V I Q A R S R M E I I
Hs_CCT8L2 L A A A G I N V A V V L G E V D E E T L T L A D K Y G I V V I Q A R S W M E I I
Ptr_470129 L A A A G I N V A V V L G E V D E E T L T L A D K Y G I V V I Q A R S R M E I I
Cf_482811 L V T A S I N V V V V W G N I N E N T L T L A D K Y G I M V I Q A R S R R D M V
Bt_618575 L A A A A N V V V V S G D I D E K T L T H A D K Y G L M V I Q V A S R R E M V
Dn_CCT8L L A A M A I N V V V V W G E I D E K T L I R A D N C G I M V I Q T K S R R E I A
La_CCT8L L A D M G I N V V V V W G E I D E K T L L R A D R S G I M V I E A K S R R E M V
jnet
conf 7 6 4 2 5 1 6 7 8 8 8 9 8 8 5 4 6 1 1 0 0 1 4 7 8 8 5 6 8 9 8 7 5 1 4 4 5 7 7 8
1A6D.A Sec Str description
14
15
16
330
340
350
360
Md_024812 Q L S H V M G I T L L P F L I P P I V A G E C V K V Y T K E M A L G L A V V F E
Mm_Gm443 Y L S E K L G T P L L G R V L P P L E P G K C H K V Y R K E F G D T A V V M F E
Rn_125233 Y L S D K L G V P L L N R I L P P L E P G K C H K V Y R M E F G E S A L I M F E
Ec_CCT8L Y L S E V L G T P L M P Y L L P P L K P G K C H R V Y R Q E L G E G L A V V F E
Hs_CCT8L1 Y L S E V L D T P L L P R L L P P Q R P G K C Q R V Y R Q E L G D G L A V V F E
Hs_CCT8L2 Y L S E V L D T P L L P R L L P P Q R P G K C Q R V Y R Q E L G D G L A V V F E
Ptr_470129 Y L S E V L D T P L L P R L L P P Q R P G K C Q R V Y R Q E L G D G L A V V F E
Cf_482811 Y L S E V L G T P L M P Y L I P P L K P G K C Q R V Y Q Q D L G E G M A V V F E
Bt_618575 Y L S E V L R T P L T P Y L V P P L E P G K C Q R V Y G Q E L G E A L A V V F E
Dn_CCT8L Y L S E V L H T P L M P R L L P P L V P G K C Q R V Y G Q E L G E A L A V V F E
La_CCT8L Y L S E V L G T P L L S Y L V P P L V P G K C L R V Y G Q E L G E S L A V V F E
jnet
conf 8 8 8 7 4 3 7 6 3 4 6 6 6 7 6 6 7 7 8 8 7 6 4 0 0 6 7 8 7 7 7 4 3 7 1 3 5 5 5 4

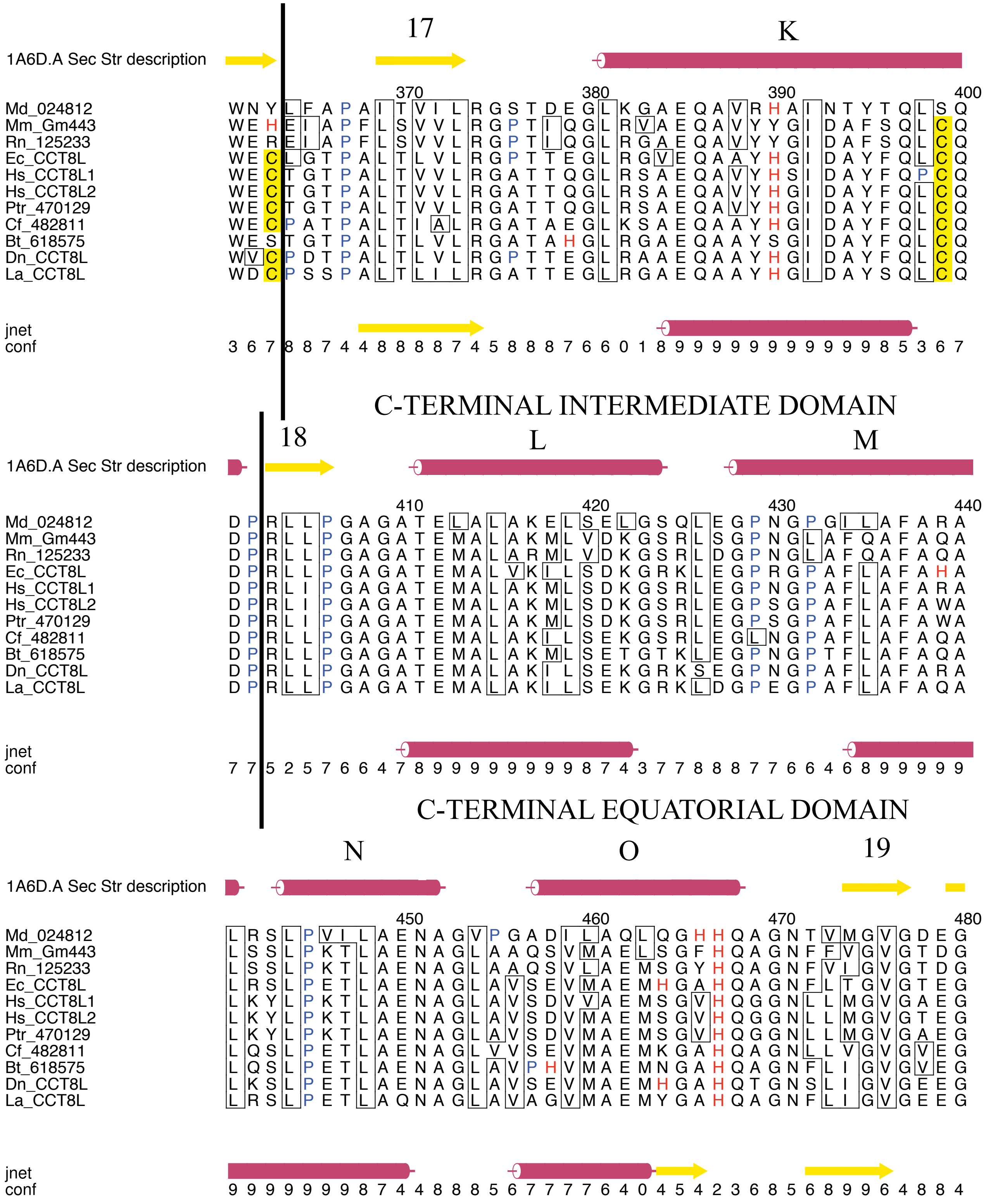
17
K
1A6D.A Sec Str description
370 380 390 400
Md_024812 W N Y L F A P A I T V I L R G S T D E G L K G A E Q A V R H A I N T Y T Q L S Q
Mm_Gm443 W E H E I A P F L S V V L R G P T I Q G L R V A E Q A V Y Y G I D A F S Q L C Q
Rn_125233 W E R E I A P F L S V V L R G P T I Q G L R G A E Q A V Y Y G I D A F S Q L C Q
Ec_CCT8L W E C L G T P A L T L V L R G P T T E G L R G V E Q A A Y H G I D A Y F Q L C Q
Hs_CCT8L1 W E C T G T P A L T V V L R G A T T Q G L R S A E Q A V Y H S I D A Y F Q P C Q
Hs_CCT8L2 W E C T G T P A L T V V L R G A T T Q G L R S A E Q A V Y H G I D A Y F Q L C Q
Ptr_470129 W E C T G T P A L T V V L R G A T T Q G L R S A E Q A V Y H G I D A Y F Q L C Q
Cf_482811 W E C P A T P A L T I A L R G A T A E G L K S A E Q A A Y H G I D A Y F Q L C Q
Bt_618575 W E S T G T P A L T L V L R G A T A H G L R G A E Q A A Y S G I D A Y F Q L C Q
Dn_CCT8L W V C P D T P A L T L V L R G P T T E G L R A A E Q A A Y H G I D A Y F Q L C Q
La_CCT8L W D C P S S P A L T L I L R G A T T E G L R G A E Q A A Y H G I D A Y S Q L C Q
jnet
conf 3 6 7 8 8 7 4 4 8 8 8 8 7 4 5 8 8 8 7 6 6 0 1 8 9 9 9 9 9 9 9 9 9 9 9 8 5 3 6 7
C-TERMINAL INTERMEDIATE DOMAIN
18
L
M
1A6D.A Sec Str description
410 420 430 440
Md_024812 D P R L L P G A G A T E L A L A K E L S E L G S Q L E G P N G P G I L A F A R A
Mm_Gm443 D P R L L P G A G A T E M A L A K M L V D K G S R L S G P N G L A F Q A F A Q A
Rn_125233 D P R L L P G A G A T E M A L A R M L V D K G S R L D G P N G L A F Q A F A Q A
Ec_CCT8L D P R L L P G A G A T E M A L V K I L S D K G R K L E G P R G P A F L A F A H A
Hs_CCT8L1 D P R L I P G A G A T E M A L A K M L S D K G S R L E G P N G P A F L A F A R A
Hs_CCT8L2 D P R L I P G A G A T E M A L A K M L S D K G S R L E G P S G P A F L A F A W A
Ptr_470129 D P R L I P G A G A T E M A L A K M L S D K G S R L E G P S G P A F L A F A W A
Cf_482811 D P R L L P G A G A T E M A L A K I L S E K G S R L E G L N G P A F L A F A Q A
Bt_618575 D P R L L P G A G A T E M A L A K M L S E T G T K L E G P N G P T F L A F A Q A
Dn_CCT8L D P R L L P G A G A T E M A L A K I L S E K G R K S E G P N G P A F L A F A R A
La_CCT8L D P R L L P G A G A T E M A L A K I L S E K G R K L D G P E G P A F L A F A Q A
jnet
conf 7 7 5 2 5 7 6 6 4 7 8 9 9 9 9 9 9 9 9 8 7 4 3 7 7 8 8 8 7 7 6 6 4 6 8 9 9 9 9 9
C-TERMINAL EQUATORIAL DOMAIN
N
O
19
1A6D.A Sec Str description
450 460 470 480
Md_024812 L R S L P V I L A E N A G V P G A D I L A Q L Q G H H Q A G N T V M G V G D E G
Mm_Gm443 L S S L P K T L A E N A G L A A Q S V M A E L S G F H Q A G N F F V G V G T D G
Rn_125233 L S S L P K T L A E N A G L A A Q S V L A E M S G Y H Q A G N F V I G V G T D G
Ec_CCT8L L R S L P E T L A E N A G L A V S E V M A E M H G A H Q A G N F L T G V G T E G
Hs_CCT8L1 L K Y L P K T L A E N A G L A V S D V V A E M S G V H Q G G N L L M G V G A E G
Hs_CCT8L2 L K Y L P K T L A E N A G L A V S D V M A E M S G V H Q G G N L L M G V G T E G
Ptr_470129 L K Y L P K T L A E N A G L A V S D V M A E M S G V H Q G G N L L M G V G A E G
Cf_482811 L Q S L P E T L A E N A G L V V S E V M A E M K G A H Q A G N L L V G V G V E G
Bt_618575 L Q S L P E T L A E N A G L A V P H V M A E M N G A H Q A G N F L I G V G V E G
Dn_CCT8L L K S L P E T L A E N A G L A V S E V M A E M H G A H Q T G N S L I G V G E E G
La_CCT8L L R S L P E T L A Q N A G L A V A G V M A E M Y G A H Q A G N F L I G V G E E G
jnet
conf 9 9 9 9 9 9 9 8 7 4 4 8 8 8 5 6 7 7 7 7 6 4 0 4 5 4 2 3 6 8 5 6 8 9 9 6 4 8 8 4

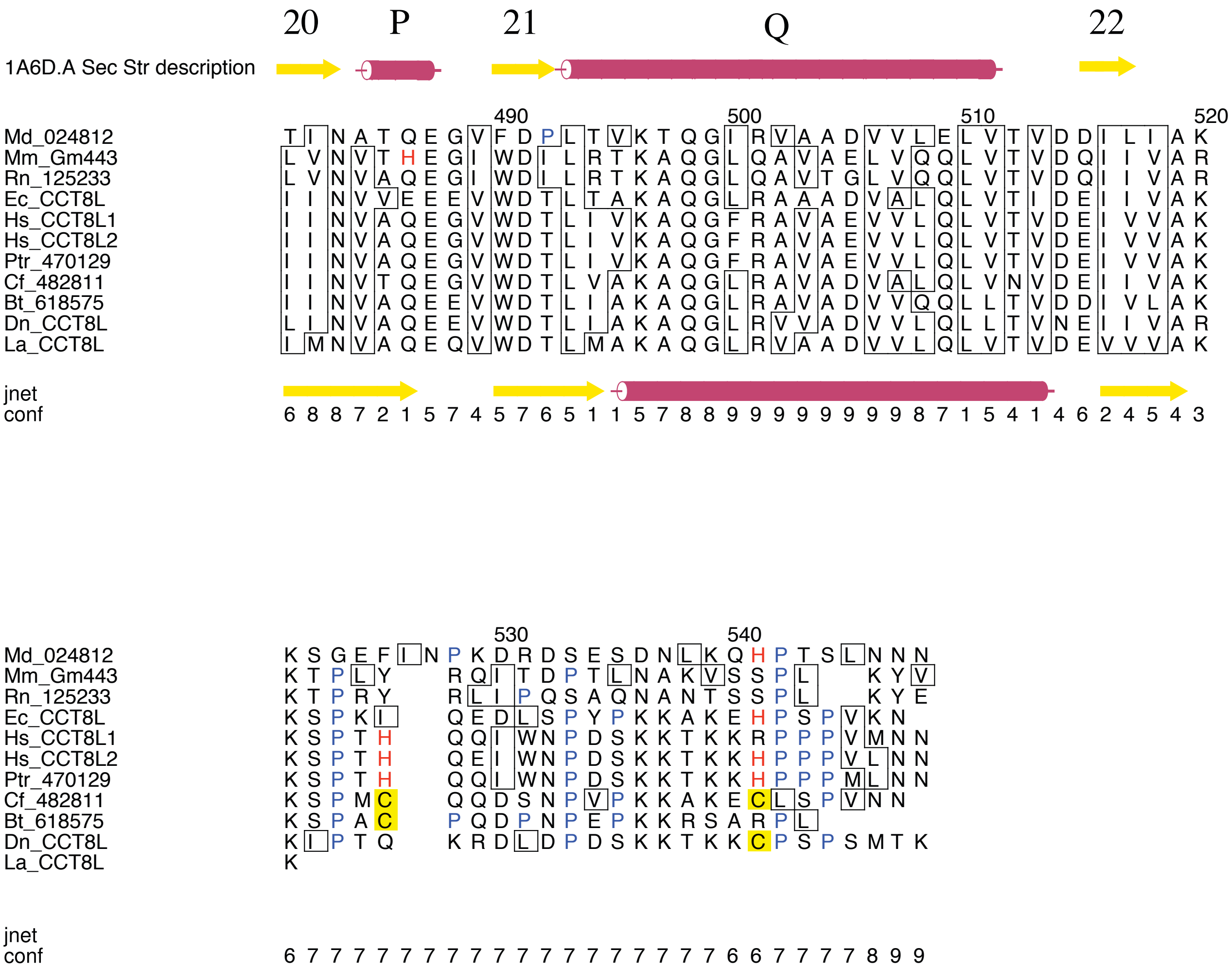

Supplementary figure S12. Alignment and secondary-structure predictions of CCT8L sequences compared to PDB secondary-structure description of 1a6d. See Legend for Supplementary figure S10 for symbols and Legend for Figure 2 for species abbreviations.
