# Supplementary material for: Chaperonin genes on the rise: new divergent classes and intense duplication in human and other vertebrate genomes
Source: BMC Evol Biol. 2010 Mar 1;10:64. doi: 10.1186/1471-2148-10-64 (PMC2846930; doi:10.1186/1471-2148-10-64)
Supplement: Additional file 18 — Table S13. Alignment and secondary-structure prediction of vertebrate MKKS protein sequences. [file 1471-2148-10-64-S18.PDF]

```
                               1          A
1A6D.A Sec Str description

             1                 10                  20                  30                  40
Xt_sca_8     M S R V E A K K P S V C T T G P M N I Q S V R D S L S V L H G V I M S C Y G P L
Gg_MKKS      M S R L E A K K P S L F I S E P L T T - - V S Q P L S L L I A I L K S C Y G P A
Md_31632     M S R V K A K T P S L C T C E L L T K E I V S K S L S G L R E I V A S C Y G P S
Oa_0078571   M S R L E A K K P S L C T S V Q L T K D V V S Q S L A V L R G I V A S C Y G P C
Mm_Mkks      M S R L E A K K P S L C K T E P L T S E K V R S T L S V L K G V I A S C Y G P S
Rn_Mkks      M S R L E A K K P S L C K T E P L T S E R V R S T L S V L K G I I A S C Y G P S
Bt_614288    M S R L E A K K P S I C R S D P L T G E R A R A S L A A L K G L V M S C Y G P A
Cf_485771    M S R L E A K K P S L C K S E P L T S E R V R A T L C V L K G I L T S S Y G P S
Ec_64409     M S R L E A K K P S L C K S E P L T S E R V R A T L S V L K G I V T S C Y G P S
Mmu_MKKS     M S R L E A K K P S L C K S E P L T S E R V R T T L S V L K R I V T S C Y G P S
Pp_L0833     M S R L E A K K P S L C K S E P L T T E R V R T T L S V F K R I V T S C Y G P S
Hs_MKKS      M S R L E A K K P S L C K S E P L T T E R V R T T L S V L K R I V T S C Y G P S
Ptr_MKKS     M S R L E A K K P S L C K S E P L T T E R V R T T L S V L K R I V T S C Y G P S
Dr_mkks      M S R I S K K K P A L C T D E P L S N S T I C Q K I T L L R N I L S T A Y G P T
Ol_EN15281   M S R V E K K A P S V C T D L P L N N A E V C E K L H L M K E L L K S C Y G P R
Ga_EN05932   M S R L V Q K S P S L C T D L P L D N S D I C T K L Y L L R Q L L K S C F G P T
Tr_SI175074  M S R L V K K S P S V C T D L P L D T T D S W K K L R V L L Q L L K S C F G P K

jnet
conf         9 9 8 7 7 7 7 7 6 3 3 5 3 4 7 7 7 7 6 4 6 8 9 9 9 9 9 9 9 9 9 9 8 7 5 3 7 8 7 6


                  2          3        B              C
1A6D.A Sec Str description

                                   50                  60                  70                  80
Xt_sca_8     G R I K Q V H N G T G G C V L T T S Q S S A L F N S F S V S K P V A K L L V A S
Gg_MKKS      G R L K Q L H N G V G G Y V C L T S Q S S A I L G H L S V S H P V L K V L T A S
Md_31632     G R L K Q L H N G V G G S I C T T S S S S L L F N N L S V T H P I L K I L T T S
Oa_0078571   G R L K Q L H N G V G G C V C T T S Q S S A I L G S L S V T H P V L K I L T A S
Mm_Mkks      G R L K Q L H N G L G G C V Y T T S Q S S A L L R N L S V T H P V L K I L T S S
Rn_Mkks      G R L K Q L H N G L G G C V C T T S Q S S A L L R N L S V T H P I L K V L T S S
Bt_614288    G R L K Q L H N G R G G S V V T T S Q S A A L L A G L P V S H P V L K V L T A A
Cf_485771    G R L K Q V H S G F G G C V C T T S Q S S A L L S N L P V T H P I L K I L T T S
Ec_64409     G R L K Q L H N G L G G C V C T T S Q S S A L L A N L S V T H P I L K I L T T A
Mmu_MKKS     G R L K Q L H N G F G G Y V C T T S Q S S A L L S H L L V T H P I L K I L T T S
Pp_L0833     G R L K Q L H N G F G G Y V C T T S Q S S A L L S H L L V T H P I L K I L T T S
Hs_MKKS      G R L K Q L H N G F G G Y V C T T S Q S S A L L S H L L V T H P I L K I L T A S
Ptr_MKKS     G R L K Q L H N G F G G Y V C T T S Q S S A L L S H L L V T H P I L K I L T T S
Dr_mkks      G R L K Q I H N N V G G H V L T T S T S T A L L K R L E M S E P L L K L I S T A
Ol_EN15281   G R L K H V R N N I G G R V T T S S A S S V I L P A L Y S S Q P L L N L I K T A
Ga_EN05932   G R L K Q V H N N I G G H V I T T S S S S V L L P A I S S S Q S F I N L I K T S
Tr_SI175074  G R L K L L H N S I G G H V V T T S T S S V L L S A I S S S L P L I N L I K T S

jnet
conf         5 1 3 6 7 7 5 4 8 8 4 5 8 8 6 5 1 4 5 7 6 6 5 3 4 8 6 1 2 7 9 9 9 9 9 9 9 9 9 9


                                           D                  E
1A6D.A Sec Str description

                                   90                  100                 110                 120
Xt_sca_8     I R N H I S C F S D S G L F A A S L C C Y L V D H F F N L N I A R H T V I K V S
Gg_MKKS      V Q N H V S R F S D C G L F T A I L C C S L I E N F K N L N I A S C T I I K I S
Md_31632     V Q N H V S H F S D C G L F T A I L C C H L I E N F Q R L N L S S A T V I K V S
Oa_0078571   V Q N H L T R F S D C G L F T A I L C C N L I E R C Q K V N L A P R T V T E A N
Mm_Mkks      V Q N H V S C F S D C G L F T A I L C C N L I E N I Q R L D L T P A T A I K L N
Rn_Mkks      V Q N H V C C F S D C G L F T A I L C C N L I E N I Q R I G L T P T T A I K L N
Bt_614288    V R N H V A C F S D G G L F T A I L C C N L V E N V Q R L G L A P T T V I K L N
Cf_485771    M Q N H V S C F R D C G L F T A I L C C N L I E N V Q R T G L T P T T V T K L N
Ec_64409     M Q N H V S C F S D C G L F T A I L C C N L I E N V Q R I G L T P T T V M K L N
Mmu_MKKS     V Q N H V S S F S D C G L F T A I L C C N L I E N V Q R L G L T P T T V I R L N
Pp_L0833     I Q N H V S S F S D C G L F T A I L C C N L I E N V Q R L D L T P T T V I R L N
Hs_MKKS      I Q N H V S S F S D C G L F T A I L C C N L I E N V Q R L G L T P T T V I R L N
Ptr_MKKS     I Q N H V S S F S D C G L F T A I L C C N L I E N V Q R L G L T P T T V I R L N
Dr_mkks      L Q H H T T R Y S D S G L F M G I F T L T L I E N T K K Y G L R T S T A I K V Y
Ol_EN15281   V L N H I S R F S D C G L F A G V F C L S L L E Q A R R H E L R E S L A I K V N
Ga_EN05932   I L N H V S R F S D C G L F A A I L C L S L I E Q A K Q S G L S F S V A I K L N
Tr_SI175074  I L N H V G R F G D C G L F A A I F C L T L I E Q S R Q S G L R G R A A A Q L N

jnet
conf         8 7 3 5 3 2 0 1 5 8 7 5 6 8 9 9 9 9 9 9 9 9 9 9 9 8 7 4 4 8 8 8 6 6 8 9 9 9 9 9
```

```
                                        4     F
1A6D.A Sec Str description

                           130                 140                 150                 160
Xt_sca_8      R S L L N M C I S Y L S A D D C A C K V K V D F N S S K P L L C L I R S V V S S
Gg_MKKS       K H L L S L C T D Y L K S K A C G C R V S V D F S N L E T L L C L V R S I L T S
Md_31632      N H L L S L C T D Y L N S E T C G C R I S V D F S N M K T L H C L V Q S I L T S
Oa_0078571    K H L L S L C V E Y L R S E V C A C R I P V D F S R T R T L L Q L V R S I L T S
Mm_Mkks       K Y L L S L C T S Y L K S E A C S C R I P V D F R S T H T F L S L V H S I L T S
Rn_Mkks       K Y L L G L S I S Y L K S E A C S C R I P V D F R S T H T F L N L V H S I L T S
Bt_614288     K H L L S L C T S Y L K S E G C A C R I P V D F S S T Q I L L S L V R S I L T S
Cf_485771     Q H L L S L C T S Y L K S E S C G C R I L V D F S S P Q T L L C L V R S I V T S
Ec_64409      K H L L R L C I S Y L K S E V C G C R I P V D F S S T Q I L L C L V R S I L T S
Mmu_MKKS      K H L L S L C I S Y L K S E T C G C R I P V D F S S T Q I L L C L V R S I L T S
Pp_L0833      K H L L S L C I S Y L K S E T C G C R I P V D F G S T Q I L L C F V R S V L T S
Hs_MKKS       K H L L S L C I S Y L K S E T C G C R I P V D F S S T Q I L L C L V R S I L T S
Ptr_MKKS      K H L L S L C I S Y L K S E T C G C R I P V D F S S T Q I L L C L V R S I L T S
Dr_mkks       K H L V E Q C N V Y L K G D S C G C K V P V E F S S C D S L V A L A R S M I T S
Ol_EN15281    K H C L S L C T S Y L Q R D D C A C R V K I D F S S S Q N L M T L A G S I I S S
Ga_EN05932    K H L L G L C T G Y L Q Q E D C G C K V K L D F C S S H N L I T L A R S I I S S
Tr_SI175074   K H L L D L C T V Y L R Q E D C G C K M E L G F N S S H N L T T L A S S I I S S

lupas_28
jnet
              9 9 9 9 9 9 9 8 8 5 2 7 8 8 8 8 4 5 7 8 7 4 4 4 8 6 6 8 9 9 9 9 9 9 9 8 7 4 4 7
```

## N-TERMINAL EQUATORIAL DOMAIN

```
                                        G           5        6
1A6D.A Sec Str description

                           170                 180                 190                 200
Xt_sca_8      K P A C M L T T Q E A D Y I S T L I L K A F I C T I P D K S G P N I V L G K S V
Gg_MKKS       K P A C M L N K T E V D C L A V L I V K A F M Y T V P C H P E T K P V L G K C V
Md_31632      K P A C L L N K K E A D H I S T L I L K A F L L T I P E K T N D F A I L G K S L
Oa_0078571    K P A C M L T V R E V D H I G A L I L R A F L L T V P Q N A E G R A I L G K S I
Mm_Mkks       K P A C M L T R K E T D H I G A L I L K A F L L T I P E S T E E R M V L G K S I
Rn_Mkks       K P A C M L T R K E I D H I G A L I L K A F L L T I P E S A E E R M V L G K S I
Bt_614288     K P A C M L I A K E V D H I S T L I L R A F L L T I P E N A Q E H I I L G K S I
Cf_485771     K P A C M L T R E E R D H L S A L I L R A F L L T I P E N T K D R L I L G N S I
Ec_64409      K P A C M L T R K E I D H V S A L I L R T F L L T I P E N T E D H I I L G K S I
Mmu_MKKS      K P A C M L T R K E T E H V S A L I L R A F L L T I P E N A E G H I I L G K S L
Pp_L0833      K P A C M L T R K E T E H V S A L I L R A F L L T I P E N A E G H I I L G K S L
Hs_MKKS       K P A C M L T R K E T E H V S A L I L R A F L L T I P E N A E G H I I L G K S L
Ptr_MKKS      K P A C M L T R K E T E H V S A L I L R A F L L T I P E N A E G H I I L G K S L
Dr_mkks       K P A C M L D S R E M Q Q I S S L I T Q A F L Y S I P C N S S G T A C F G R T V
Ol_EN15281    K P A C L L T E A E K L H I S T M A V R A F L Q T I P C S S P G P V T L G Q T V
Ga_EN05932    K P A C V L T E S E A L H I S K L A V H A F L L T V P C N R P G E V R L G K A V
Tr_SI175074   K P A C V L T Q S E V L H M S K L S V Q A F L L T V P C S S T G   V K L G E T V

jnet
conf          8 8 8 7 7 6 5 6 8 9 9 9 9 9 9 9 9 9 9 9 8 7 6 2 1 5 7 7 7 8 8 8 5 5 8 9 8 9 8 9
```

## N-TERMINAL INTERMEDIATE DOMAIN

```
              7                 8    9
1A6D.A Sec Str description

                           210                 220                 230                 240
Xt_sca_8      I V P I E G H S V S E S S V V P G L L I E M P E F C W S R S V P S S G L P F A E
Gg_MKKS       T V P L K G K R V L D S T V I P G L L I E T P E I Q F A K P F S V K R T S S D A
Md_31632      I I P L K G E R V M D S T V L P G I V I E M P E V Q L M   T F P I K K L P S N A
Oa_0078571    I V P L K G H R V M D S T V L P G L L I E M P E F L S M K T L P D R T L P G R T
Mm_Mkks       I V P L K G Q R V T D S T V L P G L L I E A S E V Q L R R L L P T Q K   A S G
Rn_Mkks       I V P L K G Q Q V T D S T V L P G L L I E A S E V Q L R R L L P T Q K   S S T
Bt_614288     I V P L K G Q R V I D S A V L P G I L I E V S E V Q L M K I L P I K K   S D S
Cf_485771     I V P L K G Q R V L D S S V L P G I L I E M S E I Q L M K I L P I K K   S E A
Ec_64409      I V P L K G Q R V I D S T V L P G I L I E M S E I Q F V K L L P V K K   P G S
Mmu_MKKS      I V P L K D Q R V I D S T V L P G I L I E M S E V Q L M R L L P I K K   S T A
Pp_L0833      I V P L K G Q R V I D S T V L P G I L I E M S E V Q L M R L L P I K K   S T A
Hs_MKKS       I V P L K G Q R V I D S T V L P G I L I E M S E V Q L M R L L P I K K   S T A
Ptr_MKKS      I V P L K G Q R V I D S T V L P G I L I E M S E V Q L M R L L P I K K   S T A
Dr_mkks       T I G I E G Q S V N H S S V F P G L L L D V P E M L L P G D L E R L G   D G P
Ol_EN15281    T V T V R G L P V L D S A M F P G L L V E T H D D F N Q T D S S         S E L
Ga_EN05932    T V S V E G H P V L N S A V F P G L L V D A P D V S G I D E S E N M R   S N P
Tr_SI175074   T V P V E G P P V K H S A V F P G L L V D M P D L L S L D K A K R P H   L R P

jnet
conf          9 9 9 8 5 4 7 4 1 5 6 7 6 4 1 1 1 0 1 1 3 6 7 7 7 7 7 7 7 7 7 6 7 7 7 7 7 7 6 4
```

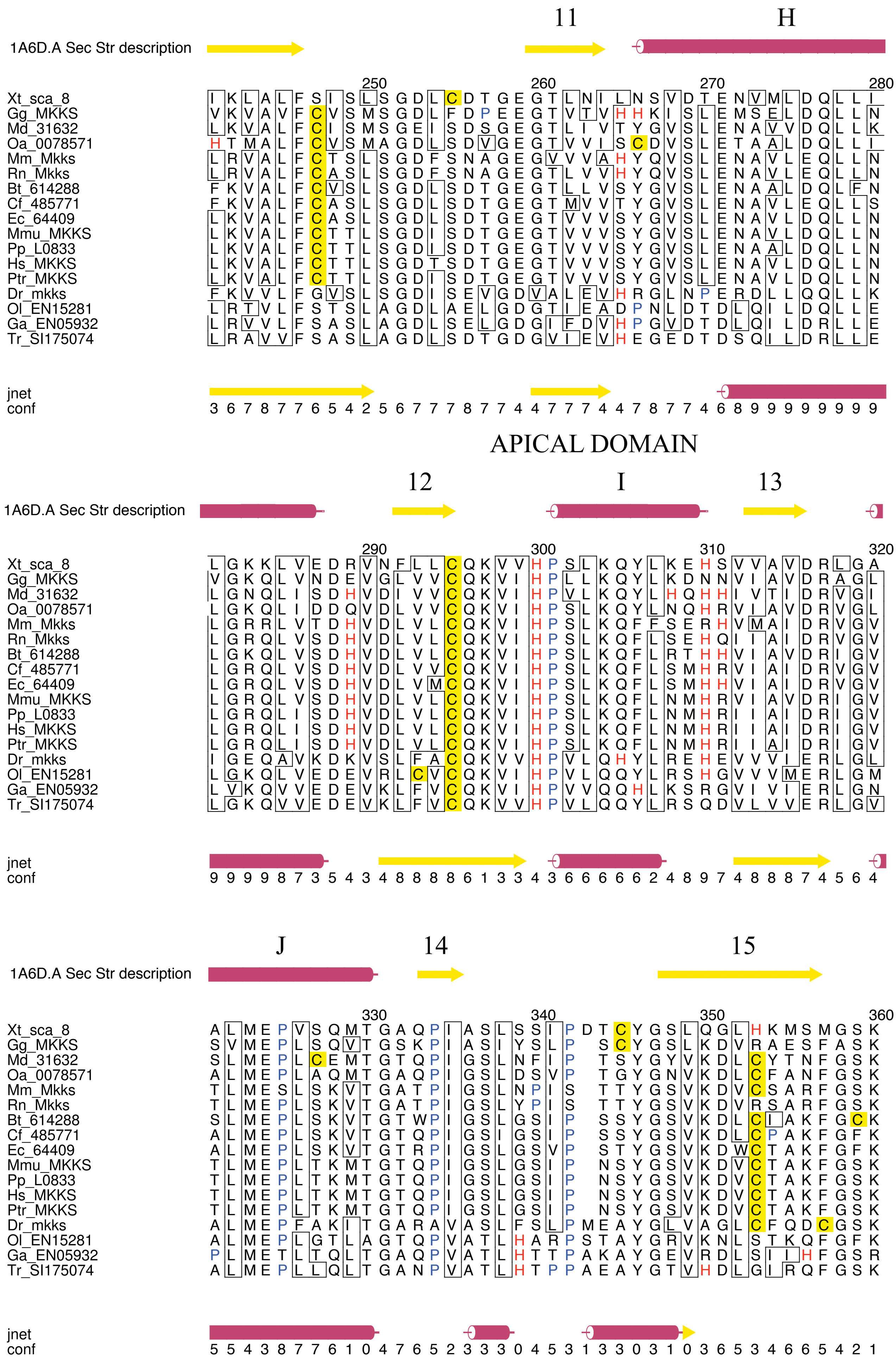
11
H
1A6D.A Sec Str description
250
260
270
280
Xt_sca_8
Gg_MKKS
Md_31632
Oa_0078571
Mm_Mkks
Rn_Mkks
Bt_614288
Cf_485771
Ec_64409
Mmu_MKKS
Pp_L0833
Hs_MKKS
Ptr_MKKS
Dr_mkks
Ol_EN15281
Ga_EN05932
Tr_SI175074
jnet
conf
APICAL DOMAIN
12
I
13
290
300
310
320
J
14
15
330
340
350
360

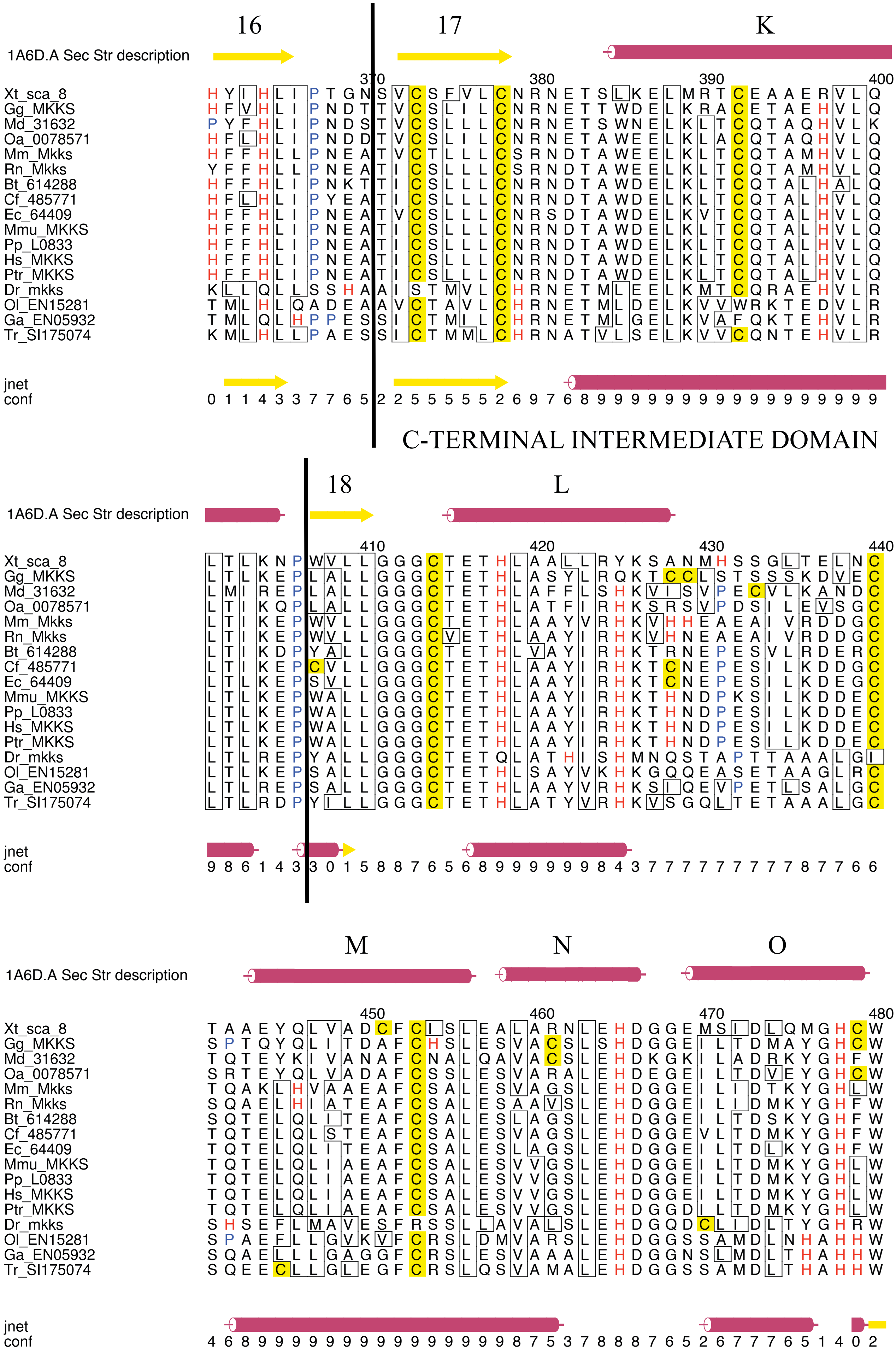
16
17
K
1A6D.A Sec Str description
370
380
390
400
Xt_sca_8 H Y I H L I P T G N S V C S F V L C N R N E T S L K E L M R T C E A A E R V L Q
Gg_MKKS H F V H L I P N D T T V C S L I L C N R N E T T W D E L K R A C E T A E H V L Q
Md_31632 P Y F H L I P N D S T V C S L L L C N R N E T S W N E L K L T C Q T A Q H V L K
Oa_0078571 H F L H L I P N D D T V C S L L L C N R N E T A W E E L K L A C Q T A Q H V L Q
Mm_Mkks H F F H L L P N E A T V C T L L L C S R N D T A W E E L K L T C Q T A M H V L Q
Rn_Mkks Y F F H L L P N E A T I C S L L L C S R N D T A W E E L K L T C Q T A M H V L Q
Bt_614288 H F F H L I P N K T T I C S L L L C N R N D T A W D E L K L T C Q T A L H A L Q
Cf_485771 H F L H L I P N E A T I C S L L L C N R N D T A W D E L K L T C Q T A L H V L Q
Ec_64409 H F F H L I P N E A T V C S L L L C N R S D T A W D E L K V T C Q T A L H V L Q
Mmu_MKKS H F F H L I P N E A T I C S L L L C N R N D T A W D E L K L T C Q T A L H V L Q
Pp_L0833 H F F H L I P N E A T I C S L L L C N R N D T A W D E L K L T C Q T A L H V L Q
Hs_MKKS H F F H L I P N E A T I C S L L L C N R N D T A W D E L K L T C Q T A L H V L Q
Ptr_MKKS H F F H L I P N E A T I C S L L L C N R N D T A W D E L K L T C Q T A L H V L Q
Dr_mkks K L L Q L L S S H A A I S T M V L C H R N E T M L E E L K M T C Q R A E H V L R
Ol_EN15281 T M L H L Q A D E A A V C T A V L C H R N E T M L D E L K V V W R K T E D V L R
Ga_EN05932 T M L Q L H P P E S S I C T M I L C H R N E T M L G E L K V A F Q K T E H V L R
Tr_SI175074 K M L H L L P A E S S I C T M M L C H R N A T V L S E L K V V C Q N T E H V L R
jnet
conf 0 1 1 4 3 3 7 7 6 5 2 2 5 5 5 5 5 2 6 9 7 6 8 9 9 9 9 9 9 9 9 9 9 9 9 9 9 9 9 9
C-TERMINAL INTERMEDIATE DOMAIN
18
L
1A6D.A Sec Str description
410
420
430
440
Xt_sca_8 L T L K N P W V L L G G G C T E T H L A A L L R Y K S A N M H S S G L T E L N C
Gg_MKKS L T L K E P L A L L G G G C T E T H L A S Y L R Q K T C C L S T S S S K D V E C
Md_31632 L M I R E P L A L L G G G C T E T H L A F F L S H K V I S V P E C V L K A N D C
Oa_0078571 L T I K Q P L A L L G G G C T E T H L A T F I R H K S R S V P D S I L E V S G C
Mm_Mkks L T I K E P W V L L G G G C T E T H L A A Y V R H K V H H E A E A I V R D D G C
Rn_Mkks L T I K E P W V L L G G G C V E T H L A A Y I R H K V H N E A E A I V R D D G C
Bt_614288 L T I K D P Y A L L G G G C T E T H L V A Y I R H K T R N E P E S V L R D E R C
Cf_485771 L T I K E P C V L L G G G C T E T H L A A Y I R H K T C N E P E S I L K D D G C
Ec_64409 L T L K E P S V L L G G G C T E T H L A A Y I R H K T C N E P E S I L K D E G C
Mmu_MKKS L T L K E P W A L L G G G C T E T H L A A Y I R H K T H N D P K S I L K D D E C
Pp_L0833 L T L K E P W A L L G G G C T E T H L A A Y I R H K T H N D P E S I L K D D E C
Hs_MKKS L T L K E P W A L L G G G C T E T H L A A Y I R H K T H N D P E S I L K D D E C
Ptr_MKKS L T L K E P W A L L G G G C T E T H L A A Y I R H K T H N D P E S I L K D D E C
Dr_mkks L T L R E P Y A L L G G G C T E T Q L A T H I S H M N Q S T A P T T A A A L G I
Ol_EN15281 L T L K E P S A L L G G G C T E T H L S A Y V K H K G Q Q E A S E T A A G L R C
Ga_EN05932 L T L R E P S A L L G G G C T E T H L A A Y V R H K S I Q E V P E T L S A L G C
Tr_SI175074 L T L R D P Y I L L G G G C T E T H L A T Y V R H K V S G Q L T E T A A A L G C
jnet
conf 9 8 6 1 4 3 3 0 1 5 8 8 7 6 5 6 8 9 9 9 9 9 8 4 3 7 7 7 7 7 7 7 7 7 8 7 7 6 6
M
N
O
1A6D.A Sec Str description
450
460
470
480
Xt_sca_8 T A A E Y Q L V A D C F C I S L E A L A R N L E H D G G E M S I D L Q M G H C W
Gg_MKKS S P T Q Y Q L I T D A F C H S L E S V A C S L S H D G G E I L T D M A Y G H C W
Md_31632 T Q T E Y K I V A N A F C N A L Q A V A C S L E H D K G K I L A D R K Y G H F W
Oa_0078571 S R T E Y Q L V A D A F C S S L E S V A R A L E H D E G E I L T D V E Y G H C W
Mm_Mkks T Q A K L H V A A E A F C S A L E S V A G S L E H D G G E I L I D T K Y G H L W
Rn_Mkks S Q A E L H I A T E A F C S A L E S A A V S L E H D G G E I L I D M K Y G H F W
Bt_614288 S Q T E L Q L I T E A F C S A L E S L A G S L E H D G G E I L T D S K Y G H F W
Cf_485771 T Q T E L Q L S T E A F C S A L E S V A G S L E H D G G E V L T D M K Y G H F W
Ec_64409 T Q T E L Q L I T E A F C S A L E S L A G S L E H D G G E I L T D L K Y G H F W
Mmu_MKKS T Q T E L Q L I A E A F C S A L E S V V G S L E H D G G E I L T D M K Y G H L W
Pp_L0833 T Q T E L Q L I A E A F C S A L E S V V G S L E H D G G E I L T D M K Y G H L W
Hs_MKKS T Q T E L Q L I A E A F C S A L E S V V G S L E H D G G E I L T D M K Y G H L W
Ptr_MKKS T Q T E L Q L I A E A F C S A L E S V V G S L E H D G G D I L T D M K Y G H L W
Dr_mkks S H S E F L M A V E S F R S S L L A V A L S L E H D G Q D C L I D L T Y G H R W
Ol_EN15281 S P A E F L L G V K V F C R S L D M V A R S L E H D G G S S A M D L N H A H H W
Ga_EN05932 S Q A E L L L G A G G F C R S L E S V A A A L E H D G G N S L M D L T H A H H W
Tr_SI175074 S Q E E C L L G L E G F C R S L Q S V A M A L E H D G G S S A M D L T H A H H W
jnet
conf 4 6 8 9 9 9 9 9 9 9 9 9 9 9 9 9 9 9 8 7 5 3 7 8 8 8 7 6 5 2 6 7 7 7 6 5 1 4 0 2

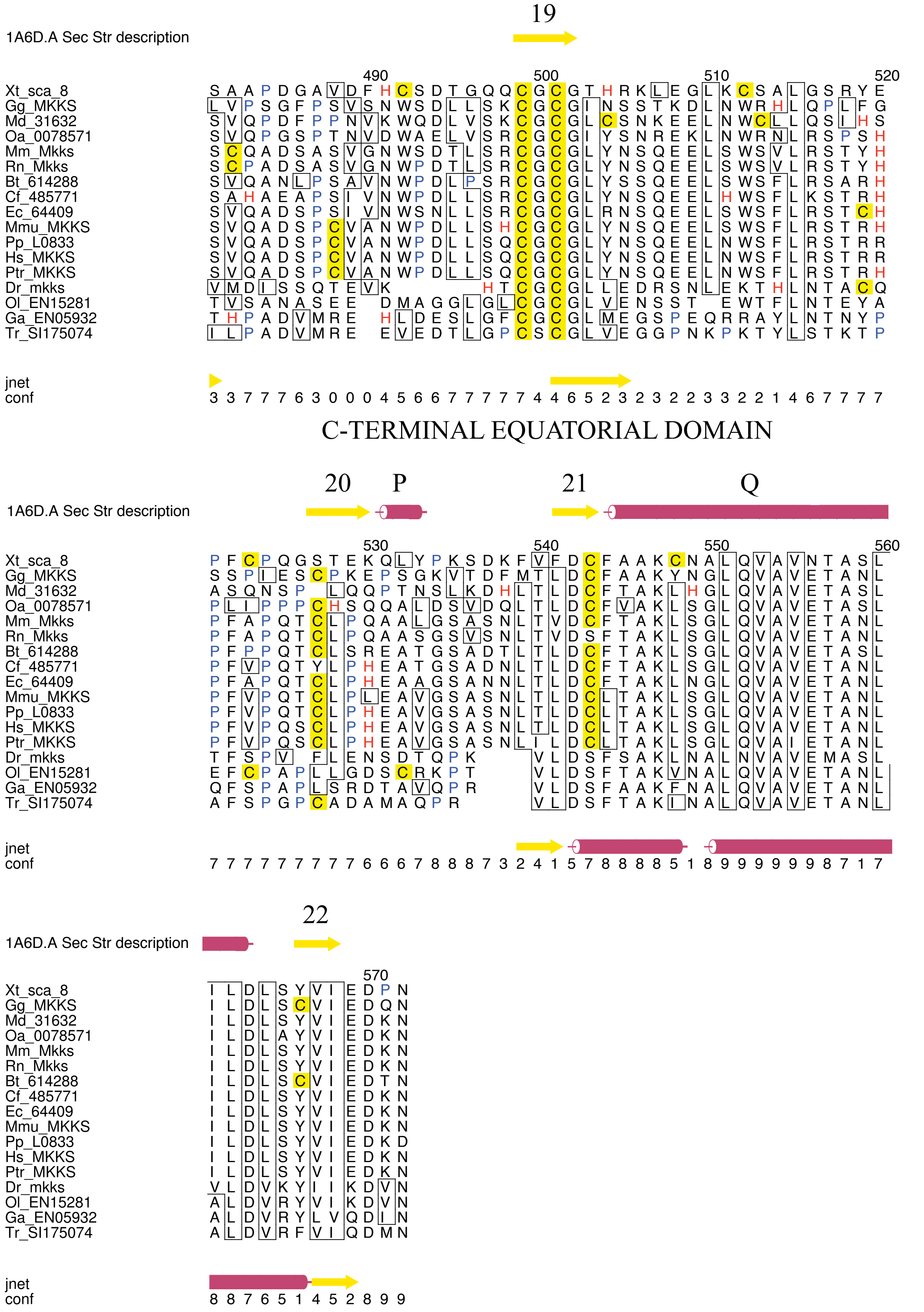


Supplementary figure S13. Alignment and secondary-structure predictions of MKKS sequences compared to PDB secondary-structure description of 1a6d. See Legend for Supplementary figure S10 for symbols and Legend for Figure 2 for species abbreviations.
