# Supplementary material for: Chaperonin genes on the rise: new divergent classes and intense duplication in human and other vertebrate genomes
Source: BMC Evol Biol. 2010 Mar 1;10:64. doi: 10.1186/1471-2148-10-64 (PMC2846930; doi:10.1186/1471-2148-10-64)
Supplement: Additional file 19 — Table S14. Alignment and secondary-structure prediction of vertebrate BBS10 protein sequences. [file 1471-2148-10-64-S19.PDF]

```
                          1            A                        2
1A6D.A Sec Str description

               1                   10                  20                  30                  40
Xl_84945       K K Y P L A V D V S K V L Q V A E S L E N I V C R C F G P D G G H V L F I K S T
Md_BBS10       M A A A T A G G V G V A L Q A A E V L E S I L S G S V G P E G R Q V L C T K P T
Mm_Bbs10           M A S Q G S V T A A L R V A E V L E S I A N R C V G P E G G Q V L C T K P T
Rn_60748           M A S Q G S V T A A L R V A E V L E T I A N R C V G P E G G L V L C T K P T
Mmu_93270      S S M A A T G S L K A A L Q V A E V L E A I V S C C M G P E G R Q V L C T K P T
Hs_BBS10       S S M A A A G S V K A A L Q V A E V L E A I V S C C V G P E G R Q V L C T K P T
Ptr_BBS10      S S M A A A G S V K A A L Q V A E V L E A I V S C C V G P E G R Q V L C T K P T
Pp_BBS10       S S M V A A G S V K A A L Q V A E V L E A I V S C C V G P E G R Q V L C T K P T
Cf_BBS10           M A A T G S V E A A L Q V A E A L E T I V S R C L G P E G R Q V L C T K P T
Bt_538638          M A A A G S V T V A L H V A E V L E T I V S G C L G P E G R Q V L C T K P T
Ec_58970           M A A A A S V K A A L Q V A E A L E S I V S C C V G P D G R Q V L C T K P T
Tn_35588       M V P V E H L H L D H V A Q I V C V L E S V I L R C F G P D G G Q V L F I R D T
Tr_sca_225     M V P V E H L H L D H V Q Q I V C V L E S V I L R C F G P D G G Q V L F I R D T
Ga_EN17383     M L P V E H L H L E H V L Q T V C V L E S V V C R S F G P E G G Q V L F T Q D T
Ol_C6_09334    M P P S E R L P L E Q V L E I V S A L E A V V L R S F G P E G G Q V L F T R D T

jnet
conf           9 9 9 8 8 7 6 4 6 8 9 9 9 9 9 9 9 9 9 9 9 8 7 5 3 7 8 8 8 8 7 4 4 8 8 8 7 2 7 6
```

```
                 3        B                  C
1A6D.A Sec Str description

                                   50                  60                70                    80
Xl_84945       G D L L I T R D G R K I L E S L L L D H P I G R I I V H S A C N H A S I T G D G
Md_BBS10       G D V L F S R D G G R L L E A L N V E H P V A R M I V T C V S M N Q N V T G D G
Mm_Bbs10       G E V L L S R D G G C L L E A L H L E H P L A R M I V A C V S S H L K K T G D G
Rn_60748       G E V L L S R D G G C L L E A L H L E H P L A R M I V A C V S S H L K K T G D G
Mmu_93270      G E V L L S R N G G R L L E A L H L Q H P I A R M I V D C V S S H L K K T G D G
Hs_BBS10       G E V L L S R N G G R L L E A L H L E H P I A R M I V D C V S S H L K K T G D G
Ptr_BBS10      G E V L L S R N G G R L L E A L H L E H P I A R M I V D C V S S H L K K T G D G
Pp_BBS10       G E V L L S R N G G R L L E A L H L E H P I A R M I V D C V S S H L K K T G D G
Cf_BBS10       G E V L L S R D G G R L L G A L H L E H P A A R L V V A Y V S S H L R K T G D G
Bt_538638      G E V L L S R D G G C L L K A L H L E H P V A R V M V A C V S S H L R K T G D G
Ec_58970       G E V L L S R D G G R L L Q A L H L E H P I A R M M V A C V S N H L R K A G D G
Tn_35588       G Q A M L S R T G S Q I L S A L R L E H P L A R V V V D C V L K H S A A T G D G
Tr_sca_225     G Q A M I S R S G S R I L S A L R L E H P L A R M V V D C V L K H S T A T G D G
Ga_EN17383     G Q A M L S R S G A R I L T A L R L E H P L A R M V V E C V L K H S T E T G D G
Ol_C6_09334    G Q A M L S R S G T C I L S A L H L E H P L A R V V V E S V W K H S R G T G D G

jnet
conf           3 0 6 7 5 3 4 6 5 6 5 4 4 3 4 7 8 8 7 7 5 6 8 9 9 9 9 9 9 9 8 7 4 4 7 8 8 7 6 5
```

N-TERMINAL EQUATORIAL DOMAIN

```
                          D                             E
1A6D.A Sec Str description

                                   90                  100                 110                 120
Xl_84945       V K S F V V L L C G V L R G L Q A A V N K S G G L T G K Q S N P N Q G H V F K R
Md_BBS10       A K T F I I L L C D L L R G L K A F T E K G G S I P S Q E R H W K I C C Q W K Y
Mm_Bbs10       A K T F I I F L C H L L R G L H A I G E K S E N I Q S H E R H W K N C C Q W K S
Rn_60748       A K T F I I F L C H L L R G L H A I G E K S E D I Q S H E R H W K N C C Q W K S
Mmu_93270      A K T F I I F L C H L L R G L H A V T D R C E N I Q T H G R H W K N C C Q W K F
Hs_BBS10       A K T F I I F L C H L L R G L H A I T D R C E N I Q T H G R H W K N C S R W K F
Ptr_BBS10      A K T F I I F L C H L L R G L H A I T D R C E N I Q T Q G R H W K N C S R W K F
Pp_BBS10       A K T F I I F L C H L L R G L H A I T D S C E N I Q T H G R H W K N C S Q W K C
Cf_BBS10       A K T F V V F L C H L L R G L H A M T D G S K H M Q T H R R H W K N C C R W K L
Bt_538638      A K T F I I F L C H L L R G L R E I T D K F E N I Q T C G R H W K N C C R W K C
Ec_58970       A K T F I I F L S H L L R G L H A I P D Q S E N S Q T L G R H W K N C C R W K F
Tn_35588       S K T F V L L L A S L L R T I H A A A C K H A S H T Y M T R Y S A E A A T A R H
Tr_sca_225     S K T F V L L L A S L L R T I H A S A C K N V S H N Y V S R Y S A E A A T G R H
Ga_EN17383     S K T F I I L M A S L L R I I H T T A C R H V S R T H T S K A A A E T A S A R H
Ol_C6_09334    S K T F I L L L A S L L R G I S K A T S R S A S H A Y G F T E A A K A A A A P Y

jnet
conf           0 1 8 9 9 9 9 9 9 9 9 9 9 9 9 9 9 8 7 4 3 7 7 8 8 8 7 7 6 6 4 6 8 9 9 9 9 9 9 9
```

```
                                   E                         4        F
1A6D.A Sec Str description
                                         130       140       150       160
Xl_84945      L S N L I M T F Q T E I L E N I I V K Q L S P H F E S F L K E T N T L S G G T I
Md_BBS10      I S Q A I L T F Q A H I L D Y I M T Q Y L R K H F L S F S G E E K K V C R S S L
Mm_Bbs10      I S Q A L Q T F Q T Q T L G C I V D R S L S R H Y L S S S T E G R K L C R H S L
Rn_60748      I S R A L L R F Q T Q T L G C I V D Q H L S R H Y L S S S A E G R T L C R R S L
Mmu_93270     I S Q A L V T F Q T Q I L D G I M D Q Y L S R H F L S S S A K E R T L C R S S L
Hs_BBS10      I S Q A L L T F Q T Q I L D G I M D Q Y L S R H F L S S S A K E R T L C R S S L
Ptr_BBS10     I S Q A L L T F Q T Q I L D G I M D Q Y L S R H F L S S S A K E R T L C R S S L
Pp_BBS10      I S Q A L L T F Q T Q I L D G I M D Q Y L S R H F L S S S A K E K T L C R S S L
Cf_BBS10      I S Q F L L T F Q T H R L D Y I V D H Y L S R H F G S S S T K E R T L C R S S L
Bt_538638     I S Q A F L T F Q T Q I L D Y V M D H Y L S R H F L S S C T K E R T L C R S S L
Ec_58970      I S Q A L L T F Q T Q I L D Y V M D E Y L S R H F L S S S T K E R T L C R N S L
Tn_35588      L A Y K L L E F A S G Q L E D L I A T A V S P Y G R S T A Q L Q P H T D S H C V
Tr_sca_225    L A F K T L E F A S E Q L E N L I T A A V I P Y G C S T A H L Q T G A G S R H V
Ga_EN17383    L A D K M L A F A S E E L D D L I A A A V V P Y G C C S A K S Q A H S N N P C V
Ol_C6_09334   F A N K L L E L V S S D L D D L I A V N V V P Y G F C             E D F T T H S G

jnet
conf          9 9 9 9 9 9 8 7 5 1 2 7 6 7 9 9 9 9 9 7 4 4 7 1 1 1 1 3 6 6 6 5 3 4 3 7 9 9
```

```
                                                    G                    5
1A6D.A Sec Str description
                                         170       180       190       200
Xl_84945      Q S V L D T Y F S G R I G Y N N Q A F I S S L V A D Y F H       K C L P Y N K
Md_BBS10      E S L L D A Y F C G R V G R N N Q K F I S Q L T C D Y F Y       K C L Q H E D
Mm_Bbs10      E L L L E A Y F C G R V G R N N H R F I S Q L M C D Y V F       K C M A C E S
Rn_60748      E L L L E P Y F C G R V G R N N H R F I S Q L M C D Y V F       K C M A C E S
Mmu_93270     E L L L E A Y F C G R V G R N N H K F I S Q L M C D Y F F       K C M T C K T
Hs_BBS10      E L L L E A Y F C G R V G R N N H K F I S Q L M C D Y F F       K C M T C K S
Ptr_BBS10     E L L L E A Y F C G R V G R N N H K F I S Q L M C D Y F F       K C M T C K S
Pp_BBS10      E L L L E A Y F C G R V G R N N H K F I S Q L M C D Y F F       K C M T C E S
Cf_BBS10      E S L L A A Y F G G R V G R N N R D F M S R L T C D Y F F       K C M A R E R
Bt_538638     E M L L G A Y F C G R V G R N N Q N L M S Q L M C D Y V F       K C T A C E S
Ec_58970      E L L L E A Y F C G R V G R N N H N L I S Q L M C D Y L F       K C V A R E G
Tn_35588      Q R L L A S F F R S R L S R S Q G H F A T N L A C D L L T       S C Q F K G A
Tr_sca_225    Q K L L A S F F C S R L S R S Q C D F A T S L T C N L L S       S C G F K G A
Ga_EN17383    Q N L L A S F F Q S R V G P T H C D L M S N L T C E L L T       N W K F K N D
Ol_C6_09334   R L L L A S F F H T R V S H V H C S Y L S E L T C E M L N       R W K Y K S D

jnet
conf          9 9 9 9 9 9 7 4 4 7 8 8 7 7 6 5 6 8 9 9 9 9 9 9 9 9 9 8 7 4 3 7 8 8 8 8 7 6 6
```

N-TERMINAL INTERMEDIATE DOMAIN

```
                 6               7                 8     9
1A6D.A Sec Str description
                                         210       220       230       240
Xl_84945      C I D D V V H T V N T C F S E L H T E V A G E P I E N S R I L S G I V L H R Q F
Md_BBS10      G K D E M I D L V D E Y F L E L C T A V T G L P V S S S K I I S G F V I H R D F
Mm_Bbs10      G V   E V F E L L D H C F A E L N V G V T G L P V S D S R I I D G L V L P R D F
Rn_60748      G I   E V F E F L D N C F V E L N V G V T G L P V S E S R I V D G L V L P R D F
Mmu_93270     G I   D V F E L V D D Y F V E L N V G V T G L P I S D S R I I A G L V L Q K D F
Hs_BBS10      G I   G V F E L V D D H F V E L N V G V T G L P V S D S R I I A G L V L Q K D F
Ptr_BBS10     G I   G V F E L V D D H F V E L N V G V T G L P V S D S R I I A G L V L Q K D F
Pp_BBS10      G I   G V F E L V D D Y F V E L N V G V T G L P V S D S R I I A G L V L Q K D F
Cf_BBS10      G S E E V L D L V D D Y F V E L S V G V T G L P V S D S R I T P G L V L P R D F
Bt_538638     R F E E V L E L V D D C F V E L K V G V T G L P V S D S R I I A G L V L Q R D F
Ec_58970      G F E E V F E L V G D C F V E L N V G V T G L P V S D S R I V A G L V L H R D F
Tn_35588      Q S L S S L Q F L T D N F P V M H T C V S G F P F T C S R L V E G Q V I H R D F
Tr_sca_225    Q P S S L L Q F L T D N F P T L H T R V S G F P F T C S R L V E G Q V I H R D F
Ga_EN17383    S P S S S L H F I R D N L A A L H T P V S G F P I S F S R L V E G Q V I H R D F
Ol_C6_09334   L P Y I S F Q F L S D N F P A L H T P V S G F P V S S S R L I E G Q V I H R D F

jnet
conf          4 6 8 9 9 9 9 9 9 8 5 5 7 3 5 8 8 8 9 8 9 8 5 4 8 8 8 8 4 5 8 8 8 7 5 2 6 5 3 4
```

```
                              10        10                          11
1A6D.A Sec Str description

                                        250                 260                 270                 280
Xl_84945      A V Y C P A E G       E I R A V I I T D Q I H Q Y L S A S D V E F A V C S D A Q
Md_BBS10      S V Y C P A D G       D M R I I I V T E P I Q P A L S S S G S E L L I N S E A Q
Mm_Bbs10      S M Y C P A D G       D I R M V I V T E I L Q P Q F S S A G S E F V L N S E T Q
Rn_60748      S V Y C P A D G       G I R M V I V T E I L Q P L F S S S S S E F V L D S E T Q
Mmu_93270     S V Y C P A D G       D I R M V I V T E T I Q P L F S T S G S E F I L N S E A Q
Hs_BBS10      S V Y R P A D G       D M R M V I V T E T I Q P L F S T S G S E F I L N S E A Q
Ptr_BBS10     S V Y R P A D G       D M R M V I V T E T I Q P L F S T S G S E F I L N S E A Q
Pp_BBS10      S V Y C P A D G       D I R M V I V T E T V Q P L F S T S G S E F I L N S E A Q
Cf_BBS10      S V Y R P A D G       D I R I V I V T E T V Q P L F S T S G S E F I L R S E A Q
Bt_538638     S V Y C P A D G       D I R I V I V T E T I Q P L F S I S G S E F T L N S E A Q
Ec_58970      S G Y C P A D G       D I R I A L V T E T I Q P I F S T S G S E F I L N S E A Q
Tn_35588      A T P C P P T G S D L P V R A V V F T G D L E P E I L T A G E V L D L C G Q T N
Tr_sca_225    A S F C P P N V S D L P V K A V I F T G G L E P E I L T S G E V L E L C G Q T S
Ga_EN17383    A A P C P R A D L Q   P V K A V V L T G Y L Q P K L L R A G E V L E L S E E R N
Ol_C6_09334   A T P C L P S K Q Q   P V K V V V F T D Y M Q P I L L S T G D V L K L T P E S S

jnet
conf          5 6 7 6 6 6 7 7 7 8 7 4 4 8 8 8 7 4 3 7 7 7 7 6 6 7 7 7 7 7 3 4 6 6 4 4 7 7 7 4
```

```
                                 H                          12            I
1A6D.A Sec Str description

                                        290                 300                 310                 320
Xl_84945      L H L S Q M Y L R Q R T E K L M K H F Q N M E V R L I L S T I K Q P E I V L F Y
Md_BBS10      F Q A S H I W I T E K T K T I M K D L Q S K G I K L L L S S S V K Q Q E T V I Y Y
Mm_Bbs10      F Q A S Q C W I T D R T K T V M N H L R G Q N V K L L L T S V K Q P D L V I Y C
Rn_60748      F Q A S Q S W I M D R T K T V M N H L R S H N V K L L L S S V K Q P D L V T Y C
Mmu_93270     F Q T S Q F W I M E K T K A I M K H L R S Q N V K L L L S S V K Q P D L V I Y Y
Hs_BBS10      F Q T S Q F W I M E K T K A I M K H L H S Q N V K L L I S S V K Q P D L V S Y Y
Ptr_BBS10     F Q T S Q F W I M E K T K A I M K H L H S Q N V K L L I S S V K Q P D L V I Y Y
Pp_BBS10      F Q T S Q F W I M E K T Q A I M K H L H S Q N V K L L I S S V K Q P D L V I Y Y
Cf_BBS10      F Q T S R F W I M E R T K A I M K H L Q S Q N V K L L L S S V K Q P D S V I Y Y
Bt_538638     F Q T S Q F W I T E R T K A I V K H L Q N Q N V K L L L S T V K Q P D L V I Y C
Ec_58970      F R A S Q F W I M E R T K A I M K H L Q S Q N V K L L L S S V K Q P D S V I F Y
Tn_35588      I V K F K A W A E R S L R C I F A N L Q R L D I S L L L S A V K Q S P A V L S L
Tr_sca_225    I V D F K A W A E R S L S C V F A N L Q R L G I S L L L S A V K Q S P A V L S L
Ga_EN17383    I M D F S A W G E R S L E C V L A N L Q S L G V S V L L S A V K Q S A A V L A L
Ol_C6_09334   I V H F S A W T E R S L E S V I A E L Q S L G V S L L L S A V K Q S D A A V A L

jnet
conf          7 8 9 9 9 9 9 9 9 9 9 9 9 9 9 9 9 9 9 8 5 4 9 7 2 7 7 7 6 2 1 6 5 7 8 9 9 9 9 9
```

APICAL DOMAIN

```
                                13             J                  14
1A6D.A Sec Str description

                                        330                 340                 350                 360
Xl_84945      A K Q N G I S V V D C L P T E E I E L V C L I T G V S P L S G D E F S G Q P L D
Md_BBS10      A K Q N G I S V V E Y I P S E E I S L L C R I I S L S P F M W A T S V C H I S D
Mm_Bbs10      A R L N S I S V V E C L S A E E V S L V Q R I T G L S P C V E V A S Q C E I S D
Rn_60748      A R L N S I S V V E C L S S E E V S L V Q R I T G L S P C V E L A S Q C H I A D
Mmu_93270     A G V N G I S V V E C L S S E E V S L I R R I I G L S P F V Q A F S Q C E I S N
Hs_BBS10      A G V N G I S V V E C L S S E E V S L I R R I I G L S P F V Q A F S Q C E I P N
Ptr_BBS10     A G V N G I S V V E C L S S E E V S L I R R I I G L S P F V Q A F S Q C E I P N
Pp_BBS10      A G V N G I S V V E C L S S E E V S L I R R I I G L S P F V Q A F S Q C E I P N
Cf_BBS10      S G L A G I S V V E C L S P E E V S L I R R I T G L S P L A H A S S Q D E I C N
Bt_538638     A G L N G I S V V E C L S S E E L S L I Q R V I G L A P F V E A S S Q Y E L S H
Ec_58970      A R L N D I S V V E C L S S E E V S L I Q R V I D L S P C V Q A S S R C E I S N
Tn_35588      A A Q A N I C V V E C V S K D D L A L F T Q L S Q A Q A V   S D C Q I I G S K N
Tr_sca_225    A A Q A N I C I V E C V S E D E L T L F A Q L S R T Q P V   S D C Q I I G P S N
Ga_EN17383    A T Q A E M S I V E C V S E D E L S L F Q R L S G A T P V   R D C R V I E P E H
Ol_C6_09334   A T Q A H M S L V E C V S E E E L S L F V Q L S G V T P V   S R G C T I Q L E H

jnet
conf          9 8 4 5 8 7 4 7 7 5 4 7 5 1 1 8 9 9 9 9 9 9 8 5 2 7 8 7 4 1 4 6 6 6 7 8 7 7 6 3
```

```
                              15                  16                  17
1A6D.A Sec Str description

                                      370                     380                     390                     400
Xl_84945       S F L I T C C Q P I L L G P R K Y V Q L T F S A T F L P H S V V I C G P V K G L
Md_BBS10       T A L V T F C R P I L L N S R R Y V H L G L L S S F I P H C L I L C G P V Q G L
Mm_Bbs10       S T L V K F C K P L I L R S K R Y V H L G L I S A F I P H S M V L C G P V L G L
Rn_60748       S A L V R F C K P L I V R S G R Y A H L G L V S A F V P H C V V L C G P V L G L
Mmu_93270      T A L V K F C K P L I L R S K R Y V H L G L I G T F I P H C V V L C G P V Q G L
Hs_BBS10       T A L V K F C K P L I L R S K R Y V H L G L I S A F I P H S I V L C G P V Q G L
Ptr_BBS10      T A L V K F C K P L I L R S K R Y V H L G L I S A F I P H S I V L C G P V Q G L
Pp_BBS10       T A L V K F C K P L I L R S K R Y V H L G L I S A F I P H S I V L C G P L Q G L
Cf_BBS10       T V L V K F C K P L T F R S K R Y V H L G V I S S F I P H C V V L C G P V Q G L
Bt_538638      T A L A K F C K P L I L R S K R F V H L G L M S S F A P H C I A L C A P V Q G L
Ec_58970       T A V V K F C K P L V L R S K R Y V H L G L I S S F A P H C L V L C G P V Q G L
Tn_35588       I A T L T F C Q P I H L G A H R Y V H L D F P D T V K P Y N L V I C G P G E G Q
Tr_sca_225     V A T L T F C R P I H L G P H R Y V H L A F Q D M V K L W N L V I C G P G E G Q
Ga_EN17383     V A T L T F C R P I L L G A H R Y V H V A F H D T V K P C S L V L C G L G E G Q
Ol_C6_09334    V A A L S F C R P I L L G G H R Y V Q V A F C E K V N P C S L V I C G P G E G Q

jnet
conf           3 6 5 4 3 2 3 6 5 4 3 4 7 8 7 4 4 8 8 8 8 5 3 7 8 8 8 7 4 5 7 7 7 4 4 7 7 7 5 7
```

C-TERMINAL

```
                                        K
1A6D.A Sec Str description

                                      410                     420                     430                     440
Xl_84945       T E Q L V S A L H G A F K M L Q L F Q P V V T N W I H T E K N E G Y C K A R Q K
Md_BBS10       T D Q H I R A F H G A F K V L R V F K V L Y L S Y K V Q R S N Q N E T S N S Q S
Mm_Bbs10       V E Q H E R A F H G A F K M L R L F T D L D L N Y I I Q T K Q Q C N P S P L Q T
Rn_60748       V Q Q H E S A F H G A F K M L R L F T D L D L N C M I Q T K E R R N P S P L Q T
Mmu_93270      I E Q H E D A L H G A F K M L R L F K D L D L N Y M T Q T S D Q N G T S S L Q T
Hs_BBS10       I E Q H E D A L H G A L K M L R L F K D L D L N Y M T Q T N D Q N G T S S L Q T
Ptr_BBS10      I E Q H E D A L H G A L K M L R L F K D L D L N Y M T Q T N D Q N G T S S L Q T
Pp_BBS10       I E Q H E D A L H G A F K M L R L F K D L D L S Y I T Q T N D Q N G T S S L Q T
Cf_BBS10       V E Q H R D A L H G A F K M L R L L K D L D L N Y L I Q T K V V N G S I Q R Q T
Bt_538638      V E Q H E N A L H G A F K M L R A F K D L D L N C V T Q T S D Q S C T S G P Q T
Ec_58970       V E Q H K D A L H G A F K M L R L F K H L D L N Y L T Q A S D Q N Y T S S P Q T
Tn_35588       T D Q Y A S A I H D A L H M L L A C R P L A I T A A K A
Tr_sca_225     T D Q Y A S A I L D A I L M L L A W Q P L A N T S A K A T E K T L N D E A S
Ga_EN17383     T D Q F A C A L K D A M R M L L T W D P I G I T A A P
Ol_C6_09334    T D Q Y I C A I R D A V C M L M T W E P K H T T N K P Y

jnet
conf           8 9 9 9 9 9 9 9 9 9 9 9 9 8 7 5 0 4 2 5 6 6 6 5 3 0 0 0 3 5 6 7 7 7 7 7 7 7 7 6
```

INTERMEDIATE DOMAIN

```
                                      450                     460                     470                     480
Xl_84945       D S V C E C N L H R I S F N Q K L E S I K C G V H S R I I N G             D I V
Md_BBS10       H L R D A P D L V L S S M E S K N D   I P S T T L T Q T P K I K E D F E S D P S
Mm_Bbs10       H L E V Y S G L G A S D T E L R A G   K P W S A H K K T P I A P S Q T D E C T C
Rn_60748       R L E V Y P R L G A S D T E L I T C   K L W S A H K E T S I D P S Q T N E C P S
Mmu_93270      Y L K V H S N L V I S D V E L E T Y   I P Y S         T P T D T F E T D E G A S
Hs_BBS10       Y L K V H S N L V I P D V E L E T Y   I P Y S T P T L T P T D T F Q T V E G T S
Ptr_BBS10      Y L K V H S N L V I P D V E L E T Y   I P Y S T P T L T P T D R F Q T V E G T S
Pp_BBS10       Y L K V H S N L V I S D V E L E T Y   I P Y S T P T L T P T D T F Q T V E G T S
Cf_BBS10       Y         S D L V V P S V E L E T C I I P C S A P K V T P T G T Y Q T D E C A A
Bt_538638      N L K V C S N L I V P S V K L E Q N   V L C S T P K V A V T D S Y Q T D E C T S
Ec_58970       Y L K V Y S N L V V P S I E L E K H   F P C S T P K V T P M D T Y Q T N E C P S
Tn_35588
Tr_sca_225                                                                           T C F
Ga_EN17383
Ol_C6_09334

jnet
conf           6 7 7 7 7 7 6 3 1 2 3 6 6 6 7 7 7 7 7 7 7 7 7 7 7 7 7 7 7 7 7 7 7 7 7 7 7 7 7 7
```

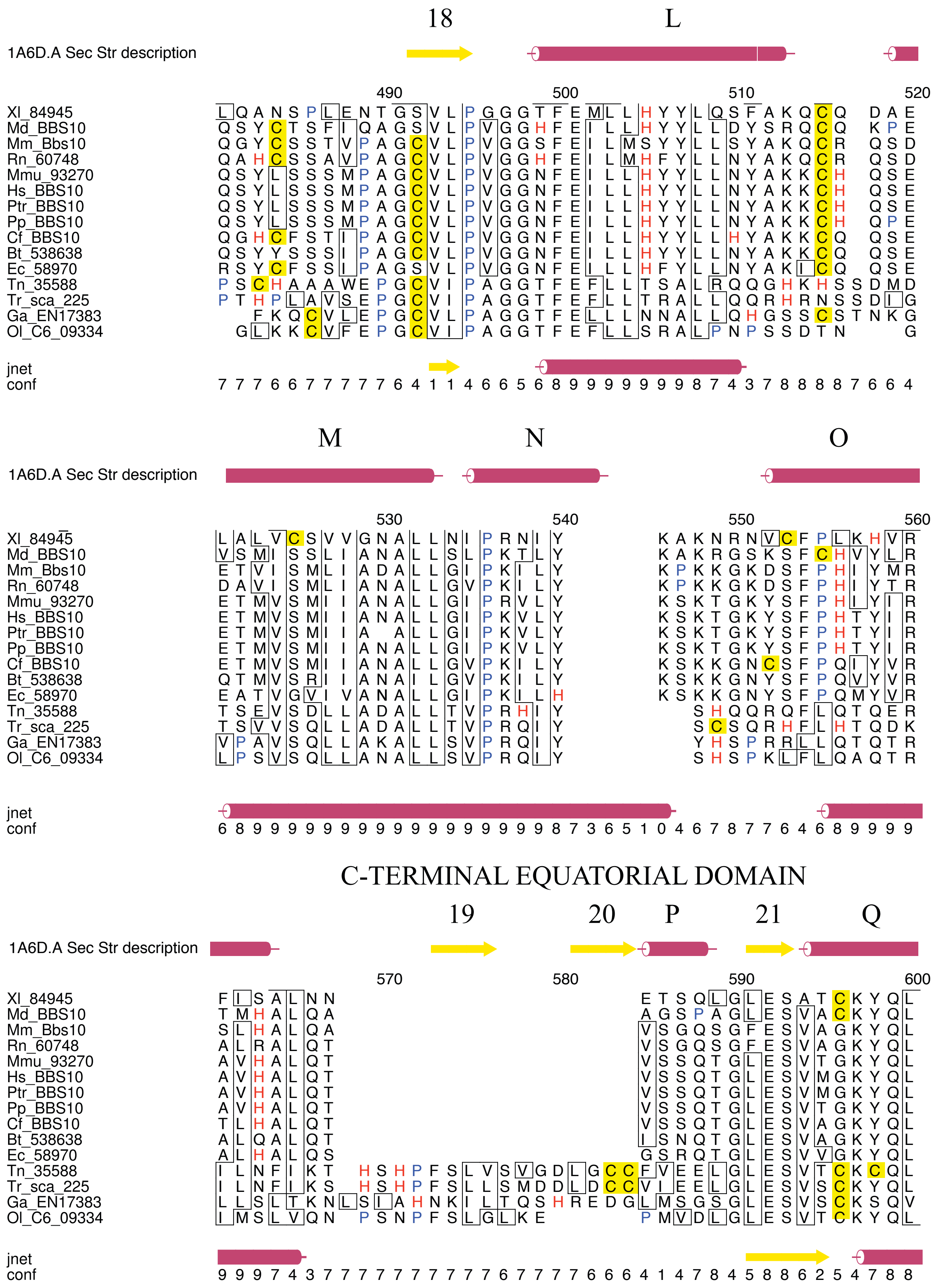

Supplementary figure S14. Alignment and secondary-structure predictions of BBS10 sequences compared to PDB secondary-structure description of 1a6d. See Legend for Supplementary figure S10 for symbols and Legend for Figure 2 for species abbreviations.
