# Supplementary material for: Chaperonin genes on the rise: new divergent classes and intense duplication in human and other vertebrate genomes
Source: BMC Evol Biol. 2010 Mar 1;10:64. doi: 10.1186/1471-2148-10-64 (PMC2846930; doi:10.1186/1471-2148-10-64)
Supplement: Additional file 20 — Table S15. Alignment and secondary-structure prediction of vertebrate BBS12 protein sequences. [file 1471-2148-10-64-S20.PDF]

```
                            A                                    2
1A6D.A Sec Str description

             1                 10                  20                  30                  40
Dr_Q08C17    M K R R H I G L Q Q L Q A I T S T A H A F L G P N K R L K F I Q D E D A G D A V
Ol_25834     K E H Q Y V G L H K L S A L A R T L H S F I G P Q K S Y K F I R D D R S G E S A
Ga_E22116    N H R Q H V G L Q K L S A L A G I T H S S L G P N K M Y K F I R D E T S G E S A
Tr_vader     N H Q Q H V G L Q K L S A L A E V S H S F L G P N K N Y K F I Q D D T T G E S A
Tn_79001     K H Q Q Q V G L Q E L C V L A Q V S H S F L G P N K N Y K F I Q D D T T G E S A
Xl_101288    A I R G H K G L Q Q L L S M A T S V N S F L G P M K S Y K F I F D Q I T H E S I
Gg_22667     N S R R H I G L Q Q L S S L A W A G R T L L G P M K A C K F V V D E S T D E S M
Oa_85745     N R R R H N G L Q Q L S S L A A T G R T F L G P V K S S K F I V D E S T L E S V
Mm_Bbs12     N R R R H V G L Q Q L L S F A Q T G R S F L G P V K A T K F I T D A E C H E S V
Rn_61608     N R R R H V G L Q Q L S S F A Q T G R S F L G P V K A T K F I T D A E C H E S V
Bt_16376     N R R R H T G L Q Q L S S F A E T G R T F L G P V K S S K F I I D E E C H E S V
Ec_72131     N K R R H M G L Q Q L S S F A E T G R T F L G P V K S S K F I I D E E C H E S V
Ptr_71296    N K R R H M G L Q Q L S S F A E T G R T F L G P L K S S K F I I D E E C H E S V
Hs_BBS12     N K R R H M G L Q Q L S S F A E T G R T F L G P L K S S K F I I D E E C H E S V
Pp_I1816     N K R R H M G L Q Q L S S F A E T G R T F L G P L K S S K F I I D E E C H E S V

jnet
conf         9 9 9 8 7 6 4 6 8 9 9 9 9 9 9 9 9 8 5 3 7 8 8 8 6 1 2 5 8 8 7 3 1 4 7 6 4 0
```

```
             3    B                   C
1A6D.A Sec Str description

                                   50                  60                  70                  80
Dr_Q08C17    L I G S C P R L L E H L E L D G S V G Q L L H E T V L A Q K K L F H S G T N T L
Ol_25834     L T C S C F C T I Q N L D P T C A L D Q L V R E T L Q E H E K I Y H T G S G C L
Ga_E22116    L A C S C F R L L E N L E L T C G V G Q L V Y E T V R A H Q K V Y G A G S G C L
Tr_vader     L V G S C F R I L E N L E L T C A V G Q L V H E T V Q A H Q R V Y H T G S G C L
Tn_79001     L V A S C F R I F E N L E L S C A V G Q L V N E T V Q A H H R V C H T G S G C L
Xl_101288    L T S S S F R L L E N L D L T S A I G Q L L N E T I Q A H H K S Y K T G T T T L
Gg_22667     L I C S A V R L M E S L D L T S A A G Q L L N E T I Q A Q S K E F K T G M S T L
Oa_85745     L T C S V V R L L E S L D L T S A V G Q L L N E T V Q A Q N S T Y G T G T S T L
Mm_Bbs12     L I S S T V R L L E G L D L T C A V G H L L N E A V Q A Q N N T Y K I G T S T L
Rn_61608     L V G S T V R L L E G L D L T C A V G H L L N E A V Q A Q N V T Y K T G A S T L
Bt_16376     L I S S T V R L L E S L D L T S A V G Q L L N E A I Q A Q N S T Y R T G T S T L
Ec_72131     L I S S T V R L L E S L D L T S A V G Q L L S E A V R A Q N N T Y R T G T S T L
Ptr_71296    L I S S T V R L L E S L D L T S A V G Q L L N E A V Q A Q N N T Y R T G I S T L
Hs_BBS12     L I S S T V R L L E S L D L T S A V G Q L L N E A V Q A Q N N T Y R T G I S T L
Pp_I1816     L I S S T V R L L E S L D L T S A V G Q L L N E A V Q A Q N N T Y R T G I S T L

jnet
conf         3 1 3 0 2 5 5 4 3 3 7 7 8 7 7 5 6 8 9 9 9 9 9 9 9 9 9 9 8 8 5 0 1 5 6 0 2 7 9
```

N-TERMINAL EQUATORIAL DOMAIN

```
                       D                                     E
1A6D.A Sec Str description

                                   90                  100                 110                 120
Dr_Q08C17    M F L A G A W S R V A L E C L N R G I S V S D I K S A M R G G L Q E C L D A C T
Ol_25834     M F L A G A W S N A A L H G L R Q G I S I T Q I I S A M S E G I E I C L D A I R
Ga_E22116    L F L A G A W S R A A T E C L Q K G V P V A R I I S A M S E G M D V C L D V C R
Tr_vader     L F L S G A W G R V A L E C L Q R G I S V G S T V S A L S E G M D I C L D I C K
Tn_79001     L F L A G T W S R A A L E C L Q R G I S P G S V V S A M S E G M D I C L E V C K
Xl_101288    F F M V G A W S S A V Q E C L H L G I P V S L I V S V M L D G L N S C I G H V H
Gg_22667     L F L V G A W S N A I L E C L Q Q N V P V S A I V S V M S E G L D S C C E K V Q
Oa_85745     L F L A G A W S N A A L E C L R Q D I P A P V I V A V M S E G L R S C S E E I R
Mm_Bbs12     L F L V G A W S R A V E D C L H L G I P T T V I V S V M S E G L N S C I E A V V
Rn_61608     L F L V G A W S R A V E D C L H L G V P T T V I V S V M S E G L N S C I E A V V
Bt_16376     L F L V G A W S S A A E E C L H L G V P M S L I A S V M S E G L N S C I E E V E
Ec_72131     L F L V G A W S S A A E E C L H M G V P V S L I V S V M S E G L N S C I E E V V
Ptr_71296    L F L V G A W S S A V E E C L H L G V P I S I I V S V M S E G L N F C S E E V V
Hs_BBS12     L F L V G A W S S A V E E C L H L G V P I S I I V S V M S E G L N F C S E E V V
Pp_I1816     L F L V G A W S S A V E E C L H L G V P I S I I V S V M S E G L N F C S E E V V

jnet
conf         9 9 9 9 9 9 9 9 9 9 9 8 8 4 3 7 8 8 8 7 6 2 7 8 9 9 9 9 9 9 9 9 9 8 8 7 5 2
```

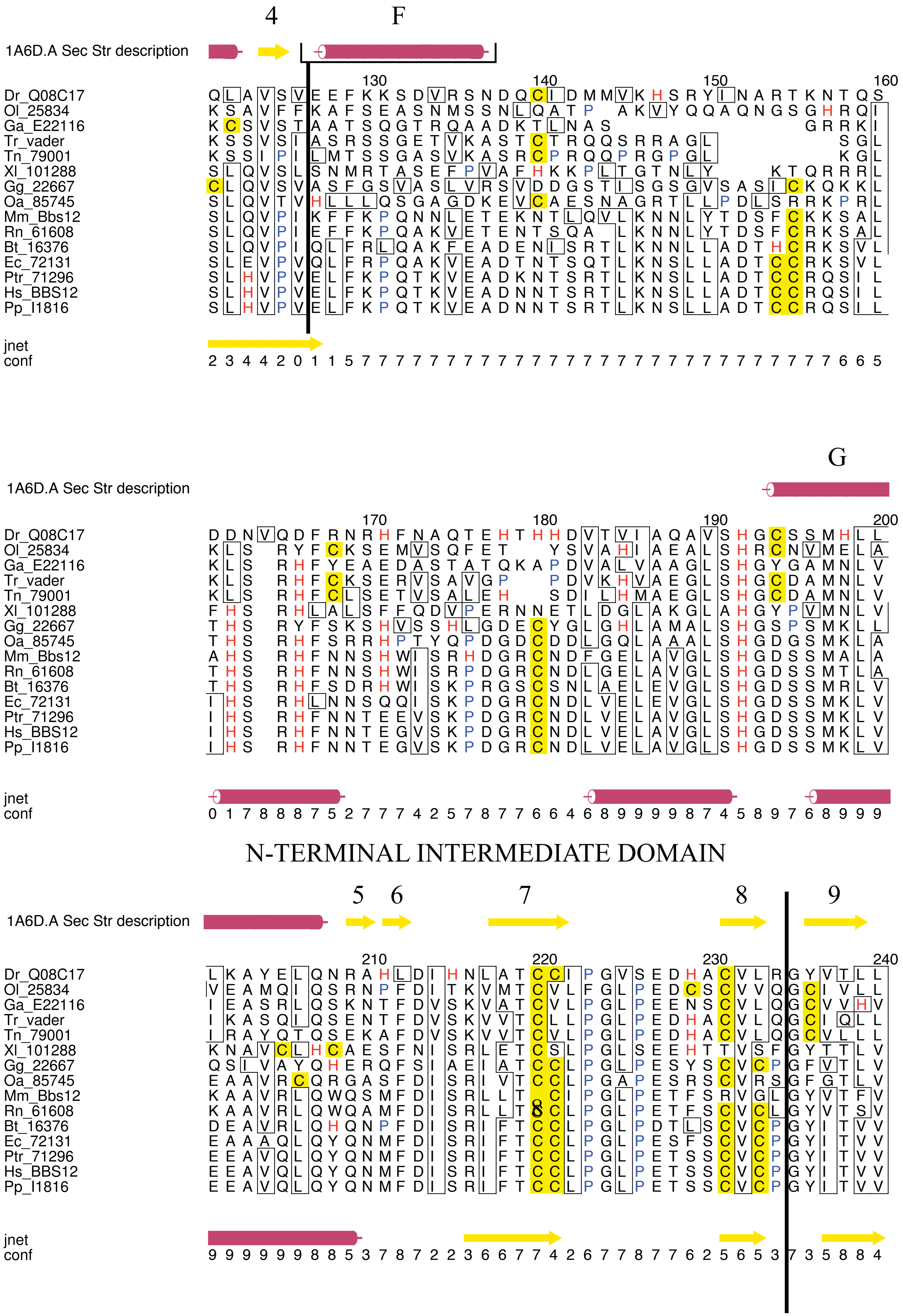
4
F
1A6D.A Sec Str description
130 140 150 160
Dr_Q08C17 QLAVSVEEFKKSDVRSNDQCIDMMVKHSRYINARTKNTQS
Ol_25834 KSAVFFKAFSEASNMSSNLQATP AKVYQQAQNGSGHRQI
Ga_E22116 KCSVSTAATSQGTRQAADKTLNAS GRRKI
Tr_vader KSSVSIASRSSGETVKASTCTRQQSRRAGL SGL
Tn_79001 KSSIPILMTSSGASVKASRCPRQQPRGPGL KGL
Xl_101288 SLQVSLSNMRTASEFPVAFHKKPLTGTNLY KTQRRRL
Gg_22667 CLQVSVASFGSVASLVRSVDDGSTISGSGVSASICKQKKL
Oa_85745 SLQVTVHLLLQSGAGDKEVCAESNAGRTLLPDLSRRKPRL
Mm_Bbs12 SLQVPIKFFKPQNNLETEKNTLQVLKNNLYTDSFCKKSAL
Rn_61608 SLQVPIEFFKPQAKVETENTSQA LKNNLYTDSFCRKSAL
Bt_16376 SLQVPIQLFRLQAKFEADENISRTLKNNLLADTHCRKSVL
Ec_72131 SLEVPVQLFRPQAKVEADTNTSQTLKNSLLADTCCRKSVL
Ptr_71296 SLHVPVELFKPQTKVEADKNTSRTLKNSLLADTCCRQSIL
Hs_BBS12 SLHVPVELFKPQTKVEADNNTSRTLKNSLLADTCCRQSIL
Pp_I1816 SLHVPVELFKPQTKVEADNNTSRTLKNSLLADTCCRQSIL
jnet
conf 2 3 4 4 2 0 1 1 5 7 7 7 7 7 7 7 7 7 7 7 7 7 7 7 7 7 7 7 7 7 7 7 7 7 7 7 7 6 6 5
G
1A6D.A Sec Str description
170 180 190 200
Dr_Q08C17 DDNVQDFRNRHFNAQTEHTHHDVTVIAQAVSHGCSSMHLL
Ol_25834 KLS RYFCKSEMVSQFET YSVAHIAEALSHRCNVMELA
Ga_E22116 KLS RHFYEAEDASTATQKAPDVALVAAGLSHGYGAMNLV
Tr_vader KLS RHFCKSERVSAVGP PDVKHVAEGLSHGCDAMNLV
Tn_79001 KLS RHFCLSETVSALEH SDILHMAEGLSHGCDAMNLV
Xl_101288 FHS RHLALSFFQDVPERNNETLDGLAKGLAHGYPVMNLV
Gg_22667 THS RYFSKSHVSSHLGDECYGLGHLAMALSHGSPSMKLL
Oa_85745 THS RHFSRRHPTYQPDGDCDDLGQLAAALSHGDGSMKLA
Mm_Bbs12 AHS RHFNNSHWISRHDGRCNDFGELAVGLSHGDSSMALA
Rn_61608 THS RHFNNSHWISRPDGRCNDLGELAVGLSHGDSSMTLA
Bt_16376 THS RHFSDRHWISKPRGSCSNLAELEVGLSHGDSSMRLV
Ec_72131 IHS RHLNNSQQISKPDGRCNDLVELEVGLSHGDSSMKLV
Ptr_71296 IHS RHFNNTEEVSKPDGRCNDLVELAVGLSHGDSSMKLV
Hs_BBS12 IHS RHFNNTEGVSKPDGRCNDLVELAVGLSHGDSSMKLV
Pp_I1816 IHS RHFNNTEGVSKPDGRCNDLVELAVGLSHGDSSMKLV
jnet
conf 0 1 7 8 8 8 7 5 2 7 7 7 4 2 5 7 8 7 7 6 6 4 6 8 9 9 9 9 8 7 4 5 8 9 7 6 8 9 9 9
N-TERMINAL INTERMEDIATE DOMAIN
5 6 7 8 9
1A6D.A Sec Str description
210 220 230 240
Dr_Q08C17 LKAYELQNRAHLDIHNLATCCIPGVSEDHACVLRGYVTLL
Ol_25834 VEAMQIQSRNPFDITKVMTCVLFGLPEDCSCVVQGCIVLL
Ga_E22116 IEASRLQSKNTFDVSKVATCVLPGLPEENSCVLQGCVVHV
Tr_vader IKASQIQSENTFDVSKVVTCLLPGLPEDHACVLQGCIQLL
Tn_79001 IRAYQTQSEKAFDVSKVVTCVLPGLPEDHACVLQGCVLLL
Xl_101288 KNAVCLHCAESFNISRLETCSLPGLSEEHTTVSFGYTTLV
Gg_22667 QSIVAYQHERQFSIAEIATCCLPGLPESYSCVCPGFVTLV
Oa_85745 EAAVRCQRGASFDISRIVTCCLPGAPESRSCVRSGFGTLV
Mm_Bbs12 KAAVRLQWQSMFDISRLLT CIPGLPETFSRVGLGYVTFV
Rn_61608 KAAVRLQWQAMFDISRLLT8CLPGLPETFSCVCLGYVTSV
Bt_16376 DEAVRLQHQNPFDISRIFTCCLPGLPDTLSCVCPGYITVV
Ec_72131 EAAAQLQYQNMFDISRIFTCCLPGLPESFSCVCPGYITVV
Ptr_71296 EEAVQLQYQNMFDISRIFTCCLPGLPETSSCVCPGYITVV
Hs_BBS12 EEAVQLQYQNMFDISRIFTCCLPGLPETSSCVCPGYITVV
Pp_I1816 EEAVQLQYQNMFDISRIFTCCLPGLPETSSCVCPGYITVV
jnet
conf 9 9 9 9 9 9 8 8 5 3 7 8 7 2 2 3 6 6 7 7 4 2 6 7 7 8 7 7 6 2 5 6 5 3 7 3 5 8 8 4

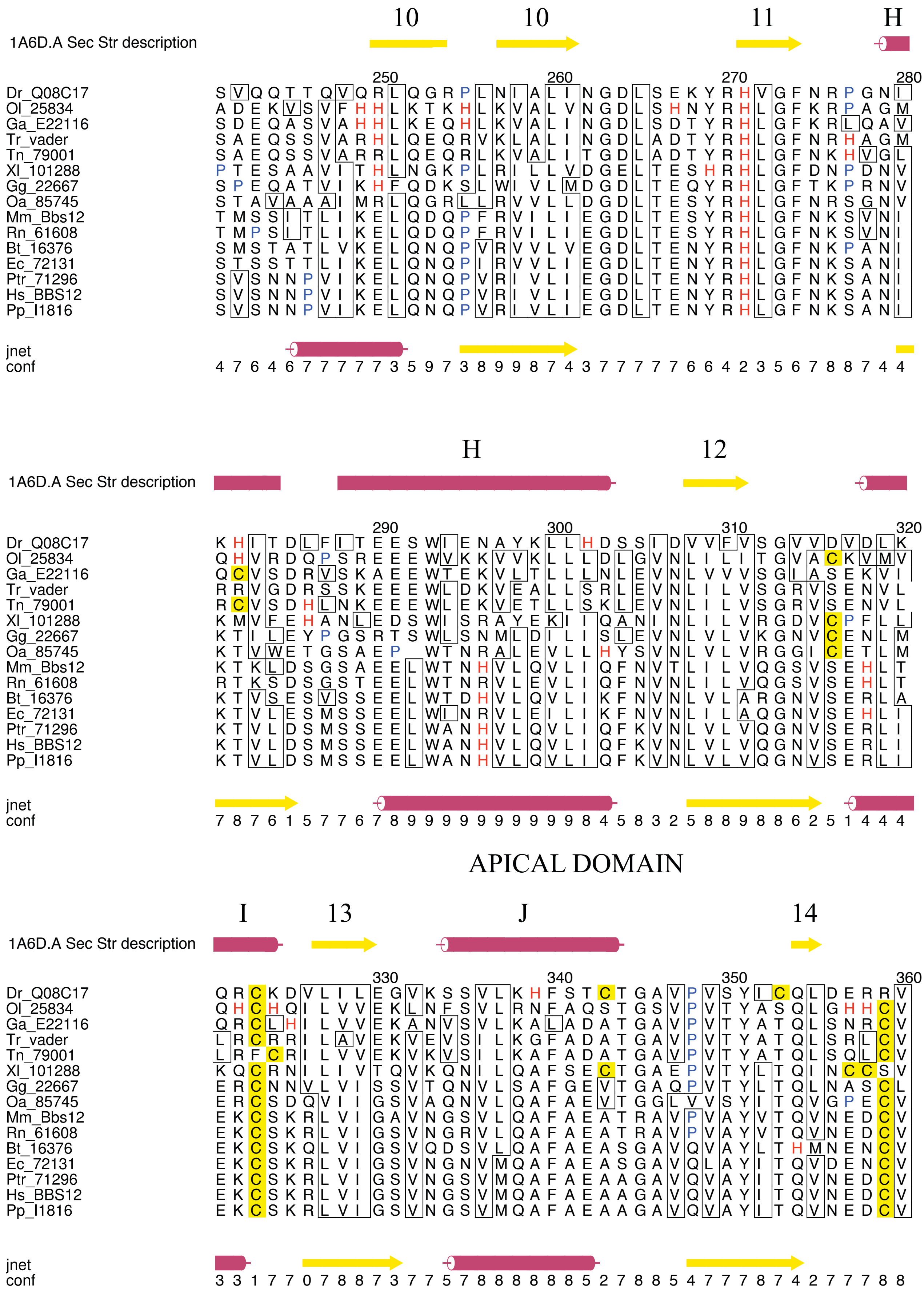
10 10 11 H
1A6D.A Sec Str description
250 260 270 280
Dr_Q08C17 S V Q Q T T Q V Q R L Q G R P L N I A L I N G D L S E K Y R H V G F N R P G N I
Ol_25834 A D E K V S V F H H L K T K H L K V A L V N G D L S H N Y R H L G F K R P A G M
Ga_E22116 S D E Q A S V A H H L K E Q H L K V A L I N G D L S D T Y R H L G F K R L Q A V
Tr_vader S A E Q S S V A R H L Q E Q R V K L A L I N G D L A D T Y R H L G F N R H A G M
Tn_79001 S A E Q S S V A R R L Q E Q R L K V A L I T G D L A D T Y R H L G F N K H V G L
Xl_101288 P T E S A A V I T H L N G K P L R I L L V D G E L T E S H R H L G F D N P D N V
Gg_22667 S P E Q A T V I K H F Q D K S L W I V L M D G D L T E Q Y R H L G F T K P R N V
Oa_85745 S T A V A A A I M R L Q G R L L R V V L L D G D L T E S Y R H L G F N R S G N V
Mm_Bbs12 T M S S I T L I K E L Q D Q P F R V I L I E G D L T E S Y R H L G F N K S V N I
Rn_61608 T M P S I T L I K E L Q D Q P F R V I L I E G D L T E S Y R H L G F N K S V N I
Bt_16376 S M S T A T L V K E L Q N Q P V R V V L V E G D L T E N Y R H L G F N K P A N I
Ec_72131 S T S S T T L I K E L Q N Q P I R V V L I E G D L T E N Y R H L G F N K S A N I
Ptr_71296 S V S N N P V I K E L Q N Q P V R I V L I E G D L T E N Y R H L G F N K S A N I
Hs_BBS12 S V S N N P V I K E L Q N Q P V R I V L I E G D L T E N Y R H L G F N K S A N I
Pp_I1816 S V S N N P V I K E L Q N Q P V R I V L I E G D L T E N Y R H L G F N K S A N I
jnet
conf 4 7 6 4 6 7 7 7 7 7 3 5 9 7 3 8 9 9 8 7 4 3 7 7 7 7 7 6 6 4 2 3 5 6 7 8 8 7 4 4
H 12
1A6D.A Sec Str description
290 300 310 320
Dr_Q08C17 K H I T D L F I T E E S W I E N A Y K L L H D S S I D V V F V S G V V D V D L K
Ol_25834 Q H V R D Q P S R E E E W V K K V V K L L L D L G V N L I L I T G V A C K V M V
Ga_E22116 Q C V S D R V S K A E E W T E K V L T L L L N L E V N L V V V S G I A S E K V I
Tr_vader R R V G D R S S K E E E W L D K V E A L L S R L E V N L I L V S G R V S E N V L
Tn_79001 R C V S D H L N K E E E W L E K V E T L L S K L E V N L I L V S G R V S E N V L
Xl_101288 K M V F E H A N L E D S W I S R A Y E K I I Q A N I N L I L V R G D V C P F L L
Gg_22667 K T I L E Y P G S R T S W L S N M L D I L I S L E V N L V L V K G N V C E N L M
Oa_85745 K T V W E T G S A E P W T N R A L E V L L H Y S V N L V L V R G G I C E T L M
Mm_Bbs12 K T K L D S G S A E E L W T N H V L Q V L I Q F N V T L I L V Q G S V S E H L T
Rn_61608 R T K S D S G S T E E L W T N R V L E V L I Q F N V N L I L V Q G S V S E H L T
Bt_16376 K T V S E S V S S E E L W T D H V L Q V L I K F N V N L V L A R G N V S E R L A
Ec_72131 K T V L E S M S S E E L W I N R V L E I L I K F N V N L I L A Q G N V S E H L I
Ptr_71296 K T V L D S M S S E E L W A N H V L Q V L I Q F K V N L V L V Q G N V S E R L I
Hs_BBS12 K T V L D S M S S E E L W A N H V L Q V L I Q F K V N L V L V Q G N V S E R L I
Pp_I1816 K T V L D S M S S E E L W A N H V L Q V L I Q F K V N L V L V Q G N V S E R L I
jnet
conf 7 8 7 6 1 5 7 7 6 7 8 9 9 9 9 9 9 9 9 9 8 4 5 8 3 2 5 8 8 9 8 8 6 2 5 1 4 4 4
APICAL DOMAIN
I 13 J 14
1A6D.A Sec Str description
330 340 350 360
Dr_Q08C17 Q R C K D V L I L E G V K S S V L K H F S T C T G A V P V S Y I C Q L D E R R V
Ol_25834 Q H C H Q I L V V E K L N F S V L R N F A Q S T G S V P V T Y A S Q L G H H C V
Ga_E22116 Q R C L H I L V V E K A N V S V L K A L A D A T G A V P V T Y A T Q L S N R C V
Tr_vader L R C R R I L A V E K V E V S I L K G F A D A T G A V P V T Y A T Q L S R L C V
Tn_79001 L R F C R I L V V E K V K V S I L K A F A D A T G A V P V T Y A T Q L S Q L C V
Xl_101288 K Q C R N I L I V T Q V K Q N I L Q A F S E C T G A E P V T Y L T Q I N C C S V
Gg_22667 E R C N N V L V I S S V T Q N V L S A F G E V T G A Q P V T Y L T Q L N A S C L
Oa_85745 E R C S D Q V I I G S A V A Q N V L Q A F A E V T G G L V V S Y I T Q V G P E C V
Mm_Bbs12 E K C S K R L V I G A V N G S V L Q A F A E A T R A V P V A Y V T Q V N E D C V
Rn_61608 E K C S K R L V I G S V N G R V L Q A F A E A T R A V P V A Y V T Q V N E D C V
Bt_16376 E K C S K Q L V I G S V Q D S V L Q A F A E A S G A V Q V A Y L T H M N E N C V
Ec_72131 E K C S K R L V I G S V N G N V M Q A F A E A S G A V Q L A Y I T Q V D E N C V
Ptr_71296 E K C S K R L V I G S V N G S V M Q A F A E A A G A V Q V A Y I T Q V N E D C V
Hs_BBS12 E K C S K R L V I G S V N G S V M Q A F A E A A G A V Q V A Y I T Q V N E D C V
Pp_I1816 E K C S K R L V I G S V N G S V M Q A F A E A A G A V Q V A Y I T Q V N E D C V
jnet
conf 3 3 1 7 7 0 7 8 8 7 3 7 7 5 7 8 8 8 8 8 5 2 7 8 8 5 4 7 7 7 8 7 4 2 7 7 7 8 8

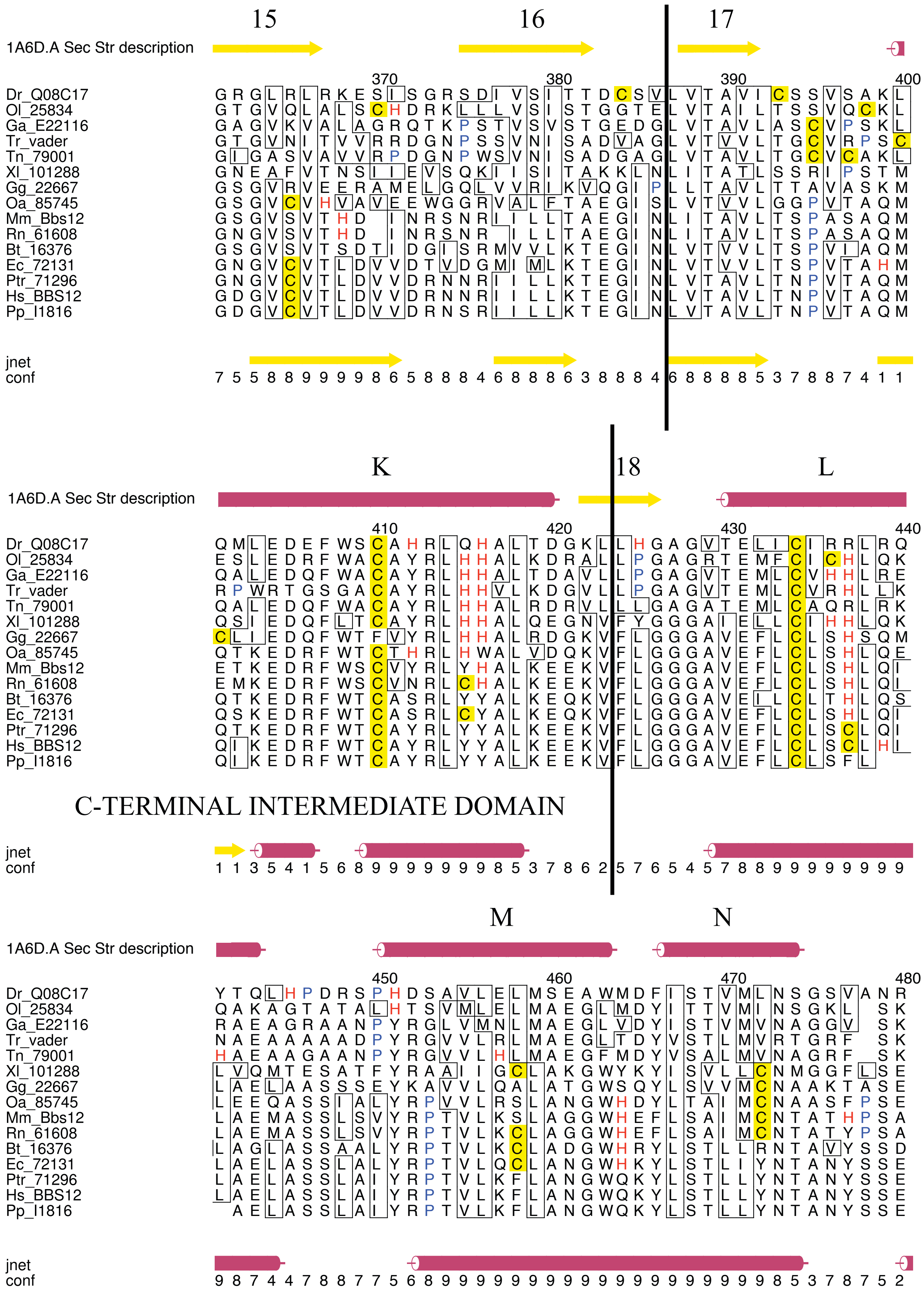
15 16 17
1A6D.A Sec Str description
370 380 390 400
Dr_Q08C17 G R G L R L R K E S I S G R S D I V S I T T D C S V L V T A V I C S S V S A K L
Ol_25834 G T G V Q L A L S C H D R K L L L V S I S T G G T E L V T A I L T S S V Q C K L
Ga_E22116 G A G V K V A L A G R Q T K P S T V S V S T G E D G L V T A V L A S C V P S K L
Tr_vader G T G V N I T V V R R D G N P S S V N I S A D V A G L V T V V L T G C V R P S C
Tn_79001 G I G A S V A V V R P D G N P W S V N I S A D G A G L V T A V L T G C V C A K L
Xl_101288 G N E A F V T N S I I E V S Q K I I S I T A K K L N L I T A T L S S R I P S T M
Gg_22667 G S G V R V E E R A M E L G Q L V V R I K V Q G I P L L T A V L T T A V A S K M
Oa_85745 G S G V C V H V A V E E W G G R V A L F T A E G I S L V T V V L G G P V T A Q M
Mm_Bbs12 G S G V S V T H D I N R S N R I L L L T A E G I N L I T A V L T S P A S A Q M
Rn_61608 G N G V S V T H D I N R S N R I L L T A E G I N L I T A V L T S P A S A Q M
Bt_16376 G S G V S V T S D T I D G I S R M V V L K T E G I N L V T V V L T S P V I A Q M
Ec_72131 G N G V C V T L D V V D T V D G M I M L K T E G I N L V T V V L T S P V T A H M
Ptr_71296 G N G V C V T L D V V D R N N R I I L L K T E G I N L V T A V L T N P V T A Q M
Hs_BBS12 G D G V C V T L D V V D R N N R I I L L K T E G I N L V T A V L T N P V T A Q M
Pp_I1816 G D G V C V T L D V V D R N S R I I L L K T E G I N L V T A V L T N P V T A Q M
jnet
conf 7 5 5 8 8 9 9 9 9 8 6 5 8 8 8 4 6 8 8 8 6 3 8 8 8 4 6 8 8 8 8 5 3 7 8 8 7 4 1 1
K 18 L
1A6D.A Sec Str description
410 420 430 440
Dr_Q08C17 Q M L E D E F W S C A H R L Q H A L T D G K L L H G A G V T E L I C I R R L R Q
Ol_25834 E S L E D R F W A C A Y R L H H A L K D R A L L P G A G R T E M F C I C H L Q K
Ga_E22116 Q A L E D Q F W A C A Y R L H H A L T D A V L L P G A G V T E M L C V H H L R E
Tr_vader R P W R T G S G A C A Y R L H H V L K D G V L L P G A G V T E M L C V R H L L K
Tn_79001 Q A L E D Q F W A C A Y R L H H A L R D R V L L L G A G A T E M L C A Q R L R K
Xl_101288 Q S I E D Q F L T C A Y R L H H A L Q E G N V F Y G G G A I E L L C I H H L Q K
Gg_22667 C L I E D Q F W T F V Y R L H H A L R D G K V F L G G G A V E F L C L S H S Q M
Oa_85745 Q T K E D R F W T C T H R L H W A L V D Q K V F L G G G A V E F L C L S H L Q E
Mm_Bbs12 E T K E D R F W S C V Y R L Y H A L K E E K V F L G G G A V E F L C L S H L Q I
Rn_61608 E M K E D R F W S C V N R L C H A L K E E K V F L G G G A V E F L C L S H L Q I
Bt_16376 Q T K E D R F W T C A S R L Y Y A L K E Q K V F L G G G A V E I L C L T H L Q S
Ec_72131 Q S K E D R F W T C A S R L C Y A L K E Q K V F L G G G A V E F L C L S H L Q I
Ptr_71296 Q T K E D R F W T C A Y R L Y Y A L K E E K V F L G G G A V E F L C L S C L Q I
Hs_BBS12 Q I K E D R F W T C A Y R L Y Y A L K E E K V F L G G G A V E F L C L S C L H I
Pp_I1816 Q I K E D R F W T C A Y R L Y Y A L K E E K V F L G G G A V E F L C L S F L
C-TERMINAL INTERMEDIATE DOMAIN
jnet
conf 1 1 3 5 4 1 5 6 8 9 9 9 9 9 9 9 8 5 3 7 8 6 2 5 7 6 5 4 5 7 8 8 9 9 9 9 9 9 9 9
M N
1A6D.A Sec Str description
450 460 470 480
Dr_Q08C17 Y T Q L H P D R S P H D S A V L E L M S E A W M D F I S T V M L N S G S V A N R
Ol_25834 Q A K A G T A T A L H T S V M L E L M A E G L M D Y I T T V M I N S G K L S K
Ga_E22116 R A E A G R A A N P Y R G L V M N L M A E G L V D Y I S T V M V N A G G V S K
Tr_vader N A E A A A A A D P Y R G V V L R L M A E G L T D Y V S T L M V R T G R F S K
Tn_79001 H A E A A G A A N P Y R G V V L H L M A E G F M D Y V S A L M V N A G R F S K
Xl_101288 L V Q M T E S A T F Y R A A I I G C L A K G W Y K Y I S V L L C N M G G F L S E
Gg_22667 L A E L A A S S S E Y K A V V L Q A L A T G W S Q Y L S V V M C N A A K T A S E
Oa_85745 L E E Q A S S I A L Y R P V V L R S L A N G W H D Y L T A I M C N A A S F P S E
Mm_Bbs12 L A E M A S S L S V Y R P T V L K S L A G G W H E F L S A I M C N T A T H P S A
Rn_61608 L A E M A S S L S V Y R P T V L K C L A G G W H E F L S A I M C N T A T Y P S A
Bt_16376 L A G L A S S A A L Y R P T V L K C L A D G W H R Y L S T L L R N T A V Y S S D
Ec_72131 L A E L A S S L A L Y R P T V L Q C L A N G W H K Y L S T L I Y N T A N Y S S E
Ptr_71296 L A E L A S S L A I Y R P T V L K F L A N G W Q K Y L S T L L Y N T A N Y S S E
Hs_BBS12 L A E L A S S L A I Y R P T V L K F L A N G W Q K Y L S T L L Y N T A N Y S S E
Pp_I1816 A E L A S S L A I Y R P T V L K F L A N G W Q K Y L S T L L Y N T A N Y S S E
jnet
conf 9 8 7 4 4 7 8 8 7 7 5 6 8 9 9 9 9 9 9 9 9 9 9 9 9 9 9 9 9 9 9 8 5 3 7 8 7 5 2

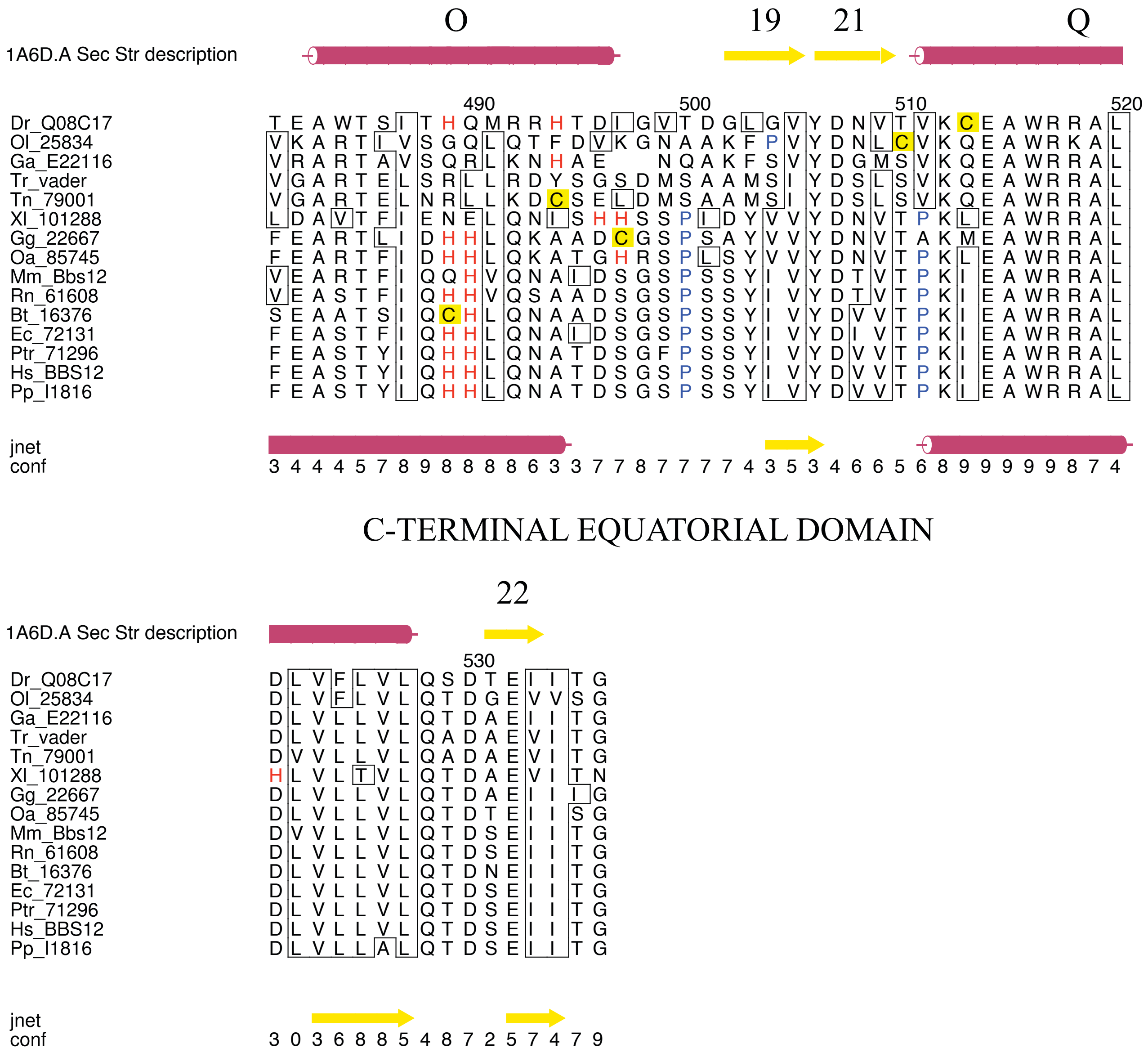

Supplementary figure S15. Alignment and secondary-structure predictions of BBS12 sequences compared to PDB secondary-structure description of 1a6d. See Legend for Supplementary figure S10 for symbols and Legend for Figure 2 for species abbreviations. A gapped alignment corresponding to helix F of 1A6D.A was automatically excluded by the prediction tool.
