# Supplementary material for: Chaperonin genes on the rise: new divergent classes and intense duplication in human and other vertebrate genomes
Source: BMC Evol Biol. 2010 Mar 1;10:64. doi: 10.1186/1471-2148-10-64 (PMC2846930; doi:10.1186/1471-2148-10-64)
Supplement: Additional file 21 — Table S6. Residue types at monomer-monomer interaction positions in thermosome, CCT, BBS, and CCT8L proteins. [file 1471-2148-10-64-S21.DOC]

Table S6. Residue types at monomer-monomer interaction positions in thermosome, CCT, BBS, and CCT8L proteins

| **A. Intra-ring** | | | | | | |
| --- | --- | --- | --- | --- | --- | --- |
| **Thermosome^1^** | **CCTs^2^** | **BBS12^3^** | **BBS10^3^** | **MKKS^3^** | **CCT8L^3^** | **Conserved aa types in CCTs** |
| 19 Q | HQRST | R | S | R | S |  |
| 24 Q | LQRV | R | V | V | L |  |
| 27 N | HNS | G* | A* | T* | S |  |
| 40 T | ST | T | C* | S | P* | Hydroxyl |
| 45 K | KNRV | L* | E* | S* | H* |  |
| 47 M | LMT | S* | R* | R* | R* |  |
| 48 D | DMN | S* | Q* | L* | Q* |  |
| 49 K | K | K | V* | K | K | Lysine |
| 50 M | ILM | F* | L | Q* | F* | Intermediate-size hydrophobic |
| 51 L | ILMV | I | C* | L | L | Intermediate-size hydrophobic |
| 52 V | ILQV | I | T* | H* | V |  |
| 57 D | DGK | H* | E* | G | E* |  |
| 58 I | AILV | S* | V | V | T* | Aliphatic |
| 60 I | ILMV | L | L | T* | C* | Intermediate-size hydrophobic |
| 76 P | PQ | A* | P | P | P |  |
| 77 T | AIT | V* | I | I | A |  |
| 79 K | KRS | Q* | R | K | W* |  |
| 80 M | LMSTV | L | M | I* | L |  |
| 83 E | DEKM | E | D | A* | E |  |
| 87 A | ALMSTV | A | S | N* | T |  |
| 88 Q | Q | Q | H* | H* | Q | Glutamine |
| 89 D | DE | N* | L* | V* | A* | Acidic |
| 90 T* | ADEIKQ | N* | K | S* | E |  |
| 18 V* | ILM | V* | K* | L | L | Intermediate-size hydrophobic |
| 119 H | HS | P* | D* | T* | P* |  |
| 120 P | PV | I* | P | P | R |  |
| 121 T | IQRST | S | L* | T | P* |  |
| 128 R | EQR | S* | K* | K* | A* |  |
| 135 R | HILRV | S* | L | I | L |  |
| 163 N* | AILQV | P* | V | P* | P* |  |
| 166 L* | EHIQRS | S | N* | M* | * |  |
| 203 N* | HKLPQV | P | N* | K | HP |  |
| 206 S | AGILRST | P* | P* | R | T |  |
| 207 V* | EGILQ | E | V* | V* | L |  |
| 208 N* | ADEMST | T | S | I* | E |  |
| 224 H | GHNSVY | N | V | Q* | G |  |
| 226 K* | DGQR | P* | -* | M* | M* |  |
| 245 I* | AGKLPTY | Y | P | G | P |  |
| 246 K | DEKMPT | R* | L* | D | A* |  |
| 247 K | AIK | H* | F* | T* | H* |  |
| 248 T | EGIMPT | L* | S* | S* | P |  |
| 249 E | DEK | G* | T* | D | N* | Charged |
| 250 I | DILMSTV | F* | S | T | A* |  |
| 251 E* | DGKNQ | N | G | G | P* |  |
| 252 A | AGHNSTV | K* | S | E* | A |  |
| 253 K | DEGKQRT | S* | E | G | T |  |
| 254 V | FILV | A* | F | T* | A* | Hydrophobic |

Table S6 (continued 1)

| **Thermosome^1^** | **CCTs^2^** | **BBS12^3^** | **BBS10^3^** | **MKKS^3^** | **CCT8L^3^** | **Conserved aa types in CCTs** |
| --- | --- | --- | --- | --- | --- | --- |
| 255 Q* | DEFLRV | N* | I* | V | C*R |  |
| 256 I | IVY | I | L* | V | L* |  |
| 257 S | DHKST | K | N* | V* | S |  |
| 259 P | AEPTVY | V | E | Y | P |  |
| 262 I* | FKLMRVY | S* | F | -* | L |  |
| 263 Q | ADEKMQT | M | Q | V* | A |  |
| 264 D* | AEKNQR | R | T* | S* | Q |  |
| 265 F | FILV | L | S* | L | F | Hydrophobic |
| 266 L | ELQRSV | Q | Q | E | S |  |
| 268 Q* | AEGMRY | D* | W* | A | G |  |
| 269 E | E | S* | I* | V* | S* | Glutamate |
| 270 T* | EKRSW | S | M* | L* | D* |  |
| 274 K | DEKLQY | N* | K | L | E |  |
| 275 Q | ADENQ | H* | A | N | K* |  |
| 297 V | EFLMVY | R* | L | S* | E |  |
| 300 H | DHKQ | E* | Y* | Q | T* |  |
| 301 Y | AFLSY | K* | Y | F | L |  |
| 304 K | ADEKQR | N* | V* | M* | K |  |
| 315 K | EFGKR | G | S* | V* | RW* |  |
| 319 E | EKNR | Q* | S* | E | I* |  |
| 328 K | EKMRSTV | V | S | Q* | P* |  |
| 330 V | ALQV | V | F* | I* | L |  |
| 331 T | ANPST | A | V* | G* | P |  |
| 332 D | HRST | Y* | P | S | R |  |
| 335 D | ADEHNPQ | Q | A | S* | P |  |
| 340 V | CDEKMV | C | L* | S* | R* |  |
| 354 D | DEGKS | R* | Y* | -* | D |  |
| 376 G | AGPS | P | P | R* | A |  |
| 377 T | ANST | V* | V* | N | T |  |
| 378 D | DEKQ | T* | H* | D | T* |  |
| 379 H | EFHLMNQ | A* | G* | T* | Q |  |
| 380 V | FILMTV | Q* | L | A* | G* | Hydrophobic |
| 500 H | KNQSW | E* | Q | S | Q |  |
| 503 E | HKLST | R* | T | Q* | R* |  |
| 504 S | AFLST | R* | S | V* | A |  |
| 507 E | ENQV | D* | Q | E | E |  |
| 508 V | AIMT | L* | C* | T | V* |  |
| 510 T | CEIRTV | L* | T | N* | L* |  |
| 511 M | LMNSTV | L | K* | L | Q* |  |
| 514 R | KLRS | Q* | T* | D* | T* |  |
| 515 I | IV | T* | I | L* | V | Beta-branched aliphatic |
| 516 D | D | D | D | S* | D | Aspartate |
| 517 D | DENQ | S* | M* | Y* | E |  |
| 518 V | ILTV | E* | V | V | I | Intermediate-size hydrophobic |
| 519 I | IMRV | I | I | I | V |  |
| 520 A | KMNRS | I* | T* | E* | V* |  |

Table S6 (continued 2)

| **B. Inter-ring** | | | | | | |
| --- | --- | --- | --- | --- | --- | --- |
| **Thermosome^1^** | **CCTs^2^** | **BBS12^3^** | **BBS10^3^** | **MKKS^3^** | **CCT8L^3^** | **Conserved aa types in CCTs** |
| 25 R* | AFKLSVY | H* | K | R* | L |  |
| 29 E* | CIMNQS | Q | Q | S | A* |  |
| 108 K | DEKRS | S | R | E | E | Charged/hydrophilic |
| 115 D | ADEKQRS | H* | D | -* | K |  |
| 116 Q | EIKQR | L* | R | -* | A*F* |  |
| 117 G | GKQ | G | E* | G | G |  |
| 429 R | KLMRV | W* | E* | E* | P* |  |
| 432 L | LVWY | N* | M* | L | P* |  |
| 439 K | DEKQR | S* | N* | S* | RW* | Charged/hydrophilic |
| 446 R | KMNRST | T | K | G* | K |  |
| 450 E | DEQV | N* | K* | H* | E |  |
| 455 D | DKNS | S | K | I* | A* | Charged/hydrophilic |
| 456 P | ALPST | S | Y* | L | V* |  |
| 457 I | AINQT | E* | S* | T | S* |  |
| 458 N | DENQRS | F* | F* | D | D |  |

^1^Residue observed in the model structure of thermosome subunit A from *T. acidophilum*; ^2^Residues observed in all human CCT subunits; ^3^ Residues observed in human BBS and CCT8L proteins; *Residue-types not observed in any human CCT subunit.
