# Supplementary material for: Chaperonin genes on the rise: new divergent classes and intense duplication in human and other vertebrate genomes
Source: BMC Evol Biol. 2010 Mar 1;10:64. doi: 10.1186/1471-2148-10-64 (PMC2846930; doi:10.1186/1471-2148-10-64)
Supplement: Additional file 22 — Table S7. Database and sequence information on all hsp60-like sequences identified in the human, mouse and rat genomes. [file 1471-2148-10-64-S22.PDF]

Table S7

sequence name	alternate names	species names	protein accession number	refseq number	ENSEMBL ID	classification	Chr.	location	strand	Exons	start	end	remarks	sequence
**CCT1-3P**		Hs			OTTHUMG00000033751	CCTalpha pseudogene 3 vert	7	p14.1	+	2	42,801,030	42,802,033	Not identified in pseudogene.org	MERPLSVFGDRSTGEAICSQNVMAAALIANTVKSSLGPVGLVKMLVDDIGDVSITNGAKV<br>LCELADLQDKEVGDGTTSVVIIAAELLKNEDELVKQKIHPTSVISGYGLACKEAVRYINE<br>NLIVNTDKVGRDCLINAAKTSTSSKIIGINGDFFANMVVDAVFAIKYTDIRGQPCYPVNS<br>VNILKAHGKSQTESMLISGYALNCVVGSQGIPKRIVNAKIACLDFGLQKTKMKLGIQVVI<br>TDPEKLDQIRQRESDITKERIQKILVTGASIILTTGGIDDMCLKYFVEAGATAVRRVFKR<br>DLKRIPKASGATILSTLANLKVLEVLARTIRQEKEIKGIQLGKEKVKLYLFADDMIVYLE<br>N
**CCT1-1P**		Hs				CCTalpha pseudogene 1 vert	12	p12.2	+	1	19986638	19987216	Not identified in pseudogene.org	LKNTKAHTSASIILRGANDIMCGEMERSLHDALCVVKRVLESKSMVPGGGAVEAALSIYLENYAT<br>SMGSQEEIAEFARSLLVIPNTLAVNAAQDSTDLVTKFRAFHNEAQVNPEHKNLKWIGLDLSNGER<br>GDNKQAGMFEPTIVKVKSLKFATEAAITILRIDDLTKLHPESKDDKHGGYEDAVHSGALND
**CCT1-2P**		Hs				CCT1alpha pseudogene 2 vert	5	p13.1	-	2	41621756	41623646	Not identified in pseudogene.org	MEGPLSVFGDQDRSTGEVVRSQNVMAASIANTVKSSLGPVGLDKMLVDDVGDVTITNDGAAILRL<br>PEVEHPAAEVLCELADLQDKEVGDGTTSIEALRYIRNLIIYTDELGRDCLINSTETWMSSKIIGI<br>NGDLFANIVADTVLVTTYTDIRGQPRYPANSVNILKAHARSQIESMLISGYALSCVVGSQGMPKI<br>IVNAEIALNSSLLDVQVVITDPEKLDQIRQKESDIIKERIPKILATDANIILIAGGIDDMCLKYF<br>VEVGAMAIRRALKRDLKCIAETSGGTVLSTLANLKGEETFETAVSGQVEEVVQERICDDELILIK<br>TSKACASASIILHGANDFVCDEMERSLHDALCIVKKVLESKSMMLVGDAVEAAFSIYPENCETSM<br>ESQEQLAIAESARPLPVIPLTVNAAQNSTDLVASLRAFHNEAQVNPESKNLKIGLDLRNGKPRHN<br>KQAGVFEPTTVKVKSLKFATEAAVTILQIDDLIKLYPESKDVKHGGYEDAAHTGALD
**TCP1**	CCT1, CCTa, TCP-1 alpha	Hs			ENST00000321394	CCTalpha (real gene) vert	6	q25.3	-	12	160,119,520	160,130,731	tcp1 = Tailless Complex Polypeptide 1	MEGPLSVFGDRSTGETIRSQNVMAAASIANIVKSSLGPVGLDKMLVDDIGDVTITNDGAT<br>ILKLLEVEHPAAKVLCELADLQDKEVGDGTTSVVIIAAELLKNADELVKQKIHPTSVISG<br>YRLACKEAVRYINENLIVNTDELGRDCLINAAKTSMSSKIIGINGDFFANMVVDAVLAIK<br>YTDIRGQPRYPVNSVNILKAHGRSQMESMLISGYALNCVVGSQGMPKRIVNAKIACLDFS<br>LQKTKMKLGVQVVITDPEKLDQIRQRESDITKERIQKILATGANVILTTGGIDDMCLKYF<br>VEAGAMAVRRVLKRDLKRIAKASGATILSTLANLEGEETFEAAMLGQAEEVVQERICDDE<br>LILIKNTKARTSASIILRGANDFMCDEMERSLHDALCVVKRVLESKSVVPGGGAVEAALS<br>IYLENYATSMGSREQLAIAEFARSLLVIPNTLAVNAAQDSTDLVAKLRAFHNEAQVNPER<br>KNLKWIGLDLSNGKPRDNKQAGVFEPTIVKVKSLKFATEAAITILRIDDLIKLHPESKDD<br>KHGSYEDAVHSGALND
**CCT8L2**	GROL, CESK1	Hs	NP_055221	NP_055221.1	ENST00000359963	CCTtheta_L1/L2 (real gene)	22	q11.1	-	1	15,451,770	15,453,440	EST available	MDSTVPSALELPQRLALNPRESPRSPEEEEPHLLSSLAAVQTLASVIRPCYGPHGRQKFL<br>VTMKGETVCTGCATAILRALELEHPAAWLLREAGQTQAENSGDGTAFVVLLTEALLEQAE<br>QLLKAGLPRPQLREAYATATAEVLATLPSLAIQSLGPLEDPSWALHSVMNTHTLSPMDHL<br>TKLVAHACWAIKELDGSFKPERVGVCALPGGTLEDSCLLPGLAISGKLCGQMATVLSGAR<br>VALFACPFGPAHPNAPATARLSSPADLAQFSKGSDQLLEKQVGQLAAAGINVAVVLGEVD<br>EETLTLADKYGIVVIQARSWMEIIYLSEVLDTPLLPRLLPPQRPGKCQRVYRQELGDGLA<br>VVFEWECTGTPALTVVLRGATTQGLRSAEQAVYHGIDAYFQLCQDPRLIPGAGATEMALA<br>KMLSDKGSRLEGPSGPAFLAFAWALKYLPKTLAENAGLAVSDVMAEMSGVHQGGNLLMGV<br>GTEGIINVAQEGVWDTLIVKAQGFRAVAEVVLQLVTVDEIVVAKKSPTHQEIWNPDSKKT<br>KKHPPPVETKKILGLNN
**CCT8-1P**	chr1.mb145,	Hs				TCP1 theta pseudogene 4 vert	1	q21.1	-	1	145141482	145143137	Identified in pseudogene.org, processed	MALQVPKAPGFAQMLKEGAKHFSELEEAVYRNIQACKELAQTTRTAYGRN<br>GMKKMVINYLEKLFVTNDAATILRELEVQHPAAKMTVMASHMQEQEVGDG<br>TNIVLVFAGALLELAEELLRIGLSVSEVIEGYEIACRKAHEILPNLVRCS<br>AKNLRDVDEVSSLLRTSVMCKQYGNEVFLAKLIVQACVSIFPDSGHFKVD<br>NIRVCKILGCGITSSSVLHGMVFKKETEGDVTSVKDAKIAVYSCPFDGM<br>ITETKGTVLIKTDEELMNLSKGEENLMDAVKAIADTGANVVVTGGKVA<br>DMALHYANKYNMMLVKLNSKWDVRLCKTVGATALPRLTPPVLEEMGHD<br>SVYLSEVGDTQVVVFKHEKEDGIISTIVLQGSTDNLMDDIERAVDDGVNT<br>FKVLTRDKRLVPGGGATEIELAKQITSYGETCPGLEQYAIKKFAEAFEAI<br>PRALAENSGENSGVKANEVISKLYAVPQEGNKNVGLDTEAVVPAVTDM<br>LEAGVLDTYLGKHWSIKLAANAAVTVLRVGQVIMAKPDGGPKPPSGKKDW<br>DDDQND
**CCT5-1P**	Human.chr13.mb78	Hs				CCTepsilon pseudogene 1 vert	13	q31.1	+	1	78382086	78382680	Not identified in pseudogene.org	ASMGTLAFD*YGPPFLIIKDQDRKSRLMGLEALKSHIMVANAVAHATRTSLGPKGLHKMV<br>VDKDGDVTVTNDGVTILSVMDVSHQIAQFDGGNV*VSG**NWRWNHRCGCPGWCLVRRSP<br>GVARPKYSSNQNSRWL*AGCLLCCGTPGQDQ*QHPC*HKRHQTPDSDCKNHAGLQSGQQL<br>LPTNG*DCCECHPPLTDMEQRGVDFELIKVESKVGGSLEDNKRIKGVIVDKYFSHPQMPK<br>KVEDAKIVILTCPFELPKPKAKHKLDVTSVEDYKALQKYEKEKF*EMIQQIKETGANLEI<br>CQWGFDDEANHLLLQNNLPAVC*VGGPEIELIAITTGGQIITKFSELMARKLGFAGLVQE<br>ISFWTTKDKMLVIEQCKNSRAITIFMRRRNKMIIEEAKQSLHGALCVIWNLIRDNHVVYG<br>GGAAEISCALPVSQEVDKCPTLEQYAMRALANTLEVIPKALSENGGMNPIQSMTKV*ASQ<br>VKEMNLALGIDCLHKGTNYMKQ*RVTETSIGKKATDISCNTNG*NDFEE*QHS*DWRI*R
**CCT5**	TCP1E, TCP1 epsilon,KIAA0098	Hs	NP_036205	NM_012073.3	ENST00000280326	CCTepsilon (real gene) vert	5	p15.2	+	11	10,303,453	10,317,892		MASMGTLAFDEYGRPFLIIKDQDRKSRLMGLEALKSHIMAAKAVANTMRTSLGPNGLDKM<br>MVDKDGDVTVTNDGATILSMMDVDHQIAKLMVELSKSQDDEIGDGTTGVVVLAGALLEEA<br>EQLLDRGIHPIRIADGYEQAARVAIEHLDKISDSVLVDIKDTEPLIQTAKTTLGSKVVNS<br>CHRQMAEIAVNAVLTVADMERRDVDFELIKVEGKVGGRLEDTKLIKGVIVDKDFSHPQMP<br>KKVEDAKIAILTCPFEPPKPKTKHKLDVTSVEDYKALQKYEKEKFEEMIQQIKETGANLA<br>ICQWGFDDEANHLLLQNNLPAVRWVGGPEIELIAIATGGRIVPRFSELTAEKLGFAGLVQ<br>EISFGTTKDKMLVIEQCKNSRAVTIFIRGGNKMIIEEAKRSLHDALCVIRNLIRDNRVVY<br>GGGAAEISCALAVSQEADKCPTLEQYAMRAFADALEVIPMALSENSGMNPIQTMTEVRAR<br>QVKEMNPALGIDCLHKGTNDMKQQHVIETLIGKKQQISLATQMVRMILKIDDIRKPGESE<br>E
**CCT7**	TCP1 eta	Hs	NP_006420	NM_006429.2	ENST00000258091	CCTeta (real gene) vert	2	p13.2	+	11	733,320,279	73,333,494		MPTPVILLKEGTDSSQGIPQLVSNISACQVIAEAVRTTLGPRGMDKLIVDGRGKATISND<br>GATILKLLDVVHPAAKTLVDIAKSQDAEVGDGTTSVTLLAAEFLKQVKPYVEEGLHPQII<br>IRAFRTATQLAVNKIKEIAVTVKKADKVEQRKLLEKCAMTALSSKLISQQKAFFAKMVVD<br>AVMMLDDLLQLKMIGIKKVQGGALEDSQLVAGVAFKKTFSYAGFEMQPKKYHNPKIALLN<br>VELELKAEKDNAEIRVHTVEDYQAIVDAEWNILYDKLEKIHHSGAKVVLSKLPIGDVATQ<br>YFADRDMFCAGRVPEEDLKRTMMACGGSIQTSVNALSADVLGRCQVFEETQIGGERYNFF<br>TGCPKAKTCTFILRGGAEQFMEETERSLHDAIMIVRRAIKNDSVVAGGGAIEMELSKYLR<br>DYSRTIPGKQQLLIGAYAKALEIIPRQLCDNAGFDATNILNKLRARHAQGGTWYGVDINN<br>EDIADNFEAFVWEPAMVRINALTAASEAACLIVSVDETIKNPRSTVDAPTAAGRGRGRGR<br>PH

**CCT8L1**	LOC155100	Hs	NP_001025037	NM_001029866.1	ENST00000021776	CCTtheta_L1/L2(real gene)	7	q36.1	+	1	151,773,495	151,775,165		MDSTVPSALELPQRLALNPRESPRSPEEEEPHLLSSLAAVQTLANVIRPCYGPHGRQKFL<br>VTMKGETVCTGCATAILRALELEHPAAWLLREAAQTQAENSGDGTAFVVLLTEALLEQAE<br>QLLKFGLPRPQLREAYATATAEVLATLPSLAIQSLGPLEDPSWALHSVMNTHTLPPMNHL<br>TKLVAHACWAIKELDGSFKPERVGVCTLHGGTLEDSCLLQGLAISGKLCGQMAAVLSGAR<br>VALFACPFGPAHPNAPATACLSSPADLAQFSKGSDQLLEKQVGQLAAAGINVAVVLGEVD<br>EETLTLADKYGIVVIQARSRMEIIYLSEVLDTPLLPRLLPPQRPGKCQRVYRQELGDGLA<br>VVFEWECTGTPALTVVLRGATTQGLRSAEQAVYHSIDAYFQPCQDPRLIPGAGATEMALA<br>KMLSDKGSRLEGPNGPAFLAFARALKYLPKTLAENAGLAVSDVVAEMSGVHQGGNLLMGV<br>GAEGIINVAQEGVWDTLIVKAQGFRAVAEVVLQLVTVDEIVVAKKSPTHQQIWNPDSKKT<br>KKRPPPVEKKKILGMNN
**CCT5-3P**		Hs				CCTepsilon<br>pseudogene 3<br>vert	5	q22.3	+	2	114876388	114877290	Not identified in pseudogene.org	SHL*VPKKVEDAKIAILTCPFEPPKPKTKHKLDVTSIDHKALHKYEKEKFEEMIQQIKETGANLA<br>ICQWGFDD*ANHLFLQNNLPVVRWVGGLEIELIAISTRGRIVPGSQSSRPRCWPMALSENSGMNP<br>IQTTTKVRARQVKEMNPALGTDCLHKGTNDMKRQHVIEILIGKKQQTSFATQMVRMILKIDDIHK<br>PGESEE
**CCT4**	TCPD, TCP-1 delta	Hs			ENSP00000233836	CCTdelta<br>(real gene)<br>vert	2	p15	-	13	61,950,076	61,969,146		MPENVAPRSGATAGAAGGRGKGAYQDRDKPAQIRFSNISAAKAVADAIRTSLGPKGMDKM<br>IQDGKGDVTITNDGATILKQMQVLHPAARMLVELSKAQDIEAGDGTTSVVIIAGSLLDSC<br>TKLLQKGIHPTIISESFQKALEKGIEILTDMSRPVELSDRETLLNSATTSLNSKVVSQYS<br>SLLSPMSVNAVMKVIDPATATSVDLRDIKIVKKLGGTIDDCELVEGLVLTQKVSNSGITR<br>VEKAKIGLIQFCLSAPKTDMDNQIVVSDYAQMDRVLREERAYILNLVKQIKKTGCNVLLI<br>QKSILRDALSDLALHFLNKMKIMVIKDIEREDIEFICKTIGTKPVAHIDQFTADMLGSAE<br>LAEEVNLNGSGKLLKITGCASPGKTVTIVVRGSNKLVIEEAERSIHDALCVIRCLVKKRA<br>LIAGGGAPEIELALRLTEYSRTLSGMESYCVRAFADAMEVIPSTLAENAGLNPISTVTEL<br>RNRHAQGEKTAGINVRKGGISNILEELVVQPLLVSVSALTLATETVRSILKIDDVVNTR
**HSPD1-2P**	Hsp60s2	Hs	AAK60261		ENSP00000328369	GroEL pseudogene 2	5	p14.3	-	1	21919402	21920175	Not recorded in pseudogene.org database, processed, retrotransposed.	MAIATGGAVFGEEGLTLNLEDVQPHDLGKVGEVIVTKDDAMLLKGKGDKAQLEKRIQEII<br>GQLDVTTSEYEKEKLNEWLAKLSDGVVVLKFGGTSDVEVNEKKDRVTDALNATRAAVEGG<br>IVLGGGFALLRCIPALDSLTPANEDQKIGMEIIKRTLKIPAMTTATNAGVEGSLIVEKIM<br>QNSSEVGYDAMVGDFMNMVEKGIIDPTKLVRTALLDAAGVASLLTTAEVVVTEIPKEEKD<br>PGMGAMGGMGGGMGGGMF
**CCT4-1P**	chrX.mb64, TCPD human	Hs			ENSG00000115484	CCTdelta<br>pseudogene 1<br>vert	X	q12	+	3	64407520	64409590	Identified in pseudogene.org	AGGYRKCSYKDREKSFQINLGNIYMAKEVANALRTSLQPXXXXXXXX<br>XXXGDMTITYDAVTIVKQMGLHPAARILAVLSKAQDIEAGDGTTSV<br>VIIAGSLLYSYNKLLQKRIHLAIISESFKALGNGIKIITDMSHMEVND<br>KETFNSTTNLLNSMLIIQHSSLISPMSVNTVIK<br>MDLATATTVDLRDIKIVKLGRTIDCSELVKGIITQKVENSGIARVEKA<br>KIGLIQFCFSAPKTEMDNQIVSDYTHVEQVLKERA<br>GDNILLTQKSLLIGALSDLELHFLNKMKIMVIKDIEKEGIEFICKTIGNK<br>PVAYIDFTTNMLGAAPLAKGVNLNSSGKLLKTAGCAGPGKA VIVVVHD<br>SNKLMIEKAKCSIYDALCFISYLVKNRALIIRYGAPEIRVALKLPEYS<br>ILRGIESYCIYDFTDDMEVTSFTLAKNTGLNHISSLAEIGNQYLQEVNTV<br>GITVQKGDILNILEEIIAQPLLVSIGVLTLATESLGILKLDDMANT
**CCT3**	TCP1 gamma	Hs	NP_005989	NM_005998.3	ENSG00000163468	CCTgamma<br>(real gene)<br>vert	1	q23.1	-	13	154,545,617	154,572,307		MMGHRPVLVLSQNTKRESGRKVQSGNINAAKTIADIIRTCLGPKSMMKMLLDPMGGIVMT<br>NDGNAILREIQVQHPAAKSMIEISRTQDEEVGDGTTSVIILAGEMLSVAEHFLEQQMHPT<br>VVISAYRKALDDMISTLKKISIPVDISDSDMMLNIINSSITTKAISRWSSLACNIALDAV<br>KMVQFEENGRKEIDIKKYARVEKIPGGIIEDSCVLRGVMINKDVTHPRMRRYIKNPRIVL<br>LDSSLEYKKGESQTDIEITREEDFTRILQMEEEYIQQLCEDIIQLKPDVVITEKGISDLA<br>QHYLMRANITAIRRVRKTDNNRIARACGARIVSRPEELREDDVGTGAGLLEIKKIGDEYF<br>TFITDCKDPKACTILLRGASKEILSEVERNLQDAMQVCRNVLLDPQLVPGGGASEMAVAH<br>ALTEKSKAMTGVEQWPYRAVAQALEVIPRTLIQNCGASTIRLLTSLRAKHTQENCETWGV<br>NGETGTLVDMKELGIWEPLAVKLQTYKTAVETAVLLLRIDDIVSGHKKKGDDQSRQGGAP<br>DAGQE
**CCT6-1P**		Hs				TCP1 zeta<br>pseudogene 1	5	p15.2	-	1	14692965	14693954	Not identified in pseudogene.org	KDGNVLFHEMQIQHTTASLIAKVATAQDDITGDSTTSNVLIIEKLLKQEDLYISEGLHL<br>RIITEGFEAAKEKALRFLEEVKIRKEMDRETLINVARTSLHTKVHAELADALTEAVVDS<br>ILAIKRQDEPIDLFMVVIMEMKHKSETDTSLIRGLVLDHGAWHPDMKKRVEDVYILKCN<br>VSLEYEKT*VNSGFFYKTAEMREKLIKAERKFIEDKS*KIELKRKVCGDSDKGFVAINQ<br>EGIDPLSLDALAKEHIVTLHRAKRRNIEGLTLASGEVALNSFENLNPDCLGHAGLVYKY<br>ILGEEKFTFIEKCNNTRSVTLLNKGPNKYTLTQLQ
**CCT2**	TCP1 beta	Hs	NP_006422	NM_006431.2	ENSP00000299300	CCTbeta<br>(real gene)	12	q15	+	14	68,266,317	68,280,052		MASLSLAPVNIFKAGADEERAETARLTSFIGAIAIGDLVKSTLGPKGMDKILLSSGRDAS<br>LMVTNDGATILKNIGVDNPAAKVLVDMSRVQDDEVGDGTTSVTVLAAELLREAESLIAKK<br>IHPQTIIAGWREATKAAREALLSSAVDHGSDEVKFRQDLMNIAGTTLSSKLLTHHKDHFT<br>KLAVEAVLRLKGSGNLEAIHIIKKLGGSLADSYLDEGFLLDKKIGVNQPKRIENAKILIA<br>NTGMDTDKIKIFGSRVRVDSTAKVAEIEHAEKEKMKEKVERILKHGINCFINRQLIYNYP<br>EQLFGAAGVMAIEHADFAGVERLALVTGGEIASTFDHPELVKLGSCKLIEEVMIGEDKLI<br>HFSGVALGEACTIVLRGATQQILDEAERSLHDALCVLAQTVKDSRTVYGGGCSEMLMAHA<br>VTQLANRTPGKEAVAMESYAKALRMLPTIIADNAGYDSADLVAQLRAAHSEGNTTAGLDM<br>REGTIGDMAILGITESFQVKRQVLLSAAEAAEVILRVDNIIKAAPRKRVPDHHPC
**CCT71P**		Hs			ENST00000399032	CCTeta pseudogene 1<br>vert	5	q15	-	1	92251627	92307366	Not identified in pseudogene.org	AKTSMDIAKYQDAKVGDSTTSVTLLAAEFLKQVKPYVEE<br>GLHLKIIIQALRTAIQLAVNDNKEITVTMKKTEKVEQKKLLGEVCHPALSSKLISQQKAF<br>FAKMVVDAVMMLDGLLQLKMIGIKKIQGGALEDSHLLPGVSFK
**CCT8**	TCP1 theta	Hs	NP_006576	NM_006585.2	ENST00000389159	CCTtheta<br>(real gene)<br>vert	21	q21.3	_	15	29,350,670	29,367,782		MALHVPKAPGFAQMLKEGAKHFSGLEEAVYRNIQACKELAQTTRTAYGPNGMNKMVINHLEKLFVTNDAA<br>TILRELEVQHPAAKMIVMASHMQEQEVGDGTNFVLVFAGALLELAEELLRIGLSVSEVIEGYEIACRKAH<br>EILPNLVCCSAKNLRDIDEVSSLLRTSIMSKQYGNEVFLAKLIAQACVSIFPDSGHFNVDNIRVCKILGS<br>GISSSSVLHGMVFKKETEGDVTSVKDAKIAVYSCPFDGMITETKGTVLIKTAEELMNFSKGEENLMDAQV<br>KAIADTGANVVVTGGKVADMALHYANKYNIMLVRLNSKWDLRRLCKTVGATALPRLTPPVLEEMGHCDSV<br>YLSEVGDTQVVVFKHEKEDGAISTIVLRGSTDNLMDDIERAVDDGVNTFKVLTRDKRLVPGGGATEIELA<br>KQITSYGETCPGLEQYAIKKFAEAFEAIPRALAENSGVKANEVISKLYAVHQEGNKNVGLDIEAEVPAVK<br>DMLEAGILDTYLGKYWAIKLATNAAVTVLRVDQIIMAKPAGGPKPPSGKKDWDDDQND

**CCT6-4P**		Hs				Tcp1 zeta pseudogene 4	3	q28	+	5	191915332	191916879	Not identified in pseudogene.org	GPSAGGFGAQHQCGAGATGHTEDQPGTQGRHEDACFCAGHIKLTKDSHVPLHKMQIHEM<br>QMLLNAFLIAKVATAQDDITGDGMTYNVLIIRELLKQANLYISEGLHPRIITEGFEVER<br>PVLDHGARHPDVKKRVEDAYILKCNVLAKESIAALHRAKSRNMERLTLACGRVALIYFY<br>DLNPDCLGLVVLVHEYTLRRSSPLFSNFQVLVPWKWQWQKTSVKGSAQLGVQAFTDALL<br>NYSQGEPVVAAEIGVWDNYCVKKQLLHSCTVMATNILLVDEIMKAINRMSSLKG
**CCT6-2P**		Hs				Tcp1 zeta pseudogene 2	11	q22.3	-	1	109013584	109014117	Not identified in pseudogene.org	NSFDDLTPDYVGYAGLLCEYTFGEEKFTFIEKYNYPSSVTLLAKEPNKYTHTQIKD AVRDGLRAVK
**CCT6A**	TCP1 zeta, CCT6, Cctz, HTR3, TCP20, TCPZ, TTCP20	Hs	NP_001753	NM_001762.3	ENST00000275603	CCTzeta (real gene)	7	p11.2	+	14	56,087,036	56,098,269		MAAVKTLNPKAEVARAQAALAVNISAARGLQDVLRTNLGPKGTMKMLVSGAGDIKLTKDG<br>NVLLHEMQIQHPTASLIAKVATAQDDITGDGTTSNVLIIGELLKQADLYISEGLHPRIIT<br>EGFEAAKEKALQFLEEVKVSREMDRETLIDVARTSLRTKVHAELADVLTEAVVDSILAIK<br>KQDEPIDLFMIEIMEMKHKSETDTSLIRGLVLDHGARHPDMKKRVEDAYILTCNVSLEYE<br>KTEVNSGFFYKSAEEREKLVKAERKFIEDRVKKIIELKRKVCGDSDKGFVVINQKGIDPF<br>SLDALSKEGIVALRRAKRRNMERLTLACGGVALNSFDDLSPDCLGHAGLVYEYTLGEEKF<br>TFIEKCNNPRSVTLLIKGPNKHTLTQIKDAVRDGLRAVKNAIDDGCVVPGAGAVEVAMAE<br>ALIKHKPSVKGRAQLGVQAFADALLIIPKVLAQNSGFDLQETLVKIQAEHSESGQLVGVD<br>LNTGEPMVAAEVGVWDNYCVKKQLLHSCTVIATNILLVDEIMRAGMSSLKG
**CCT6-3P**		Hs				Tcp1 zeta pseudogene 3	7	q11.21	+	8	64162812	64171325	Not identified in pseudogene.org TCPZ3P & TCPZ5P are next to each other on chr. 7	MLLHFFSIFPLNHLKQIQHPTASLIAKVATAQDDITGDGTTSNVLIIGELLKQADLYISE<br>GLHPRIITEGFEAVKEKALHFLEEVKVSREMDKETLKDVARASLCTKVHAELADVLTEAV<br>VGSILAIKRKDEPIDLFMILCSFKEGIVALHRAKRRNMERLTLACGGVALNSFEDLSPDC<br>LGHAGLVYEYTLIKDAVRDGLRAVKNAVDDGCVVPGAGAVEVAMAEALNKYKLSVKGKAQ<br>LGVQAFADALLVIPKQQKQALWDNDCVKKQLLHSCTDCHQHSLG
**CCT6B**	Cctz2, TSA303, Tcp20)	Hs	Q92526	Q92526.4	ENST00000314144	CCTzeta (real gene)	17	q12	-	14	30,279,183	30,312,525		MAAIKAVNSKAEVARARAALAVNICAARGLQDVLRTNLGPKGTMKMLVSGAGDIKLTKDG<br>NVLLDEMQIQHPTASLIAKVATAQDDVTGDGTTSNVLIIGELLKQADLYISEGLHPRIIA<br>EGFEAAKIKALEVLEEVKVTKEMKRKILLDVARTSLQTKVHAELADVLTEVVVDSVLAVR<br>RPGYPIDLFMVEIMEMKHKLGTDTKLIQGLVLDHGARHPDMKKRVEDAFILICNVSLEYE<br>KTEVNSGFFYKTAEEKEKLVKAERKFIEDRVQKIIDLKDKVCAQSNKGFVVINQKGIDPF<br>SLDSLAKHGIVALRRAKRRNMERLSLACGGMAVNSFEDLTVDCLGHAGLVYEYTLGEEKF<br>TFIEECVNPCSVTLLVKGPNKHTLTQVKDAIRDGLRAIKNAIEDGCMVPGAGAIEVAMAE<br>ALVTYKNSIKGRARLGVQAFADALLIIPKVLAQNAGYDPQETLVKVQAEHVESKQLVGVD<br>LNTGEPMVAADAGVWDNYCVKKQLLHSCTVIATNILLVDEIMRAGMSSLK
**HSPD1-5P**		Hs				GroEL pseudogene 5	12	q13.2	+	2	55191053	55192769	Not identified in pseudogene.org	MLRLPTVFRQMRLVSRVLAPHLTGAYAKDVKFGADARALMLQGIDLLADAVALTMGPKGR<br>RTVIIEQSWGSPKVTKDGVTVAKSIDLKDKYKNIGAKLVQDVANNTNEETGDGPTTATVL<br>ARSIAKEGIEKISKGANPVEIRRGVMLVVDAVIAELKKQSKPVTTPEEIAQVATISANGD<br>KEIGNISDAMKKVGRKGVITVKDGKTLNDELEIIEGIKFDRGYISPYFINTSKGQKCEFQ<br>DAYVLLSEKKISSVQSVVPALEIANAHRKPLVIIAEDVDGEALSTLVLNRLKVGLQDVAI<br>KAPGFGDNRKNQLKDMAIATGGAVFGEEGLTLNLEDVQPHDLGNVGEVIVTKDDATLLKE<br>KGDKAQIEKRIQEIIEQLDVTTSEYEKEKLNERLAKLSDVVAVLKVGGTSDVEVNEKKDR<br>VRMPLMLQELLLKKALYWEGVVRTALLDAAGVTSLLTTAEVVVTEIPKEEKDPGMGAMGR<br>MGGGMGSGMF
**HSPD1-6P**		Hs				GroEL pseudogene 6	3	P22.3	-	4	36783612	36785195	Not identified in pseudogene.org	VDLLVDAVAITMGPKGRTVIIEQSWGSPKVTKDGVTVAKSIDLKNKYKNIEAKLVQDVAN<br>NTNEEAGDGPTTATVLACSIAKEGFEKISKGANPVEIRRGVMLAVDAVIAEPKKQSKPVT<br>TPEEIAQVATISASGDKEIGHILSDAVKKVGRKGVITVKDGKTLNDGLEIIESLKFDRGY<br>VSPYFINTSKGQKCEFQEAYVLLSEKKISSVQSIVPALETASAHRKPLVIITEDSRLQGF<br>VTKNQLKDMAVTTVGAVFGEEGLILHFEDVQPHDLGKVGEVIVTKDDAMLLKGKDGIAMP<br>KVGGTSDVEANEKKDRVTDALNATRAAVEEGIVLGGAMTIAKNAGVEGSSIVEKIMQRSS<br>EVGYDATRVGDFVNMVGKGIIDPRKVVRTALLDAAGVASLLTTAEVVVTEIPKEEKDPGM<br>GAMGGMGCGMGGGML
**HSPD1-7P**		Hs				GroEL pseudogene 7	8	p23.1	+	6	7263938	7265475	Not identified in pseudogene.org	MGPKGRTVIIEHSWGSPKVTKDGVTDAKSIDLKDKYKSIGAKLVQDVANNTDEETGGWHY<br>HCCCTGMLYFQIRLPEAVDAVIAELKKQSKPVTKPEEIAQVATISANGDKEIGNIISDAM<br>KKFGRKGIITKCEFQDAYVLLHEKKISSVQSIVTALEIANAYCKPLVIIAGDIDGEALTT<br>LILNRLKVGLQVVAVKAPGFGDNRKNQLKDTVIATGGEVGEVTVIKDYAMLLKGKGNKSQ<br>IEKCVQEIIDQSDVTTSEYEKEKVSGETFRWSSCAEGGCALLRCIPALDSFTPANEDKII<br>GIEIIKRTLKIPAMTIAKNAVGYDTMLGDVVNMVEKDIIDPTKVVRTASLDAAGMASLLT<br>TAAVVVTEIPKEGNSPGMGAMCGMGGGLF
**HSPD1-22P**		Hs				GroEL pseudogene 22	21	q21.3	-	3	29181851	29183334	Not identified in pseudogene.org	VDLLLTAVAITMGPKGKTVITEQNWGSPKVTKDGVTVARSIDLKDKYKNIAAKLVQDVAN<br>NTNEEAGDGTTTATVAKEVFKKFSKGANPVEIKRSVMLAVDAVIAELKKQSKPVTTPEEI<br>AQVATISANGDKEIGNLISDAMKKVRRKGFTTDIFLHTLLIHQKVGNVNSKKISSVQSIV<br>PDLEIANAHRKLLVIIAENVDGETLSTLILNRRKLGLQVVAVKAPGFGDNRKNHLKGMTI<br>ATGGAVFGEEELTSNLEDVQPHDLGEVGELDVTTSEYEKEKLNEQLAKLSDGVAVLKVSG<br>TSHVEVNEKKNRVTDALNATRAAVEEGIVLGGGCTLLR
**HSPD1-4P**		Hs				GroEL pseudogene 4	6	q15	+	1	88065673	88066269	Not identified in pseudogene.org	MTTPGEIAQVATISANGDKEIVNIISDANKFGRKGVLTVKDGKTLNDELEIIKGIKFD*GYISPY<br>FINTSKG*KCEFQDAYVVLNEMKISSVQTTVPAL*IANPHCKSLVIIAEDIDREARSTFIFNRLK<br>VGLQIIAVKAPDFGDNRKNQREDTAIATGGLVFSEEGLALNFEAI*PRNLEKVGEVILTKYDAML<br>LKGK
**HSPD1-8P**		Hs				GroEL pseudogene 8	4	q31.21	+	4	145986418	145987946	Not identified in pseudogene.org	CLSLNASRQSWGSPKVTKDSVTVAKSIDLKDKYKNTGAKLVQDVANNTNEEAVDGTTTVT<br>ALARSIAKEGFEKISKGANPVEIRRGVMLAVDAIIAEPKKQSKPVTTPEEIARVATISAN<br>GDKEIGNIISDAMKKVGSKGIITVNNGKSYISPYLINTSKGQKCEFQDAYVLLSEKKISS<br>VQSIAPALEIANAYFKDPGFGDNRNNQLKDMAIATGGAVFAEEGLTLNLEDVQPHDLGKV<br>GEVIVTKDDAMLLKGKGDKAQIEKCVQEIIEQLDVTTIAVLKVGGTSDAEVNEKQDRVTD<br>ALNATRAAVEEGIVLRGGRALLRCIPALDSLTPVNEDHNIGIEIIKKTLKFPAMTIAKNA<br>GVEVSLIVEKIMQSSSEVGYDAMGRDFVNMVEKGIIDTTKFVRTALLDASGVASLLTTAE<br>VLVTEIPKEEKDPGMGAMDGMGGGMGGGMF
**HSPD1-9P**		Hs				GroEL pseudogene 9	8	p23.1	-	5	7785932	7787502	Not identified in pseudogene.org,HSPD9P & HSPD7P are next to each other on chr. 8	CRLFVDAVAITMGPKGRTVIIEHSWGSPKVTKDGVTDAKSIDLKDKYKSIGAKLVQDKVS<br>KGANPVEIKRGVMLAVDAVIAELKKQSKPVTKPEEIAQVATISANGDKEIGNIISDAMKK<br>GYISPYFLNTSKGEKCEFQDAYVLLHEKKISSVQSIVPALEIANAYCKPLVIIAEDIDGE<br>ALTTLILNRLKVGLQVVAVKAPGFGDNRKNQLKDTPRDVGEVGEVTVIKDDAMLLKGKGN<br>KSQIEKCVQEIIDQSDVTTSEYEKEKLNGETFRWSSCAEGGCALLRCIPALDSFTPANED<br>KIIGIEIIKRTLKIPAMTIAKNAGVDGFLIVEKIMQSSSEVGYDTMLGDVVNMVEKDIID<br>PTKVVRTALLDAAGMASLLTTAAVVVTKIPKEGNSPGMGAMCGMGGGLF
**HSPD1-10P**		Hs				GroEL pseudogene 10	12	p13.31	+	4	8058884	8082857	Not identified in pseudogene.org	MLLPLPTVFPQMRLLSRVLAPHLTRAYAKDVKFGADARALMLQGVDLLADAVAVTMGPKG<br>RTVIIEQSWGSPQVTKDAKLVQDIANNTNEEAGNGTTSATVLARSIAKEGFKKISKGANP<br>VEIRKGVMLAVDAVIAELRKQSKFVTTPEEIAQVATTSANGDKEIGNIISNAMKKVGRKG<br>VITVKDGKTLNDELEIIESGWARCLMPSIVPALEIVNAHRKPLVIIAEDVDGEALSTFIL<br>NRLNVGLQVVAVKALLVTIEPAEEPGERYGYCYWWKPGYLIVTGRHTAGCLPGSVKPSRL<br>R

**HSPD1-11P**		Hs				GroEL pseudogene 11	5	q15	+	5	95130459	95132169	Not identified in pseudogene.org	MKILGLPSLSADETRLLGSSSHLGLCQTCKIWCRCLSLNSSRCRPFSRCRSCYSGAEGKN<br>YVASNTNEEAGDGTTTATVLALPMVKKVFEKITRGANPVKIRKGMMLAIDALIAELKKQS<br>KPMTTLEEIIQFAIISANRDKSATSFLKQLKAGPQVIAVKAPGLLTIERTSLKIWLLLLV<br>VQCLEKGVGLITQEKLERSLGPKVMLCSLKEMVTSLELKIVFKKSLSIAVLKVGVTSDVK<br>TNEKKDRVTDALNAIRAATEEDIILGWGCALLQFIPALDSLTPTNKNKKLLQQLCSVPQK<br>LVMSYGWGFCEYGGRGIIGPTKDVRTALLDAAWVASPLITAEVTEIPKEEKDPGMGAVGG<br>MGGGISDS
**HSPD1-12P**		Hs				GroEL pseudogene 12	13	q31.1	+	6	78321372	78323341	Not identified in pseudogene.org	LVSRVLKPHLPQADAKDVNFGADAQNLMLQDGVTVAKSIDLKDKYKNIGAKLVQNVANNT<br>NKEAGDGITTATILACSTAKKALRRLAKVLIQWKSEVWRQKKIGSIISGAMKKVGRKVVL<br>TVKDGKTLNDELEITEGGRAWLHFSMLINTSKGHKCEFQDAYVLLSEKNISGVQSIVPAL<br>EIASAYLKPLVTIAEDIDEETLSTLILNRLKVGLQVVAAKVSGFGDNGASLKIWLLLLLD<br>IITSGYEKKKLNEYLAKLSDGVAVLMVGETSDVQVKDKKDRFTDVLNATRAAIEDGIVLL<br>QCIPALDSLTPTNDDFFKCVEGSLIAEKIMQDSSEVGYDAMIGDSVNMVEKGIIDSTNIV<br>RTALLHAAEVASLLPTAEVVVIEIPKKENPGMSAIGGMGDGLF
**HSPD1-13P**		Hs				GroEL pseudogene 13	6	q25.2	+	1	153068626	153068943	Not identified in pseudogene.org	MEIIKRTLKIPAMTIAKNASVERSLIVEKIMQSSSEVGYDAMVRDFVNMVEKAIIDPTKI<br>VRAALSDAAGVASPLTTAEIVVTKIPKKREGPWNGCNGWNGRWYER
**HSPD1**	GROEL, HSP60, SPG13, CPN60, HuCHA60	Hs	NP_002147	NM_002156.4	ENSG00000144381	GroEL (real gene)	2	q33.1	_	11	198,060,018	198,071,817		MLRLPTVFRQMRPVSRVLAPHLTRAYAKDVKFGADARALMLQGVDLLADAVAVTMGPKGR<br>TVIIEQSWGSPKVTKDGVTVAKSIDLKDKYKNIGAKLVQDVANNTNEEAGDGTTTATVLA<br>RSIAKEGFEKISKGANPVEIRRGVMLAVDAVIAELKKQSKPVTTPEEIAQVATISANGDK<br>EIGNIISDAMKKVGRKGVITVKASDGKTLNDELEIIEGMKFDRGYISPYFINTSKGQKCE<br>FQDAYVLLSEKKISSIQSIVPALEIANAHRKPLVIIAEDVDGEALSTLVLNRLKVGLQVV<br>AVKAPGFGDNRKNQLKDMAIATGGAVFGEEGLTLNLEDVQPHDLGKVGEVIVTKDDAMLL<br>KGKGDKAQIEKRIQEIIEQLDVTTSEYEKEKLNERLAKLSDGVAVLKVGGTSDVEVNEKK<br>DRVTDALNATRAAVEEGIVLGGGCALLRCIPALDSLTPANEDQKIGIEIIKRTLKIPAMT<br>IAKNAGVEGSLIVEKIMQSSSEVGYDAMAGDFVNMVEKGIIDPTKVVRTALLDAAGVASL<br>LTTAEVVVTEIPKEEKDPGMGAMGGMGGGMGGGMF
**HSPD1-14P**	RP11-206L1.2	Hs			OTTHUMG00000016753	GroEL pseudogene 14	13	q13.3	_	4	37465288	37466827	Not identified in pseudogene.org,Processed pseudogene, vega transcript available, annotated in ENSEMBL.	MRTVSRVLAPYLTWAYAKDVKCGIDAQALMLQGAELLADAVAITMGSKGRAVIIQQSCGD<br>PKVTKDGVTVEKSIDLKDEYNSIGVKLVQDVANNTSEEAGDDTTTVLFWHALLPRMASRR<br>LVKVLIYWKWGESIVPALEVANAHHKTLVIIVEDIDGEALSTFVLNRLKNQLKDLAIATG<br>GAVFGDEGLALNLEYVQPRDLGKVGEVIVTKDYAMLLKGKGDKSQIEKKIFKKSWSKRQS<br>YRSLNAITAVEEGIVLGRGCALLQYVPALYSLTPASEDQNIGIEIKRIFKIAAMTIAKNA<br>GIEGSFIVKKITQSSIEVGYDAMVRDFVNMVGKGIIDPTKFLKTVLLDAFGWPLC
**HSPD1-15P**		Hs				GroEL pseudogene 15	5	p14.3	+	4	19269394	19270353	Not identified in pseudogene.org	MEPKRRIVIIEESWGRPKVTKDGVIVAHSINLEDKYENIGAKPVQDVSNNRIEEVEDCTT<br>TATVLACPIVKEREKLAKVLIQRKSGDRERNWQHISYTVNNAGRKGVITVNNGKTLNDEL<br>EIMEGMKFDQGYISPYFINTSKVYFTVNNMVKPLVITAENVLNSIKNKLKDKTTAISGEV<br>FEQEGFTLNLEVVEPHDLEEIGEVIATEDDAMLLKGNYGKAQIEKCIQDTQPVRCHN
**HSPD1-16P**		Hs				GroEL pseudogene 16	11	q22.3	+	3	105082802	105083755	Not identified in pseudogene.org	MNWELLKALSLIKVLFSHALYTSKGQRCEFQDTYILFNKKKICSIQFVVLALAIADAHRK<br>PLVIITEDDYGEALSTFILNRLKVCLQIIAVRTPGFDDNRKNQLNDIAIATGVIEQLNTT<br>IHKKESQNEHLGKLLDGIPVLKVHGTGNVKMNEKKELQNSSEVDYEAMFGDSPNVVEKKI<br>IDLAKAVRLLYHVLL
**HSPD1-17P**		Hs				GroEL pseudogene 17	1	p35.1	+	3	34077070	34078293	Not identified in pseudogene.org	MEGLVEISKGVNPVETRKGVMLCADTVITKLKKQPKPVTTPEEISPFTQCLQMETNKSAI<br>SFLMIKVGIWATVVKTPGFGDTERPNKAIAIMAWCLEESLTLKLEDVQFHDLGKVGAVIV<br>TKDDAMFLEGKAIAKNSGVEGSPTDSRENYAKLPRSWLCTTLRDFLKMVEKIIINSTKVV<br>KSALLDAASVDSQLTTAKLRPLEFLKKRRIWHG
**HSPD1-18P**		Hs				GroEL pseudogene 18	20	q13.32	+	5	56105684	56108736	Not identified in pseudogene.org	SFTKQGQTNTQPLTPTPKTLRPPTVLHQTRPASIPWPSSALDLHQRLKIWCLSEPNASRC<br>DSVAVAMGLNRRKIIIKQSGSRQTQSNKRCAELVQDTAFGTNEEAGMAAPGSVDKKSWQC<br>VVQWAPGEGVMEATHAVPAELRKQPVTTEEGKSLKNLLRLQMETKRKLHSIISGARRKGT<br>FSPPYVIDTSKSSKYEFQDACTHQSLEERYIPDLGINREEPCSASRLLQGGACDIWDWLQ<br>AEQRSQYISAEAERSTSHGLEPREEERISQPCPLLCICPVQASTASHLDQQQRP
**HSPD1-1P**	Human.chr5.mb135, Q96RI3	Hs			ENSG00000162241	GroEL pseudogene 1	5	q31.1	-	1	135744902	135745039	Identified in pseudogene.org	MVEKGISDPTKVVRPALLDAAGVASLLTAAQVAVTQIPKEETGLGMD
**HSPD1-3P**		Hs				GroEL pseudogene 3	20	q13.12	-	4	43602029	43602280	Not identified in pseudogene.org	MLPLPTVLCQMELMPR*LAPHLLRAYTKDIKLGTDAQALMLQGVTFNPML*L*Q<br>QGKTENRVGEVPK**KKLFTVAKSNDLKDK
**HSPD1-19P**		Hs				GroEL pseudogene 19	10	q11.23	+	1	50318868	50319008	Not identified in pseudogene.org	MVGTTILDADGMASLLTTAEAVVTEIPKEEKNPGMGGMGGGMRGGMF
**HSPD1-2P**		Hs				CCTeta pseudogene 2<br>vert	6	q25.1	+	1	150242815	150243240	Not identified in pseudogene.org	LKEGTDSSQGIPQLVNNLSTCQVIAKAVRATLGACGMDKLIVDGGGKATISND<br>VATILKLLHVVHLAAKTLVDIAKSQDTEVGDGTISVTLLAAEFLKQVKLQVKDG<br>LHPQLNIRAVCTATQLVVNTIKEIAVTVKKADKVE
**HSPD1-20P**		Hs				GroEL pseudogene 20	12	q21.31	-	1	78924341	78924478	Not identified in pseudogene.org	IIKPIMIVRTAPTDIAGVASLLSTAEFAITVISKENKKFAMDRMGV
**HSPD1-21P**		Hs				GroEL pseudogene 21	5	q12.1	-	6	60994430	60994876	Not identified in pseudogene.org	RPETLKVVPAAFEGGFFLRGGWAWFQRIPIWDS*TATNKDQKTDTEIVL<br>*NIQFKMPAVTLTRADG*EGSLTAENIV*SPSEVSCEALRGDLWRTG<br>ERMVGRGTGIIVTIMVGRAAASLPETEKKVSEDPKEKDPGTGRLG<br>GGGMGGSEF
**CCT4-2P**		Hs				CCTdelta pseudogene 2	7	q34	-	4	140344301	140345787	Not identified in pseudogene.org	IEAGDSTASVGTIAGSLIGSWAKLLQKGIHPTITSKSSQKSLEKGIEILSNISQPVELND<br>RETLLNSATSSLNSQVVFQYSSSASSDECKCSDESDPATGTTVNLRDINIQSCVILYPIV<br>KQYMTAVKKLGGKIDDCELVEGLILTQKVVNSGITQFEKAKIGHIQFCLSAPNRFGNQVA<br>VSDNNIEKEDVEFICKTIGTKPVARTDQVTLTCWVLLNAMEVIPTTLAENAGFESHFYSN<br>RTKKSTCLTRKKLQALVSKGQYFHFGKLVAQLL
**CCT3-1P**		Hs				CCTgamma pseudogene 1<br>vert	8	p22	+	2	16177578	16178178	Not identified in pseudogene.org	MLKIKKIGDEDFAFISKCKDPKVRTILLQGNGKETLSKMDHNIQDAVQVYCNFLFDPHLV<br>PGDGASEMAVIHVLTEKSKVMTGMNNGHTGLLPGLPSTPRRTMRPGEPLAVKLQTSRAAM<br>ETVVLLLWIDVITTGHKRKGKEQGQQGKTTGASKE
**CCT6-5P**	Human.chr7.mb64	Hs			ENSP00000275603	TCP1 zeta pseudogene 5	7	q11.21	+	10	64853564	64865440	Identified in pseudogene.org	MAAAKTLNPKAKVAGAQAALAFNISGARGLQDVLRTNLGPKGTIKILVSGAGDIKLTKDT<br>GVDDQPWPTCALLHEMQIQHPTASLIAKVATAQDDITGDGTTSNVLIIGELLKQADLYIS<br>EAGVQWHLGSLQRLSPGFKQFPASASSVARITGMRHHARLILYLSQRRGFSTCEGKALHF<br>LEEVKVSREMDKETLKDVARASLCTKVHAELADVLTEAVVGSILAIKRKDEPIDLFMILE<br>VNSGVFYKSAEREKLIKAERKFIEDRVKTNNRTEKESLYPDCLRHAGLVYEYTLKCNNPR<br>SVTLLIKGPNKPTLRSKMQLCGSGAGAVEVAMAEALNKYKLSVKGKAQLGVQAFADVLLV<br>IPKQQKQALWDNDCVKKQLLHSCTGCHQHSLG

**MKKS**	McKusick-Kaufman syndrome protein, BBS6	Hs			ENST00000347364	MKKS<br>(real gene)	20	p12.2	-	4	10,333,898	10,342,162		MSRLEAKKPSLCKSEPLTTERVRTTLSVLKRIVTSCYGPSGRLKQLHNGFGGYVCTTSQS<br>SALLSHLLVTHPILKILTASIQNHVSSFSDCGLFTAILCCNLIENVQRLGLTPTTVIRLN<br>KHLLSLCISYLKSETCGCRIPVDFSSTQILLCLVRSILTSKPACMLTRKETEHVSALILR<br>AFLLTIPENAEGHIILGKSLIVPLKGQRVIDSTVLPGILIEMSEVQLMRLLPIKKSTALK<br>VALFCTTLSGDTSDTGEGTVVVSYGVSLENAVLDQLLNLGRQLISDHVDLVLCQKVIHPS<br>LKQFLNMHRIIAIDRIGVTLMEPLTKMTGTQPIGSLGSICPNSYGSVKDVCTAKFGSKHF<br>FHLIPNEATICSLLLCNRNDTAWDELKLTCQTALHVLQLTLKEPWALLGGGCTETHLAAY<br>IRHKTHNDPESILKDDECTQTELQLIAEAFCSALESVVGSLEHDGGEILTDMKYGHLWSV<br>QADSPCVANWPDLLSQCGCGLYNSQEELNWSFLRSTRRPFVPQSCLPHEAVGSASNLTLD<br>CLTAKLSGLQVAVETANLILDLSYVIEDKN
**BBS10**	Bardet-Biedl syndrome 10	Hs	NP_078961	NM_024685.3	ENST00000313898	BBS10<br>(real gene)	12	q21.2	-	2	75,263,727	75,266,269		MLSSMAAAGSVKAALQVAEVLEAIVSCCVGPEGRQVLCTKPTGEVLLSRNGGRLLEALHL<br>EHPIARMIVDCVSSHLKKTGDGAKTFIIFLCHLLRGLHAITDREKDPLMCENIQTHGRHW<br>KNCSRWKFISQALLTFQTQILDGIMDQYLSRHFLSIFSSAKERTLCRSSLELLLEAYFCG<br>RVGRNNHKFISQLMCDYFFKCMTCKSGIGVFELVDDHFVELNVGVTGLPVSDSRIIAGLV<br>LQKDFSVYRPADGDMRMVIVTETIQPLFSTSGSEFILNSEAQFQTSQFWIMEKTKAIMKH<br>LHSQNVKLLISSVKQPDLVSYYAGVNGISVVECLSSEEVSLIRRIIGLSPFVPPQAFSQC<br>EIPNTALVKFCKPLILRSKRYVHLGLISTCAFIPHSIVLCGPVHGLIEQHEDALHGALKM<br>LRQLFKDLDLNYMTQTNDQNGTSSLFIYKNSGESYQAPDPGNGSIQRPYQDTVAENKDAL<br>EKTQTYLKVHSNLVIPDVELETYIPYSTPTLTPTDTFQTVETLTCLSLERNRLTDYYEPL<br>LKNNSTAYSTRGNRIEISYENLQVTNITRKGSMLPVSCKLPNMGTSQSYLSSSMPAGCVL<br>PVGGNFEILLHYYLLNYAKKCHQSEETMVSMIIANALLGIPKVLYKSKTGKYSFPHTYIR<br>AVHALQTNQPLVSSQTGLESVMGKYQLLTSVLQCLTKILTIDMVITVKRHPQKVHNQDSE<br>DEL
**BBS12**	C4orf24, FLJ35630, FLJ41559	Hs	NP_689831	NM_152618.2	ENSG00000181004	BBS12<br>(real gene)	4	q27	+	1	123,882,498	123,884,627		MVMACRVVNKRRHMGLQQLSSFAETGRTFLGPLKSSKFIIDEECHESVLISSTVRLLESL<br>DLTSAVGQLLNEAVQAQNNTYRTGISTLLFLVGAWSSAVEECLHLGVPISIIVSVMSEGL<br>NFCSEEVVSLHVPVHNIFDCMDSTKTFSQLETFSVSLCPFLQVPSDTDLIEELHGLKDVA<br>SQTLTISNLSGRPLKSYELFKPQTKVEADNNTSRTLKNSLLADTCCRQSILIHSRHFNRT<br>DNTEGVSKPDGFQEHVTATHKTYRCNDLVELAVGLSHGDHSSMKLVEEAVQLQYQNACVQ<br>QGNCTKPFMFDISRIFTCCLPGLPETSSCVCPGYITVVSVSNNPVIKELQNQPVRIVLIE<br>GDLTENYRHLGFNKSANIKTVLDSMRLQEDSSEELWANHVLQVLIQFKVNLVLVQGNVSE<br>RLIEKCINSKRLVIGSVNGSVMQAFAEAAGAVQVAYITQVNEDCVGDGVCVTFWRSSPLD<br>VVDRNNRIAILLKTEGINLVTAVLTNPVTAQMQIKEDRFWTCAYRLYYALKEEKVFLGGG<br>AVEFLCLSCLHILAEQSLKKENHACSGWLHNTSSWLASSLAIYRPTVLKFLANGWQKYLS<br>TLLYNTANYSSEFEASTYIQHHLQNATDSGSPSSYILNEYSKLNSRIFNSDISNKLEQIP<br>RVYDVVTPKIEAWRRALDLVLLVLQTDSEIITGHGHTQINSQELTGFLFL
**CCT5-2P**		Hs				CCTepsilon pseudogene 2 vert	13	q31.1	-	1	78382866	78382967		VTSAEDYKALQKYEKEKF*EMIQQIKETGANLAI
**Mkks**		Mm	EDL28392	CH466519.1		MKKS<br>(real gene)	2	qF3		4	136700005	136706971	Mus musculus (house mouse), repredicted in fgeneplus.	MSRLEAKKPSLCKTEPLTSEKVRSTLSVLKGVIASCYGPSGRLKQLHNGLGGCVYTTSQS<br>SALLRNLSVTHPVLKILTSSVQNHVSCFSDCGLFTAILCCNLIENIQRLDLTPATAIKLN<br>KYLLSLCTSYLKSEACSCRIPVDFRSTHTFLSLVHSILTSKPACMLTRKETDHIGALILK<br>AFLLTIPESTEERMVLGKSIIVPLKGQRVTDSTVLPGLLIEASEVQLRRLLPTQKASGLR<br>VALFCTSLSGDFSNAGEGVVVAHYQVSLENAVLEQLLNLGRRLVTDHVDLVLCQKVIHPS<br>LKQFFSERHVMAIDRVGVTLMESLSKVTGATPIGSLNPIVSTTYGSVKDVCSARFGSKHF<br>FHLLPNEATVCTLLLCSRNDTAWEELKLTCQTAMHVLQLTIKEPWVLLGGGCTETHLAAY<br>VRHKVHHEAEAIVRDDGCTQAKLHVAAEAFCSALESVAGSLEHDGGEILIDTKYGHLWSC<br>QADSASVGNWSDTLSRCGCGLYNSQEELSWSVLRSTYHPFAPQTCLPQAALGSASNLTVD<br>CFTAKLSGLQVAVETANLILDLSYVIEDKN
**Bbs10**		Mm	XP_125817			BBS10<br>(real gene)	10	qD1	+	4	110735779	110738219		MASQGSVTAALRVAEVLESIANRCVGPEGGQVLCTKPTGEVLLSRDGGCLLEALHLEHPLARMIVACVSS<br>HLKKTGDGAKTFIIFLCHLLRGLHAIGEKGKDSFTSENIQSHERHWKNCCQWKSISQALQTFQTQTLGCI<br>VDRSLSRHYLSVFSSSTEGRKLCRHSLELLLEAYFCGRVGRNNHRFISQLMCDYVFKCMACESGVEVFEL<br>LDHCFAELNVGVTGLPVSDSRIIDGLVLPRDFSMYCPADGDIRMVIVTEILQPQFSSAGSEFVLNSETQF<br>QASQCWITDRTKTVMNHLRGQNVKLLLTSVKQPDLVIYCARLNSISVVECLSAEEVSLVQRITGLSPCVL<br>PEVASQCEISDSTLVKFCKPLILRSKRYVHLGLISTCAFIPHSMVLCGPVLGLVEQHERAFHGAFKMLRQ<br>LFTDLDLNYIIQTKQQCNPSPLAYDNSRERNHSPETDKYQDIVAKSKNKLETQTHLEVYSGLGASDTELR<br>AGKPWSAHKKTPIAPSQTDEMLKCLPPERSGIIDNCDLSIENHSTGNPTAEDTGTEISFEHLQVSDNAGK<br>GYTLPVMRKSLDTCTCQGYCSSTVPAGCVLPVGGSFEILMSYYLLSYAKQCRQSDETVISMLIADALLGI<br>PKILYKPKKGKDSFPHIYMRSLHALQASQPMVSGQSGFESVAGKYQLLTSVLQCLMKILTIDLIINIKRQ<br>PQKTADQESEDEF
**Gm443**		Mm	NP_941023<br>XP_144240	NM_198621.2		CCTtheta_L1/L2<br>(real gene)	5	qA3	+	1	25022107	25023792	EST available	MAVSPPSRATQTKVQSDLELPQRLKPGLEKTPESQGEEPSYILRATAAAQTLASIIRSCYGPFGRQKFLV<br>TAKGETVCTGHAAAILKALDLEHPAAQFVQELAQTQVENAGDGTVFVVLLTEALLEQAHYLLWAGLTPTQ<br>LREAFATATAEVLTALPSLAIRSLGPLEDPSWALYSVISTHTLSNSDYLTKLVAQACWVSREPNGSFKPE<br>SIVVCVLQGGKLTDSRIFPGVAIAGKLCGQKTEVLGDARVALFNCPFGPTNPFTLATPRLSNPEELLRFR<br>KQTEQVEKEIAQLAIMDINVAVVLGEVNEKSVDQANYCGIMVIQAKSRKEIVYLSEKLGTPLLGRVLPPL<br>EPGKCHKVYRKEFGDTAVVMFEWEHEIAPFLSVVLRGPTIQGLRVAEQAVYYGIDAFSQLCQDPRLLPGA<br>GATEMALAKMLVDKGSRLSGPNGLAFQAFAQALSSLPKTLAENAGLAAQSVMAELSGFHQAGNFFVGVGT<br>DGLVNVTHEGIWDILRTKAQGLQAVAELVQQLVTVDQIIVARKTPLYRQITDPTLNAKVSSPLRAKFFGK<br>YV
**Bbs12**		Mm	NP_001008502	NM_001008502.2		BBS12<br>(real gene)	3	qB	+	1	37217982	37220105		MEMACRVINRRRHVGLQQLLSFAQTGRSFLGPVKATKFITDAECHESVLISSTVRLLEGL<br>DLTCAVGHLLNEAVQAQNNTYKIGTSTLLFLVGAWSRAVEDCLHLGIPTTVIVSVMSEGL<br>NSCIEAVVSLQVPIHNVFDHMDNTSTVYKLETVNATLCPFLQDPSGSGLLQEKRDFKDAT<br>SPLLSTYSLSGRHAESPKFFKPQNNLETEKNTLQVLKNNLYTDSFCKKSALAHSRHFNRT<br>DNSHWISRHDGFLEQLESTPKVLRCNDFGELAVGLSHGDHSSMALAKAAVRLQWQSLCLQ<br>QANWMAPFMFDISRLLTCCIPGLPETFSRVGLGYVTFVTMSSITLIKELQDQPFRVILIE<br>GDLTESYRHLGFNKSVNIKTKLDSGELSEDSAEELWTNHVLQVLIQFNVTLILVQGSVSE<br>HLTEKCMHSKRLVIGAVNGSVLQAFAEATRAVPVAYVTQVNEDCVGSGVSVTFWMSPHDI<br>NRSNRIAILLTAEGINLITAVLTSPASAQMETKEDRFWSCVYRLYHALKEEKVFLGGGAV<br>EFLCLSHLQILAEQSLNRGNHACLGWLPDSSSWMASSLSVYRPTVLKSLAGGWHEFLSAI<br>MCNTATHPSAVEARTFIQQHVQNAIDSGSPSSYILSEYSKLSSGVFHSGISDNLELVPRV<br>YDTVTPKIEAWRRALDVVLLVLQTDSEIITGLVHTEMNSQELDGVLFL
**Cct6a**		Mm	NP_033968			CCTzeta<br>(real gene)	5	F	+	13	130293315	130321693		MAAVKTLNPKAEVARAQAALAVNISAARGLQDVLRTNLGPKGTMKMLVSGAGDIKLTKDGNVLLHEMQIQ<br>HPTASLIAKVATAQDDITGDGTTSNVLIIGELLKQADLYISEGLHPRIITEGFEAAKEKALQFLEQVKVS<br>KEMDRETLIDVARTSLRTKVHAELADVLTEAVVDSILAIRKKDEPIDLFMVEIMEMKHKSETDTSLIRGL<br>VLDHGARHPDMKKRVENAYILTCNVSLEYEKTEVNSGFFYKSAEEREKLVKAERKFIEDRVKKIIELKKK<br>VCGDSDKGFVVINQKGIDPFSLDALAKEGIVALRRAKRRNMERLTLACGGIALNSFDDLNPDCLGHAGLV<br>YEYTLGEEKFTFIEKCNNPRSVTLLVKGPNKHTLTQIKDAIRDGLRAVKNAIDDGCVVPGAGAVEVALAE<br>ALIKYKPSVKGRAQLGVQAFADALLIIPKVLAQNSGFDLQETLVKVQAEHSESGQLVGVDLSTGEPMVAA<br>EMGVWDNYCVKKQLLHSCTVIATNILLVDEIMRAGMSSLKG

**Cct6b**		Mm	Q61390	Q61390		CCTzeta<br>(real gene)	11	B5	-	14	82532867	82577729		MAAIKIANPGAEVTRSQAALAVNICAARGLQDVLRPTLGPKGALKMLVSGAGDIKLTKDGNVLLHEMQIQ<br>HPTASIIAKVAAAQDHVTGDGTTSNVLIIGELLKQADLYISEGLHPRIITEGFDVAKTKALEVLDEIKVQ<br>KEMKREILLDVARTSLQTKVHAELADILTEAVVDSVLAIRRPGVPIDLFMVEIVEMRHKSETDTQLIRGL<br>VLDHGARHPRMRKQVRDAYILTCNVSLEYEKTEVSSGFFYKTVEEKEKLVKAERKFIEDRVQKIIDLKQK<br>VCAESNKGFVVINQKGIDPVSLEMLAKHNIVALRRAKRRNLERLTLACGGLAVNSFEGLSEECLGHAGLV<br>FEYALGEEKFTFIEDCVNPLSVTLLVKGPNKHTLIQIKDALRDGLRAVKNAIEDGCVVPGAGAVEVAIAE<br>ALVNYKHRVQGRVRLGIQAFADALLIIPKVLAQNSGYDLQETLIKIQTKHAESKELLGIDLNTGEPMAAA<br>EAGIWDNYCVKKHLLHSCTVIATNILLVDEIMRAGMSSLRD
**Cct1**	c-cpn, CCT, Cct1, Ccta, p63, Tcp-1, Tp63, TRic	Mm	P11984		ENSMUST00000089024	CCTalpha<br>(real gene)<br>vert	17	A2			13,109,331	13,117,933		MEGPLSVFGDRSTGEAIRSQNVMAAASIANIVKSSLGPVGLDKMLVDDIGDVTITNDGAT<br>ILKLLEVEHPAAKVLCELADLQDKEVGDGTTSVVIIAAELLKNADELVKQKIHPTSVISG<br>YRLACKEAVRYINENLIINADELGRDCLTNTAKTSMSSKIIGINGDYFANMVVDAVLAVK<br>YTDARGQPRYPINSVNILKAHGRSQIESMLINGYALNCVVGSQGMPKRIVNAKIACLDFS<br>LQKTKMKLGVQVVITDPEKLDQIRQRESDITKERIQKILATGANVILTTGGIDDMCLKYF<br>VEAGAMAVRRVLKRDLKCVAKASGATILSTLANLEGEETFEVTMLGQAEEVVQERICDDE<br>LILIKNTKARTSASIILRGANDFMCDEMERSLHDALCVVKRVLESKSVVPGGGAVEAALS<br>IYLENYATSMGSREQLAIAEFARSLLVIPNTLAVNAAQDSTDLVAKLRAFHNEAQVNPER<br>KNLKWIGLDLVHGKPRDNKQAGVFEPTIVKVKSLKFATEAAITILRIDDLIKLHPECKDD<br>KHGSYENAVHSGALDD
**Cct8**		Mm	NP_033970			CCTtheta<br>(real gene)<br>vert	16	qC3.3	-	15	87484236	87496033		MALHVPKAPGFAQMLKDGAKHFSGLEEAVYRNIQACKELAQTTRTAYGPNGMNKMVINRLEKLFVTNDAA<br>TILRELEVQHPAAKMIVMASHMQEQEVGDGTNFVLVFAGALLELAEELLRIGLSVSEVISGYEIACKKAH<br>EILPELVCCSAKNLRDVDEVSSLLRTSIMSKQYGSETFLAKLIAQACVSIFPDSGNFNVDNIRVCKILGS<br>GIYSSSVLHGMVFKKETEGDVTSVKDAKIAVYSCPFDGMITETKGTVLIKTAEELMNFSKGEENLMDAQV<br>KAIAGTGANVIVTGGKVADIALHYANKYNIMLVRLNSKWDLRRLCKTVGATALPKLTPPVQEEMGHCDSV<br>YLSEVGDTQVVVFKHEKEDGAISTIVLRGSTDNLMDDIERAVDDGVNTFKVLTRDKRLVPGGGATEIELA<br>KQITSYGETCPGLEQYAIKKFAEAFEAIPRALAENSGVKANEVISKLYSVHQEGNKNVGLDIEAEVPAVK<br>DMLEASILDTYLGKYWAIKLATNAAVTVLRVDQIIMAKPAGGPKPPSGKKDWDDDQND
**Cct1P**		Mm				CCTalpha<br>pseudogene	4	qC1	-	3	67963157	67964324	Not identified at pseudogene.org or, at ENSEMBL	MEDPLSLFGDCSSGEVICSHNVMAAASIVNIGKTSLGSVGLDKILVDDIGDITITNYDET<br>ILMLIIIAELLKNSDELVKQKINPTSVIRGYCRACKEAVCYIDENLIINTDKLGRDCLTN<br>AAKTSMSSKIIGINDDFFANMVVDTVLVAKYTDVRGQPLYPVNSVNVRKACGRSQIESML<br>INAYALSCVVGYPGMPKLITLRNWTKLDRIRYHQGENSEDPETGVHVILTTSGIDDMFLK<br>YFVESGAMSVRRVLKRDLTHMAKASRASILSLLANLEDEETFETTMLGQVKEVVQERICD<br>DELILIKNTKAHTCASVISCVLKQSTLYMVIFVW
**Cct71P**		Mm				CCTeta<br>pseudogene 1<br>vert	X	A1.3	+	5	12863162	12881543	Not identified at pseudogene.org or, at ENSEMBL	FYDKDSSGWSSSKDKDAYSSFGSRGDSRGKSSFFGDRGSGSRGRRDNLWIRTQALVSNIS<br>ACQVVAEAVRTTLGHCGMDKLIMDGRGKATISNDGATILKLLEIGDLSLKSSFSLKLPHP<br>ILPPRQDRGVHLTTPYTKWGLPLDVCDTRAKWQGTVSLSARYLLNQLSGGWLYLVPHVWC<br>HLTRPAGLVIRGSRGDLLLRTKWGGGRERRTPSGRNNTETSLISIKVSLAKTCTIILCSD<br>TEQFMEETERSLHDAIMIVRRAIKNDSVVAGGGAIEMELSKYLWDYSRTIPGKQQLLNGA<br>YAKALEIIPRQLCDNAGGMWYGVDINNEHIAGNFQAFVWEPAMVRINALTAASEAACLIV<br>SMDKSIKNSHSTVDPSAPTAGCGRGQAHFH
**Cct7**		Mm	P80313	P80313.1		CCTeta<br>(real gene)<br>vert	6	D1	+	11	85409109	85418268		MMPTPVILLKEGTDSSQGIPQLVSNISACQVIAEAVRTTLGPRGMDKLIVDGRGKATISN<br>DGATILKLLDVVHPAAKTLVDIAKSQDAEVGDGTTSVTLLAAEFLKQVKPYVEEGLHPQI<br>IIRAFRTATQLAVNKIKEIAVTVKKQDKVEQRKMLEKCAMTALSSKLISQQKVFFAKMVV<br>DAVMMLDELLQLKMIGIKKVQGGALEESQLVAGVAFKKTFSYAGFEMQPKKYKNPKIALL<br>NVELELKAEKDNAEIRVHTVEDYQAIVDAEWNILYDKLEKIHQSGAKVILSKLPIGDVAT<br>QYFADRDMFCAGRVPEEDLKRTMMACGGSIQTSVNALVPDVLGHCQVFEETQIGGERYNF<br>FTGCPKAKTCTIILRGGAEQFMEETERSLHDAIMIVRRAIKNDSVVAGGGAIEMELSKYL<br>RDYSRTIPGKQQLLIGAYAKALEIIPRQLCDNAGFDATNILNKLRARHAQGGMWYGVDIN<br>NENIADNFQAFVWEPAMVRINALTAASEAACLIVSVDETIKNPRSTVDPPAPSAGRGRGQ<br>ARFH
**Cct12P**		Mm				CCTalpha<br>pseudogene	17	qA1	+	2	13129036	13136541	Not identified at pseudogene.org or, at ENSEMBL	LSRIGLDLINGKPRDRHAGAFEPTIVKVKSLKRHRKVDLREFKVNLIYKTIL<br>RLYGETLTQKQRKTKKTKNNDQRKLHPESKDDKHGSYENAVHSGALDD
**Cct72P**		Mm				CCTeta<br>pseudogene 2	18	E4	+	5	87401390	87403113	Not identified at pseudogene.org or, at ENSEMBL	MSDIRACQVIAEKERITLGRHGMEKLTVDGPGIATNSNDGATVLKLLDVVHLATKTFVDI<br>SKSQDAEVGDGNTSMTLLVVEFLKQLTVNRIREIAVTVKKQNKIGQKRMLEKCAMTTLSS<br>KLISRQKAFFAKMAFDAVAMFDALLQPKYPGVNPSIASIALLNVELELKPEKDNAEIRVH<br>TVEGYQAIDAKVILSKLLIGDVAIQYFADRNKFCAVGVPEEDLKRMMKACGGSIQIIVDA<br>LIPQVLGCCLVFEEIQIEGERYNFFTTGPKDKTRTIIFCGGAEQFMEETKRVDINNEDIA<br>GNFQVFVREPAMVHINALTTAFEAACLIVSMDETI
**Cct3**		Mm			ENSMUSP00000001452	CCTgamma<br>(real gene)<br>vert	3	qF1	+	13	88103257	88125467		MMGHRPVLVLSQNTKRESGRKVQSGNINAAKTIADIIRTCLGPKSMMKMLLDPMGGIVMT<br>NDGNAILREIQVQHPAAKSMIEISRTQDEEVGDGTTSVIILAGEMLSVAEHFLEQQMHPT<br>VVISAYRMALDDMISTLKKISTPVDVNNREMMLSIINSSITTKVISRWSSLACNIALDAV<br>KTVQFEENGRKEIDIKKYARVEKIPGGIIEDSCVLRGVMINKDVTHPRMRRYIKNPRIVL<br>LDSSLEYKKGESQTDIEITREEDFTRILQMEEEYIHQLCEDIIQLKPDVVITEKGISDLA<br>QHYLMRANVTAIRRVRKTDNNRIARACGARIVSRPEELREDDVGTGAGLLEIKKIGDEYF<br>TFITDCKDPKACTILLRGASKEILSEVERNLQDAMQVCRNVLLDPQLVPGGGASEMAVAH<br>ALTEKSKAMTGVEQWPYRAVAQALEVIPRTLIQNCGASTIRLLTSLRAKHTQESCETWGV<br>NGETGTLVDMKELGIWEPLAVKLQTYKTAVETAVLLLRIDDIVSGHKKKGDDQNRQTGAP<br>DAGQE
**Cct31P**		Mm				TCP1 pseudogene	11	qC	-	5	90721084	90722575	Not identified at pseudogene.org or, at ENSEMBL	MLLDPMGGIVMTNDGNAILGEIQVQHPAVKSMIEISRTQDEEHFLEQQMHPTVVISAYRM<br>TLGDMINTLKKISTPVDVNNYEMMLNIINSSITTNKKNDIKKYSRVEKIPGGITEDSCIL<br>HGVIINKDVTHPVMSCYIKNPQIVLLNSSLEYKKVSQTDIEITRKEDFTRILQMKDECIQ<br>QLCEDIIQHRILFCDDLVITEKGISDLAQHYLIWVNVTATHRVWKTDNNHFARACSKACT<br>ILLRGASKEILSEVEHNLQDATQVCRNVQLDPQLVPGCGASEIAVAHDLTEKSKAMTGVE<br>QWPYRAVAQALEAMPRTWIQNCGASTICLLTSLRAKHTQEKQGIWEPLAVELQTYKTAVE<br>TEVLHLWIDDIICGHKKKGDDQNRQTRAPDADQE
**Cct4**		Mm	NP_033967	NM_009837.1		CCTdelta<br>(real gene)<br>vert	11	qA3.3	+	13	22890754	22902933		MPENVASRSGAPTAGPGSRGKSAYQDRDKPAQIRFSNISAAKAVADAIRTSLGPKGMDKM<br>IQDGKGDVTITNDGATILKQMQVLHPAARMLVELSKAQDIEAGDGTTSVVIIAGSLLDSC<br>TKLLQKGIHPTIISESFQKALEKGLEILTDMSRPVQLSDRETLLNSATTSLNSKVVSQYS<br>SLLSPMSVNAVMKVIDPATATSVDLRDIKIVKKLGGTIDDCELVEGLVLTQKVANSGITR<br>VEKAKIGLIQFCLSAPKTDMDNQIVVSDYAQMDRVLREERAYILNLVKQIKKTGCNVLLI<br>QKSILRDALSDLALHFLNKMKIMVVKDVEREDIEFICKTIGTKPVAHIDQFTADMLGSAE<br>LAEEVSLNGSGKLFKITGCTSPGKTVTIVVRGSNKLVIEEAERSIHDALCVIRCLVKKRA<br>LIAGGGAPEIELALRLTEYSRTLSGMESYCVRAFADAMEVIPSTLAENAGLNPISTVTEL<br>RNRHAQGEKTTGINVRKGGISNILEEMVVQPLLVSVSALTLATETVRSILKIDDVVNTR

**Cct2**		Mm	NP_031662	NM_007636.2		CCTbeta<br>(real gene)	10	qD2	-	14	116490071	116500106		MASLSLAPVNIFKAGADEERAETARLSSFIGAIAIGDLVKSTLGPKGMDKILLSSGRDAA<br>LMVTNDGATILKNIGVDNPAAKVLVDMSRVQDDEVGDGTTSVTVLAAELLREAESLIAKK<br>IHPQTIISGWREATKAAREALLSSAVDHGSDEARFWQDLMNIAGTTLSSKLLTHHKDHFT<br>KLAVEAVLRLKGSGNLEAIHVIKKLGGSLADSYLDEGFLLDKKIGVNQPKRIENAKILIA<br>NTGMDTDKIKIFGSRVRVDSTAKVAEIEHAEKEKMKEKVERILKHGINCFINRQLIYNYP<br>EQLFGAAGVMAIEHADFAGVERLALVTGGEIASTFDHPELVKLGSCKLIEEVMIGEDKLI<br>HFSGVALGEACTIVLRGATQQILDEAERSLHDALCVLAQTVKDPRTVYGGGCSEMLMAHA<br>VTQLANRTPGKEAVAMESFAKALRMLPTIIADNAGYDSADLVAQLRAAHSEGHITAGLDM<br>KEGTIGDMAVLGITESFQVKRQVLLSAAEAAEVILRVDNIIKAAPRKRVPDHHPC
**Cct5**		Mm	NP_031663	NM_007637.2	ENSMUST00000022842	CCTepsilon<br>(real gene)<br>vert	15	qB3.2	-	11	31520686	31531460		MASVGTLAFDEYGRPFLIIKDQDRKSRLMGLEALKSHIMAAKAVANTMRTSLGPNGLDKM<br>MVDKDGDVTITNDGATILSMMDVDHQIAKLMVELSKSQDDEIGDGTTGVVVLAGALLEEA<br>EQLLDRGIHPIRIADGYEQAARIAIQHLDKISDKVLVDINNPEPLIQTAKTTLGSKVINS<br>CHRQMAEIAVNAVLTVADMERRDVDFELIKVEGKVGGRLEDTKLIKGVIVDKDFSHPQMP<br>KKVVDAKIAILTCPFEPPKPKTKHKLDVMSVEDYKALQKYEKEKFEEMIKQIKETGANLA<br>ICQWGFDDEANHLLLQNGLPAVRWVGGPEIELIAIATGGRIVPRFSELTSEKLGFAGVVQ<br>EISFGTTKDKMLVIEKCKNSRAVTIFIRGGNKMIIEEAKRSLHDALCVIRNLIRDNRVVY<br>GGGAAEISCALAVSQEADKCPTLEQYAMRAFADALEVIPMALSENSGMNPIQTMTEVRAR<br>QVKESNPALGIDCLHKGSNDMQYQHVIETLIGKKQQISLATQMVRMILKIDDIRKPGESE<br>E
**Cct32P**		Mm				CCTgamma<br>pseudogene<br>vert	4	B3	+	3	52913471	52915552	Not identified at pseudogene.org or, at ENSEMBL	MMGHHLVLVLSLNTKRESGRKVQSGNINAAKTIADIIRTCLGPKSMMKMLWDPMGDIQMH<br>PTVVINAYRMALDDMISTLEKISTPVDVNNHEMMLNIINSSITTKVISRWSSLACTIALD<br>VVKTVQFEKKNRKKIDTKKYTRYPPELVSLAAYVAEDGLVSHRWEERPRGIANFICLSRG<br>ERQVQVGVEEYIQQLCEDIIQLKPDVVITEKGISDLAQHYLMRANVTAICRVWKTDNNHI<br>ARACRARIVDQSKELREDDVGTRAGLLEIKKIGDEYFTFITYYKDPKACTSLLRGASKEI<br>LSEVERNLQDVMQVCHNGLLDPQLVPGGGASEMAVADALREKSKAMTGVEQWPYRAVAQA<br>VEVIPQTLIQNCGTSTIHLLSSLRAEHTQESCKTWGAKDETGTLMDMKELGIWETLAVKL<br>QTYKTAVETEVLHLWIDDIASGHKKKGDDQNWQTSAPDAGQE
**Cct41P**		Mm				CCTdelta<br>pseudogene 1	7	qA3	-	3	26227193	26228371	Not identified at pseudogene.org	RGVCDVASMWRSESNLRESVLFCQDSCTKLVQKGRHPTIISESFQKASEKDLEILPDMSQ<br>PVPLSDRDTLLNSASTSLKSKVVSQYSSLLSPKVANSGTTGVEKAKIGHVPFCSSAPKTD<br>MDNQIVVSDYAQMDQVLREERAYILNLVKHVKKTGCDVLLIQRSILRDALSDLALHFLIT<br>GEEAIRSTPTGLNPISTVTELRNRHAQGENARCSNVQKAVLINILEEMVIQPLLVSVSAL<br>TLATEIAEHPEN
**Cct33P**		Mm				CCTgamma<br>pseudogene<br>vert	6	qE3	-	1	113303406	113303687	Not identified at pseudogene.org	MVLVQGQAYPIEIRDECTTFIPEHRDPEACTTLLRGAIEERLLEEECNLAGCHASVSRIL<br>LDPQLVLVVEPQRWLCPCLDRKIQGHDWYGTMAI
**Cct6A1P**		Mm				TCP1 zeta<br>pseudogene 1	8	qB1.3	+	3	52361244	52362111	Not identified at pseudogene.org	MECADVLIEAVVNSLEAFKVSDKPIVIFIFGIMHVKHKYKTYMRLITRFIFVSLECEKTE<br>VGSEFYYKREADKEKLLHGVETIEVTIKEFMIKYKSNLKARVQLRVLAFADVLHIIHKVL<br>AQGSGFDILNMLRSKLNTQNQISLGKKGLREMTVKQDKTRHKKSR
**Cct6A2P**		Mm				TCP1 zeta<br>pseudogene 2	14	qE2.1	-	2	97306133	97306902	Not identified at pseudogene.org	LHVFVLDHAAWYPDTEEGVEHSYICTCNVSLEYEQTDASPGVQSFVHAWLSNLKVRAQKF<br>SFNLQTLKFKVNIHNQINL
**Cct6A3P**		Mm				TCP1 zeta<br>pseudogene 3	18	qE3	+	1	79544122	79545468	Not identified at pseudogene.org	GQLVCVDLNLGEPRIAAEICIWSNSSLKKQLLHPCTVIASNTLKRAGILSL
**Hspd1**		Mm	P63038	P63038.1			1	qC1.2	-	11	55135135	55143783		MLRLPTVLRQMRPVSRALAPHLTRAYAKDVKFGADARALMLQGVDLLADAVAVTMGPKGRTVIIEQSWGS<br>PKVTKDGVTVAKSIDLKDKYKNIGAKLVQDVANNTNEEAGDGTTTATVLARSIAKEGFEKISKGANPVEI<br>RRGVMLAVDAVIAELKKQSKPVTTPEEIAQVATISANGDKDIGNIISDAMKKVGRKGVITVKDGKTLNDE<br>LEIIEGMKFDRGYISPYFINTSKGQKCEFQDAYVLLSEKKISSVQSIVPALEIANAHRKPLVIIAEDVDG<br>EALSTLVLNRLKVGLQVVAVKAPGFGDNRKNQLKDMAIATGGAVFGEEGLNLNLEDVQAHDLGKVGEVIV<br>TKDDAMLLKGKGDKAHIEKRIQEITEQLDITTSEYEKEKLNERLAKLSDGVAVLKVGGTSDVEVNEKKDR<br>VTDALNATRAAVEEGIVLGGGCALLRCIPALDSLKPANEDQKIGIEIIKRALKIPAMTIAKNAGVEGSLI<br>VEKILQSSSEVGYDAMLGDFVNMVEKGIIDPTKVVRTALLDAAGVASLLTTAEAVVTEIPKEEKDPGMGA<br>MGGMGGGMGGGMF
**Hspd1P**		Mm			ENSMUSG00000058809	GROEL pseudogene 1	11	qA5	+	1	41312239	41313954	Identified as pseudogene at ENSEMBL, not at pseudogene.org	MLRLPTVLRQMRPVSRALAPHLTRAYAKDVKFGADARALMLQAVDLLADAVAVTMGPKGR<br>TVIIEQSWGSPKVTKDGVTVAKSIDLKDKYKNIGAKLVQDVANNTNEEAGDGTTTSTVLA<br>RSIAKEGFEKISKGANPVEIRRGVMLAVDAVIAELKKQSKPVTTPEEIAQVATISANGDK<br>DIGNIISDAMKKVGRKGVITVKDGKTLNDELEIIEGMKFDRGYISPYFINTSKGQKCEFQ<br>DAYVLLSEKKISSVQSIVPTLEIANAHRKPLVIIAEDVDGEALSTMVLNRLKVGLQVVAV<br>KAPGFGDNRKNQLKDMAIATGGAVFGEEGLNLNLEDVQAHDLGKVGEVIVTKDDAMLLKG<br>KGDKAHIEKRIQEITEQLDITTSEYEKEKLNERLAKLSDGVAVLKVGGTSDVEVNEKKDR<br>VTDALNATRAAVEEGIVLGGGCALLRCIPALDSLKPANEDQKIGIEIIKRALKIPAMTIA<br>KNAGVEGSLIVEKILQSSSEVGYDARLGDFVNMVEKGIIDPTKVVRTALLDAAGVASLLT<br>TAEAVVTEIPKEEKDPGMGAMGGMGG
**Hspd2P**		Mm				GROEL pseudogene 2	4	qC7	-	1	105808716	105810383	Not identified at pseudogene.org	MRPVSPELALHLTQAYAKDGKFDVDAGALMLQGVDLLADAVAVTMGPKGRAVIIEQNWGS<br>PKVKNGVTVTKSIDLKDKYKNIRAKLVQDVANNTNEEAGDGTTATVLACSIANEGFEKIS<br>KGANSVEIQREIAQVATISANGGKEIGDINTDAKTKVGRKGVITGKDGKSLNDELEIIEG<br>MKLDREYISLYFINTSKGQKCEFQGAYVLLSEKKISSDWFTVPALEIANAHQKPLVIITE<br>DVDGEALSTLVLNRPKVGPQVVAVKAPGFGDNRKSQLKDMAIATGGAVFEEEGLKLNLKD<br>AQAHDLRKVVEVIATKDDAMLLKGKGDKAHIEKRIQEITEQLDITTSEYEKEKLKERLAK<br>LSDGVAVLKVGGTSDVEVNEEKDRVTDVLNATRAAVEEGIVLGGGCALRWCIPALDSLKP<br>ANEDQKIGIEVIKRALKIPAMTIVKNADAEGSLIVEQILQSSSEVGYEAMLGDFVNTVEK<br>GIIDLRKV
**Hspd3P**		Mm				GROEL pseudogene 3	14	qE2.3	+	5	101742761	101744429	Not identified at pseudogene.org	MRPVSWALTPLLTQAYAKDVKFGADAQVLMLQGVDLLASTLAVKMGPTGKTVTIKQSWGH<br>PKEKKRWEIAQVASISANGDKDIGNIISDAMKKAGRKGAITVVAIKAPGFGDNRKNQLKD<br>MAIATGDLGKVVEVIVTKDDAMLLKGKADKAHTEKRTQKITEHLDITTGDYEKEKLDERL<br>AKVSDGVAVLKVGGTSDVEVTEKKELQTVSLKATRAAVEEGIVLGRGCALLRCIPALDSL<br>NPANEDQKIGVGGSLIVEKILQSSSEVGCGTMLGDFVNMVENGIIDPTKV
**Hspd4P**		Mm				GROEL pseudogene 4	8	qB3.1	-	3	64965611	64967286	Not identified at pseudogene.org	MGPKRRTLIIEQSQGSPKVTKGEVTVTKPIDLKDKCKTIRSKLVQGVANNTNEEAGDGTT<br>TVSVLARSIANDFEKISKGKNPLEIRRGVMLAVSAVIAQLKKRSKPVTTPEEITQIATIS<br>ANRGKDFGNIISDAMRKEALVIIAEDGDRETLRTLVLNRLTVGLQVVAVKAPGFGDNRKN<br>QLKNMAIASGGVVFGEKGLNMNLEDVQAHDLGKVLEVIVTKNGAMLLKGKANNDKKIGIV<br>IIKGALKIPAMMIVKNADVEGPLIVEKILQSSSAVSYDTMLGDFVNMVEKGIIDPTKVVR<br>TALLDAAMVFSLLTT
**Hspd5P**		Mm				GROEL pseudogene 5	8	qC2	+	6	85271516	85279593	Not identified at pseudogene.org	MRLMSLALAPLLTQAYAKDVKFGADAQALMLQGVDLLANVVAVTMGPKGKTVIIKQSWEI<br>PKAKLVQDVANNTNEEAGYGTTTVSVLARSIAKEGFEKISKGANPLEIRRGGINCSGCYN<br>FSNGNKDIGNIISDAMKKVKHVNSKMPVLLSEKKISSVQSIVPAFEIANAHQKPLVIITE<br>DVDGEVSLPSPTSRKEAKKPMSFSMQRSSKCTGLEQAKRKVEEVIVTKDDAMLLKGKGDK<br>AHIEKRIQKITEQLYITTSEDEKEKLNERLAKLSDG

**Hspd6P**		Mm				GROEL pseudogene 6	1	qC1.1	-	5	49455871	49457564	Not identified at pseudogene.org	MRPVSPALAPHLTWAYAKDIKFVNFIKDKYKNIGHKLVQVVANNTNEEAGDGITTTTVLA<br>WSIAKEDFEKSKSVNSKMPMFLLSEMKFSSVQSIVSALKIVNAHQMPLVIIAEDIDRVAL<br>RSLILNKLKVCLQGLNQNLGDVHAHVIGKLGEVLFTKEDAMLLKGKGDKVHIEKHIQEIT<br>EQLDITTALDSLKPTNEDQKIGIEIIKRALKIPAMMIAKDAGVKGSLTVDKILQNSSEVG<br>YDAVLEDFVNMVEKGIIDLTKVIRTTLLDAAGVAFLLTTA
**Hspd7P**		Mm				GROEL pseudogene 6	3	qE3	-	3	77889757	77891210	Not identified at pseudogene.org	MGSRGRTVIIEESWGSTKVTKDGITEAKSIALQDEYKNTGAKLVQDVANNTKEGAGDGTT<br>YATVLKQSKPMAIPEEFAQVATISANGVKDTGNIISDAMKKVGRKWFILGKDGKTLNYEL<br>ENIEGKQFDRGYISPYFVKEKEKLNKQVAKLSDGVTVLKVGGTSDVEVNEKKDRVTDILS<br>ATGAAVEEGIVLGGGCALLRCVPALDSFKPSNEDQKIGIEIIKRALKIPPMAIAKNADDK<br>GSVIVEQILQSSSEVGYDIKLGDFFTWCKRESLIQQKFKNCLNGCCWGGLLAHYI
**Hspd8P**		Mm				GROEL pseudogene 7	4	qC4	-	5	89108911	89111283	Not identified at pseudogene.org	LALPLTGSYAKDVKFGVDVRALTLQNVDLLADVLAVTMGPKGRTVIIGQSWGSLKVTKDG<br>ACSTAKKAFERISKKANPVETQRGVMELVRFLPFLQMETSIGNIISDAMTKAGRKGVVTG<br>KDGKKSLDDELEVTEMKLDRGYIYPYFINISKGYIVPALKTANAHWKPLVIIVEDDDGEA<br>LSTMVLNMVKVGLQVVAVRAPGFGDNRKNQLKICGYCYWCVTRADVEESIVLREGCVLLQ<br>CMPVLDSLKPANGNQKIGIEITKGALNIPAMTMQR
**Hspd9P**		Mm				GROEL pseudogene 8	12	qA1.1	-	1	11672585	11673644	Not identified at pseudogene.org	SNSLTILKPLTTPEEIAQVAIISANGNKDVGNIISDVMKKGGRKGVITVKDGKSLNDKL<br>EIIKGMKFNRGYISPYFINTSKGQKCEFQNAYVLLSEKKFSSVQSIVPEIANAPRTPLVI<br>IAEDGDGEVVSTLVLNRLKVGLQVIAVKAPRFRDNRKNQLKFMAVTTGGVVSGEEGLHLN<br>LEDVQAHDIGKVGEVIVTKDDAMLLKGKGDKAHIEKCIQEIIEQVDITTGEYEKEKMNKQ<br>LPKLSDRVAVLK
**Hspd10P**		Mm				GROEL pseudogene 9	X	qC3	+	2	93743990	93745672	Not identified at pseudogene.org	DFANNTNKEAGDGTIIISVLAVSIAKDVSKVANPVEIQRCMMLSVDVVIAELKIQSKSVT<br>TPEEIDHIATISENGNKDIGNIISDATKKIKRLLTVKAPGFGDNKKNQLPDMATGGAVFK<br>EEALNLNLEDVKTHDLGKVGEVIVTKDDATLLKGKVDKVQIEKNGCKKSLGSYTSQLVII<br>KRES
**Hspd11P**		Mm				GROEL pseudogene 10	6	qA1	+	3	12515616	12517225	Not identified at pseudogene.org	DVANNINEEAGNGITIAIVLAQSIAKNGSEKISKGANPVKIWRGVILAVDAVIVVLKKQF<br>EPVATPEEISQVATSPANGDQVIENIILDAMNKVGRKGVITGKDIFPHILLTYQKSLVII<br>AKDVDREALYTPVSNRLKVGIQVVAIKASGLVDNRKNQLTDMLLPLVPDNEDQKIGIEII<br>KRILKIPAMTIAKNAGVEGPFIVEKILQSSSEVGYDAMLGDFVNVVENGIIDSTKV
**Hspd12P**		Mm				GROEL pseudogene 11	1	qC1.1	+	2	45234912	45235851	Not identified at pseudogene.org	MTTLEEIVQVAVISANGDKNIGNIISDAMKKVGRKGVITVKDGKILHDELEIIEGMKFEI<br>GCISTYFINTSKEALGDDPEDVDGETLSTLVLNRLIVGLQILAVKAPGFGDNKKNQLINM<br>AIATGGVMFGEED
**Hspd13P**		Mm				GROEL pseudogene 12	15	qA2	-	2	24133639	24135093	Not identified at pseudogene.org	MKHDRGYISSHFIDTYIGQYCEFQDAYALMSEKKIPSGVALLKVKVNEKKDRVTGAFNAV<br>RAVVEEGIILVRTRALLQCIPALDSLKPAIEDQKICIEIKEHSKFLHSSKVGCDPMLGDF<br>VNMVEKGIINSTKIVRLL
**Hspd14P**		Mm				GROEL pseudogene 13	2	qC3	+	1	79001216	79001752	Not identified at pseudogene.org	MRPVSRALALHLTWVYTKDVKFGVDARALMLQCVDLLADAVAVTMGPKGRTVIAEQSWQV<br>PKMLPIAQKKEAEDGITTATVLAHSMAKEGFKGFNPVEIQK
**Hspd15P**		Mm				GROEL pseudogene 14	9	qA1	+	1	12819585	12820103	Not identified at pseudogene.org	GVAVLKVGGRSDVEVTEKKDRVTDAFNAIRAAVEEGIVLGRGCSLLWCISALDSLKPAN<br>EVQKIGIEIIKRALKIPAMMIAKNAGIEGSLIEKIPQSSSKVSYGAMLGDFVHMVEKKII<br>DPTKA
**Hspd16P**		Mm				GROEL pseudogene 15	3	qE1	-	1	61872231	61873436	Not identified at pseudogene.org	MGFGEEGWNLNPEDVQTHVLGKVGEVIVTKDDDKLLKEKCDKIQFEKCIQEMTKQLEITI<br>SEYEKEKLNEQLAKSLSGVVVLKVQGT
**Hspd17P**		Mm				GROEL pseudogene 16	1	qA1	+	2	5053347	5054702	Not identified at pseudogene.org	DKLVQGVAINTNEEACDGTTIATVLSWSIANQGFEKISKVSKTVKIPEENIQVAMISANG<br>DKEFGNIVCDAMKMAGRMIIMTVMDGKTLNDELEIIEGMKSGIFPNISLIRGRYRTYSKR<br>LKIPAMTSAKNAVVEGPLVVEKILQSSVEFGYDAILGDFVNMVGRGIIEPTEV
**Hspd18P**		Mm				GROEL pseudogene 17	8	qB2	-	1	58011087	58011631	Not identified at pseudogene.org	MDSLKPANGDLKISIKIIKRALEVTAMTIAENVGVGGSLIIEEILQISSEVGYNAKLGDF<br>MNMIENGIIDQTKVLRSALLDASGVASLLTTGEAVVIEIPKKEMVPGMGAMAR
**Hspd19P**		Mm				GROEL pseudogene 18	16	qC1.3	+	1	61460831	61461298	Not identified at pseudogene.org	MIPKKTGVEGSWIDKKILQISSEVGYDTLLGDFVNMVEKGIIDTTKVVRPALLDAARVAS<br>LLTTVNAVVTEAPKEEKDTGTSAMGGMGV
**Hspd20P**		Mm				GROEL pseudogene 19	2	qE1	+	2	98441180	98441466	Not identified at pseudogene.org	DQERGIEIIKRTLKIPAMMIAKNAEVGYDAILGDFVNIVEKGIIDPTKV
**Hspd21P**		Mm				GROEL pseudogene 20	4	qC5	+	2	91375464	91376660	Not identified at pseudogene.org	MPLKGKGKGDKAQIERCTQECIPAFTLLNPANENQKIGIEITKRLLKISAMTAAMSI
**Cct81P**		Mm				CCTeta pseudogene 1	2	qC1.1	+	1	51795721	51795882	Not identified at pseudogene.org	MTHQTHYERSGSASPCCTNDIMASLTQQQEVGVMSAHRVNNTVLVTLTAHPSISISHDSS<br>NFNNEHIRVLLDDIKGEENLIDAQSNTTVGSGEDVIITGDKVANMDLHYASKYNTINRTF<br>LQGMEGTEFASIKIEMRGWLHETTHVKEGAKHITPYGEPRPGLEQYTRKKLIVAFEAIIW<br>ALSEISRVKANDW
**Cct73P**		Mm				CCTeta pseudogene 3	1	qA4	+	4	20811426	20812537	Not identified at pseudogene.org	TTLGPHGNLLWMAKAKQQFLLLKLLDGGHPAAKTLVDIAGSEDTDTSDGTTVVILLAEEF<br>LKQSGFEMQPKNYENPTIAFSNVELKLKAEKDNAEIRVHTHQAILDAKRNILYDKLVIYF<br>ADRNMFCAGHWPEEDLKRTMMACGGSTQTNGNALIPDVLGHSQVFKETQIRGKRCNFFTG<br>FPKAKTCTTILC
**Cct74P**		Mm				CCTeta pseudogene 4	11	qB1.2	-	1	48381857	48382517	Not identified at pseudogene.org	MKPTPVILIKEGTDSSQGIPQLVSNKSACQVIVEAVRTTLGHHGMDKLIVDGRGKATISN<br>DGATILKLPNFLKHVKPYVEEGLHPQIIIHTATQLAVNKIIAIAVTVKKQDKVEQRKMLE<br>KCAVTALSSKMISQQKAFFTKMEPAMVHINTLTAASEAACLIVSMDETIKNPRST
**Cct75P**		Mm				CCTeta pseudogene 5	13	qB1	+	1	58647550	58647741	Not identified at pseudogene.org	NTFSSAKFETRPKESKNAKIALLNVELELKAEKDNAEIRVHT
**Cct82P**		Mm				CCTeta pseudogene 2	3	qB	-	3	35774568	35782271	Not identified at pseudogene.org	PGVQLLNVTLFVTHDEACSLIWRHILYLMEGNGKYIGQQIISYGDTYPGLPQYAFRKFTE<br>LFEAIPWALALDQGIFAVKDMLEAGAVSLHLGKYKAAHGPYCCKPCNDQSGHHPKPAGGS<br>KPPCGKKKTGMMTKKI
**Hspd22P**		Mm				GROEL pseudogene 21	17	qC	-	1	48192945	48193097	Not identified at pseudogene.org	KFGADARALMLQGVDLLADAVAVTMGPTGRTVIIEQRWGSPK
**Mkks**		Rn	NP_001008354	NM_001008353.1		MKKS<br>(real gene)	3	3q36	-	4	124975607	124981825		MSRLEAKKPSLCKTEPLTSERVRSTLSVLKGIIASCYGPSGRLKQLHNGLGGCVCTTSQSSALLRNLSVT<br>HPILKVLTSSVQNHVCCFSDCGLFTAILCCNLIENIQRIGLTPTTAIKLNKYLLGLSISYLKSEACSCRI<br>PVDFRSTHTFLNLVHSILTSKPACMLTRKEIDHIGALILKAFLLTIPESAEERMVLGKSIIVPLKGQQVT<br>DSTVLPGLLIEASEVQLRRLLPTQKSSTLRVALFCASLSGDFSNAGEGTLVVHYQVSLENAVLEQLLNLG<br>RQLVSDHVDLVLCQKVIHPSLKQFLSEHQIIAIDRVGVTLMEPLSKVTGATPIGSLYPIVSTTYGSVKDV<br>RSARFGSKYFFHLLPNEATICSLLLCSRNDTAWEELKLTCQTAMHVLQLTIKEPWVLLGGGCVETHLAAY<br>IRHKVHNEAEAIVRDDGCSQAELHIATEAFCSALESAAVSLEHDGGEILIDMKYGHFWSCPADSASVGNW<br>PDTLSRCGCGLYNSQEELSWSVLRSTYHPFAPQTCLPQAASGSVSNLTVDSFTAKLSGLQVAVETANLIL<br>DLSYVIEDKN

**60748**	RGD1560748_predicted	Rn				BBS10<br>(real gene)	7	q21	+	2	50317911	50320406		MASQGSVTAALRVAEVLETIANRCVGPEGGLVLCTKPTGEVLLSRDGGCLLEALHLEHPLARMIVACVSS<br>HLKKTGDGAKTFIIFLCHLLRGLHAIGEKEKDSFTSEDIQSHERHWKNCCQWKSISRALLRFQTQTLGCI<br>VDQHLSRHYLSAFSSSAEGRTLCRRSLELLLEPYFCGRVGRNNHRFISQLMCDYVFKCMACESGIEVFEF<br>LDNCFVELNVGVTGLPVSESRIVDGLVLPRDFSVYCPADGGIRMVIVTEILQPLFSSSSSEFVLDSETQF<br>QASQSWIMDRTKTVMNHLRSHNVKLLLSSVKQPDLVTYCARLNSISVVECLSSEEVSLVQRITGLSPCVL<br>PELASQCHIADSALVRFCKPLIVRSGRYAHLGLVSTCAFVPHCVVLCGPVLGLVQQHESAFHGAFKMLRQ<br>LFTDLDLNCMIQTKERRNPSPLADSSNRESSHSPKTGKYQDVVAKNKDKLETQTRLEVYPRLGASDTELI<br>TCKLWSAHKETSIDPSQTNEIPKRLSPEKSRIVDNCEVFLENNPTGNPTAEDTRTEMSFKHLQVADNPGE<br>GYTLPVTYKSPDTCPSQAHCSSAVPAGCVLPVGGHFEILMHFYLLNYAKQCRQSDDAVISMLIANALLGV<br>PKILYKPKKGKDSFPHIYTRALRALQTRQPIVSGQSGFESVAGKYQLLTSVLQCLMKILTIDLIINVKRQ<br>PQKTGDQESEDEL
**125233**	MGC125233	Rn	NP_001032883<br>XP_575319			CCTtheta_L1/L2<br>(real gene)	4	q11	-	1	5004090	5005772		MAVSSSQVTQTKVQSDLELPQRLKLGLEKNPESQGEEPLCILRATAAAQTLASIIRSCYGPYGLQKFLVS<br>AQGETVCTGHAAAILKALELEHPAARFVQELAQTQAENTGDGTAFVVLLTEALLEQAQYLLWAGLTPAQL<br>REAFVTATAEVLTALPSLAICSLGPLEDPSWALYSVMSTHTLSNAEYLTKLVAQACWISREPNGSFKPES<br>IVVCILQGGILTDSRIIPGIAICGKLCGRKTEVLNDARVALFNCPFGPSNPFAPATLRLSSPEELIRFRK<br>QTEQVEMEIAELAMMGINVAVVLGEVNERSVDQADYCGVMVIQVKSRKEIVYLSDKLGVPLLNRILPPLE<br>PGKCHKVYRMEFGESALIMFEWEREIAPFLSVVLRGPTIQGLRGAEQAVYYGIDAFSQLCQDPRLLPGAG<br>ATEMALARMLVDKGSRLDGPNGLAFQAFAQALSSLPKTLAENAGLAAQSVLAEMSGYHQAGNFVIGVGTD<br>GLVNVAQEGIWDILRTKAQGLQAVTGLVQQLVTVDQIIVARKTPRYRLIPQSAQNANTSSPLRAKFFGKY<br>E
**61608**	RGD1561608_predicted	Rn	XP_345203			BBS12	2	q25	+	1	123866726	123868843		MEMDYRVLNRRRHVGLQQLSSFAQTGRSFLGPVKATKFITDAECHESVLVGSTVRLLEGL<br>DLTCAVGHLLNEAVQAQNVTYKTGASTLLFLVGAWSRAVEDCLHLGVPTTVIVSVMSEGL<br>NSCIEAVVSLQVPIHNVFDHIDNTSTVYKLETVDVSLCPFLQVPSGSGLLEEKHDFKDAT<br>SQLLSTYSLSGRRAKSPEFFKPQAKVETENTSQALKNNLYTDSFCRKSALTHSRHFNRTE<br>NSHWISRPDGFLEHLRSTPKVLRCNDLGELAVGLSHGDHSSMTLAKAAVRLQWQAVCLQP<br>ANCMAPFMFDISRLLTCCLPGLPETFSCVCLGYVTSVTMPSITLIKELQDQPFRVILIEG<br>DLTESYRHLGFNKSVNIRTKSDSGQLSEDSTEELWTNRVLEVLIQFNVNLILVQGSVSEH<br>LTEKCMHSKRLVIGSVNGRVLQAFAEATRAVPVAYVTQVNEDCVGNGVSVTFWTGPHDIN<br>RSNREILLTAEGINLITAVLTSPASAQMEMKEDRFWSCVNRLCHALKEEKVFLGGGAVEF<br>LCLSHLQILAEQSLNKGNHACLGWLPDSSSWMASSLSVYRPTVLKCLAGGWHEFLSAIMC<br>NTATYPSAVEASTFIQHHVQSAADSGSPSSYILSEYSELSSGLFHSDISNNLELVPRVYD<br>TVTPKIEAWRRALDLVLLVLQTDSEIITGLVHTQMNSQELDGVLFL
**Cct6a**		Rn	NP_001028856<br>XP_213765	NM_001033684.1		CCTzeta<br>(real gene)	12	12q13	+	14	27992366	28002203		MAAVKTLNPKAEVARAQAALAVNISAARGLQDVLRTNLGPKGTMKMLVSGAGDIKLTKDGNVLLHEMQIQ<br>HPTASLIAKVATAQDDITGDGTTSNVLIIGELLKQADLYISEGLHPRIITEGFEAAKEKALQFLEQVKVS<br>KEMDRETLIDVARTSLRTKVHAELADVLTEAVVDSILAIRKKDEPIDLFMVEIMEMKHKSETDTSLIRGL<br>VLDHGARHPDMKKRVENAYILTCNVSLEYEKTEVNSGFFYKSAEEREKLVKAERKFIEDRVKKIVELKKK<br>VCGDSDKGFVVINQKGIDPFSLDALAKEGIVALRRAKRRNMERLTLACGGIALNSFDDLNPDCLGHAGLV<br>YEYTLGEEKFTFIEKCNNPRSVTLLVKGPNKHTLTQIKDAIRDGLRAVKNAIDDGCVVPGAGAVEVALAE<br>ALIKYKPSVKGRAQLGVQAFADALLIIPKVLAQNSGFDLQETLVKVQAEHSESGQLVGVDLNTGEPMVAA<br>EMGVWDNYCVKKQLLHSCTVIATNILLVDEIMRAGMSSLKG
**L63658**	LOC363658	Rn	NP_001014250<br>XP_343949	NM_001014228.1		CCTzeta<br>(real gene)	10	q26	-	11	72107176	72144991		MAAIKIANPGAEVTRSQAALAVNICAARGLQDVLRPSLGPKGALKMLVSGAGDIKLTKDGNVLLHEMQIQ<br>HPTASIIAKVAAAQDHITGDGTTSNVLIIGELLKQADLYISEGLHPRIIAEGFDVAKTKALEVLDKIKVQ<br>KEMKREMLLDVARTSLRTKVHTELADILTEAVVDSVLAIRRPGIPIDLFMVEIVEMRHKSETDTQLVRGL<br>VLDHGARHPRMKKQVQDAYILICNVSLEYEKTEVSSGFFYKTVEEKEKLVKAERKFIEDRVQKIIDLKQK<br>VCAESNKGFVVINQKGIDPVSLEMLAKHNIVALRRAKRRNLERLTLACGGLAVNSLEDLSEECLGHAGLV<br>FEYTLGEEKFTFIEDCVNPLSVTLLVKGPNKHTLIQIKDALRDGLRAVKNAIEDGCVVPGAGAVEVAIAE<br>ALVNYKHCVQGRARLGIQAFADALLIIPKVLAQNSGYDLQETLIKIQTKHAESKELVGIDLNTGEPMVAA<br>EAGIWDNYCVKKHILHSCTVIATNVLLVDEIMRAGMSSLRE
**Tcp1**		Rn	NP_036802			CCTalpha<br>vert	1	q11	-	12	42103629	42111145		MEGPLSVFGDRSTGEAIRSQNVMAAASIANIVKSSLGPVGLDKMLVDDIGDVTITNDGAT<br>ILKLLEVEHPAAKVLCELADLQDKEVGDGTTSVVIIAAELLKNADELVKQKIHPTSVISG<br>YRLACKEAVRYINENLIINTDELGRDCLINAAKTSMSSKIIGINGDFFANMVVDAVLAVK<br>YTDIRGQPRYPVNSVNILKAHGRSQIESMLINGYALNCVVGSQGMLKRIVNAKIACLDFS<br>LQKTKMKLGVQVVITDPEKLDQIRQRESDITKERIQKILATGANVILTTGGIDDMCLKYF<br>VEAGAMAVRRVLKRDLKRIAKASGASILSTLANLEGEETFEATMLGQAEEVVQERICDDE<br>LILIKNTKARTSASIILRGANDFMCDEMERSLHDALCVVKRVLESKSVVPGGGAVEAALS<br>IYLENYATSMGSREQLAIAEFARSLLVIPNTLAVNAAQDSTDLVAKLRAFHNEAQVNPER<br>KNLKWIGLDLVHGKPRDNKQAGVFEPTIVKVKSLKFATEAAITILRIDDLIKLHPESKDD<br>KHGGYENAVHSGALDD
**Cct8**		Rn	XP_213673			CCTtheta<br>(real gene)<br>vert	11	q11	-	15	27234460	27245292		MALHVPKAPGFAQMLKDGAKHFSGLEEAVYRNIQACKELAQTTRTAYGPNGMNKMVINRLEKLFVTNDAA<br>TILRELEVQHPAAKMIVMASHMQEQEVGDGTNFVLVFAGALLELAEELLRIGLSVSEVITGYEIACKKAH<br>EILPDLVCCSAKNLRDVDEVSSLLRTSIMSKQYGSEEFLAKLISQACVSIFPDSGNFNVDNIRVCKILGS<br>GVYSSSVLHGMVFKKETEGDVTSVKDAKIAVYSCPFDGMITETKGTVLIKTAEELMNFSKGEENLMDAQV<br>KAIAGTGANVIVTGGKVADMALHYANKYNIMLVRLNSKWDLRRLCKTVGATALPKLTPPVLEEMGHCDSV<br>YLSEVGDTQVVVFKHEKEDGAISTIVLRGSTDNLMDDIERAVDDGVNTFKVLTRDKRLVPGGGATEIELA<br>KQITSYGETCPGLEQYAIKKFAEAFEAIPRALAENSGVKANEVISKLYSVHQEGNKNVGLDIEAEVPAVK<br>DMLEASILDTYLGKYWAIKLATNAAVTVLRVDQIIMAKPAGGPKPPSGKKDWDDDQND
**Cct1P**		Rn				CCTalpha<br>pseudogene 1	4	q34	-	1	122326746	122328035	Not identified at pseudogene.org	EAVRYINENLIINTDELGRDCLINAAKTSMSSKIIGINGDFFANMVVDAVLAVKYTDIRG<br>QPRYPVNSVNILKAHGRSQIESMLINGYALNCVVGSQGMLKRIVNAKIACLDFSLQKTKM<br>KLGVQVVITDPEKLDQIRQRESDITKERIQKILATGANVILTTGGIDDMCLKYFVEAGAM<br>AVRRVLKRDLKRIAKASGASILSTLANLEGEETFEATMLGQAEEVVQERICDDELILIKN<br>TKARTSASIILRGANDFMCDEMERSLHDALCVLKGVLESKSVVPGGGAVEAALSIYLENY<br>ATSMGSREQLAIAEFARSLLVIPNTLAVNAAQDSTDLVAKLRAFHNEAQVNPERKNLKWI<br>GLDLVHGKPRDNKQAGVFEPTIVKVKSLKFATEAAITILRIDDLIKLHPESKDDKHGGYE<br>NAVHSGALDD
**Cct2**		Rn	NP_001005905<br>XP_216891	NM_001005905.1		CCTbeta<br>(real gene)	7	q22	-	9	56394922	56402261	Not identified at pseudogene.org	MASLSLAPVNIFKAGADEERAETARLSSFIGAIAIGDLVKSTLGPKGMDKILLSSGRDAS<br>LMVTNDGATILKNIGVDNPAAKVLVDMSRVQDDEVGDGTTSVTVLAAELLREAESLIAKK<br>IHPQTIIAGWREATKAAREALLSSAVDHGSDEVKFWQDLMNIAGTTLSSKLLTHHKDHFT<br>KLAVEAVLRLKGSGNLEAIHVIKKLGGSLADSYLDEGFLLDKKIGVNQPKRIENAKILIA<br>NTGMDTDKIKIFGSRVRVDSTAKVAEIEHAEKEKMKEKVERILKHGINCFINRQLIYNYP<br>EQLFGAAGVMAIEHADFAGVERLALVTGGEIASTFDHPELVKLGSCKLIEEVMIGEDKLI<br>HFSGVALGEACTIVLRGATQQILDEAERSLHDALCVLAQTVKDPRTVYGGGCSEMLMAHA<br>VTMLASRTPGKEAVAMESFAKALRMLPTIIADNAGYDSADLVAQLRAAHSEGRITAGLDM<br>KEGSIGDMAVLGITESFQVKRQVLLSAAEAAEVILRVDNIIKAAPRKRVPDHHPC
**Cct21P**		Rn				CCTbeta<br>pseudogene 1	X	q31	+	2	94540595	94541059	Not identified at pseudogene.org	ECILKHGINCFVSRQLIYNYPEEIFSTAGVIAIEHADFGGVEFLALVTGKLMQFPGLAVG<br>DVCTIVLHGATQDILDEAERTLYDALCVLAQIVKDPRTVYGGGCLELLMAHVVTKLASGT<br>PGKEV

**Cct3**		Rn	NP_954522<br>XP_215611	NM_199091.1		CCTgamma<br>(real gene)<br>vert	2	q34	+	12	180383106	180433933	Not identified at pseudogene.org	MMGHRPVLVLSQNTKRESGRKVQSGNINAAKTIADIIRTCLGPKSMMKMLLDPMGGIVMTNDGNAILREI<br>QVQHPAAKSMIEISRTQDEEVGDGTTSVIILAGEMLSVAEHFLEQQMHPTVVISAYRMALDDMVSTLKKI<br>STPVDVNNRDMMLNIINSSITTKVISRWSSLACNIALDAVKTVQFEENGRKEIDIKKYARVEKIPGGIIE<br>DSCVLRGVMINKDVTHPRMRRYIKNPRIVLLDSSLEYKKGESQTDIEITREEDFTRILQMEEEYIQQLCE<br>DIIQLKPDVVITEKGISDLAQHYLMRANVTAIRRVRKTDNNRIARACGARIVSRPEELREDDVGTGAGLL<br>EIKKIGDEYFTFITDCKDPKACTILLRGASKEILSEVERNLQDAMQVCRNVLLDPQLVPGGGASEMAVAH<br>ALTEKSKAMTGVEQWPYRAVAQALEVIPRTLIQNCGASTIRLLTSLRAKHTQENCETWGVNGETGTLVDM<br>KELGIWEPLAVKLQTYKTAVETAVLLLRIDDIVSGHKKKGDDQNRQTGAPDAGQE
**Cct31P**		Rn				CCTgamma<br>pseudogene 1<br>vert	19	p11	+	3	21483523	21485110	Not identified at pseudogene.org	MVGHCPVLLLSQNTQRVSGRKVQSGNINTAKTIADIIWTCLEPKSIMKMLLDPARGIVMT<br>NDEVGDGTTSVIILAGEMLSVAEHFLEQQMHPTVVISAYHMALDDMINTLKKISIPVDVN<br>NRDMMLNIINSSITTKVISRWSSLACNIALDAVKTVQFEENGRKEIAMKKYARVEKIPRG<br>IFEDSCVLRRVMINKDVTHPRMHRYIKNPRIVLLVSFLDLCSWIVSPPEELREDDVGTGA<br>GLLEIKKIGEEYFTFITDCKDPKACIILLRGASKRDTIGSRTQPPGCLASVHNVLLDPQL<br>VTGGGASEMAVAHALTQKSKAMTGVEQWPYRALAQALEVIPQTLIQNCGASTIRLLTSLR<br>AQHTQENCETWGVNGATGTLCGLERAGYLGAIGHEATNIQNSSGGCSSASADEDIVSGHK<br>KKGDDQNRQTSAADAGQE
**Cct32P**		Rn				CCTgamma<br>pseudogene 2<br>vert	13	q13	-	4	51648197	51649727	Not identified at pseudogene.org	NTKRESGRKVQSGNVNAAKRSANIIWTCLEPKSMMKMLLDPMMTNDEMLSVSKHFLQQQM<br>HPTVVISAYRMALDGMISTLKQISTPVDGKNHDVVLNIINSSITTKSSVTGGFLLNPANE<br>RRLHLQLCKDIIQLKDDVVITENGISDLVQHYLMWANVTAIRRVRKTDNNHIARACGART<br>RSQRRDNIGSRTQPPGCHASVRNVLLDPQRVPSGHVEQWSYRAVAQALEVIPLTLIQNYG<br>ASTIRLLISLRAKHTQENCEIWGVKSKT
**Cct33P**		Rn				CCTgamma<br>pseudogene 3<br>vert	X	q31	+	3	93409548	93410990	Not identified at pseudogene.org	IQVQHPAAKSIIEISGTQDEVARDGTISVIILVGEMLSVAEQFLEQQTHSTVVSSAYHMA<br>LDDMLSTLKQINSPVDVNDRDMMLNIINSSIITKVIRLWSSLAYNIALDAVKIIGLCYWI<br>LLWNTKQEKARVSLRLHERRISPESCKWEKSTLSSCDAMQVCCNVLLDPYLMPSDGASEM<br>AVAHAVTEKAKARTVMEPWPYRAVAQALE
**Cct34P**		Rn				CCTgamma<br>pseudogene 4<br>vert	4	q42	-	2	149237097	149237635	Not identified at pseudogene.org	AISDLAQHHLVWANVTATPRVWKTVNNDAARACGARIVSAETQRLAPLFLEEPARKYFWK<br>KSAPWQDAMQVCHSILLDPQLVPGSGASEMAHALIEKPKAMTG
**Cct4**		Rn	NP_877966	NM_182814.2		CCTdelta<br>(real gene)<br>vert	14	q22	+	13	103675244	103687376	Not identified at pseudogene.org	MPENVASRSGPPAAGPGNRGKGAYQDRDKPAQIRFSNISAAKAVADAIRTSLGPKGMDKMIQDGKGDVTI<br>TNDGATILKQMQVLHPAARMLVELSKAQDIEAGDGTTSVVIIAGSLLDSCTKLLQKGIHPTIISESFQKA<br>LEKGLEILTDMSRPVQLSDRETLLNSATTSLNSKVVSQYSSLLSPMSVNAVMKVIDPATATSVDLRDIKI<br>VKKLGGTIDDCELVEGLVLTQKVANSGITRVEKAKIGLIQFCLSAPKTDMDNQIVVSDYAQMDRVLREER<br>AYILNLVKQIKKTGCNVLLIQKSILRDALSDLALHFLNKMKIMVVKDIEREDIEFICKTIGTKPVAHIDQ<br>FTPDMLGSAELAEEVSLNGSGKLFKITGCTSPGKTVTIVVRGSNKLVIEEAERSIHDALCVIRCLVKKRA<br>LIAGGGAPEIELALRLTEYSRTLSGMESYCVRAFADAMEVIPSTLAENAGLNPISTVTELRNRHAQGEKT<br>TGINVRKGGISNILEEMVVQPLLVSVSALTLATETVRSILKIDDVVNTR
**Cct5**		Rn	NP_001004078<br>XP_215516	NM_001004078.1		CCTepsilon<br>(real gene)<br>vert	2	q22	-	11	83681899	83692935		MASVGTLAFDEYGRPFLIIKDQDRKSRLMGLEALKSHIMAAKAVANTMRTSLGPNGLDKMMVDKDGDVTV<br>TNDGATILSMMDVDHQIAKLMVELSKSQDDEIGDGTTGVVVLAGALLEEAEQLLDRGIHPIRIADGYEQA<br>ARIAIQHLDKISDNVLVDINNPEPLIQTAKTTLGSKVVNSCHRQMAEIAVNAVLTVADMERRDVDFELIK<br>VEGKVGGRLEDTKLIKGVIVDKDFSHPQMPKEVLNAKIAILTCPFEPPKPKTKHKLDVTSVEDYKALQKY<br>EKEKFEEMIAQIKETGANLAICQWGFDDEANHLLLQNGLPAVRWVGGPEIELIAIATGGRIVPRFSELTS<br>EKLGFAGVVREISFGTTKDKMLVIEQCKNSRAVTIFIRGGNKMIIEEAKRSLHDALCVIRNLIRDNRVVY<br>GGGAAEISCALAVSQEADKCPTLEQYAMRAFADALEVIPMALSENSGMNPIQTMTEVRARQVKESNPALG<br>IDCLHKGSNDMQYQHVIETLIGKKQQISLATQMVRMILKIDDIRKPGESEE
**Cct51P**		Rn				CCTepsilon<br>pseudogene 1	X	q32	-	2	111419580	111419580	Not identified at pseudogene.org	KDQDCKSGLLEFEALKSHIMAAKAVAKTTQMSLGPNTLNMMIVDKGGDVTVNNEDANILS<br>MIDVDHEIAKLRVELSKSQDDENRYGTTRMDVLKHLDKISNNVLVDINNPESLGSKVVNS<br>CHQQMTEISVNSIITVADTELRDVDFELIKWDSKILGWLENTRLIKHVIIDKDFSHPQMS<br>GKKMVDSNISILMCHFEPAKQKKRHKL
**Cct52P**		Rn				CCTepsilon<br>pseudogene 2	8	q13	-	1	17626224	17626583	Not identified at pseudogene.org	RSLIHYNRVVYGGGAAEISCALGVSQEVDKCPTLEQYAMRTFADALEVIHMALSENGDMNPFQTMTEVLARQVEESNPA
**Cct6A1P**		Rn				CCTzeta<br>pseudogene 1	8	q13	-	5	31685049	31688384	Not identified at pseudogene.org	MAAVKTLNPKAEVACVQAALAVNICLARGLQDVLRTNLGPKGTMKMLVSGLHPRIITEGF<br>EAAKEKALQFLEQVKVSKEMDRETLIDVARTSLRTKVHAELADVLTEAVVDSILAIRKKD<br>EPIDLFMVEIMEMKHKSETDTSLIRGLVLDHGARHPDMKKRVENAYILTCNVSLEYEKTE<br>VNSGFFFFFNKSAEEREKLVKAERKFIEDRVKKSCSWKEGIVALHRAKRRNMERLTPACG<br>GIALNSFDDLNPDCLGHAGLFYEYTLVHDGGRRASSSSSVREMGDSGSSTEKFTFIEKYN<br>NPRSVTLLVKGPNKHTLTQIKDVIRDGLRAVKNAIDDGCVVPGAGAAEAFADTLLIIPKV<br>LAQNSGFDLQETLVKAEHSESGQLVGVDLNTGEPMVAAEMGVWDNYCVKKQLLHSCTVIT<br>TNILL
**Cct6A2P**		Rn				CCTzeta<br>pseudogene 2	10	q32.1	+	2	88702370	88703903		AVNISAAGGLQDVLRTNLRPKGTMKMLVSGAGDTKLTKDGNVLLNEMPIQHPIASLIAKV<br>ATAQDDISGDGTTSSVLIIRYLLKQVDLYISEGLHPRIITEGFKAAKEKALQFLEQVKVS<br>KEMDRKTLINVARTSLWTKVHAELADVLTEAVVASILAIGKKDERIDLFMVQIMEMKHKS<br>ETDTSLIRGLKKVCGDSDKAFVVINQKGIDPFSLDALAKEGIVALCRAKRRHMERMTLAC<br>SGIALNSFDDLNPDCLGHAGLVYEYTLGEEKFTFIEKCNNPSSVTLLVKGPNKHMLTQIK<br>DAIRDGLRAVKNATDDGCVVPGAGAEVALAEALIKYKPSVKDRAQLGVQAFADALLIIPK<br>VLVQNSGFDLQETLVKVQAEHSEFGQLVRVDLNTGEPMVATDMGVWDNYCVKKQLLHSCT<br>VVVTKFLL
**Cct6A3P**		Rn				CCTzeta<br>pseudogene 3	5	q11	+	3	240279	241905		MAVVKTLNPKAEVARVQAALVVNISAAPGLQDVLRTNLGPKGTVKMLVSGAGDIKLTKDG<br>NVLLYEMFFFYLQQIQHPTASLIAKVATVQDDIIDDDTTFNVLIIGELLKQADLYISEGL<br>HPRIITESFETAKEKARQFLKQVKVNKEMDRETLIDVARTSLRTKVHAEFADVLTETVVY<br>SILAIRKKDEPIDLFLVEIMEMKHKSETDTNLIRGLVVDHQARHSDMKKRVENAYILTCN<br>VSLDAEEREKLVKAERKFIEDRVKKIIVLKKKVCGDSDKGFVVINLKGIDPFSLDALADK<br>GILPLRRAKRRNMERLTLDCGPNKHMLTQIKDAIRDGLGGVKMLLMMAVLSRWRCSRSGT<br>AFADALLIISKVLAQNSGFDLQETLVKVQAEHSETGQLVGVDLNTGEPMVAAEIGVWYNY<br>YVKKQLLHSCTVIATNILLVNEIMRAG
**Cct6A4P**		Rn				CCTzeta<br>pseudogene 4	5	q24	-	3	76957850	76959406		MKMLVSGSGDIELTKDGNVLLHEMQIQHPTASLIAKMATAQGDITGDGTTSNVLIIGSCS<br>NKCLYRGYGGLHFGHWEKEEPTELFMVETMEMKHKYETDTSLIRGLVLDHGAWHPDMKKR<br>VENAYILTGNSAEEREKLVKAERKLIENRVKKIIELKKKVCGDLDKGFLIINQKGIDPFS<br>LDALAKEGIVALCRAKRRKADSCLCAGAAEVALAEALIKYKPSVKGRTQLGVQAFADALL<br>IIPKVLLQNSGFGLQETLVKVHAEHLESGQLVGVDLNTGEPMVASEMGVWDNYCVKKQLL<br>PSCTLIATNILLVDKIMRAGMSSLK

**Cct6A5P**		Rn				CCTzeta pseudogene 5	18	p11	-	4	35236443	35237988		MMAVKTLNPKAEVAGAQAVLVVNSSAAWGLQDVLRTNSGLKGTVKMFVSGAGDIKCTKDG<br>NDDITGDGTTSNVLIIGELHKQEDLYVSEGLHPRIISEGFEAAKEKALQFLEQVKVSKEM<br>DRETLIDIKDLLLIKRVLTPFLCKRRYHSSAQSQEEKHGKLTLACGGIALNSFDDLNLDC<br>LGHAGLVYEYTLGKEMFTCIEKCSNPCSVTLLVKGPNKHMLTQIKDTIRDGLRAVKNAID<br>DGCVVPVVPGAGAEVALAEALIKYKPSVKGRAQLGVQACTDALLIIPKVLAQNSGFDLQK<br>TLKFKLNIQNLVS
**Cct6A6P**		Rn				CCTzeta pseudogene 6	1	q12	-	5	67148659	67170130		MAVVKTLNPKTEVAREQAALVVNSSAALVLQDDTMKKLVSGAEDIKLTKDGNVLIHEMQI<br>QHPTASLIAKVATAQDDITGDGTISNVLIIRELLKQIDLYVSEGLHPRIISEGFEAAKEK<br>ALQFLEQVKVSKEIDRETLINMARVSLLQFMLNLLGLVLDHGVWHPDMKKRVESAYILTC<br>NVSLEYEKTEGNSGSFTRIELKEIELKKKVCGDSDKGFVVINQKGIDPFSLDALVNEAMN<br>LSVDLNPDCLGHAGLVYEYTLGEEKFTVMKKRDNLQSVTLLAKGPNKHTLTQIKDAIRDG<br>LRAVKMLLMMAVLSWVQVLVQKSSFDLQEKRVKVKAEHSESCQLVGVDLSTGEAMVATEM<br>SDWDNYCVKKQLLHSCTVMATNILLVDEIMRAG
**Cct6A7P**		Rn				CCTzeta pseudogene 7	1_random		-	5	1252980	1254555		MKKLVSGAEDIKLTKDGNVLIHEMQIQHPTASLIAKVATAQDDITGDGTISNVLIIRELL<br>KQIDLYVSEGLHPRIISEGFEAAKEKALQFLEQVKVSKEIDRETLINMARKRVESAYILT<br>CNVSLEYEKTEGNSGSFTRTQEEKHGKADTCVGIAMNLSVDLNPDCLGHAGLVYEYTLGE<br>EKFTVMKKRDNLQSVTLLAKGPNKHTLTQIKDAIRDGLRAVKMLLMMAVLSWVQVLVQKS<br>SFDLQEKRVKVKAEHSESCQLVGVDLSTGEAMVATEMSDWDNYCVKKQLLHSCTVMATNI<br>LLVDEIMRAG
**Cct6A8P**		Rn				CCTzeta pseudogene 8	13	q22	-	5	79050104	79053455		VVRAQTALAVNISTAWGQQDVLRTNLRPKGTVKMPVSGAGDTKLTKDDNTVDLYISKGLH<br>PRITEGFEAAKEKALQFLEQVKGSKEMDRGTLIDVAKTSLRTKVHAELADVLTGAVTMEV<br>KHKSETDTSLARGLALDLVAQVSLEYKKTEMPLERRYHSSVQSQEEKHGKLTLACGGIAL<br>NSFDNLNPDGLGHSGLAYKYTLKRSSPVLRSVTIPVLSVYWCSRSGPGRGSRIKYKPSVK<br>DRAQLGVQACADALLIIPKVLAQNSAFDLQETLKFKLNIQNPVSLS
**Cct6A9P**		Rn				CCTzeta pseudogene 9	16	q12.5	-	4	88811021	88812383		MVAVKTLNPKADVARTQAALAVNISEALGVQDVLRSNLGSKGTMKTLISGARDIKNSKDS<br>NVLLHEMQIQHPIASLIAKVATAQDCITGNGTTSNVLIIWELLQQVDIYISKGLHPRIIT<br>GGLEIAKEKNSKLWWTPFWLLGKKDEPIDLSMVENMEMKHKSETDTNLISRLFLDHGMEL<br>KKIIELKKKVCGDSDKGFVIINQKGTNPFYLDALLKEVTLLIKGPNNHMLTQIKDAIRDG<br>LRAIKNAIDDGCVVSGAGAVGLALAETLINYKPSVKGRAQLGVQAFADTLLIFPK
**Cct6A10P**		Rn				CCTzeta pseudogene 10	1	q12	-	3	67148933	67150124		LGPKDTMKKLVSGAEDIKLTKDGNVLIHEMQIQHPTASLIAKVATAQDDITGDGTISNVL<br>IIRELLKQIDLYVSEGLHPRIISEGFEAAKEKALQFLEQVKVSKEIDRETLINMARVSLL<br>QFMLNLLGLVLDHGVWHPDMKKRVESAYILTCNVSLEYEKTEGNSGSFTRIELKEIELKK<br>KVCGDSDKGFVVINQKGIDPFSLDALVNEAMNLSVDLNPDCLGHAGLVYEYTLGEEKFTV<br>MKKRDNLQSVTLLAKGPNKHTLTQIKDAIRDGLRAVKMLLMMAVLSWVQ
**Cct6A11P**		Rn				CCTzeta pseudogene 11	6	q23	+	5	83591559	83592798		SVGLHPRIITEDFEAAKQKALQCVEQVKVSTEMHREMLIDVARISLQSKVHAEFSDVLTE<br>AVIMEMKHKSETDTSLITGLVLDHGAKHPDMKKKIELIKIVELKKKVCGNSDKEFVIINQ<br>KGIDPFSLDTLVKEGLIYEYTLCEEKFNFIEKCNCSRSLTLLVNGPNKHTLTEIKVAIRD<br>GLRAVKNAVDDGCVVCVQLVGVDLNTGERMVAAEMGMWDNYCVKKQLLHPCTVIATNILL<br>VDEIMRAG
**Cct6A12P**		Rn				CCTzeta pseudogene 12	9	q22	-	4	50508448	50509665		RIITEGFEAAKERALQFLEQVKASKEMNRKTLIDVAKTSLQSKVHVELADVLTEAVVNSI<br>LAIRKRDEPTYLFVVEIMEMNHKSETDTSLIKGLNLDHGAGHPDMKKRVDNAYILMCNSE<br>EEKEKLVKAERTFIDDTVKNIIDLNKKLCTEPKGETWKADTSCGGIALNFFANLTPDCLG<br>HGGLVYEYIMGPTKSMLTEIKDSIRDGLRDVKNAIDDGHRCNRSGTGRRSDQIQTQCEGQ<br>GLAWGPGICRHLAYHSQAETMMATEMGLWDNYCVNKQLLHSCIIITTNFLLVEEIMRAG
**Cct6A13P**		Rn				CCTzeta pseudogene 13	14	q11	-	1	66456610	66457113		EKCNNPHSVTLPVKGPNKHMLTQIKDALRDGLRAVNNVIDNGSVVPGTDALLIIPKVLEQ<br>NSGFDLQETLVKVQAEHCKSNQLVDVDLNTSELMVVTEMS
**Cct6A14P**		Rn				CCTzeta pseudogene 14	1	q12	-	1	67168555	67168845		VLVQKSGFDLQEKRVKVKAEHSESCQLVGVDLNTGEAIVATEMSEWDNYCVKKQLLHSCT<br>AMATNILLVDEIMRAG
**Cct6A15P**		Rn				CCTzeta pseudogene 15	5	q11	+	2	8728585	8728815		MVAAEMGVWDNYCVKKQLLHSCTVIATNILLVDEIMRAGMSSLKG
**Cct6A16P**		Rn				CCTzeta pseudogene 16	18	q12.3	+	1	76283952	76284215		VLAQTSSSDLWETLVMIQTEHSELSRLVCVDLNTGEPRVVAEVSVS
**Cct7**		Rn	NP_001100073<br>XP_001073942<br>XP_216180	NM_001106603.1		CCTeta<br>(real gene)<br>vert	4	q34	+	11	119714400	119722770		MMPTPVILLKEGTDSSQGIPQLVSNISACQVIAEAVRTTLGPRGMDKLIVDGRGKATISNDGATILKLLD<br>VVHPAAKTLVDIAKSQDAEVGDGTTSVTLLAAEFLKQVKPYVEEGLHPQIIIRAFRTATQLAVNKIKEIA<br>VTVKKQDKVEQRKMLEKCAMTALSSKLISQQKVFFAKMVVDAVMMLDELLQLKMIGIKKVQGGALEESRL<br>VAGVAFKKTFSYAGFEMQPKKYKNPKIALLNVELELKAEKDNAEIRVHTVEDYQAIVDAEWNILYDKLEK<br>IHQSGAKVILSKLPIGDVATQYFADRDMFCAGRVPEEDLKRTMMACGGSIQTSVNALIPDVLGRCQVFEE<br>TQIGGERYNFFTGCPKAKTCTIILRGGAEQFMEETERSLHDAIMIVRRAIKNDSVVAGGGAIEMELSKYL<br>RDYSRTIPGKQQLLIGAYAKALEIIPRQLCDNAGFDATNILNKLRARHAQGGMWYGVDINNEDIADNFQA<br>FVWEPAMVRINALTAASEAACLIVSVDETIKNPRSTVDAPAPAAGRGRGQGRFH
**Cct71P**		Rn				CCTeta pseudogene 1	1	q43	+	1	216186823	216188454		MMPTPVILLKEGTDSSQGIPQLVSNMSACEVIAEAVRTTLGPHGMDKLIVDGRGKATISN<br>DGATILKLLDVVHPAAKTLMDIAKSQDAEVGDGTTSVTLLAAEFLKQVKPYVEEGLHPQI<br>IIRALLTATQLAVNKIKEIAVTVKKQDKVEQRKMLEKCAMTALSSKLIFQQKVFFAKMVV<br>DAVQGGALEESRLMAGVAFKKTFSYAGFEMQPKKYKNPKIALLNVELKLKAEKDNAEIRV<br>HTVEDYQAIVDSEWNILYDKLEKIHQSGAKVILSKLPIGDVATQYFADRDMFCAGRVPEE<br>DLKRTMMACGGSIQTSVNALIPDVLGRCQVFEETQIGGERYNFFTGCPKAKTCTIILHGG<br>AEQFMEETERSLHDAIMIVRKAIKNDSVVAGGRATEMELSKYLWDYSRTIPGKQQLLIGA<br>YAKALEIIPRQLCDNAGFDATNILNKLRARHAQEGMWYGVDINKEDIADNFQAFVWEPAM<br>MCINTLTAASEAACLIVSVDETIKNPRSTVDAPAPAAGRGRGQGRFH
**Cct72P**		Rn				CCTeta pseudogene 2	5	q36	+	3	145306136	145307710		MPTPVILLKEGTDSFQGFPQLVTPVPAKRLLRLELGRCGTDKLIVDGQGKATISNGGATV<br>RKSLDVVHPAAKTLVLLPNPKTLRLVMAPPHSELISGRKVFAKMAAYAVMMPDEVLQLTG<br>AGAAFEKTFSYAGFEMQPEKSKNPKSALLNVELERKAEKDDAEIRVHTGEDFQAMMDTKW<br>SIRYDKLEKTHQSGAIVSKLPIGDVAIQYSADRDVFCADCKPEEDLKKTMTACGGSIQSS<br>VDALTPDVLGYCQVFEETPIGGERHNFFTGCPKAKTCTSLLHGGAEQFMEEAERSLHDAI<br>ITVRRAIKNDSVAAGGGLCDNAGFDATNILNKLQAGHAQGGMWYVVDINNKDIAQNLQAF<br>VWEPASEAACLIVSVDETIKSPRSTVDAPGPAAGG
**Cct73P**		Rn				CCTeta pseudogene 3	9	q13	+	3	19556657	19557897		MTVKKQDKVEQRKMLEKSAVTAPSSELISQQKVCFAKVVSDAVMMLDETLSLGLGLKGSP<br>RSIRTLLNVELKLKAQKDNVETTVHTADEYKEIVDDKQNILDGKLVIGSSSCCRLEEDLK<br>RLMNVKALIPDVLGHSQVSEETQIGGERCNFFILRPRHVASSSVVALSSLSLHGAIMFVR<br>SVIKYDS
**Cct81P**		Rn				CCTeta pseudogene 1	11	q12	+	1	43940890	43941006		MNKMVINHLEKLFMT
**Cct82P**		Rn				CCTeta pseudogene 2	3	q12	+	1	32962674	32962763		GDKVTNMALHYANKGNTML

**Hspd1**	LOC684747	Rn					9	q31	-	11	53884643	53894161		MLRLPTVLRQMRPVSRALAPHLTRAYAKDVKFGADARALMLQGVDLLADAVAVTMGPKGRTVIIEQSWGSPKVTKDGVTVAKSIDLKDKYKNIGAKLVQDVANNTNEEAGDGTTTATVLARSIAKEGFEKISKGANPVEIRRGVMLAVDAVIAELKKQSKPVTTPEEIAQVATISANGDKDIGNIISDAMKKVGRKGVITVKDGKTLNDELEIIEGMKFDRGYISPYFINTSKGQKCEFQDAYVLLSEKKISSVQSIVPALEIANAHRKPLVIIAEDVDGEALSTLVLNRLKVGLQVVAVKAPGFGDNRKNQLKDMAIATGGAVFGEEGLNLNLEDVQAHDLGKVGEVIVTKDDAMLLKGKGDKAHIEKRIQEITEQLDITTSEYEKEKLNERLAKLSDGVAVLKVGGTSDVEVNEKKDRVTDALNATRAAVEEGIVLGGGCALLRCIPALDSLKPANEDQKIGIEIIKRALKIPAMTIAKNAGVEGSLIVEKILQSSSEVGYDAMLGDFVNMVEKGIIDPTKVVRTALLDAAGVASLLTTAEAVVTEIPKEEKDPGMGAMGGMGGGMGGGMF
**Hspd1P**		Rn				GROEL pseudogene 1	14	q22	+	1	107651987	107653696		ASTTHSPSPNETSVSGTGSSSHYAKDVKFGADARALMLQGVDLLADAVAVTMGPKGRTVMIEQSWGSPKVTKDGVTVAKSIDLKDKYKNIGAKLVQDVANNTNEEAGDGTTTATVLARSIAKEGFEKISKGANPVEIRRGVMLAVDAVIAELKKQSKPVTTPEEIAQVATISANGDKDIGNIISDAMKKVGRKGVITVKDGKTLNDGLEIIEGMKFDRGYISPYFINTSKGQKCEFQDAYVLLSEKKISVHYVDGEALSTLVLNRLKVGLQVVAVKAPGFGDNRKNQLKDMAIATGGAVFGEEGLNLNLEDVQAHDLGKVGEVIVTKDDAMLLKGKGDKAHIEKRIQEITEQLDITTSEYEKEKLNERLAKLPDGVAVLKVGGTSDVEVNEEKDRVTDALNATRAAVEEGIVLGGGYQKIGIEIIKRALKIPAMTIAKNAGVEGSLIVEKILQSSSEVGYDAMLGDFVNMVEKGIIDPTKVVRTALLDAAGVASLLTTAEAVVTEIPKEEKDPGMGAMGGMGGGMGGG
**Hspd2P**		Rn				GROEL pseudogene 2	14	p11	+	1	42554408	42556112		MLRLPTVLCQMRPVSRALAPHLTQAYAKDVKFGADARALMLQGVDLLADAVAVPVGPKGRTVTLEQSWGSTKVTKDGVTIAKLIDLKDKYKNIGIKLVQDVANDTNEEAGDGTTTATVLARSIAKEGLEKISQGAEPVGIRRGVMSAVDAVIAELKKQSKPGTTPEETAQVATTSANGDKDTGNIISDAVKKVGRKGVITKISSVQSIVPALEIANAHRKPLVTIAEDVDGEALRTPVLNRLKVGLQVVADKAPGFGDNRKNQLKDMTIATGSAVFGEELNLNLEDVQAHDLGKVGQVIVTKDDAMLLKGKGDKAHIEKILGFIKPANEDQKIGIEIVKRALKIPAMTIAKNAGVEGSLIVEKILQSSSELVVRTALLDAAGVVSLLTTAEAVVTEIPKEEKDPGMGSMGGMGGGMGGG
**Hspd3P**		Rn				GROEL pseudogene 3	17	q12.1	+	2	58629782	58633066		PKVTKDGVTVAKSIDLKDKYKNIGAILVQDVANNTNEEAGDGTTTATVLAWSIAKEGLEKISKGANPVEIRRGVMLAVDAVIAELKKQSKPVTTPEEIAQVATISANRDKDTGNIISDAVKKVGRKGVITVKDGKTPNDELEIIEGMKFDRGYISPYFINTSKGQKCEFQDAYVLLSEKKISSVQSTVPALEIANAHRKPLVIIAEDDDAEALSTLVLNRLKVGLQVVAVKAPGFVDNRENQLKDKAITIGGTVFGEEEKHVQEVTEQLDVTTSEYEKEKLNERLAKLSDGVAVLKVGGTSDVEVNEKKDRVTDALNATRAAVEEGIVLGEGCALLRCIPALDSLKLANEDQKIGIEIIKRALKIPAMIIAKNADFEKMVEKGIIDPKKVVRTALLDAAGMASLLTTAETVVTEIPKEEKDPGMGAMGGMGGDMGGG
**Hspd4P**		Rn				GROEL pseudogene 4	14	p11	-	2	41523338	41525050		ALITHSPLPDQTSVSGTGSSSHSGLCQRCKIWCGSQALMLQGVDLLADAVAVPMGPKGRTVIIEQSWGSPKVTKDGVTVAKSIDLKDKYKNIGTELVQDVADNTNKEAGDGTTTATVLARSIAKEGLEKISQGANPVEIQRPVMLAVDAVIAELKKQSKPVTTPEETAQVATTSANGDKDTGNIISDAVKKVGRKGVITFDRGYISPYFINTSKGQKCEFQDAYVLLSEKKISSVQSIVRALEIANAHRKPLVTIAEDVDGEALSTPVLNRLKVGLQVVADKAPGFGDNRKNQLKDMTIATGSAVFGEEELNLNLEDVQAHDLGKVGQVIVTKDDAMLLKGKGDKAHFEKRNQEITEQLDVTTSEYEKEKLNERLAKLPDGVAVLKVGGTSDVEVNEEKDRVTDALNATRAAVEEAIVLEGTVLCFVIDPTKVVRTALLDAAGVVSLLTTAEAVVTEIPKEEKDPGMGSMSGMRGAVGGG
**Hspd5P**		Rn				GROEL pseudogene 5	1	q35	-	2	179041398	179043070		LLADAVAVPMGPKGRTVIIEQSWGSPKVTKDGVTVAKSIDLKDKYKNIGATLVQNVANNTKRLGMHHHCYCSGRSIAKEGFEKNSKGANPVEIRRGVMLTVDAVIAELKKPSKPVTTPEEISQVATISANGDKDIGNIISDAMKKVGRKGVIAVKDGKTLNDELEIIEGMKCDRENMSPYFINTSKGQKCEFQDAYVLLSEKKISSVRSIVPALEIANAHRKSLVIIAEDVDGEALSTLVLNRLKTGLQAVAVKAPGFGDNRKNQLNDTVIVTGGAVFGEERLNLNLEDVQAHDLGKVGEVIVTKDDAMLLKGKGDEAHIEKCIQEITEQLDVTTSEYEKEKLNERLAKLPDGVAVLKVGGTSDVDVEVNEKKDRVTDALNATRAAVEKGIVVEGSLRVQKILQSSSEVGYDAMLGDFVNMVRKGIIDPTKVVRTALLDAPGVASLLSTAEAVVTEIPKEEKDPGMGAMGGMGGGVGGG
**Hspd6P**		Rn				GROEL pseudogene 6	20	q11	+	4	30691244	30692932		HSPSPDETSVSGTGSSLHSGLCQRVKFGADAPALMLQGVDLLANAVAVTTGPKGRTVTTEQSWESFKVTKDGATVAKSIDLKDKYKNNGAKLVQDVANNTNEEAEDGTTTATLLAWSIAKEGFEKISKGANPVGIRRGVMVADDAVVAELKKQSKPVTTPEEIAIISTNGDKGIGNIISDATKKVGRKGYISPYFINVSKGQKCEFQDAYILLREKKISSVRSIVPALEIANAHRKPLVIIAEDVDGEALSALVLNRLKVGLQVVAVKAPGFEDNRKNQFKDMAIATGGAVFVEEGLNLNLEDVQDHDLGKVGEVIVTEEDAMFLKGEGDKAHIEKHIQEITEQPDVTNEYEKEKLNERLAKLPDGVAVLKVGGTTAVEEGIVLGGGCALFHCIPALHSLKPANEDQKICIEIIKRALKIPAMTVAKNAGVEGSLIVEKILQSSSEVDYDAVLGDFVNMVERELLIQKGSVVTDISKEEKDPGKGAMGGIGGGMGGG
**Hspd7P**		Rn				GROEL pseudogene 7	1	q22	-	2	103951550	103953587		LPTVLHQMRPVSRALTPHLTWAYAKDVKYGADARASMFQGVVLLVHAVAVTIWPKGKTVIIDQSWGSPKDAPNNTNEEAGDGTTTATVLAPSVAKEGFEKISKGANPVGIRRGMMLAVDAVTAELKKQSKPVTTPEEIAQVATISANGDKDIGNIISDAMKVGRKGVIKVKDGKTLNNELEIIEGMKCDRGYIFPYFINTSQDQKCEFQMPMFYVDGEALSTLGLNRLAVSLQLVAVRVQGFGDNKKNQLKDMAIATGDGVAVLKVGGTSDVEVNEKKDRVTDALSATRAAVEEGISANEDQKIGIEIIKRALKIPAMTIAKNTGIEGSLIVDKNSTEFLRSWGGPLANYSRSFSDIPKEEKDLGMDTMGGMRGGMGGG
**Hspd8P**		Rn				GROEL pseudogene 8	13	p13	+	1	3553285	3554991		PTVLHQIRPVSRSLAPHLTRSYAKDIKYGVDARALMLQGVDLLVNAVAVTMGPKGRTLIIEQSWGSPKDVANNTNEEAGNGTTTATVLAWSIAKVGFEKFSKGANPVEIRRGVMLTVDAVIAEFKKQSKPVTITEEIAQVVTISANGDKDFGNIISGAVKKVGRKGVITVKDRKTLNDELETIEGMKFDRGYIFPYFSNTSKGQKCEFLNAYVLLSEKKISSVKSTVPALEIVNAHWKPLVEIAEDVDGEALSIVVLNRLKVGLQVVAVKAPGFGDNRKNQLKDMAFTAGYTVFGEEGLDLNLKDVQAHGLGKVEEVIFTNDDAMLLKGKGDKVHIEKCIQEITEQLDITTSVKLGGTSDVEVNEKKGRVTDAIDATRAAVEEGIVLGGGYSLLWCIPVLDSLKPANKDQRIGIEIIKRALNIPAMMIAKNTGAEGSLIVEKILQCSSEVSYDAMLGDFVNMIEKGTIDPKKGCKNCFTGCC
**Hspd9P**		Rn				GROEL pseudogene 9	2	q43	+	1	234250749	234252288		PSPDETVSRALAPLLTRAYGKDVKFGVDARASVLQGSWGSLKVTKDGVTVAQSIDLKDKYKNIGAKVVQDVTNNPNEEAGDVTTTATLLAQSIAKEGFLKIGKGVNPVEIQRGVMLAVDAVIAELKKQSKPATTPEEIAQVARISANGDKDIGDIISDAMKKVGRKGVITVKDGKTQNDELEIIEGMKFDRGYISPYFINTSKFIVSALEVANAHGKPLVIIAEDVDGEALSALVLNRLNVGLQVVAVKAPGSAVFGEERLNLNLEDVQARDLGKVGEVIVTQDDAMLLKGKGDKGHTENCIQEITEQLEITTSESEKEKLNERLANLSNGVSPADKDQKIGVEIIKRALKIPAVTIAKNAGVEGSLIVEKILQSSSEIGYDAVLGEFVNM
**Hspd10P**		Rn				GROEL pseudogene 10	5	q24	-	4	76955304	76956924		NAVAVTMESKRTVLIEQSLGSPKVTKDGVTVAKKSKSRETWRTAVDAVIAELKKQSKPVTTPEEIVQVATISANGDKDIGNIISDSTKVGRKGAITVKDGKTLNDDLEIIEGMKFDRRCISLYFITTSKGQKCEFQDAYVLLSEKKISSVQSIVPALEIANAHRKPLVIIAEDVDGEALSTLVLDRLKVGLQVVAVQSSRETLEMSLSPKMMRCFWKEKLTLKNVKGKGDKAHIEKHVQEITEQLDITTSEYKKEKPNEWLGKLADGVAVLQVGRTSGMEASEKKDRVTDAFNASRATFEEGIVLGGGCALLRCIPALDSLKPANEDQNIGIEIIKRALKIPAVTIAKNAILIVEKILIIDPTKVVRTVLLDAAGVASLLTTAEAVVTEIPKEKKDPGMGAMEGWEGGMRDG

**Hspd11P**		Rn				GROEL pseudogene 11	6	q12	+	5	19170317	19172008		MLELNTVLHQVRPVSWTLFPHLTLAYAKDTQFGTDAKALLFQDIDLLADVVVVTKGPKGR<br>TVIIEQSWGSPKVTKDGITIRKSIDLNDEYKNNRANLVQNEGFQKINKGANPVEIQRVKD<br>GKILNDELEIIEGMKFDRGYISSYFINTSKGQKCEFQDGYVLLSEKKFSSVQSIVPSLEI<br>ANGSPWKIQFKDTAISTGGAVFGKEGLNLNLEDVQAHDLGKVGDVIVTKDGSLLVEKILQ<br>SSSEDGYDVMLRDFVNMVEKGIFDPTKVRRTALLAAAAVDSLLTIAEVKVTKIPNEEKNS<br>GMGAMGGMVGGMG
**Hspd12P**		Rn				GROEL pseudogene 12	2	q42	+	1	220584917	220585779		MLRLPTVLLKMRPMSQALAPHLTWVYAKDVKFGAAKLVQDVANNTNKEAGDGTTTATVLA<br>WSIAEEGFEKISKGVNPVEIWRGVMLADDAVIAELKKQSKPVPTPEEIAQVATISANGDK<br>DIGNIISDAMKKVGRKGAITVKDGKTLNDELEIIEGMKFDRGYISPYFINISKCHKCEFQ<br>DAYVLLSEKNISSVQTIVPALEIANAHRKPLVIIAEDVDGEALSTL
**Hspd13P**		Rn				GROEL pseudogene 13	9	q22	+	4	43887785	43896197		MVLHQMKPVSRALTLPLTRACIKDIKFGADARALMLQGIVNLLAIAIVVIMGSKGRTVII<br>EQSWRSPKITKDWFSVAKSIDLKDKYKIMELNLFRMLPITQTKRLGMAPPLSLFWQVEIQ<br>RDVMSAVDAVITELNKQSKPVTAPEEIAQVATISANVEKDIGNIISDAMKKSIVSALEIA<br>NAHQSSLVIITEVKAPGFGDNRKNQFKVMAITIGGVAVGPLNLNLEDVQDHDLGKVGEII<br>VTKDDAILLKGIGDKAHIEK
**Hspd14P**		Rn				GROEL pseudogene 14	5	q32	-	4	109039079	109040435		AVIAELIKQSKPVTAPEEIAQVASISTNGDKDIGNIISDAIEKVGRKGVITVKDEQTLMD<br>KLEIIEAIVPALEVANTRRKPLLIMVEDVDGETLSTLVLNRLEVGLQVVSVKALGFRDNR<br>KNQLKGVSIATGGEVFGEGRLNLNLEEVQAHDLGKVGSVTVNRDGTMFLKGKGDKAHIEK<br>CIQGITEQLDITTSEYEKGKLNKLLAKLSDRVAVWKDKWYGTAVEESIILTEGWVLLQCI<br>PVLRSLKPANGDQKTAEAVRTEIPKEENDPRIGAMDGKAVGMG
**Hspd15P**		Rn				GROEL pseudogene 15	4	q21	+	5	37890620	37892172		SLALAPHLTQAYAKYVKFHTDPEALMLQGVVLIIDAVAVCYNGIKGKNSGHWIDWESPRH<br>RDGSTTAAVLACSIAKGGFDKSSKGANPVQIQRKVTLAVDAVIAELNQSKPVTTPKKLLS<br>GKKISSAQPMVHALEIANAHPKALVIITEDVVRDTLSTLVLNRLKVPSDGVPVLKLGGTS<br>DVEVGEKNGRITDVLSFIRAAVEEGIVLGGGCLRKFCRFPQKLVMMLFFFEIFVNMVENR<br>IIDPTKVVRISILDAAGVASLLIATKAVMTEIPKEETDPGT
**Hspd16P**		Rn				GROEL pseudogene 16	X	q32	+	1	110646161	110647153		YVLLSGKKISSVQIIVPTFEIANAHRKPLVIIAEDVDGEALRTLVLNRLKTILQVVTVKA<br>PGFAENRKNQFKDMAIATGGVVFGEEGLNLNLKDVQPQDLREIGEAIVTKDDAILLKGKV<br>TKLTLKNKKLNERLAKLSDGIAVLKFGGTCDIEVNEKKDRVTDDLNFPTAAVQEGIVLGR<br>GCALLRCIPALNSLNLLMKNLSVESSLLVEKILQSSSEVAYKAMVVDFVNMVEKGIIDPT
**Hspd17P**		Rn				GROEL pseudogene 17	X	q31	+	4	84112821	84114244		KSILAQFVQDVANNTNKGTGDGTTTITVLAWSIANEGFENVSKGANPVEIQGSMMLTVDA<br>VIVELKTQSKSMTTPEEIALLATISANVKCNGESIGNIISDVTKKVGRKGCHDRKDGKAL<br>SDELEVSESMKFDRGYISIYQHIKRSKCDFQDAYVLLSTKKVSSPLNRKYTCFEQTQSWS<br>SGCSSQSSKVWGQEEPLTDMAIATGGGVFGEEGLNLNLEDAKAHHLRKVGEVIVTKDDAV<br>LLKGKDGVAVLKVGEISDVEVNKKKDRVTDALSATRASVKEDIVLVGAALHFSSSSDVGY<br>DAILRDFVSMEKGINNRSNKGCKNCFTGYCWGDQSCSDRNS
**Hspd18P**		Rn				GROEL pseudogene 18	9	q22	-	1	46394084	46394824		GGAVFGEEGLNLSLEDVYAHDLGKVGEVNVNVTKDDATLLKGKGDKTDIEKCIQEITEQL<br>DIPTGEYEKESWMSDLQNFQTEAVLKAGGQVTLKCVRRRQGYTCSQCYRSSCGRRGCALL<br>RCIPVLDSLKPANEDQKIGIEITKRAGKIPAMMTAKNEGVEGSLIVEKILQSSSEVGYEA<br>MLGDFVNMAEKGIIDRTKVVRTTLLDAGVASLLTTTEAVVTEIPKEEKDPGMGAMGGMGG<br>GMGGG
**Hspd19P**		Rn				GROEL pseudogene 19	20	q13	-	1	52019335	52019963		KGDKAHIEKCTQEITEQLDITTKEGVADALKATRTAVEEGIVLGGGCAQVQCIPALDSLK<br>PANEDQKIGIEIIKRALKIPVITIVKNAVVEGSLIVEKILQSSPEVGYGAMLGDFVSMVE<br>KGIIDPTKFVRIALLDAAEVASLLTIAEAVVTEIPEEEKDPGKGSLGGMGWGTG
**Hspd20P**		Rn				GROEL pseudogene 20	15	p16	+	2	8089404	8090167		MGPKRRTVIIEQNRKSPKATKDGVTVAKSISVKDKHKTIRDKLVQDVANNTKNRLEMAPP<br><br>LPLFRHSLLPRRALRRSAKGKSREVRKTLKDQKIGREIIKRALKIPAMIIVKSAGLEGSL<br><br>IVEKILQSSSEVGYDSMLGDFVNMVEKGIINPKKILRAALPNAAGMASLLTTLEAVVT
**Hspd21P**		Rn				GROEL pseudogene 21	X	q35	+	1	132806121	132806537		GIVLGVGCALLQCMPALNSLKATNEDQQIGVEIIKRALKIPAMMIAKNAGVEGSLIVVKI<br>LQSSSEVGYDAMLGDFVNMMEKGIIDPTNVVRTALLDAAGVASLLTTVEAVVPEIPKEEK<br>DPGMGAMGGMGGGMEGG
**Hspd22P**		Rn				GROEL pseudogene 24	17	q12.1	-	1	58562739	58563142		KEKLNERLAKLSDGVAVLKVGGPSDVEVNEKKARVHRCSQCSKTAAVEEGIVLGEGCALL<br>RCIPALDSLKLANEDQKIGIEIIKRALKIPAMIIAKNA
**Hspd23P**		Rn				GROEL pseudogene 25	2	q31	-	4	151675083	151676174		MKEIGRKSVITVKDGKPLNNELEIDAYFLLSEKKISSVPFIVPALEIGNAYWKSLVIIAE<br>DNDGKAIRSLVLNMLKFAPQVVVIRALGFGDNRQNHMAIVSGAHVLRKRMHVLKDHVLTK<br>DDDKLLKEKGDKLKLKNVFKKSLSSWTSQLFLRHWHDAMLRDFVNMWKGEIIDPTKVVRT<br>TLLDAVEMALLVTTAET
**Hspd24P**		Rn				GROEL pseudogene 26	15_random		-	1	414366	414686		QKICVESIKRALKIPAMTIAKNAGIEGSLIVEKILQSSSEVGYDAMLGDFVNMVEKGIID<br>LTKVVRTALLDAAGVASLLMTAEAVVTEIPKEEKEPAMGAM
**Hspd25P**		Rn				GROEL pseudogene 27	2	q22	-	2	75608618	75611557		SIEPALKIANAHWKPLVITADDIDKKSLSTLDLTRLKLLKKVLFLEGPELCFRTLKIPAM<br>KFSKNVGVEESLTVDKILQRSSEVGYNAMLEDFVNMVEKGIIDPTKIIRLLYWMLL
**Hspd26P**		Rn				GROEL pseudogene 28	6	q23	-	1	80028738	80029070		MTIAKNAGVERSSIVEKILQNSSEAGYDIMLGDFVNMVEKGIIDPTNILRTALLGASGVT<br>SLLTTAKAVVKEISKEEKDPGMDAMGGMGGGMG
**Hspd27P**		Rn				GROEL pseudogene 29	7	q11	+	2	11777078	11777682		IFPYFNDTSKDDRCELQDAYFLLSEKKISSVPFIVPSLEIGNAYWKSLVIIAEDDDGKAI<br>SSLVLNMHLSEYVKERLNEKLANFQM
**Hspd28P**		Rn				GROEL pseudogene 30	2	q16	+	1	62800517	62800732		SSSEVGYEAMLGDFVNMVEKGIIDPQKVVRTASLDATGVASLLTTSGAVVTEIPKEEKDP<br>GV
**Hspd29P**		Rn				GROEL pseudogene 31	5	q12	-	5	14696828	14698283		MLPITQMKRLGSGSPIATLLSRRALRRLAKATVPEEIVQVAMISANGDEDIGNIICDAKK<br>MVGRTMLTRSPDALSSVGLNRLIVGIQVITAKSPRFGGNRKNQTKDMAVTPGGAVLKERE<br>WNSNLKDVQAHDLGMSVKRKNYEWLMKASGGAAVLKVGGSSDAEENEEKDCYGYSECFTS<br>SCSEKKKKKRNVKNAVVEGTLIVKEILQSSAEVGYDAARWKEESLIQQR
**Hspd30P**		Rn				GROEL pseudogene 32	9	q31	-	1	53891997	53892110		MLRLSTVLRQMRPVSRALAPHLTRAYAKDVKF
**Hspd31P**		Rn				GROEL pseudogene 33	4	q22	+	1	53483173	53483310		ASLLTTAKAVVTEIPKEEKDPRMGAMGRM
**Hspd32P**		Rn				GROEL pseudogene 34	4	q13	-	1	30834360	30834470		MVEKGIIGSTMVVRTALLDTPGVASLLTTTEAV

**Tcp1**		Rn	NP_036802	NM_012670.1		Cctalpha<br>(real gene)<br>vert	1	q12	-	12	42103629	42111145		MEGPLSVFGDRSTGEAIRSQNVMAAASIANIVKSSLGPVGLDKMLVDDIGDVTITNDGATILKLLEVEHP<br>AAKVLCELADLQDKEVGDGTTSVVIIAAELLKNADELVKQKIHPTSVISGYRLACKEAVRYINENLIINT<br>DELGRDCLINAAKTSMSSKIIGINGDFFANMVVDAVLAVKYTDIRGQPRYPVNSVNILKAHGRSQIESML<br>INGYALNCVVGSQGMLKRIVNAKIACLDFSLQKTKMKLGVQVVITDPEKLDQIRQRESDITKERIQKILA<br>TGANVILTTGGIDDMCLKYFVEAGAMAVRRVLKRDLKRIAKASGASILSTLANLEGEETFEATMLGQAEE<br>VVQERICDDELILIKNTKARTSASIILRGANDFMCDEMERSLHDALCVVKRVLESKSVVPGGGAVEAALS<br>IYLENYATSMGSREQLAIAEFARSLLVIPNTLAVNAAQDSTDLVAKLRAFHNEAQVNPERKNLKWIGLDL<br>VHGKPRDNKQAGVFEPTIVKVKSLKFATEAAITILRIDDLIKLHPESKDDKHGGYENAVHSGALDD

Table S7. Gene and pseudogene information, including start and end position, chromosomal location, strand, number of exons, GenBank accession number for functional genes, and Ensembl or Pseudogene.org ID for pseudogenes.
